# Weight loss aggravates obesity-induced hypothalamic inflammation in mid-aged mice

**DOI:** 10.1007/s11357-025-01933-x

**Published:** 2025-10-17

**Authors:** Alon Zemer, Yulia Haim, Alexandra Tsitrina, Vered Chalifa-Caspi, Habib Muallem, Yair Pincu, G. William Wong, Uri Yoel, Alon Monsonego, Assaf Rudich

**Affiliations:** 1 Department of Clinical Biochemistry and Pharmacology, Faculty of Health Sciences, Ben-Gurion University of the Negev, Beer-Sheva, Israel; 2 The Shraga Segal Department of Microbiology, Immunology and Genetics, Faculty of Health Sciences and School of Brain Sciences and Cognition, Ben-Gurion University of the Negev, Beer-Sheva, Israel; 3 Ilse Katz Institute of Nanoscale Science and Technology, Ben-Gurion University of the Negev, Beer-Sheva, Israel; 4 Department of Health and Exercise Science, University of Oklahoma, Norman, OK USA; 5 Harold Hamm Diabetes Center, Oklahoma City, OK USA; 6 Department of Physiology, Pharmacology and Therapeutics, Johns Hopkins University School of Medicine, Baltimore, MD USA; 7 Soroka University Medical Center, Beer-Sheva, Israel; 8 Faculty of Health Sciences, Ben-Gurion University of the Negev, Beer-Sheva, Israel

**Keywords:** Weight loss, Aging, Obesity reversal, Hypothalamic inflammation

## Abstract

While neuroinflammation is an established response to both weight gain and aging, the hypothalamic neuroinflammatory early response to weight loss (WL) remains unknown, particularly in mid-age. Here, we questioned whether WL-induced rapid restoration of normoglycemia is mediated by the resolution of hypothalamic microgliosis in mid-aged mice. Mid-aged (1 year) mice were fed normal chow (NC) or a high-fat diet (HFD, 8 weeks), and WL was induced by a 2-week switch back to NC. Key findings were compared to young (7 weeks) mice. Mid-aged WL mice lost within 2 weeks 54% of their excess body weight, somewhat less than young mice (68%). Whole-hypothalamus RNA sequencing revealed ~ 4 times more differentially expressed genes (DEGs) in mid-aged than in young mice (2419 vs. 670, respectively). Nevertheless, in both ages, WL did not reverse but rather aggravated the transcriptomic response to HFD in ~ 80% of DEGs, uncovering downregulated metabolic (oxidative phosphorylation, thermogenesis) and neurodegenerative pathways, particularly in mid-age. Consistently, in the arcuate nucleus (ARC), HFD-induced increase in microglial number and volume further increased in weight-losing mid-aged mice. Consistently, only in mid-aged mice, WL further increased HFD-induced mean single-microglial volume in ARC, corresponding to increased pNFκB-(p-p65) nuclear staining. Despite aggravated hypothalamic and microglial changes, mid-aged mice fully normalized impaired glucose tolerance by early WL. Adipose tissue inflammation was not resolved in mid-aged WL mice, and adipose tissue crown-like-structure density correlated with ARC microglial cell volume (Rho(ρ) = 0.691, *p* = 0.001). In conclusion, in mid-aged mice, obesity-induced hypothalamic and microglial changes were aggravated during the early response to WL. The resolution of these obesity-induced changes is not required for the normalization of glucose intolerance.

**Supplementary Information:**

The online version contains supplementary material available at https://doi.org/10.1007/s11357-025-01933-x.

## Introduction

Investigating the pathophysiology of obesity and its complications has been greatly aided by rodent models. Yet, while in the clinic losing excess weight is the common dealing, preclinical research has focused on the triggers, mechanisms, and consequences of weight gain, leaving the sequence/dynamics of changes during WL relatively poorly characterized. This is an important gap of knowledge, since uncovering which obesity-related alterations reverse before others can exclude key causal relationships, according to the temporality principle in revealing causation (in essence, if B precedes A, A cannot be the cause for B). For example, hypothalamic inflammation, characterized by glial cells’ activation, is a rapid response to hyper-nutrition and weight gain [1]. It is documented in both rodent obesity models [1–4] and humans [5] and precedes obesity-induced glucose intolerance. Given CNS’s documented regulation of peripheral metabolism [6], hypothalamic inflammation is now considered causal to the occurrence of dysglycemia during the development of obesity [7–9]. Yet, similar analyses of how these resolve during WL (obesity reversal), vis-a-vis weight change dynamics and resolution of adipose inflammation, remain unclear. In particular, is the resolution of hypothalamic inflammation during weight loss required/causal for the normalization of dysglycemia to occur?

An additional level of complexity is how age affects obesity reversal. Here, again, a gap exists: most rodent obesity models utilize young mice, equivalent to human late teen/young adulthood, while obesity in humans is particularly prevalent among mid-aged humans [10]. Age is a major factor in weight dynamics and in the pathogenesis of obesity and its complications: Weight at 17–25 years still increases, particularly in males, at a higher rate than during young adulthood (25–45 years), a phenomenon observed in all baseline BMI percentiles [11]. Furthermore, weight loss and/or regain may be more harmful in mid-age, due to largely unknown reasons [12, 13]. Thus, the health impact of weight gain and loss in mid-age [14, 15] and dissecting out the “age factor” from the frequently longer obesity duration in older age remain poorly characterized.

In the present study, we hypothesized that reversal of obesity-induced hypothalamic changes in mid-aged mice exposed to an equal duration of obesogenic nutrition as in young mice is required for normalization of obesity-induced dysglycemia. Our results demonstrate that, contrary to our hypothesis, normalization of dysglycemia does not depend on the reversal of obesity-induced transcriptomic and microglial hypothalamic changes. In fact, mid-aged mice exhibit aggravated transcriptional and microglial changes in response to early WL, correlating with sustained adipose tissue inflammation.

## Research design and methods

### Animals and experimental protocols

**Fig. 1:**
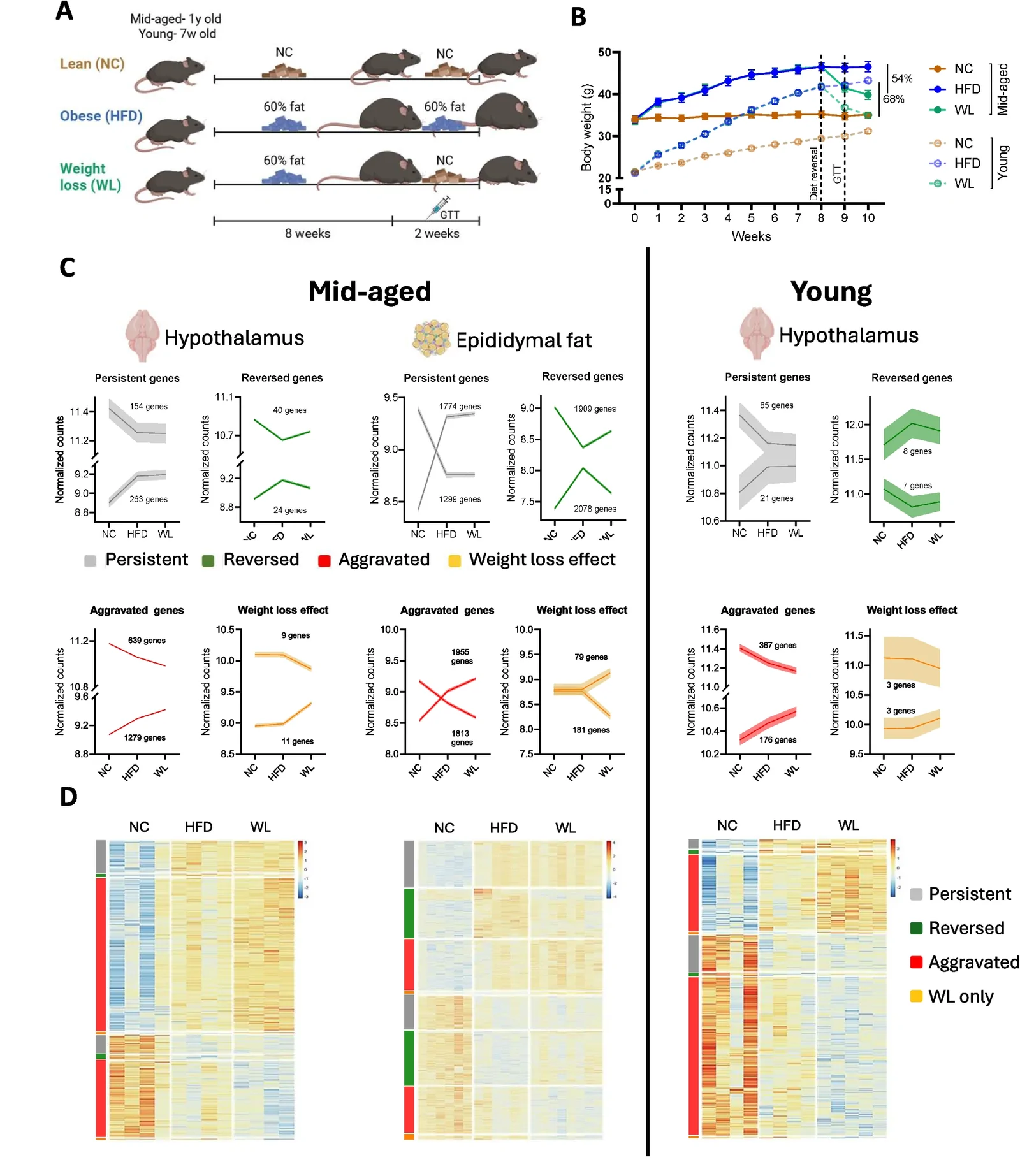
Weight dynamics reveal weight loss-associated aggravation of hypothalamic transcriptomic changes. **A** Mice were fed NC diet (20 and 18 mice per group of young and mid-aged, respectively) or HFD (42 and 33 mice per group of young and mid-aged, respectively) for 8 weeks, after which half of the HFD-fed mice were switched back to the NC diet for 2 more weeks (WL). GTT was performed in week 9. **B** Weekly body weight measurements in grams (mean ± SD). **C** Pattern of gene expression change analysis. From left to right: Hypothalami of mid-aged mice, epididymal fat of mid-aged mice, and hypothalami of young mice were subjected to whole-tissue (bulk) RNA sequencing. DEGs were defined by FDR-adjusted *p*-value < 0.05 in any contrast, as described in the methods. Pattern of change analysis identified eight initial clusters based on fold-change threshold between dietary groups, as detailed in Supplementary Methods. The *Y*-axis denotes normalized counts in log2 scale (using DESeq2 variance stabilizing transformation). Final patterns were as follows: Persistent: genes either up- or downregulated, respectively, between HFD and NC, and unchanged further between WL and HFD. Reversed: genes either up-regulated in HFD compared to NC and downregulated in the WL compared to HFD or exhibiting the mirror pattern. Aggravated: genes increased between HFD and NC and further in the WL to HFD comparison or decreased from NC to HFD to WL. Reverse only: genes unchanged between HFD and NC, but then either up- or downregulated between WL and HFD. The number of genes included in each pattern and tissue is indicated in the graphs. **D** Heatmaps of DEGs from whole hypothalami (left) and epididymal fat (middle) bulk RNA-sequencing data from mid-aged mice. Bulk RNA sequencing from young mice hypothalami (right). Of note, each heatmap panel is independently generated for each tissue/age group, so rows represent a single transcript only within the same heatmap to compare between the experimental groups

The study was approved by the Ben-Gurion University Institutional Animal Care and Use Committee (Protocol IL-69–11–2021) and was conducted according to the Israeli Animal Welfare Act (National Research Council, 1996). Five-week-old male C57BL/6 mice were purchased from Envigo RMS Ltd. (Jerusalem, Israel). Mice were acclimatized for 2 weeks, with free access to a normal chow (NC) diet (15% of calories from fat, Ssniff Spezialdiäten GmbH) and water. Animals were housed two mice per cage under controlled temperature (22 ± 1 °C), in a 12-h light–dark cycle. The dietary protocol for diet-induced obesity and early weight loss was published previously [16]. Briefly, at 7 weeks, 2/3 of the mice were randomly transferred to a high-fat diet (HFD, 60% calories from fat, D12492, Research Diets, NJ) and weighed weekly. Eight weeks after HFD initiation, half of the HFD fed mice were switched back to NC diet for an additional 2 weeks of obesity reversal (“weight loss” (WL) group), totaling 10 weeks of dietary intervention (Fig. 1A). Mid-aged (“retired breeder”) mice were purchased one mouse per cage at the age of 8 months from Envigo RMS Ltd. and maintained in the same housing conditions until the age of 12 months. Then, mice were allocated to the same dietary protocol as described above for 10 weeks. At the end of each experiment, mice were euthanized with isoflurane. The brain, blood, and gonadal (visceral) fat depot were weighed and collected.

### Glucose tolerance tests (GTTs)

For GTT, at week 9 of the dietary intervention, mice were fasted for 6 h and injected i.p. with 1.5 g/kg body weight glucose in sterile PBS solution. We used this based on the considerations detailed in [17] and [18]. Blood samples were obtained from the tail vein immediately before injection and 15, 30, 45, 60, 90, and 120 min after glucose administration. Glucose levels were measured via a glucometer (FreeStyle Freedom Lite, Abbott).

### Brain collection and immunostaining

Animals were anesthetized with isoflurane and perfused with 20 mL of sterile PBS. Brains were excised and incubated overnight in a 4% paraformaldehyde (PFA) solution and then transferred to a 30% sucrose solution for an additional 48 h. The brains were subsequently embedded in OCT compound before sectioning into 45 μm sections using a Leica cryostat. The sections were washed with PSB containing 0.05% Triton X-100 and subsequently incubated overnight at 4 °C with anti-Iba1 and anti-pNFκB antibodies, as detailed in Table S1. Next, sections were washed three times with 0.01% Tween-20 in PBS and then incubated with the appropriate secondary antibodies as detailed in Table S1. Finally, the sections were stained with DAPI for nuclear staining.

### Confocal microscopy and image analysis

Images were obtained using the Olympus FluoView 1000 confocal microscope (Olympus, Hamburg, Germany) and Zeiss LSM 880 confocal microscope at 800 × 800 and 1024 × 1024-pixel resolutions, and each image contained 20–22 steps z-stacks, with 0.75 µm step size. Whole-brain imaging was performed using ZEISS Cell Discoverer 7. Representative images of the whole hypothalamic area were created with FIJI software. Reconstitution of microglial cells was done using z-stack images. Imaris software (Oxford Instruments, UK) was used to assess the volume of surfaces, as well as the colocalization of microglial pNFkB-positive cells, and to detect nuclei. Each analysis is based on all ARC microglia (as described in Supplemental Fig. 2), with a minimum of 21 analyzed microglia per mouse.

**Fig. 2:**
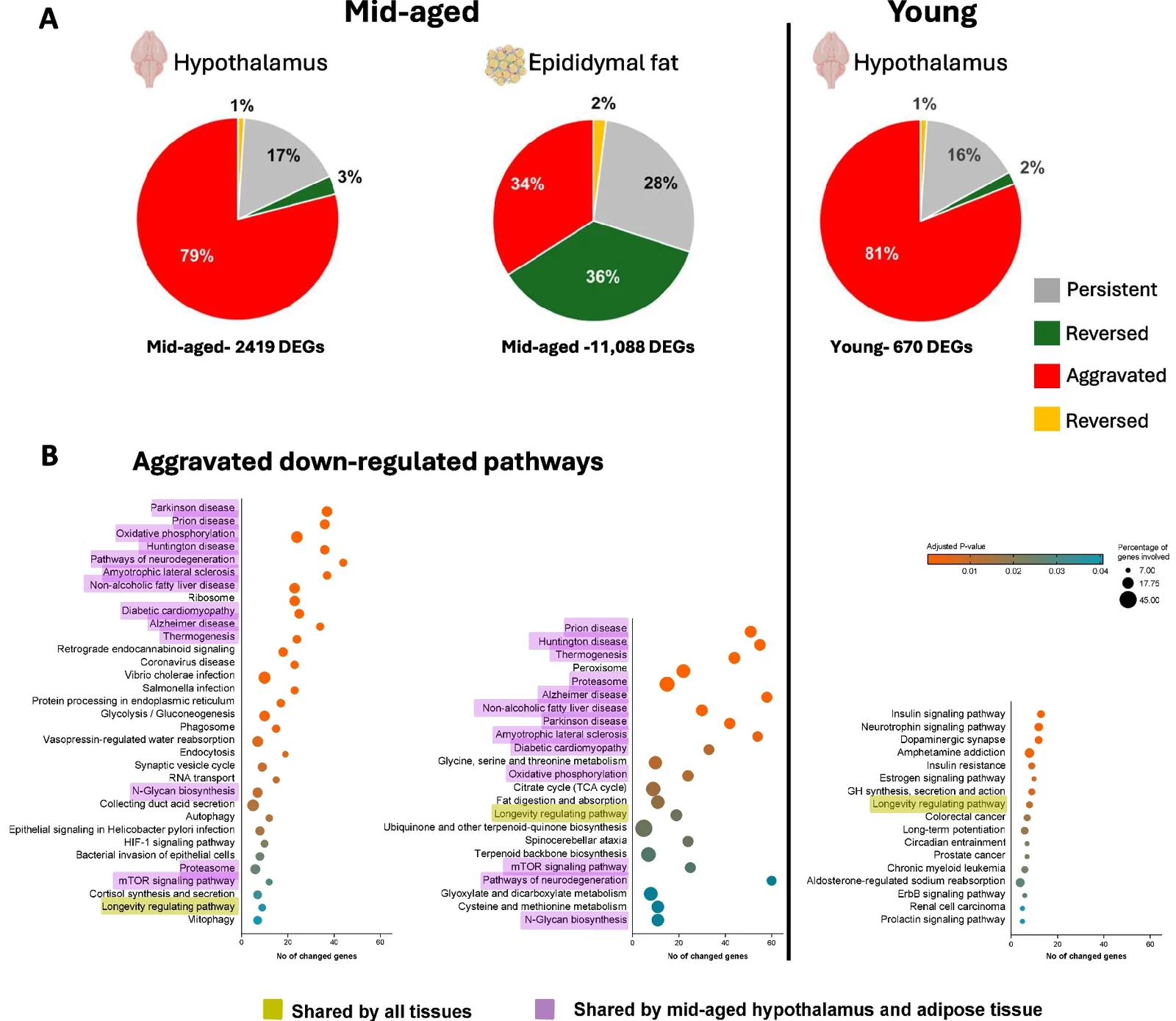
Early weight loss induces age-dependent augmentation of hypothalamic transcriptomic changes. **A** Pie charts representing the percentage of DEGs in the final four merged expression change patterns (see Fig. 1C), corresponding to the color code shown on the side of the heatmaps in Fig. 1D. **B** KEGG pathway enrichment analysis of DEGs exhibiting downregulated-aggravated pattern (down-down) as shown in Fig. 1, in mid-aged: hypothalami (*n* = 639 genes), in epididymal adipose tissue (*n* = 1813 genes), and in young hypothalami (*n* = 367 genes), respectively. The *X*-axis represents the number of DEGs involved in the pathway, the circle diameter represents the percentage of DEGs out of the total genes in the pathway, and color corresponds to the adjusted *p*-value of the enrichment. Shown are pathways with a cut-off FDR-*p*-value < 0.05 and ≥ 7% of the genes of the pathway. KEGG pathways common to the hypothalami and adipose tissue only of mid-aged mice are marked in purple, and pathways common to all tissues are highlighted in gold. The full list of significantly enriched KEGG pathways (FDR-adjusted *p*-value < 0.05) is provided in Supplemental Table S4

### Adipose tissue histology and PCR

Epididymal adipose tissue was subjected to several analyses upon its collection: (1) For histological studies, tissue was preserved in 4% PFA, processed, and embedded in paraffin. Then, 5 μm sections were stained with hematoxylin and eosin (H&E). Adipocyte size measurements were performed using QuPath software (v 0.3.2), with an average measurement of *n* = 5 fields, including an average of 563 adipocytes per mouse. Crown-like structures were manually identified and counted per 100 adipocytes. (2) For gene expression studies, 100 mg of tissue was snap-frozen in liquid nitrogen. Then, RNA was extracted using RNeasy Lipid tissue mini kit (QIAGEN, Germany), and 2 µg was reverse-transcribed with High-Capacity cDNA kit (Applied Biosystems, Thermo Fischer Scientific group, CA, USA). Taq-man probes were used to amplify specific genes (Tnf-Mm00443258_m1; Ccl2-Mm00441242_m1; C1qtnf6-Mm00511605_m1; Il1rn-Mm00446186_m1; Il10-Mm01288386_m1; Lep-Mm00434759_m1; Adipoq-Mm00456425_m1). Gene expression results were normalized to two house-keeping genes (Rplpo-Mm00725448_s1, Hprt1-Mm03024075_m1) and calculated using the ^−ΔΔ^Ct equation.

### RNA extraction and RNA sequencing data analysis

Total RNA was extracted from hypothalami and epididymal adipose tissue with RNeasy Lipid Tissue Mini Kit (QIAGEN, Germantown, MD) and quantified using TapeStation. RNA from 45 mice (four or more from each group) was sequenced using NextSeq 500 Illumina. Bioinformatic analysis was carried out as detailed in Supplementary Methods. Briefly, sequence reads were quality trimmed and filtered, aligned to the mouse genome (GRCm38), and quantified using RSEM. Statistical testing for differential expression was done using DESeq2 [19]. In each tissue/age group, DEGs were identified as genes with an FDR-adjusted *p*-value < 0.05 in at least one of the three possible between-group contrasts (HFD/NC, WL/NC, WL/HFD). DEGs were then divided into eight predefined clusters, according to their fold change (FC) between diet groups, in an analysis we termed “patterns of change analysis” (Fig. 1C, D). FC was calculated from the *z*-score of normalized Log2 expression values and expressed on a linear scale. The following trends were defined: “up” (FC ≥ 1.2), “down” (FC ≤  − 1.2), and “unchanged” (− 1.2 < FC < 1.2). Clusters with equivalent but opposite patterns were grouped into broader categories (Fig. 1C, D): “persistent” (up-unchanged and down-unchanged), “reversed” (up-down and down-up), “aggravated” (up-up and down-down), and “reverse only” (unchanged-up and unchanged-down). Enrichment analysis was carried out on the original eight clusters using Enrichr (https://maayanlab.cloud/Enrichr/).

### Statistical analysis

Statistical analyses were performed using GraphPad Prism version 10.4.1 (GraphPad Software, San Diego, CA, USA). For comparisons involving more than two groups, the Kruskal–Wallis test (nonparametric ANOVA) was applied, followed by Dunn’s multiple-comparison test to adjust for pairwise testing. For comparisons between two groups, the Mann–Whitney *U* test (nonparametric equivalent of the *t*-test) was used. When the *t*-test was used, the analysis is indicated in the corresponding figure legends. Significance was denoted in the figures as follows: *p* < 0.05 (*), *p* < 0.01 (**), *p* < 0.001 (***), and *p* < 0.0001 (****). For paired analysis, we used Wilcoxon signed-rank tests; significance was marked by “$” to indicate *p* < 0.05. Correlation analyses (Fig. 5) were conducted using Spearman’s rank correlation. Significance levels were indicated as follows: *p* < 0.075 (#), *p* < 0.05 (*), *p* < 0.01 (**), and *p* < 0.001 (***). All tests were two-tailed unless otherwise specified.

### Data resource sharing and availability

Bulk RNA-seq datasets and resources generated and analyzed during the current study are available from the corresponding authors upon reasonable request.

## Results

### Age impact on weight and hypothalamus transcriptomic changes during WL

Despite obesogenic memory, many metabolic and organ changes induced in mice by dietary obesity are fully reversible, given sufficient time back on the NC diet [16, 20]. However, the relative tissue-specific dynamic response during weight loss, particularly in mid-age following exposure to equal duration of HFD, as in the established HFD-induced obesity in young mice, has not been fully characterized. We therefore set up an ad libitum HFD and dietary obesity reversal paradigm to assess the effects of aging on weight, glycemia, and hypothalamic changes in response to early WL, before excess weight gain had been completely lost. For this purpose, mid-aged (1 year) mice were subjected to NC or HFD (60% fat) for 8 weeks. At this point, 50% of the HFD group was switched back to an ad libitum NC diet for up to 2 weeks (early weight loss group, “WL”). A glucose tolerance test was performed 1 week after the dietary switch (week 9), and brains and gonadal adipose tissues were collected at week 10 (Fig. 1A). Key findings were compared to the established young (7 weeks) HFD model.

Mid-aged mice weighed 60% more at baseline (21.36 ± 1.45 vs. 34.19 ± 3.10 g, respectively, *p* < 0.0001) and were weight-stable on NC during the entire 10-week protocol, unlike young mice (Fig. 1B). Two weeks of WL resulted in a rapid 54% loss of excess weight (defined as the difference between the mean weight of HFD/NC), in mid-aged mice, nearly significantly different than in young mice (68%, *p* = 0.056, Fig. 1B). Together, these data demonstrate similarities and uniqueness of the mid-aged HFD-obesity model: On NC, mid-aged mice are weight-stable, while young mice exhibit continuous weight gain; in response to dietary intervention, weight dynamics are similar, with mid-aged mice exhibiting lower ability to rapidly lose previously gained weight.

Next, to characterize in an unbiased manner how nutritional interventions affect mid-age hypothalamus, we used bulk RNA sequencing. Bulk RNA-seq of adipose tissue from these mice was used to assess tissue differential responses, while the hypothalami of young mice were analyzed to determine the effect of age on the hypothalami transcriptome. DEGs were defined and subjected to patterns of change analysis, uncovering four trajectories of change between NC-HFD-WL (persistent, reversed, aggravated, WL-only effect), as detailed in “Research Design and Methods” (Fig. 1C, D). Mid-aged mice hypothalami exhibited nearly 4.5-fold fewer altered genes than adipose tissue (2419 vs. 11,088 DEGs, respectively) but ~ fourfold more DEGs than in young mice’s hypothalami (670 DEGs, Fig. 1C, D, Table S2,S3). Yet, despite the difference in the number of DEGs, the hypothalami of both age groups exhibited ~ 80% genes in the “aggravated” pattern, i.e., genes that were increased or decreased in the HFD vs. NC group, and further changed, in the same direction, by WL (Figs. 1C and 2A). Interestingly, only a small percentage of genes exhibited the “reversed” gene expression pattern (2.6% and 2.2% of DEGs for mid-aged and young mice, respectively) (Fig. 2A). This suggests an age-independent, but tissue-specific, response pattern to WL, as in mid-aged adipose tissue, only 34% of equally defined DEGs exhibited the aggravated pattern, whereas 36% reversed (Fig. 2A).

Since most hypothalamic DEGs exhibited the aggravated change pattern, pathway enrichment analysis was performed on this DEG group. Curiously, in the hypothalamus, we did not detect enriched KEGG pathways among up-regulated-aggravated genes (i.e., increased in HFD vs. NC and further increased in the WL group), while in adipose tissue of the same mice, up-regulated pathways expectedly signified obesity-related adipose tissue inflammation (SupplementaryFig. 1). Yet, KEGG analysis of downregulated-aggravated genes (decrease in HFD vs. NC and further decreased by WL) revealed age-dependent differences in key pathways within the hypothalamus. Downregulated-aggravated genes in mid-aged mice were enriched in key metabolic pathways, including oxidative phosphorylation, glycolysis, and mitophagy (Fig. 2B,SupplementaryTableS4). Notably, oxidative phosphorylation, thermogenesis, mTOR signaling, and proteasome pathways were also enriched in epididymal adipose tissue of mid-aged mice, but not in the hypothalami of young mice (Fig. 2B). Interestingly, common KEGG pathways were more robustly altered (number and percentage of genes involved in the pathway and the statistical significance) in the two tissues of the mid-aged mice compared to the hypothalami of young mice (Fig. 2B,SupplementaryTableS4).

### Hypothalamic microglial changes in response to WL in mid-aged mice

**Fig. 3:**
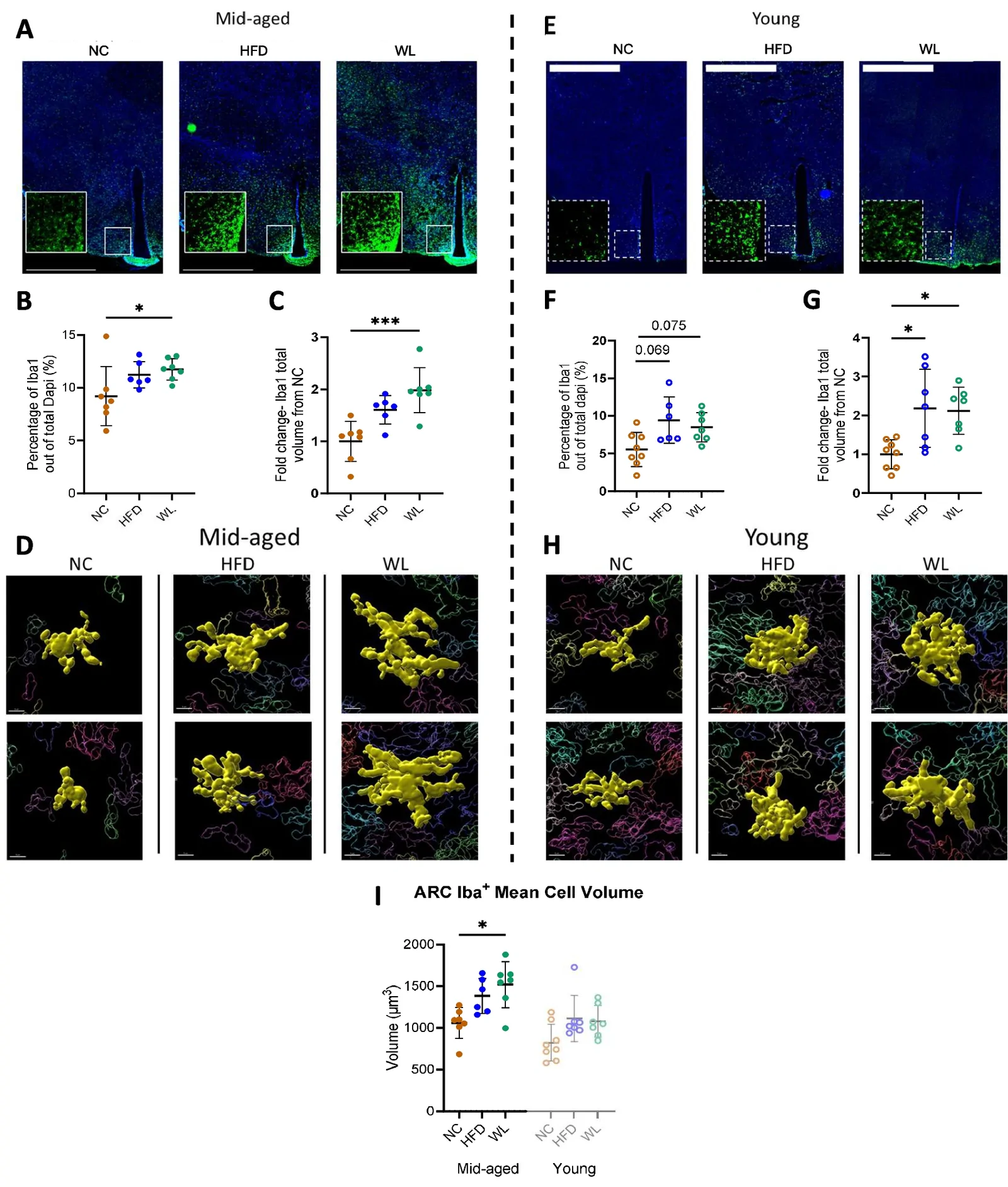
Hypothalamic microglia show age-dependent changes upon acute obesity reversal. **A**, **E** Representative images of whole hypothalami scans from mid-aged (**A**) and young (**E**) mice (DAPI—blue; Iba1—green); scale bar, 1000 µm. **B, F** Mid-aged (**B**) and young (**F**) percentages of Iba1 + cells among the total identified nuclei in the ARC of mid-aged mice (mean ± SD). **C, G** Total Iba1 volume (fold change from NC) of mid-aged (**C**) and young (**G**) mice, compared with that in the NC group (mean ± SD). **D, H** Representative images of single microglia (yellow) in the ARC of mid-aged (**D**) and young (**H**) mice. Scale bar, 8 µm. **I** Average volume of Iba1-positive cells in the ARC of mid-aged and young mice (mean ± SD). *p < 0.05, **p < 0.01, ***p < 0.001

Despite inducing multiple DEGs, whole-hypothalami RNA-seq did not uncover clear inflammatory signatures. We reasoned that this may reflect inflammation involving specific cell types and/or at sub-locations within the hypothalamus, which are masked by the bulk-tissue analysis. Since the periventricular areas of the hypothalamus include nuclei involved in energy homeostasis, such as the arcuate nucleus, we wished to characterize in detail microglia, a major immune cell type in this region. Overall observation of the entire hypothalami revealed an elevation in Iba1 expression in obese mice, which was mostly evident in the ARC (Fig. 3A, E), suggesting spatially distinct coordinates of hypothalamic inflammation. Upon WL, mid-aged mice exhibited aggravated obesity-induced microglial changes (Fig. 3A). Quantitative analysis revealed HFD-induced increases in both the number of microglia (percent of Iba1-positive cells) and the total volume occupied by Iba1-positive cells (Fig. 3B, C), consistent with previous reports in obese young mice (Fig. 3F, G) [1, 3, 5]. WL in mid-aged mice exhibited further increase in percent microglia and their volume beyond the effect of HFD (Fig. 3B, C), while young mice displayed a mild and statistically insignificant ~ 24% reversal of hypothalamic microglial staining (Fig. 3F, G**)**.

To further increase cytomorphological resolution to the single microglia level, we performed advanced image analysis using Imaris (Methods, surfaces analysis diagram demonstrated in Supplementary Fig. 2). The mean volume of individual microglia seemed to increase and showed poor reversal in the HFD and WL groups (Fig. 3D, H, I). In mid-aged mice, WL aggravated the cytomorphological changes induced by HFD, with individual cells exhibiting larger somas and thicker branches than those of HFD-fed and NC hypothalamic microglia (Fig. 3D, I). Jointly, these cytomorphometric analyses are largely consistent with the notion that, unlike weight dynamics, microglial changes are aggravated in mid-aged mice in the early response to WL.

**Fig. 4:**
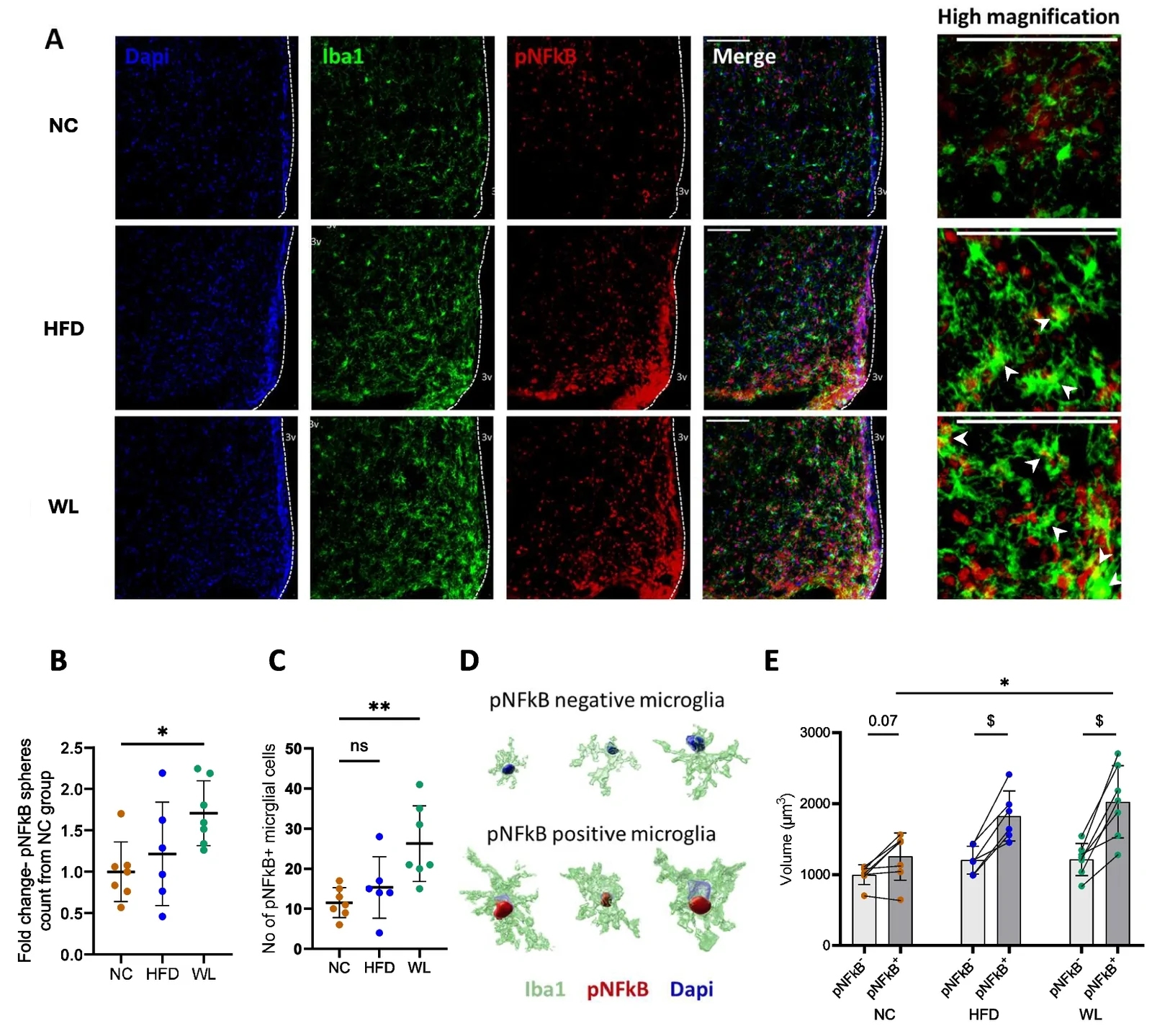
ARC microglia upregulate pNFkB expression upon acute obesity reversal in mid-aged mice. **A** Representative images of mice ARC stained for DAPI (blue), Iba1 (green), and pNFkB (red). Scale bar, 100 µm. **B** Fold change of pNFkB spheres number in the ARC of HFD and WL mid-aged mice compared with those in the NC group. **C** Number of pNFkB + microglia (mean ± SD). **D** Representative images of single pNFkB − and pNFkB + microglia from the three diet groups. **E** Averaged volume of pNFkB + or pNFkB − microglia in the ARC of mid-aged mice (mean ± SD). Connecting lines represent the same mouse. *p < 0.05, **p < 0.01 in a nonparametric ANOVA test (**B**, **C**, **E**). ^$^*p* < 0.01 in each intergroup (NC, HFD, and WL), by Wilcoxon’s signed-rank test paired analysis

To molecularly characterize the localized morphological microglial changes in mid-aged mice, we performed immunofluorescence microscopy, co-staining for phosphorylated p65-NFκB. HFD and WL increased pNFκB staining, expectedly in the nuclei, suggesting pro-inflammatory cellular activation. The total number of pNFκB-positive nuclei, many of which were non-microglial cells, was increased in the ARC in HFD compared to NC and was further higher in WL (Fig. 4A, B). This trend was also observed among microglia (Iba1 +/pNFκB + cells, Fig. 4C). Importantly, the volume of pNFκB-positive microglia was markedly greater than that of pNFκB-negative microglia under all three conditions (Fig. 4D, E). Jointly, these findings suggest that in mid-aged mice, the increased total microglial volume in the ARC following obesity and acute weight loss is a combination of increased microglial number and larger volume of pNFκB-positive microglial cells.

### WL in mid-aged mice normalizes dysglycemia while aggravating adipose tissue inflammation

**Fig. 5:**
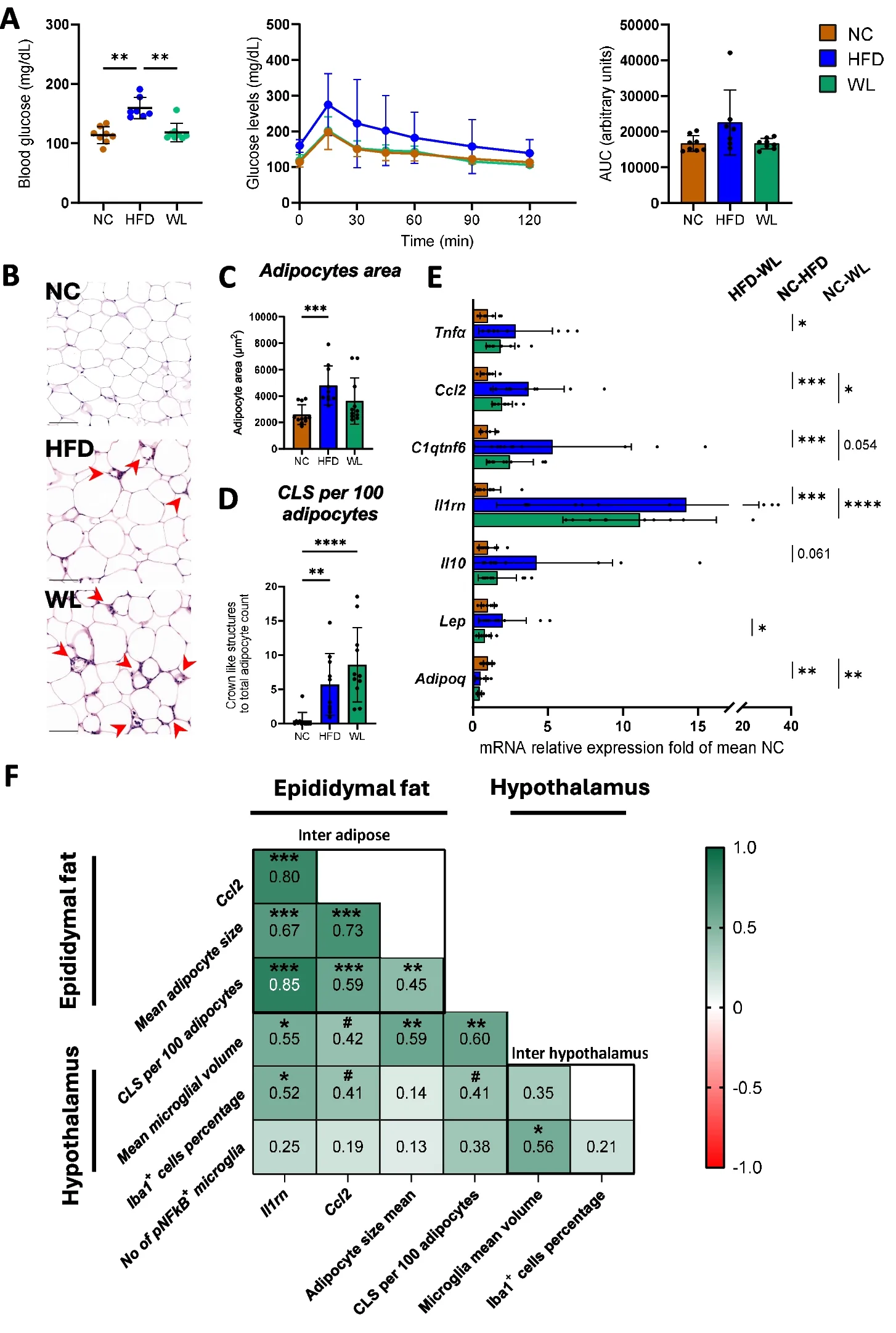
Effect of early weight loss on glycemia, adipose tissue inflammation, and its association with hypothalamic microglial changes in mid-aged mice. **A** From left to right: Fasting glucose levels, GTT curves, and AUCs for mid-aged mice (mean ± SD). **B** Representative images of mice epididymal adipose tissue stained for H&E. Crown-like structures (CLSs) marked with red arrows. Scale bar, 100 µm. **C** Left: adipocyte size (area) analysis (mean ± SD). **D** CLS count per 100 adipocytes (mean ± SD). **E** Mice epididymal adipose tissue mRNA expression analysis, fold change relative to NC group (mean ± SD). **F** Correlation matrix between epididymal adipose tissue and microscopic hypothalami parameters of the same mice. ^#^*p* < 0.075, *p < 0.05, **p < 0.01, ***p < 0.001, ****p < 0.0001

We next assessed the physiological correlates of the age-dependent, WL-induced aggravation of the localized basomedial (ARC) hypothalamic inflammation. Given the evidence for central sensing and regulation of peripheral glucose homeostasis and obesity-induced hypothalamic changes, we next assessed glycemic parameters and their relation to microglia activation in the ARC. Ten weeks of HFD consumption induced fasting hyperglycemia and significant glucose intolerance in both mouse age groups, with a lessened severity of dysglycemia seen in mid-aged mice (Fig. 5A) compared to young mice (Supplementary Fig. 3). Nevertheless, 1 week into the dietary switch, when only 42% of the excess body weight gained from 8 weeks of HFD was lost in the WL group (Fig. 1B), both fasting glycemia and glucose excursion after an intra-peritoneal glucose load were fully normalized (Fig. 5A and Supplementary Fig. 3). When looking at the adipose tissue, we previously demonstrated persistent adipose tissue inflammation in this WL model in young mice [16]. In mid-aged mice, peri-epidydimal fat expectedly demonstrated adipocyte hypertrophy and increased crown-like structure density induced by HFD (Fig. 5B–D). Weight loss trended to reverse adipocyte hypertrophy, but to increase CLS percentage (per 100 adipocytes, Fig. 5C, D). Adipose tissue expression of a panel of adipocytokines mostly showed non-significant trends of reversal (Fig. 5E, D). Intercorrelation analysis revealed strong associations within adipose tissue or hypothalamic inflammatory parameters. In the hypothalamus, this was particularly pronounced between microglial volume and percent pNFκB microglia, consistent with results in Fig. 4. Intriguingly, microglial cell volume, though not hypothalamic pNFκB-positive microglia percentage, significantly correlated (*p* ≤ 0.02) with several adipose tissue inflammatory parameters (Fig. 5F), suggesting coordinated adipose-hypothalamic inflammatory response to WL in mid-age.

## Discussion

Hypothalamic inflammation, specifically involving microglia, has been proposed to link obesity with dysmetabolism [3, 21]. In the present study, we entertained two intertwined hypotheses to better understand the physiology and dynamics of WL: (i) that reversal of obesity-induced hypothalamic changes is required for normalization of obesity-induced dysglycemia, and (ii) - that aging negatively affects the reversibility of changes induced by the same-duration obesity. Our findings disprove the first, and support the second hypothesis: (i) Dysglycemia is fully and rapidly restored upon dietary switch, in mid-age as in young mice, without parallel reversal of obesity-induced hypothalamic inflammation. (ii) WL in mid-age aggravates hypothalamic microglia inflammatory changes, which were induced by prior weight gain/obesity. Our results provide several novel insights into the complex associations between weight dynamics, dysglycemia, and hypothalamic and adipose tissue inflammation in obesity and its reversal and into how age affects microglial adaptation to obesity and early WL.

Central regulation of glucose homeostasis has been convincingly shown in rodent models since the 1990s [22]. Hypothalamic inflammation has been proposed to mediate metabolic dysregulation in obesity, but high glucose [23] and/or FFA [24] levels have also been proposed to induce hypothalamic microglial changes in obesity, potentially generating a “feed-forward” pathogenic feedback loop. Here, we investigated the associations between hypothalamic and microglial changes and dysglycemia during WL. Studies in both human and mouse have demonstrated that dysglycemia is rapidly corrected during early WL, clearly preceding the restoration of normal weight [16]. Microglial changes in response to obesity are also reversible, as shown in previous reports [20]. We also observed normalization of obesity-induced microglial changes in both mid-aged and young mice when extending the obesity reversal time (i.e., the switch to NC after HFD) from 2 to 5 weeks, when weight stabilized (see Supplemental Fig. 4). Since glycemia was fully normalized as early as 1 week following the dietary switch, it occurred independently of either reversal of whole hypothalamus obesity-induced transcriptomic changes or of the cytomorphological changes of hypothalamic microglia that are consistent with inflammatory activation, contrasting our initial hypothesis. Moreover, mid-aged mice exhibited further aggravation of the obesity-induced microglial changes in response to early WL. This corresponded to the transcriptomic changes in the hypothalamus, with most of the genes altered by obesity (up- or downregulated) exhibiting further aggravation of this effect early into the dietary switch. Interestingly, a recent study suggested that hypothalamic inflammation may be required to restore normoglycemia [25], and our results would suggest that this may be particularly operational in mid-age. It would be interesting to assess whether this represents an age-dependent difference in the function of microglia, as previously suggested [26], and whether this augmented response to early weight loss/dietary switch could explain the counter-intuitive, more severe impact of obesity and dysmetabolism in childhood and adolescence than in mid-age.

Microgliosis, a general term referring to the increased numbers (density) and morphological changes in microglia, has been documented in both humans and murine models of obesity by several groups [1–3]. Yet, some groups failed to clearly document obesity-related astrogliosis [27], or suggested that the development or robustness of such changes depends on the model (mouse/rat, diet and its duration, anatomical location in the brain) [28]. Nevertheless, activated glia cells is generally considered a manifestation and/or a cause of hypothalamic inflammation during obesity. In early WL, our results demonstrate the complexity of deciphering hypothalamic inflammation: Whole hypothalamus (bulk) RNA-seq demonstrated that this tissue exhibits much less pronounced transcriptomic alterations than adipose tissue does. Moreover, among hypothalamic DEGs, inflammatory processes are not evidently enriched. This may indicate that the inflammatory response is more subtle, regulated mainly post-transcriptionally, and/or occurs in well-defined spatial coordinates and/or specific cell types comprising the hypothalamus (microglia, astrocytes, neurons, etc.). Such effects may be masked when assessed in the whole hypothalamus and could be revealed using single-cell-based spatial transcriptomics and/or imaging. As for spatial considerations, indeed, MBH that includes specific areas of the hypothalamus, such as the median eminence, ARC, and PVN, seems to be more affected by obesity and its reversal compared to more lateral regions (and nuclei) within the hypothalamus. These regions exhibit a greater number of microglial cells, a greater volume which they occupy, and a greater percentage of pNFκB-positive nuclei and total pNFκB signal intensity, in response to obesity, and in mid-age, further in response to WL. Remarkably, under all conditions, the cell volume of pNFκB-positive microglia was larger than that of pNFκB-negative microglia, suggesting that larger microglial cell volume and NFκB activation are linked. These findings support the notion that microglial changes represent an inflammatory response, which likely also involves other cell types, as previously shown in obesity. Consistently, this may extend beyond the hypothalamus, as adipose tissue CLS correlated with hypothalamic microglial cell volume and pNFκB-positive cell percentage. The downstream effect of ARC microglial activation remains elusive, but as our experiment revealed a rapid metabolic improvement with further microglial activation, we suggest a possible beneficial effect of this acute augmentation of microglial activation in mid-aged mice.

Strengths/novelty of this study include the focus on WL in mid-aged mice, since our findings fill a significant gap of knowledge given that the majority of mouse weight gain and weight loss studies are performed in young mice. Moreover, human clinical obesity is more prevalent in mid-age, and healthcare professionals deal more with attempts to manage obesity, thereby facing the effects of WL. A major weakness is that the cause/mediator of the aggravation of obesity-induced microglial changes upon early WL in mid-aged mice remains to be defined. Both inflammatory (adipo)cytokines and/or free fatty acids, particularly saturated FA like palmitate, have been shown to activate microglia [3, 29, 30]. In the context of “acute” WL, accompanied by a reduction in adipocyte size and increased/maintained adipose tissue inflammation, enhanced adipose tissue lipolysis is likely to occur, elevating circulating free fatty acid levels. Decreased oxidative phosphorylation was one of the top pathways downregulated by obesity and further decreased by WL. If, as a result, the hypothalami of mid-aged mice had reduced capacity to catabolize fatty acids, aggravated microglial changes could reflect enhanced lipotoxicity and/or fatty acid–induced inflammation via a receptor such as TLR4 [29]. Future studies are needed to assess if a more gradual induction of WL could prevent the aggravated microglial response to WL in mid-age.

In conclusion, during early WL (weight-losing phase of obesity reversal) in mid-aged mice, rapid glycemic normalization occurs independently of reversal of hypothalamic microglial inflammatory changes. Moreover, obesity-induced microglial changes are further aggravated during early weight loss. This aggravation of acute WL response mirrors the whole hypothalamus transcriptome, although not clearly engaging the whole-hypothalamus inflammatory pathway activation. Identifying both the upstream signals for hypothalamic microgliosis (possibly metabolic-inflammatory mediators from adipose tissue) and its downstream consequences during WL could uncover possible ways to modulate the metabolic benefits and possibly the ability to sustain weight loss in individuals living with obesity, considering age-dependent effects.

## Supplementary Information

Below is the link to the electronic supplementary material.

ESM 1 Supplemental Fig. 1: KEGG pathway enrichment analysis of adipose tissue DEGs included in the aggravated up-regulated pattern. 1955 adipose tissue DEGs included in the aggravated up-regulated pattern (up-up, see Fig. 1C) were subjected to KEGG pathway enrichment analysis using Enrichr (https://maayanlab.cloud/Enrichr/). X-axis represents the number of DEGs involved in the pathway, circle diameter represents the percentage of DEGs out of the total genes in the pathway, and color corresponds to the adjusted p-value of the enrichment. Shown in the graph are selected pathways among the top enriched pathways sorted by FDR-adjusted p-value for each pathway. (PDF 140 KB)

ESM 2 Supplemental Fig. 2: Imaris software confocal images-based surfaces analysis. Scheme of Imaris confocal image-to-surface analysis transformation. Scale bar, 100 µm. (PDF 141 KB)

ESM 3 Supplemental Fig. 3: Young mice I.P GTT results. From left to right: Fasting glucose levels, GTT curves and AUCs for young (left) and mid-aged (right) mice (mean ± SD). GTT conditions were identical to those of mid-aged mice, and are detailed in Methods. *- p < 0.05, **—p < 0.01, ***—p < 0.001, ****-p < 0.0001. (PDF 216 KB)

ESM 4 Supplemental Fig. 4: Young and mid-aged mice 5w weight loss.** A.** Young and mid-aged mice were randomly assigned to NC or a 60% HFD, and weighed weekly. Eight weeks after dietary initiation, HFD fed mice were switched back to NC diet for an additional 5 weeks of obesity reversal (‘Weight loss’ group, 5w WL), totaling 13 weeks of dietary intervention. **B**. Weekly body weight measurements in grams (mean). **C, F.** Mid-aged (**C.**) and young (**F.**) percentage of Iba1 + cells among the total identified nuclei in the ARC (mean ± SD). **D, G.** Total Iba1 volume in mid-aged (**D.**) and young (**G.**) mice (mean ± SD). **E, H.** Average volume of Iba1-positive cells in the ARC of mid-aged (**E.**) and young (**H.**) mice (mean ± SD). (PDF 435 KB)

ESM 5 (PDF 96.7 KB)

ESM 6 (PDF 1.64 MB)

ESM 7 (PDF 5.44 MB)

ESM 8 (PDF 60.4 KB)
