# Supplementary material for: Weight loss aggravates obesity-induced hypothalamic inflammation in mid-aged mice
Source: GeroScience. 2025 Oct 17;48(4):5329–42. doi: 10.1007/s11357-025-01933-x (PMC13575038; doi:10.1007/s11357-025-01933-x)
Supplement: Supplementary file 1 [file 11357_2025_1933_MOESM1_ESM.pdf]

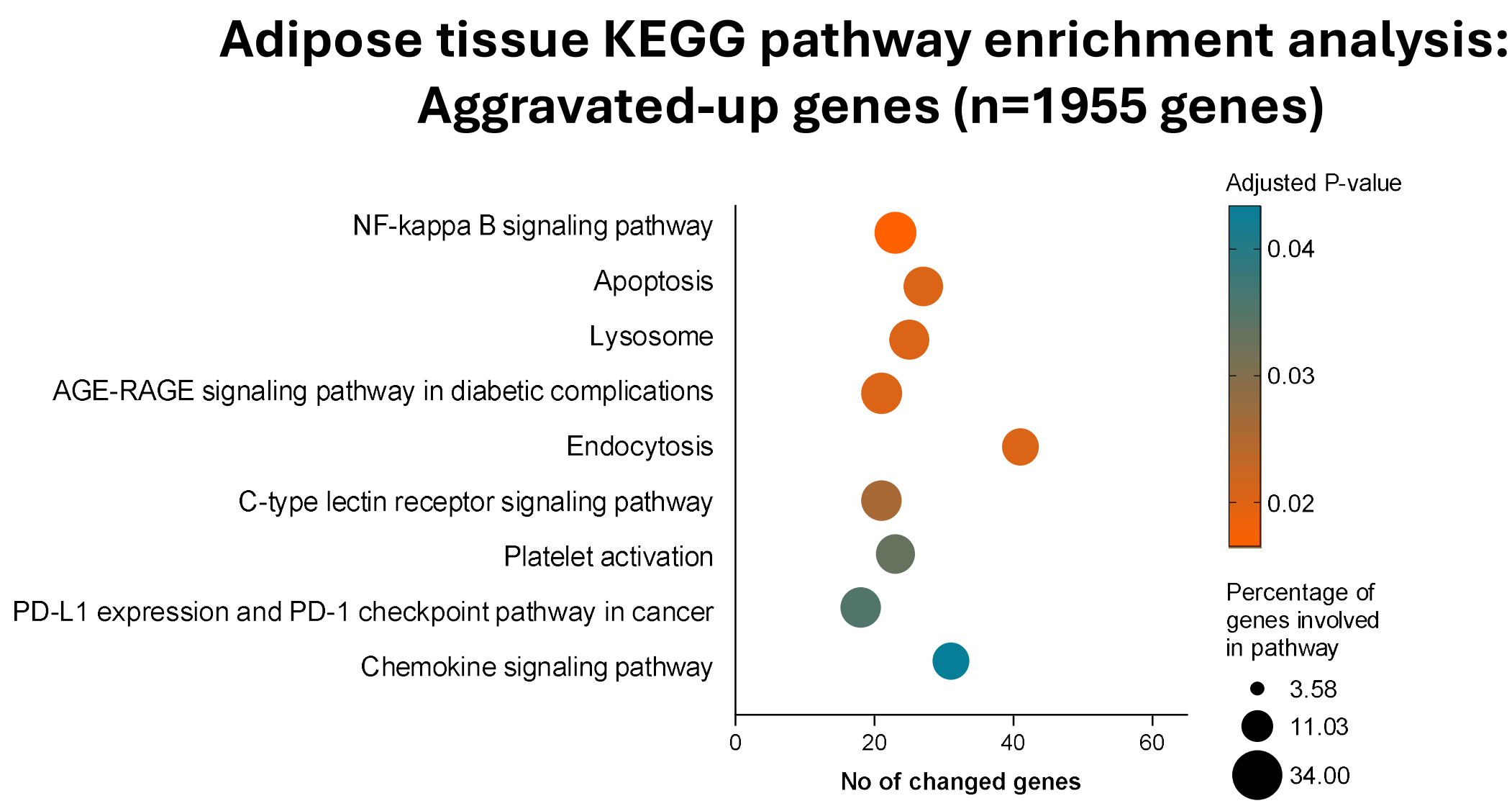

Supplementary Figure 1
