# Supplementary material for: Weight loss aggravates obesity-induced hypothalamic inflammation in mid-aged mice
Source: GeroScience. 2025 Oct 17;48(4):5329–42. doi: 10.1007/s11357-025-01933-x (PMC13575038; doi:10.1007/s11357-025-01933-x)
Supplement: Supplementary file 2 [file 11357_2025_1933_MOESM2_ESM.pdf]

Imaris analysis- ARC Iba1 surfaces acquisition diagram

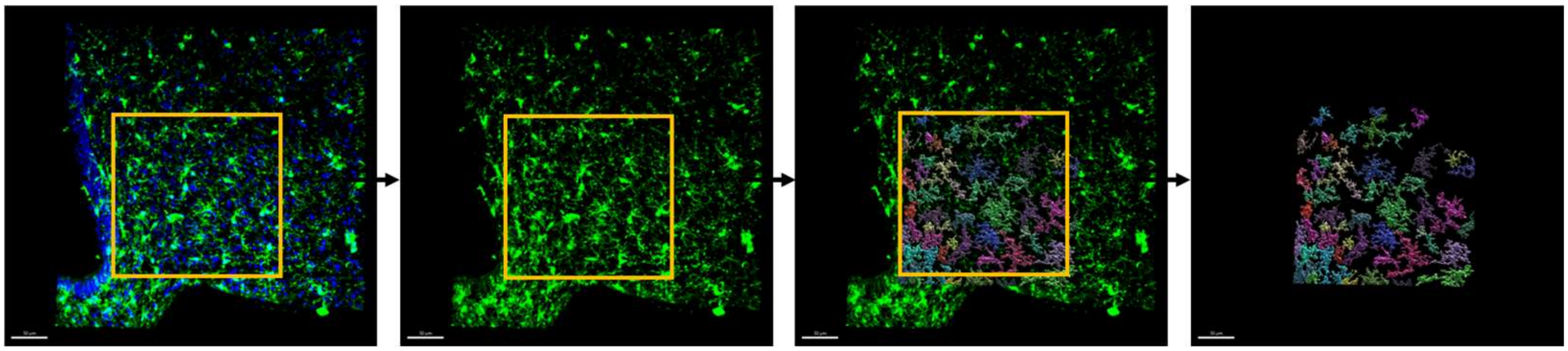

Supplementary Figure 2
