# Supplementary material for: Weight loss aggravates obesity-induced hypothalamic inflammation in mid-aged mice
Source: GeroScience. 2025 Oct 17;48(4):5329–42. doi: 10.1007/s11357-025-01933-x (PMC13575038; doi:10.1007/s11357-025-01933-x)
Supplement: Supplementary file 5 [file 11357_2025_1933_MOESM5_ESM.pdf]

Supplemental Table S1: Used Antibodies

Antibody target/ Fluorophore	Dilution	Supplier	Catalog no.	Species	Application
Iba-1	1:500	Abcam, United Kingdom	ab5076	Goat	Primary antibody
pNFkB	1:500	Abcam, United Kingdom	ab86299	Rabbit	Primary antibody
Alexa Fluor 488	1:500-250	Abcam, United Kingdom	ab150073	Donkey Anti Rabbit	Secondary antibody
Alexa Fluor 647	1:250	Jackson ImmunoResearch Laboratories	705-605-147	Donkey Anti Goat	Secondary antibody
DAPI	1:2500	Biolegend, CA, USA	422801	-	Nuclear staining
