# Supplementary material for: Weight loss aggravates obesity-induced hypothalamic inflammation in mid-aged mice
Source: GeroScience. 2025 Oct 17;48(4):5329–42. doi: 10.1007/s11357-025-01933-x (PMC13575038; doi:10.1007/s11357-025-01933-x)
Supplement: Supplementary file 6 [file 11357_2025_1933_MOESM6_ESM.pdf]

Supplemental table S2

Gene information | Normalized counts per sample

Ensembl_Gene_ID	Gene_Symbol	Gene_Title	Gene_Type	Young_01 (Y_NC)	Young_02 (Y_NC)	Young_03 (Y_NC)	Young_05 (Y_NC)	Young_06 (Y_HFD)	Young_07 (Y_HFD)	Young_08 (Y_HFD)	Young_09 (Y_HFD)	Young_11 (Y_Reverse)	Young_12 (Y_Reverse)	Young_13 (Y_Reverse)	Young_14 (Y_Reverse)	Young_15 (Y_Reverse)
ENSMUSG00000020889	Nr1d1	nuclear receptor subfar	protein_coding	1,801	1,605	1,856	1,902	2,232	2,419	2,040	1,904	2,052	2,164	2,154	1,986	2,227
ENSMUSG00000019027	Dnah1	dynein, axonemal, heav	protein_coding	1,395	1,404	1,354	1,433	1,952	1,538	1,501	1,641	1,561	1,634	1,570	1,735	1,642
ENSMUSG00000028137	Celf3	CUGBP, Elav-like family	protein_coding	3,144	3,166	2,838	2,832	3,850	3,542	3,369	2,965	3,273	3,512	3,364	3,562	3,319
ENSMUSG00000021432	Slc35b3	solute carrier family 35,	protein_coding	207	265	266	212	327	304	311	323	278	330	333	298	382
ENSMUSG00000029538	Srsf9	serine and arginine-rich	protein_coding	612	623	666	616	744	687	656	731	685	736	677	715	750
ENSMUSG00000056972	Magel2	MAGE family member L	protein_coding	1,732	2,142	1,921	1,736	2,543	2,146	2,001	2,329	2,047	2,376	2,099	2,343	2,320
ENSMUSG00000038141	Tmem181a	transmembrane proteir	protein_coding	1,584	1,704	1,776	1,781	1,836	1,881	1,885	1,890	1,828	1,838	1,844	1,966	1,884
ENSMUSG00000039199	Zdhhc1	zinc finger, DHHC doma	protein_coding	678	844	833	809	962	880	920	916	893	935	935	973	953
ENSMUSG00000044308	Ubr3	ubiquitin protein ligase	protein_coding	5,635	6,433	6,135	5,969	7,261	7,107	6,758	7,023	6,858	6,615	7,055	7,501	6,829
ENSMUSG00000048310	Pskh1	protein serine kinase H	protein_coding	240	253	259	251	291	323	303	302	304	290	322	310	314
ENSMUSG00000053754	Chd8	chromodomain helicase	protein_coding	3,666	4,191	3,746	3,957	4,569	4,617	4,908	3,958	5,077	4,585	4,525	4,069	4,677
ENSMUSG00000056342	Usp34	ubiquitin specific peptic	protein_coding	7,870	8,195	8,190	8,479	8,801	9,100	9,289	9,080	9,822	9,171	8,689	8,860	8,767
ENSMUSG00000029419	Ajm1	apical junction compon	protein_coding	1,727	1,826	1,884	1,657	2,029	1,867	1,986	2,114	1,955	2,130	2,025	2,050	1,942
ENSMUSG00000044700	Tmem201	transmembrane proteir	protein_coding	1,836	1,811	2,055	1,773	2,083	2,004	2,070	2,229	2,081	2,140	2,129	2,165	1,994
ENSMUSG00000035713	Usp35	ubiquitin specific peptic	protein_coding	499	592	699	552	820	684	770	757	795	823	800	761	702
ENSMUSG00000046020	Pofut1	protein O-fucosyltransf	protein_coding	775	903	1,036	798	1,065	1,059	1,051	1,027	1,078	1,013	1,126	1,082	1,011
ENSMUSG00000040327	Cul9	cullin 9	protein_coding	1,745	1,783	1,930	1,889	2,192	2,004	2,143	2,236	2,256	2,114	2,096	2,198	1,891
ENSMUSG00000020802	Ube2o	ubiquitin-conjugating e	protein_coding	4,096	4,128	4,382	4,309	4,545	4,321	4,465	4,756	4,798	4,462	4,560	4,526	4,418
ENSMUSG00000037224	Zfyve28	zinc finger, FYVE domai	protein_coding	406	417	435	414	473	436	487	534	538	485	513	465	459
ENSMUSG00000018401	Mtmr4	myotubularin related p	protein_coding	2,401	2,459	2,481	2,531	2,783	2,628	2,695	2,863	2,726	2,939	2,667	2,681	2,654
ENSMUSG00000027006	Dnajc10	DnaJ heat shock proteir	protein_coding	2,549	2,514	2,615	2,582	2,798	2,796	2,838	3,192	2,878	2,940	2,984	2,878	2,784
ENSMUSG00000006342	Susd2	sushi domain containin	protein_coding	1,031	1,104	1,055	1,111	1,401	1,191	1,203	1,235	1,186	1,236	1,210	1,146	1,222
ENSMUSG00000021091	Serpina3n	serine (or cysteine) pep	protein_coding	1,310	1,266	870	1,188	2,572	1,726	1,700	2,009	1,288	1,692	1,386	1,468	1,462
ENSMUSG00000051790	Nlgn2	neuroligin 2	protein_coding	8,719	8,253	8,040	8,227	9,370	8,813	9,116	9,523	8,521	8,715	8,637	9,770	8,950
ENSMUSG00000026204	Ptprn	protein tyrosine phosph	protein_coding	13,125	13,921	13,475	13,578	15,894	14,426	14,740	15,964	14,723	15,403	14,567	15,643	14,240
ENSMUSG00000045039	Megf8	multiple EGF-like-doma	protein_coding	6,429	6,905	7,481	6,766	7,817	7,275	7,833	8,177	7,776	7,493	7,643	7,915	7,319
ENSMUSG00000022761	Lztr1	leucine-zipper-like tran	protein_coding	2,201	1,980	2,238	2,012	2,505	2,443	2,171	2,511	2,313	2,391	2,364	2,298	2,325
ENSMUSG00000037727	Avp	arginine vasopressin	protein_coding	3,032	2,205	2,717	2,642	5,172	3,527	4,149	6,136	3,164	4,209	3,784	4,295	4,205
ENSMUSG00000040859	Bsdc1	BSD domain containing	protein_coding	1,456	1,448	1,461	1,445	1,686	1,534	1,635	1,757	1,641	1,587	1,629	1,545	1,634
ENSMUSG00000004996	Mri1	methylthioribose-1-pho	protein_coding	237	277	282	249	283	306	320	312	317	303	300	353	336
ENSMUSG00000051747	Ttn	titin	protein_coding	56	87	200	116	311	287	492	125	180	362	317	575	399
ENSMUSG00000001870	Ltbp1	latent transforming gro	protein_coding	187	265	311	184	330	377	282	227	396	348	351	310	315
ENSMUSG00000023826	Prkn	parkin RBR E3 ubiquitin	protein_coding	189	280	330	161	359	344	319	267	376	429	412	370	357
ENSMUSG00000029168	Dpysl5	dihydropyrimidinase-lik	protein_coding	1,984	2,492	2,537	2,096	2,696	2,436	2,498	2,372	2,683	2,805	2,721	2,602	2,611
ENSMUSG00000045482	Trrap	transformation/transcri	protein_coding	4,750	5,791	5,745	4,915	6,329	5,946	6,055	5,421	6,238	6,097	6,279	6,034	5,998
ENSMUSG00000053693	Mast1	microtubule associated	protein_coding	3,260	3,744	3,628	3,567	3,848	3,839	3,926	3,667	4,017	4,142	4,009	3,850	3,649
ENSMUSG00000033767	Tmem131l	transmembrane 131 lik	protein_coding	255	442	384	337	455	512	501	343	501	486	539	453	450
ENSMUSG00000117113	Gm49883	predicted gene, 49883	TEC	156	246	263	212	336	317	294	219	308	316	334	309	282
ENSMUSG00000107877	4933427D14Rik	RIKEN cDNA 4933427D1	protein_coding	568	936	942	755	1,055	1,049	1,017	810	1,229	1,169	939	1,199	1,125
ENSMUSG00000028041	Adam15	a disintegrin and metall	protein_coding	1,550	1,821	1,929	1,764	1,989	1,960	2,035	1,865	2,169	1,963	2,142	2,119	2,102
ENSMUSG00000047909	Ankrd16	ankyrin repeat domain	protein_coding	477	644	765	629	689	715	720	698	826	784	800	859	838
ENSMUSG00000097767	Miat	myocardial infarction as	lncRNA	5,868	9,034	10,281	7,818	10,065	9,628	10,129	9,915	11,774	10,981	11,640	11,187	11,280
ENSMUSG00000113852	Gm48062	predicted gene, 48062	TEC	261	437	484	352	521	499	463	450	580	548	560	523	549
ENSMUSG00000034160	Ogt	O-linked N-acetylglucos	protein_coding	6,949	8,815	9,536	8,112	9,294	9,500	9,317	8,852	9,865	10,086	10,457	9,501	10,105
ENSMUSG00000055670	Zzef1	zinc finger, ZZ-type wit	protein_coding	1,901	2,629	2,678	2,410	2,642	2,709	2,793	2,644	2,982	2,965	3,106	2,863	2,913
ENSMUSG00000039801	Cplane1	ciliogenesis and planar	protein_coding	2,680	3,566	3,501	3,048	3,439	3,653	3,628	3,508	3,950	3,880	3,721	3,862	3,774
ENSMUSG00000107182	Gm43268	predicted gene 43268	TEC	216	342	355	297	354	400	353	321	396	388	416	416	408

DESeq2 results for differential expression | Expression pattern

Ensembl_Gene_ID	linearFC.Y_HFD.vs.NC	pvalue.Y_HFD.vs.NC	padj.Y_HFD.vs.NC	pass.Y_HFD.vs.NC	linearFC.Y_Reverse.vs.NC	pvalue.Y_Reverse.vs.NC	padj.Y_Reverse.vs.NC	pass.Y_Reverse.vs.NC	linearFC.Y_Reverse.vs.HFD	pvalue.Y_Reverse.vs.HFD	padj.Y_Reverse.vs.HFD	pass.Y_Reverse.vs.HFD	Pattern_No.	Clustering_order
ENSMUSG00000020889	1.2	0.00142	0.122		1.18	0.0021	0.0431	up	-1.02	0.779	1		1	1
ENSMUSG00000019027	1.19	0.00126	0.121		1.17	0.0024	0.0461	up	-1.02	0.721	1		1	2
ENSMUSG00000028137	1.15	0.00226	0.141		1.14	0.00235	0.0459	up	-1.01	0.864	1		1	3
ENSMUSG00000021432	1.32	0.00079			1.36	0.000131	0.0103	up	1.02	0.75	1		1	4
ENSMUSG00000029538	1.12	0.00579			1.13	0.00182	0.04	up	1.01	0.801	1		1	5
ENSMUSG00000056972	1.2	0.00245	0.146		1.19	0.00237	0.046	up	-1.01	0.883	1		1	6
ENSMUSG00000038141	1.09	0.00121	0.121		1.09	0.000783	0.0262	up	-1	0.986	1		1	7
ENSMUSG00000039199	1.16	0.0026	0.151		1.18	0.000337	0.0174	up	1.02	0.66	1		1	8
ENSMUSG00000044308	1.16	0.000153	0.0477	up	1.15	0.000184	0.0125	up	-1.01	0.806	1		1	9
ENSMUSG00000048310	1.21	0.00081			1.23	0.00024	0.0139	up	1.01	0.837	1		1	10
ENSMUSG00000053754	1.16	0.00121	0.121		1.18	0.000155	0.011	up	1.02	0.705	1		1	11
ENSMUSG00000056342	1.11	0.000129	0.0477	up	1.11	0.0000673	0.00711	up	-1	0.969	1		1	12
ENSMUSG00000029419	1.13	0.00136	0.121		1.14	0.000257	0.0145	up	1.01	0.761	1		1	13
ENSMUSG00000044700	1.12	0.000906	0.11		1.12	0.000391	0.0191	up	1	0.944	1		1	14
ENSMUSG00000035713	1.29	0.00011			1.32	0.00000879	0.00173	up	1.02	0.692	1		1	15
ENSMUSG00000046020	1.19	0.0027	0.151		1.21	0.000791	0.0262	up	1.01	0.834	1		1	16
ENSMUSG00000040327	1.17	0.00145	0.123		1.15	0.00267	0.0486	up	-1.02	0.728	1		1	17
ENSMUSG00000020802	1.07	0.00464	0.182		1.08	0.00112	0.0311	up	1.01	0.769	1		1	18
ENSMUSG00000037224	1.15	0.00736			1.17	0.00154	0.0367	up	1.02	0.699	1		1	19
ENSMUSG00000018401	1.11	0.000108	0.0477	up	1.11	0.0000944	0.00878	up	-1	0.885	1		1	20
ENSMUSG00000027006	1.13	0.00131	0.121		1.13	0.00118	0.0318	up	-1	0.895	1		1	21
ENSMUSG00000006342	1.17	0.000166	0.0477	up	1.11	0.00591	0.0709		-1.05	0.228	1		2	22
ENSMUSG00000021091	1.73	0.00000876	0.0343	up	1.26	0.0481	0.21		-1.37	0.0067	1		2	23
ENSMUSG00000051790	1.11	0.00012	0.0477	up	1.07	0.00508	0.0654		-1.03	0.212	1		2	24
ENSMUSG00000026204	1.13	0.0000977	0.0477	up	1.1	0.00087	0.0273	up	-1.02	0.439	1		2	25
ENSMUSG00000045039	1.13	0.0000327	0.0343	up	1.11	0.000239	0.0139	up	-1.02	0.485	1		2	26
ENSMUSG00000022761	1.14	0.000223	0.0519		1.11	0.00259	0.0479	up	-1.03	0.384	1		2	27
ENSMUSG00000037727	1.79	0.0000416	0.0343	up	1.48	0.00346	0.0549		-1.21	0.163	1		2	28
ENSMUSG00000040859	1.14	0.000147	0.0477	up	1.11	0.00193	0.0411	up	-1.03	0.38	1		2	29
ENSMUSG00000004996	1.16	0.0265			1.22	0.00176	0.0394	up	1.05	0.405	1		3	30
ENSMUSG00000051747	2.65	0.00626			3.2	0.000587	0.0229	up	1.21	0.576	1		3	31
ENSMUSG00000001870	1.28	0.0508			1.45	0.00202	0.0419	up	1.13	0.296	1		3	32
ENSMUSG00000023826	1.34	0.00875			1.61	0.00000524	0.00128	up	1.21	0.0686	1		3	33
ENSMUSG00000029168	1.1	0.0673	0.39		1.18	0.000639	0.0241	up	1.07	0.134	1		3	34
ENSMUSG00000045482	1.12	0.024	0.292		1.16	0.00228	0.0452	up	1.03	0.5	1		3	35
ENSMUSG00000053693	1.07	0.0244	0.293		1.11	0.000841	0.0273	up	1.03	0.326	1		3	36
ENSMUSG00000033767	1.27	0.0159			1.37	0.000953	0.0289	up	1.08	0.438	1		3	37
ENSMUSG00000117113	1.32	0.00986			1.4	0.000913	0.0282	up	1.06	0.537	1		3	38
ENSMUSG00000107877	1.23	0.0935	0.446		1.41	0.00274	0.049	up	1.15	0.218	1		3	39
ENSMUSG00000028041	1.11	0.0154	0.273		1.19	0.0000216	0.00343	up	1.07	0.086	1		3	40
ENSMUSG00000047909	1.12	0.156			1.3	0.000373	0.0187	up	1.16	0.0365	1		3	41
ENSMUSG00000097767	1.2	0.0572	0.371		1.38	0.00053	0.0218	up	1.14	0.144	1		3	42
ENSMUSG00000113852	1.25	0.0505			1.43	0.00108	0.0306	up	1.14	0.222	1		3	43
ENSMUSG00000034160	1.11	0.105	0.456		1.2	0.00226	0.0449	up	1.08	0.178	1		3	44
ENSMUSG00000055670	1.12	0.0411	0.33		1.23	0.0000752	0.00767	up	1.1	0.0692	1		3	45
ENSMUSG00000039801	1.11	0.096	0.449		1.2	0.00255	0.0477	up	1.08	0.205	1		3	46
ENSMUSG00000107182	1.17	0.109			1.33	0.00253	0.0477	up	1.13	0.177	1		3	47

z-score expression values for heatmap visualization

Ensembl_Gene_ID	Young_01.zscore (Y_NC)	Young_02.zscore (Y_NC)	Young_03.zscore (Y_NC)	Young_05.zscore (Y_NC)	Young_06.zscore (Y_HFD)	Young_07.zscore (Y_HFD)	Young_08.zscore (Y_HFD)	Young_09.zscore (Y_HFD)	Young_11.zscore (Y_Reverse)	Young_12.zscore (Y_Reverse)	Young_13.zscore (Y_Reverse)	Young_14.zscore (Y_Reverse)	Young_15.zscore (Y_Reverse)
ENSMUSG00000020889	-1.0364631	-2.08150336	-0.7624696	-0.53644662	0.93998596	1.69164682	0.10619869	-0.52727323	0.16275774	0.65229223	0.60933483	-0.13838658	0.92032621
ENSMUSG00000019027	-1.10138655	-1.03399338	-1.38803263	-0.83297033	2.25102844	-0.13961618	-0.37852281	0.50656893	0.01084451	0.46274609	0.06785662	1.06241798	0.51305931
ENSMUSG00000028137	-0.44946635	-0.37068504	-1.56246307	-1.58485616	1.77676163	0.85725131	0.30895022	-1.08828526	-0.00747239	0.76443235	0.29244121	0.91938164	0.14400991
ENSMUSG00000021432	-1.88019263	-0.55341195	-0.54082514	-1.76865891	0.66529944	0.22892737	0.35526573	0.57824334	-0.28702012	0.72212796	0.77330782	0.10390766	1.60302944
ENSMUSG00000029538	-1.51577863	-1.27010654	-0.35136892	-1.43789857	1.19612791	0.08424031	-0.56275954	0.94989632	0.03075817	1.04954079	-0.12250206	0.6410357	1.30881507
ENSMUSG00000056972	-1.69682638	0.08391192	-0.83587919	-1.67880532	1.54636073	0.09797124	-0.49122068	0.79554962	-0.30207019	0.96321526	-0.0892309	0.84459633	0.76242753
ENSMUSG00000038141	-2.53492957	-1.2088164	-0.45453664	-0.40036977	0.14993242	0.60115	0.63660582	0.68887372	0.07735457	0.17261794	0.2283996	1.41299927	0.63071903
ENSMUSG00000039199	-2.67701217	-0.48010648	-0.61670073	-0.90506426	0.88364084	-0.04172279	0.41627137	0.36854102	0.10030746	0.58114172	0.57980025	1.00579781	0.78510597
ENSMUSG00000044308	-2.07284767	-0.46955272	-1.04329283	-1.37682777	1.00357636	0.74280827	0.13052596	0.59705821	0.30803225	-0.13012034	0.65348082	1.40055958	0.2565999
ENSMUSG00000048310	-1.75475178	-1.27004376	-1.03748782	-1.33605026	0.08003423	1.13332927	0.48811281	0.45890149	0.51649575	0.05911561	1.09972922	0.72568254	0.83693269
ENSMUSG00000053754	-1.59681635	-0.31211002	-1.39181145	-0.86677162	0.52077259	0.62100737	1.2139886	-0.8639738	1.54380814	0.55561827	0.42660359	-0.59672703	0.7464117
ENSMUSG00000056342	-1.82244185	-1.14903482	-1.15956857	-0.58133475	0.0411161	0.59974193	0.94301767	0.56369536	1.87675367	0.72920017	-0.17166308	0.15339729	-0.02287911
ENSMUSG00000029419	-1.49889359	-0.76019368	-0.34284784	-2.03972189	0.64691643	-0.46450541	0.35531065	1.19860134	0.14782823	1.29506405	0.61664891	0.78571444	0.06007836
ENSMUSG00000044700	-1.36002399	-1.55108914	0.21056413	-1.84272331	0.40354113	-0.14023906	0.31615694	1.35253204	0.38675638	0.78195814	0.70736009	0.94250265	-0.207296
ENSMUSG00000035713	-2.12415507	-1.09868159	-0.05752088	-1.52310055	0.97181612	-0.19700488	0.55854361	0.45304329	0.76491161	0.99041439	0.81008621	0.48371966	-0.03207193
ENSMUSG00000046020	-2.13558378	-0.84984295	0.33716765	-1.89140619	0.57273182	0.52382232	0.46021407	0.25622773	0.68375077	0.14253708	1.06479545	0.71390532	0.12168072
ENSMUSG00000040327	-1.73038066	-1.48616208	-0.58107667	-0.82700897	0.88481869	-0.14958618	0.62446073	1.11489293	1.21752992	0.46632559	0.366445	0.9153761	-0.81563441
ENSMUSG00000020802	-1.72316506	-1.5565139	-0.276864	-0.63831286	0.50304963	-0.57931128	0.12379471	1.4823148	1.67170229	0.10714424	0.57716667	0.41497601	-0.10598125
ENSMUSG00000037224	-1.40260516	-1.141187	-0.68767723	-1.21921305	0.19108582	-0.66443675	0.49016908	1.48406938	1.58236772	0.45725495	1.05745956	-0.00939518	-0.13789214
ENSMUSG00000018401	-1.6615579	-1.26015815	-1.11250523	-0.77789097	0.8234196	-0.14338453	0.28159157	1.3003302	0.47608805	1.75009909	0.10655845	0.19593535	0.02147445
ENSMUSG00000027006	-1.31251936	-1.50846146	-0.94291142	-1.12164294	0.04264652	0.02949902	0.24498057	1.96368064	0.44960397	0.76097022	0.97627474	0.44798229	-0.03010277
ENSMUSG00000006342	-1.64489334	-0.79328542	-1.35736106	-0.71934057	2.22525418	0.15206736	0.27853193	0.61959162	0.10492152	0.62284309	0.35710555	-0.32915202	0.48371717
ENSMUSG00000021091	-0.47618286	-0.60462104	-1.94733547	-0.83639801	2.10816119	0.55900348	0.50121072	1.14165682	-0.53933431	0.48394923	-0.26829733	-0.05403964	-0.06777278
ENSMUSG00000051790	-0.17007756	-1.10775455	-1.55522596	-1.16241932	1.06283964	0.01279307	0.59291231	1.33969955	-0.56211265	-0.176879	-0.33084984	1.77866367	0.27841062
ENSMUSG00000026204	-1.62889679	-0.70820507	-1.2165036	-1.09782838	1.36751584	-0.15077669	0.18731467	1.43631732	0.16876939	0.87555652	0.00170977	1.11783208	-0.35280507
ENSMUSG00000045039	-2.097069	-1.06628823	0.09383742	-1.35907554	0.73049024	-0.31130048	0.76055263	1.38391131	0.65475035	0.11758056	0.40406999	0.91102626	-0.22248551
ENSMUSG00000022761	-0.49244224	-1.90655776	-0.26831374	-1.69053589	1.25086767	0.91231824	-0.67420795	1.28043971	0.17092725	0.62184569	0.46805464	0.08345317	0.24415121
ENSMUSG00000037727	-0.65266178	-1.74563853	-1.03298444	-1.12846112	1.22786824	-0.12567266	0.44533048	1.83929656	-0.5053189	0.49628285	0.12149346	0.56774191	0.49272393
ENSMUSG00000040859	-1.17875955	-1.26749307	-1.12255333	-1.30027741	1.10478541	-0.36860698	0.62502501	1.75023036	0.67707499	0.16170534	0.56477581	-0.25683891	0.61093232
ENSMUSG00000004996	-1.95519875	-0.60673288	-0.47261305	-1.55546611	-0.41565895	0.26972327	0.68907852	0.43916789	0.60552884	0.17188112	0.09818574	1.60581099	1.12629339
ENSMUSG00000051747	-1.70414388	-1.28053002	-0.25632354	-0.96196799	0.42630476	0.29840289	1.23427033	-0.87651782	-0.40224462	0.68479062	0.45654834	1.52821931	0.85319163
ENSMUSG00000001870	-1.74741067	-0.42111778	0.25016925	-1.79971382	0.49929545	1.08128314	-0.16607638	-1.02683096	1.30474645	0.72713197	0.77144019	0.23101144	0.2960717
ENSMUSG00000023826	-1.7144254	-0.41556768	0.16864306	-2.19233559	0.48389142	0.31925053	0.04446513	-0.58078049	0.65515903	1.16928248	1.00834845	0.59473263	0.45933642
ENSMUSG00000029168	-2.24426238	0.00034885	0.17949198	-1.70964017	0.78535494	-0.22639768	0.02391519	-0.49141497	0.7371552	1.17958125	0.87528871	0.42804358	0.46253551
ENSMUSG00000045482	-2.18790539	-0.00796114	-0.09469443	-1.81226239	0.97466169	0.28433999	0.48590465	-0.73484203	0.81519955	0.56112272	0.88747303	0.44784358	0.38112017
ENSMUSG00000053693	-2.33302297	-0.12743453	-0.62992804	-0.90023458	0.30923885	0.27253302	0.63004804	-0.46090077	0.99858026	1.4915164	0.96897027	0.32013783	-0.53950377
ENSMUSG00000033767	-2.34934049	0.1405811	-0.52771062	-1.13099415	0.28833645	0.87564959	0.76400251	-1.05806155	0.76007897	0.60688445	1.13075853	0.26670821	0.23310698
ENSMUSG00000117113	-2.38241682	-0.47740083	-0.15853537	-1.12297893	1.01011625	0.72500855	0.35776133	-0.98082867	0.58067794	0.70117635	0.97650588	0.60273558	0.16817873
ENSMUSG00000107877	-2.41125455	-0.15838895	-0.12802177	-1.15464111	0.4108885	0.38405461	0.23359537	-0.83534514	1.15079813	0.90522148	-0.14599997	1.0290476	0.72004581
ENSMUSG00000028041	-2.43077572	-0.72260906	-0.10267424	-1.06284127	0.2266374	0.06746269	0.47236995	-0.46270342	1.16429244	0.08346424	1.02768405	0.91403352	0.82565942
ENSMUSG00000047909	-2.56059942	-0.7134205	0.39206286	-0.86506522	-0.29109281	-0.05002985	0.00078839	-0.19932464	0.90034782	0.55479423	0.68393395	1.15457763	0.99302756
ENSMUSG00000097767	-2.68657028	-0.43871765	0.24001123	-1.19495241	0.12821886	-0.1044694	0.16195384	0.04956249	0.95338352	0.58661034	0.8930337	0.68396113	0.7279746
ENSMUSG00000113852	-2.59679408	-0.36377125	0.1220054	-1.33849081	0.47498844	0.26435559	-0.09498775	-0.22588278	0.99820297	0.71460298	0.82685845	0.48964684	0.729266
ENSMUSG00000034160	-2.60798751	-0.41070008	0.31823725	-1.18055394	0.0796785	0.28297844	0.10260614	-0.37197607	0.63330043	0.8388005	1.1749169	0.28421926	0.8564802
ENSMUSG00000055670	-2.7825874	-0.1974767	-0.046284	-0.89769001	-0.15550284	0.04698291	0.29303691	-0.15133992	0.82549135	0.77784418	1.15763577	0.49376107	0.63612869
ENSMUSG00000039801	-2.58953885	0.07381009	-0.09849959	-1.39412672	-0.26764287	0.30242931	0.23657451	-0.07888972	1.03877846	0.86923218	0.47552396	0.82503541	0.60731385
ENSMUSG00000107182	-2.65668605	-0.22236568	0.0010246	-1.00789197	-0.01622498	0.69628634	-0.03857917	-0.57741026	0.64187614	0.51097372	0.92143209	0.93554111	0.81202413
