# Supplementary material for: Weight loss aggravates obesity-induced hypothalamic inflammation in mid-aged mice
Source: GeroScience. 2025 Oct 17;48(4):5329–42. doi: 10.1007/s11357-025-01933-x (PMC13575038; doi:10.1007/s11357-025-01933-x)
Supplement: Supplementary file 7 [file 11357_2025_1933_MOESM7_ESM.pdf]

Supplemental table S3

Gene information | Normalized counts per sample | DESeq2 results for differential expression | Expression pattern | z-score expression values for heatmap visualization

Ensembl_Gene_ID	Gene_Symbol	Gene_Title	Gene_Type	Midaged_01	Midaged_02	Midaged_03	Midaged_04	Midaged_05	Midaged_06	Midaged_07	Midaged_08	Midaged_09	Midaged_10	Midaged_11	Midaged_12	linearFC_M_HFD	M_HFD.pvalue
ENSMUSG00000107352	Gm43660	predicted gene	lncRNA	153	208	171	225	270	272	215	269	300	297	220	251	1.36	0.00255
ENSMUSG00000095123	Gm21781	predicted gene,	lncRNA	301	409	328	390	465	493	463	499	520	472	440	497	1.35	0.0000177
ENSMUSG00000085635	Gm14565	predicted gene	lncRNA	31	59	36	49	69	77	53	75	74	75	65	61	1.57	0.00233
ENSMUSG00000086430	4930551O13Rik	RIKEN cDNA 49	lncRNA	3	11	5	7	16	14	12	15	20	16	13	13	2.29	0.00953
ENSMUSG00000060671	Atp8b2	ATPase, class I,	protein_coding	1,993	2,170	1,849	2,418	2,318	2,665	2,436	2,618	2,670	2,617	2,348	2,473	1.19	0.00446
ENSMUSG00000058706	0610030E20Rik	RIKEN cDNA 06	protein_coding	479	506	449	561	595	650	587	618	599	665	593	570	1.23	0.000209
ENSMUSG00000115144	Gm7968	predicted gene	TEC	21	38	18	51	55	73	47	62	69	79	52	53	1.86	0.0112
ENSMUSG00000031174	Rpgr	retinitis pigmer	protein_coding	517	568	524	585	649	689	607	603	647	648	599	654	1.16	0.00343
ENSMUSG00000097430	Gm10544	predicted gene	lncRNA	20	30	16	39	56	67	45	47	64	59	42	49	2.02	0.000281
ENSMUSG00000069631	Strada	STE20-related k	protein_coding	491	563	452	560	593	654	572	594	628	623	577	636	1.17	0.00364
ENSMUSG00000100813	Gm28874	predicted gene	lncRNA	37	52	23	47	64	76	60	55	67	69	46	68	1.6	0.00508
ENSMUSG00000038010	Ccdc138	coiled-coil dom	protein_coding	177	249	189	294	255	284	290	258	305	290	252	274	1.19	0.0238
ENSMUSG00000048038	Ccdc187	coiled-coil dom	protein_coding	425	714	556	732	678	855	880	761	881	875	739	790	1.31	0.00491
ENSMUSG00000055065	Ddx17	DEAD box helic	protein_coding	10,990	13,210	11,610	13,380	13,422	14,527	13,704	13,704	14,744	14,848	13,434	13,364	1.13	0.00903
ENSMUSG00000032267	Usp28	ubiquitin specif	protein_coding	278	383	276	414	424	490	505	472	447	543	422	522	1.4	0.000447
ENSMUSG00000028957	Per3	period circadiar	protein_coding	1,016	1,275	1,009	1,226	1,349	1,477	1,401	1,368	1,463	1,498	1,360	1,348	1.24	0.000133
ENSMUSG00000037818	Abhd18	abhydrolase do	protein_coding	472	653	478	668	691	741	809	761	777	820	705	703	1.32	0.000677
ENSMUSG00000103672	Gm37621	predicted gene,	TEC	85	117	71	140	121	162	152	134	153	120	144	164	1.37	0.00425
ENSMUSG00000025982	Sf3b1	splicing factor 3	protein_coding	6,880	7,892	6,604	7,997	8,031	8,690	8,045	7,999	8,626	8,161	8,110	8,348	1.12	0.00455
ENSMUSG00000017677	Wsb1	WD repeat and	protein_coding	2,850	3,624	2,615	3,811	3,589	4,290	3,744	3,821	3,912	4,017	3,790	4,162	1.2	0.0049
ENSMUSG00000106683	Gm43263	predicted gene	lncRNA	38	93	30	104	107	126	115	107	123	122	114	131	1.71	0.00626
ENSMUSG00000055897	Ppp4r1l-ps	protein phosph	transcribed_unprocess	508	741	536	712	723	861	734	801	864	795	749	787	1.25	0.00638
ENSMUSG00000045672	Col27a1	collagen, type X	protein_coding	252	346	234	363	350	443	397	416	429	412	399	402	1.35	0.000023
ENSMUSG00000112095	Sros1	non-coding RNA	lncRNA	177	266	173	289	283	367	340	318	360	335	322	337	1.44	0.00147
ENSMUSG00000081471	Gm14735	predicted gene	processed_pseudoge	93	139	113	185	181	224	182	210	184	228	195	189	1.5	0.000182
ENSMUSG00000054387	Mdm4	transformed m	protein_coding	2,458	3,401	2,780	3,852	3,852	4,135	3,597	3,785	3,756	3,928	3,906	3,589	1.23	0.00334
ENSMUSG00000058006	Mdn1	midasin AAA AT	protein_coding	3,122	4,577	3,526	4,737	4,928	5,857	5,035	5,337	4,973	5,223	5,519	5,112	1.33	0.000139
ENSMUSG00000097451	Rian	RNA imprinted	lncRNA	19,782	22,427	20,032	23,643	23,935	25,125	23,932	24,373	24,520	24,797	25,278	24,163	1.13	0.0000959
ENSMUSG00000091476	Catspere2	cation channel	protein_coding	98	130	113	159	158	189	184	183	175	169	168	175	1.42	0.000993
ENSMUSG00000034462	Pkd2	polycystin 2, tr	protein_coding	1,231	1,425	1,249	1,628	1,614	1,790	1,718	1,646	1,684	1,639	1,706	1,631	1.22	0.00268
ENSMUSG00000107000	Gm43481	predicted gene	TEC	89	148	101	187	172	219	191	180	209	194	184	201	1.45	0.00311
ENSMUSG00000108350	Gm44950	predicted gene	TEC	279	378	328	481	463	481	440	456	477	440	449	460	1.25	0.00315
ENSMUSG00000112990	Gm47372	predicted gene,	TEC	74	118	93	151	155	161	159	143	151	163	147	167	1.41	0.00698
ENSMUSG00000042564	Fam227a	family with seq	protein_coding	258	361	239	391	411	471	433	379	415	436	460	464	1.35	0.000375
ENSMUSG00000048271	Rbm33	RNA binding m	protein_coding	2,706	3,507	2,693	3,727	3,647	4,016	3,481	3,621	3,741	3,746	3,880	3,781	1.17	0.0142
ENSMUSG00000106826	Gm42583	predicted gene	TEC	41	96	44	115	126	131	105	110	102	120	120	135	1.59	0.00672
ENSMUSG00000110779	Gm48271	predicted gene,	TEC	679	1,050	693	1,131	1,169	1,095	1,161	1,160	1,263	1,091	1,266	1,096	1.29	0.00487
ENSMUSG00000107840	Gm44241	predicted gene,	TEC	0	3	0	9	10	10	12	14	14	13	9	10	3.73	0.00221
ENSMUSG00000103697	Gm38020	predicted gene,	TEC	386	679	343	856	804	879	789	806	843	864	871	768	1.45	0.00797
ENSMUSG00000086370	Ftx	Ftx transcript, X	lncRNA	1,153	1,610	1,139	1,906	1,931	1,936	1,915	1,976	1,932	1,875	1,964	1,897	1.33	0.00103
ENSMUSG00000100826	Snhg14	small nucleolar	lncRNA	4,607	10,475	4,610	13,106	13,237	15,878	13,191	15,126	15,546	15,431	15,284	14,498	1.75	0.00332
ENSMUSG00000020599	Rgs9	regulator of G-p	protein_coding	1,201	1,262	1,235	1,406	1,330	1,553	1,590	1,416	1,494	1,459	1,503	1,409	1.15	0.00323
ENSMUSG00000037791	Phf12	PHD finger prot	protein_coding	1,530	1,584	1,420	1,857	1,668	1,906	1,933	1,781	1,904	1,846	1,790	1,815	1.14	0.00662
ENSMUSG00000032705	Exd2	exonuclease 3'-	protein_coding	1,289	1,393	1,239	1,614	1,539	1,704	1,798	1,655	1,743	1,772	1,695	1,521	1.21	0.00664
ENSMUSG00000102780	Gm38253	predicted gene,	TEC	69	86	56	109	98	122	132	102	127	136	108	101	1.41	0.00597
ENSMUSG00000001986	Gria3	glutamate rece	protein_coding	2,668	2,751	2,625	3,050	3,061	2,947	3,156	2,997	3,077	2,993	3,059	2,904	1.1	0.00133
ENSMUSG00000024384	Iws1	IWS1, SUPT6 in	protein_coding	1,288	1,360	1,171	1,643	1,695	1,530	1,812	1,728	1,663	1,556	1,929	1,599	1.24	0.00356
ENSMUSG00000026812	Tsc1	TSC complex su	protein_coding	2,571	2,808	2,500	2,873	2,973	3,027	3,068	2,923	2,950	3,138	3,035	2,866	1.11	0.000889
ENSMUSG00000069793	Slfn9	schlafen 9	protein_coding	115	142	82	146	177	169	175	146	160	177	174	154	1.36	0.00179
ENSMUSG00000019841	Rev3l	REV3 like, DNA	protein_coding	2,365	2,516	2,214	2,940	2,963	3,016	3,016	3,111	2,793	3,131	3,065	2,902	1.21	0.000188
ENSMUSG00000084372	Gm13988	predicted gene	processed_pseudoge	23	34	21	41	60	56	58	59	58	68	55	56	1.95	0.0000634
ENSMUSG00000104342	Gm36401	predicted gene,	TEC	8	14	9	20	29	31	28	32	30	28	33	27	2.35	0.000168
ENSMUSG00000026153	Fam135a	family with seq	protein_coding	929	932	1,002	1,132	1,136	1,119	1,030	1,189	1,143	1,105	1,075	1,207	1.12	0.00816
ENSMUSG00000042249	Grk3	G protein-coup	protein_coding	2,735	3,100	3,082	3,375	3,487	3,472	3,285	3,556	3,405	3,507	3,323	3,552	1.12	0.000866
ENSMUSG00000041890	Git2	GIT ArfGAP 2	protein_coding	1,234	1,302	1,329	1,432	1,513	1,647	1,430	1,524	1,475	1,498	1,516	1,582	1.15	0.000152
ENSMUSG00000108557	Gm44835	predicted gene	TEC	13	20	20	33	40	51	24	34	35	31	39	43	1.74	0.00604
ENSMUSG00000035107	Dcbld2	discoidin, CUB	protein_coding	856	976	928	1,457	1,407	1,808	1,272	1,386	1,357	1,365	1,534	1,668	1.39	0.000673
ENSMUSG00000106001	Gm42826	predicted gene	TEC	13	13	12	51	48	64	31	48	48	37	51	51	2.13	0.00123
ENSMUSG00000029328	Hnrnpdl	heterogeneous	protein_coding	3,749	4,120	3,773	4,758	4,911	5,198	4,262	4,787	4,783	4,948	4,713	5,161	1.17	0.00355
ENSMUSG00000096039	D830030K20Rik	RIKEN cDNA D8	protein_coding	262	309	264	419	399	451	337	405	404	418	401	421	1.27	0.00469
ENSMUSG00000052707	Tnrc6a	trinucleotide re	protein_coding	4,786	5,397	4,749	6,634	6,562	6,658	5,640	6,700	6,224	6,432	6,703	6,522	1.19	0.00454
ENSMUSG00000097986	Gm26953	predicted gene,	lncRNA	86	112	68	161	211	195	127	198	173	184	212	213	1.71	0.00359
ENSMUSG00000035649	Zcchc7	zinc finger, CCH	protein_coding	1,363	1,630	1,407	2,141	2,063	2,310	2,106	2,102	2,056	2,077	2,202	2,535	1.31	0.000976
ENSMUSG00000048154	Kmt2d	lysine (K)-speci	protein_coding	4,415	5,264	4,492	7,128	6,753	7,649	6,400	6,723	6,488	6,807	7,375	7,671	1.29	0.00183
ENSMUSG00000094488	Or1e33	olfactory recep	protein_coding	86	136	80	187	196	224	169	191	185	177	202	215	1.59	0.0011
ENSMUSG00000104498	Gm37110	predicted gene,	TEC	204	284	221	415	405	449	328	382	391	361	420	455	1.39	0.00369
ENSMUSG00000043314	Or2z2	olfactory recep	protein_coding	20	28	21	52	60	68	41	58	59	41	70	77	1.87	0.00255
ENSMUSG00000028292	Rars2	arginyl-tRNA sy	protein_coding	387	467	422	515	553	589	485	531	513	544	558	603	1.21	0.00179
ENSMUSG00000092341	Malat1	metastasis asso	lncRNA	12,023	18,949	11,149	30,797	37,771	44,677	24,062	33,661	29,624	32,663	43,037	51,141	1.92	0.00154
ENSMUSG00000102349	Gm37376	predicted gene,	TEC	330	411	298	713	925	1,093	691	905	743	790	981	1,289	2.06	0.00196
ENSMUSG00000021431	Snrnp48	small nuclear ri	protein_coding	784	776	702	939	888	1,061	843	1,022	905	863	1,030	1,016	1.19	0.003
ENSMUSG00000028402	Mpdz	multiple PDZ do	protein_coding	2,287	2,195	2,082	2,906	2,704	3,196	2,974	3,089	2,634	2,457	3,349	3,346	1.26	0.0032
ENSMUSG00000019873	Reep3	receptor access	protein_coding	1,855	1,834	1,627	2,012	2,049	2,162	2,073	2,015	2,142	2,079	2,103	2,126	1.13	0.00798
ENSMUSG00000079070	Gm3985	predicted gene	lncRNA	17	20	9	31	39	37	37	33	40	35	41	33	1.86	0.0012
ENSMUSG00000108199	Gm44249	predicted gene,	TEC	44	38	24	75	73	97	64	75	89	70	70	80	1.7	0.00456
ENSMUSG00000109105	Gm44873	predicted gene	TEC	64	66	38	101	92	110	87	101	101	99	106	96	1.44	0.0123
ENSMUSG00000053925	Gm9929	predicted gene	lncRNA	60	66	51	115	121	115	94	105	120	97	125	113	1.48	0.00417
ENSMUSG00000082765	Gm14411	predicted gene	unprocessed_pseudo	20	28	18	53	67	75	41	55	58	46	69	51	1.97	0.000985
ENSMUSG00000115148	Gm49125	predicted gene,	TEC	50	83	35	133	148	139	115	146	146	113	132	153	1.82	0.00113
ENSMUSG00000059429	Or1l4	olfactory recep	protein_coding	301	387	270	511	533	630	479	563	595	531	552	583	1.5	0.000045
ENSMUSG00000075383	Or1n1	olfactory recep	protein_coding	70	101	74	137	146	157	134	155	153	150	151	153	1.55	0.00258
ENSMUSG00000079177	Fam228a	family with seq	protein_coding	48	73	41	110	113	139	99	105	101	110	121	106	1.67	0.00133
ENSMUSG00000112633	Gm47627	predicted gene,	TEC	36	59	29	104	118	123	81	88	95	105	133	108	1.79	0.00317
ENSMUSG00000037876	Jmjd1c	jumonji domair	protein_coding	2,866	2,954	2,744	3,568	3,514	3,714	3,408	3,533	3,470	3,488	3,687	3,606	1.17	0.00196
ENSMUSG00000056673	Kdm5d	lysine (K)-speci	protein_coding	1,061	1,150	1,003	1,366	1,345	1,468	1,247	1,416	1,403	1,385	1,447	1,395	1.19	0.000158
ENSMUSG00000030002	Dusp11	dual specificity	protein_coding	1,790	2,287	1,686	3,021	2,882	3,329	2,766	3,133	2,923	3,000	3,562	3,265	1.38	0.00102
ENSMUSG00000085028	Slc2a4rg-ps	Slc2a4 regulato	transcribed_unitary_	97	142	85	176	190	217	183	217	192	199	235	210	1.61	0.0000568
ENSMUSG00000110519	Or7g23	olfactory recep	protein_coding	135	180	108	268	265	293	243	253	279	250	294	276	1.52	0.00268
ENSMUSG00000032525	Nktr	natural killer tu	protein_coding	3,971	5,422	3,793	7,091	6,832	7,520	6,901	7,219	7,345	7,167	7,929	7,634	1.4	0.000466
ENSMUSG00000074733	Zfp950	zinc finger prot	protein_coding	1,445	1,723	1,363	2,174	2,253	2,267	2,133	2,160	2,189	2,306	2,438	2,246	1.31	0.000122
ENSMUSG00000087662	Gm11373	predicted gene	lncRNA	29	25	14	39	58	56	33	34	44	48	46	51	1.67	0.00895
ENSMUSG00000108532	Gm32647	predicted gene,	lncRNA	9	22	3	17	38	41	13	20	31	32	25	27	2.1	0.00966
ENSMUSG00000102995	A330074H02Rik	RIKEN cDNA A3	TEC	12	14	9	20	33	30	22	26	37	16	26	28	1.99	0.00407
ENSMUSG00000026096	Osgepl1	O-sialoglycopro	protein_coding	381	398	354	446	531	500	435	476	509	487	481	474	1.23	0.00179
ENSMUSG00000105748	Gm43088	predicted gene	TEC	17	20	6	21	48	39	23	35	49	33	39	35	2.24	0.000305
ENSMUSG00000087306	A230004M16Rik	RIKEN cDNA A2	lncRNA	13	15	11	26	40	25	31	24	27	39	28	33	1.86	0.00665
ENSMUSG00000068206	Pick1	protein interact	protein_coding	890	961	844	949	1,085	1,011	1,039	996	1,024	1,051	982	1,157	1.13	0.00575
ENSMUSG00000103039	Gm37123	predicted gene,	TEC	29	28	21	43	59	51	47	35	51	48	36	66	1.57	0.0124
ENSMUSG00000026074	Map4k4	mitogen-activa	protein_coding	3,359	3,339	3,222	3,721	4,075	4,103	4,048	3,691	3,870	3,659	3,947	4,149	1.17	0.0000831
ENSMUSG00000028556	Dock7	dedicator of cy	protein_coding	1,422	1,461	1,442	1,911	1,908	2,211	1,962	1,773	2,015	1,945	1,789	2,000	1.26	0.00162
ENSMUSG00000109501	Gm14377	predicted gene	lncRNA	15	27	8	48	43	54	51	49	35	54	57	67	2.02	0.00566
ENSMUSG00000038884	Shfl	shiftless antivir	protein_coding	2,097	2,256	1,870	2,548	2,624	2,835	2,410	2,500	2,416	2,601	2,719	2,917	1.18	0.00206
ENSMUSG00000072778	Vmn2r27	vomeronasal 2,	protein_coding	9	15	6	25	28	33	26	26	22	24	26	38	1.97	0.00418
ENSMUSG00000068328	Aup1	ancient ubiquit	protein_coding	1,014	1,032	939	1,060	1,194	1,215	1,179	1,160	1,162	1,153	1,156	1,236	1.17	0.00002
ENSMUSG00000025616	Usp16	ubiquitin specif	protein_coding	644	701	564	722	856	805	773	800	828	806	758	899	1.23	0.00216
ENSMUSG00000046111	Cep295	centrosomal pr	protein_coding	839	983	654	983	1,153	1,184	1,141	1,171	1,164	1,084	1,083	1,254	1.35	0.0000834
ENSMUSG00000111946	Gm47495	predicted gene,	processed_pseudoge	7	11	2	15	24	25	17	13	24	15	20	25	2.24	0.00811
ENSMUSG00000115632	Gm17922	predicted gene,	processed_pseudoge	9	17	8	25	44	38	28	29	36	30	31	54	2.33	0.00216
ENSMUSG00000056267	Cep70	centrosomal pr	protein_coding	492	572	531	650	748	749	592	649	707	642	666	713	1.22	0.00362
ENSMUSG00000097877	Gm26703	predicted gene,	lncRNA	136	180	167	212	252	266	174	220	267	224	211	238	1.31	0.0046
ENSMUSG00000105155	Gm42910	predicted gene	TEC	4	8	8	15	21	25	15	15	19	21	22	21	2.18	0.0054
ENSMUSG00000106671	Gm42900	predicted gene	TEC	39	69	42	89	112	123	83	88	112	108	101	105	1.69	0.000826
ENSMUSG00000044072	Eml6	echinoderm mi	protein_coding	966	1,347	1,105	1,558	1,824	1,861	1,381	1,543	1,689	1,613	1,667	1,700	1.33	0.00106
ENSMUSG00000109787	Gm45286	predicted gene	TEC	46	87	68	109	119	138	95	110	124	117	121	123	1.48	0.00138
ENSMUSG00000025650	Col7a1	collagen, type V	protein_coding	54	99	122	111	187	244	102	151	173	173	187	183	1.78	0.00103
ENSMUSG00000018581	Dnah11	dynein, axonem	protein_coding	128	180	171	213	308	330	212	284	293	315	314	221	1.63	0.00018
ENSMUSG00000104080	Gm38279	predicted gene,	TEC	138	183	147	199	260	261	173	244	230	257	233	223	1.41	0.000105
ENSMUSG00000040033	Stat2	signal transduc	protein_coding	551	718	637	687	820	837	750	735	742	796	886	801	1.21	0.00773
ENSMUSG00000043286	Pnpla1	patatin-like pho	protein_coding	3	8	6	12	25	17	15	13	16	18	24	14	2.4	0.00395
ENSMUSG00000045225	Or5w19	olfactory recep	protein_coding	70	105	89	123	179	148	127	128	132	147	170	163	1.5	0.000777
ENSMUSG00000024871	Doc2g	double C2, gam	protein_coding	27	51	30	42	74	81	53	38	68	56	71	60	1.61	0.00626
ENSMUSG00000027185	Nat10	N-acetyltransfe	protein_coding	653	847	652	946	1,235	1,160	936	908	1,061	923	1,155	1,077	1.37	0.000267
ENSMUSG00000044906	4930503L19Rik	RIKEN cDNA 49	protein_coding	266	306	322	357	441	448	393	367	431	383	452	361	1.31	0.0000833
ENSMUSG00000085133	B930095G15Rik	RIKEN cDNA B9	lncRNA	1,272	1,528	1,456	1,642	1,652	1,783	1,688	1,607	1,707	1,666	1,820	1,583	1.14	0.00663
ENSMUSG00000100555	Gm8173	predicted gene	processed_pseudoge	0	3	6	20	19	34	24	14	26	26	30	17	3.11	0.00133
ENSMUSG00000111481	Gm47652	predicted gene,	TEC	1,604	2,316	1,836	2,720	2,642	3,047	2,569	2,770	2,762	2,609	3,260	2,878	1.3	0.00236
ENSMUSG00000028391	Wdr31	WD repeat dom	protein_coding	576	710	630	786	804	854	783	837	798	797	884	895	1.21	0.00283
ENSMUSG00000095959	Gm10845	predicted gene	lncRNA	255	343	284	440	453	533	418	505	477	443	575	519	1.44	0.0000709
ENSMUSG00000103182	Gm37091	predicted gene,	TEC	21	41	32	55	64	73	54	64	53	47	73	69	1.72	0.00203
ENSMUSG00000105302	Gm19817	predicted gene,	TEC	0	6	2	11	21	20	13	12	14	10	20	16	3.64	0.00169
ENSMUSG00000117001	Gm49858	predicted gene,	processed_pseudoge	27	86	59	111	201	182	90	175	163	110	193	133	2.3	0.0000204
ENSMUSG00000104925	Gm43061	predicted gene	lncRNA	3	7	8	15	19	21	17	23	16	17	25	18	2.51	0.00158
ENSMUSG00000085802	Gm16059	predicted gene	lncRNA	1	6	5	11	16	14	12	12	15	12	21	12	2.44	0.0106
ENSMUSG00000112530	Gm47738	predicted gene,	lncRNA	177	232	218	303	367	342	322	327	330	296	384	306	1.46	0.000171
ENSMUSG00000007411	Mark3	MAP/microtubu	protein_coding	1,416	1,474	1,366	1,456	1,536	1,620	1,640	1,571	1,638	1,576	1,603	1,564	1.11	0.000814
ENSMUSG00000038775	Vill	villin-like	protein_coding	93	86	71	73	107	155	126	129	134	111	124	123	1.59	0.000196
ENSMUSG00000020115	Tbk1	TANK-binding k	protein_coding	1,135	1,097	948	1,250	1,291	1,654	1,350	1,385	1,292	1,448	1,448	1,305	1.28	0.0000593
ENSMUSG00000108027	Gm44271	predicted gene,	lncRNA	16	8	8	16	24	35	27	21	30	30	32	27	2.2	0.000818
ENSMUSG00000086589	Gm12915	predicted gene	lncRNA	16	10	5	19	23	37	26	35	31	29	42	26	2.43	0.000214
ENSMUSG00000098395				89	86	71	108	158	172	161	182	156	176	203	174	1.9	0.00000968
ENSMUSG00000014039	Prdm15	PR domain con	protein_coding	345	451	345	370	475	666	466	502	461	477	640	472	1.39	0.00136
ENSMUSG00000027522	Stx16	syntaxin 16	protein_coding	3,134	3,252	3,150	3,193	3,350	3,715	3,313	3,508	3,437	3,417	3,504	3,438	1.09	0.000476
ENSMUSG00000097399	Gm26555	predicted gene,	lncRNA	7	7	7	7	14	43	11	24	15	25	35	21	3.19	0.000441
ENSMUSG00000002055	Spag5	sperm associat	protein_coding	163	251	190	261	284	339	241	292	254	279	335	322	1.34	0.0125
ENSMUSG00000113847	Gm48799	predicted gene,	lncRNA	185	333	227	284	428	525	287	374	327	321	501	454	1.57	0.00264
ENSMUSG00000081974	Gm11960	predicted gene	transcribed_processe	78	115	81	117	142	193	118	135	156	123	160	171	1.49	0.00376
ENSMUSG00000107168	Gm42507	predicted gene	TEC	5	10	8	11	18	25	14	17	17	17	21	23	2.19	0.00525
ENSMUSG00000024542	Cep192	centrosomal pr	protein_coding	316	378	334	506	479	585	450	440	486	508	510	479	1.27	0.00757
ENSMUSG00000108857	Gm44578	predicted gene	processed_pseudoge	16	21	9	48	52	75	35	37	58	46	57	45	2.08	0.00247
ENSMUSG00000093866	Or6c1	olfactory recep	protein_coding	13	17	9	23	31	40	20	27	29	27	35	31	1.85	0.00668
ENSMUSG00000075081	Or4a74	olfactory recep	protein_coding	163	238	155	261	275	379	249	278	317	305	303	289	1.44	0.000891
ENSMUSG00000093385	A330044P14Rik	RIKEN cDNA A3	transcribed_unproce	56	75	51	99	87	147	91	99	108	102	119	111	1.5	0.00396
ENSMUSG00000103032	Gm37121	predicted gene,	TEC	8	18	8	19	24	45	24	29	23	31	32	35	2.3	0.000304
ENSMUSG00000006241	Ccdc159	coiled-coil dom	protein_coding	129	180	122	162	204	250	193	224	217	219	227	205	1.46	0.0000119

ENSMUSG00000111312	Gm47205	predicted gene,	TEC	15	17	18	25
ENSMUSG00000105852	Gm42890	predicted gene	lncRNA	48	55	60	84
ENSMUSG00000030243	Recql	RecQ protein-lil	protein_coding	446	462	533	476
ENSMUSG00000106995	Gm33167	predicted gene,	TEC	45	49	57	61
ENSMUSG00000097352	C920009B18Rik	RIKEN cDNA C9	transcribed_unproce	8	1	9	2
ENSMUSG00000038126	Mphosph9	M-phase phosp	protein_coding	617	490	522	705
ENSMUSG00000032198	Dock6	dedicator of cyt	protein_coding	509	482	474	632
ENSMUSG00000098024	Gm27003	predicted gene,	lncRNA	12	4	9	15
ENSMUSG00000084964	Gm15503	predicted gene	lncRNA	1	5	9	1
ENSMUSG00000022369	Mtbp	Mdm2, transfor	protein_coding	36	56	48	52
ENSMUSG00000022160	Mettl3	methyltransfera	protein_coding	428	473	435	432
ENSMUSG00000073274	Gm14636	predicted gene	lncRNA	0	2	1	2
ENSMUSG00000045455	Gm9797	predicted pseu	transcribed_processe	0	3	0	0
ENSMUSG00000106951	5930430L01Rik	RIKEN cDNA 59	lncRNA	96	104	83	103
ENSMUSG00000098120	Gm5914	predicted gene	lncRNA	17	17	16	17
ENSMUSG00000100213	Gm28151	predicted gene	lncRNA	48	44	41	41
ENSMUSG00000104876	Trdc	T cell receptor (	TR_C_gene	9	20	12	19
ENSMUSG00000107096	Gm43597	predicted gene	lncRNA	373	419	445	451
ENSMUSG00000021481	Zfp346	zinc finger prot	protein_coding	445	553	486	500
ENSMUSG00000035158	Mitf	melanogenesis	protein_coding	137	157	141	154
ENSMUSG00000091784	Gm17022	predicted gene	processed_pseudoge	12	15	18	19
ENSMUSG00000118310	Gm50268	predicted gene,	TEC	5	8	6	13
ENSMUSG00000052783	Grk4	G protein-coupl	protein_coding	144	172	165	206
ENSMUSG00000100183	Gm28512	predicted gene	lncRNA	48	67	53	67
ENSMUSG00000086907	Gm15298	predicted gene	lncRNA	61	87	60	85
ENSMUSG00000024006	Stk38	serine/threonir	protein_coding	983	1,032	982	1,288
ENSMUSG00000026305	Lrrfip1	leucine rich rep	protein_coding	1,002	1,065	985	1,092
ENSMUSG00000001768	Rin2	Ras and Rab int	protein_coding	1,012	1,119	1,047	963
ENSMUSG00000002205	Vrk3	vaccinia related	protein_coding	456	470	441	380
ENSMUSG00000079224	Gm6565	predicted gene	processed_pseudoge	0	0	0	0
ENSMUSG00000041498	Kif14	kinesin family n	protein_coding	6	4	2	5
ENSMUSG00000021209	Ppp4r4	protein phosph	protein_coding	916	933	936	887
ENSMUSG00000055296	Tmem245	transmembrane	protein_coding	3,800	3,843	3,804	4,002
ENSMUSG00000075113	Or4c29	olfactory recep	protein_coding	4	7	6	13
ENSMUSG00000033021	Gmppa	GDP-mannose	protein_coding	479	476	433	456
ENSMUSG00000073102	Drc1	dynein regulato	protein_coding	483	394	420	434
ENSMUSG00000005907	Pex1	peroxisomal bic	protein_coding	796	718	727	786
ENSMUSG00000036550	Cnot1	CCR4-NOT tran	protein_coding	4,394	4,319	3,897	4,154
ENSMUSG00000039254	Pomt1	protein-O-man	protein_coding	398	415	316	362
ENSMUSG00000029521	Chek2	checkpoint kina	protein_coding	43	57	26	102
ENSMUSG00000116908	Gm49599	predicted gene,	TEC	60	69	57	92
ENSMUSG00000056459	Zbtb25	zinc finger and	protein_coding	323	326	366	414
ENSMUSG00000070733	Fryl	FRY like transcr	protein_coding	2,547	2,511	2,693	2,840
ENSMUSG00000083734	Gm16209	predicted gene	processed_pseudoge	7	4	11	17
ENSMUSG00000028017	Egf	epidermal grow	protein_coding	39	54	40	58
ENSMUSG00000097207	6030443J06Rik	RIKEN cDNA 60	lncRNA	22	23	21	28
ENSMUSG00000111854	Gm48740	predicted gene,	lncRNA	10	25	15	23
ENSMUSG00000055072	Gm9964	predicted gene	TEC	46	53	35	59
ENSMUSG00000034761	Map4k5	mitogen-activa	protein_coding	1,134	1,133	1,082	1,401
ENSMUSG00000087620	5330434G04Rik	RIKEN cDNA 53	lncRNA	412	485	430	541
ENSMUSG00000108465	Gm45110	predicted gene	TEC	46	65	50	76
ENSMUSG00000091511	Vmn2r87	vomeronasal 2,	protein_coding	41	32	26	36
ENSMUSG00000115591	Gm31282	predicted gene,	lncRNA	50	25	39	51
ENSMUSG00000107571	Gm43877	predicted gene,	TEC	4	0	0	3
ENSMUSG00000110189	Gm45665	predicted gene	processed_pseudoge	53	25	21	54
ENSMUSG00000025878	Uimc1	ubiquitin intera	protein_coding	416	387	374	414
ENSMUSG00000009555	Cdk9	cyclin-depende	protein_coding	1,414	1,325	1,388	1,397
ENSMUSG00000027668	Mfn1	mitofusin 1	protein_coding	2,386	2,402	2,359	2,672
ENSMUSG00000023170	Gps2	G protein pathv	protein_coding	382	334	317	389
ENSMUSG00000037364	Srrt	serrate RNA eff	protein_coding	3,046	2,920	2,890	3,369
ENSMUSG00000042359	Osbpl6	oxysterol bindir	protein_coding	2,405	2,393	2,350	2,511
ENSMUSG00000021500	Ddx46	DEAD box helic	protein_coding	2,190	2,100	2,075	2,512
ENSMUSG00000031256	Cstf2	cleavage stimul	protein_coding	3,887	3,941	3,641	4,656
ENSMUSG00000037674	Rfx7	regulatory facto	protein_coding	1,729	1,730	1,731	1,969
ENSMUSG00000040867	Begain	brain-enriched	protein_coding	3,628	3,804	3,386	4,210
ENSMUSG00000040840	4930579G18Rik	RIKEN cDNA 49	lncRNA	49	44	29	61
ENSMUSG00000118501	Gm53048	predicted gene,	unprocessed_pseudo	5	4	2	9
ENSMUSG00000028820	Sfpq	splicing factor	protein_coding	5,088	5,071	4,435	5,915
ENSMUSG00000039860	Srrm3	serine/arginine	protein_coding	1,185	1,110	1,043	1,409
ENSMUSG00000094030	Gm21833	predicted gene,	protein_coding	166	131	123	265
ENSMUSG00000052752	Traf7	TNF receptor-as	protein_coding	714	686	653	895
ENSMUSG00000060445	Sycp2	synaptonemal c	protein_coding	27	27	18	70
ENSMUSG00000066760	Psg16	pregnancy spec	protein_coding	1,176	1,133	1,212	1,452
ENSMUSG00000074292	Gm10660	predicted gene	lncRNA	15	18	24	67
ENSMUSG00000028637	Ccdc30	coiled-coil dom	protein_coding	519	489	537	629
ENSMUSG00000052676	Zmat1	zinc finger, mat	protein_coding	1,003	902	990	1,313
ENSMUSG00000111436	Gm47356	predicted gene,	processed_pseudoge	58	58	62	121
ENSMUSG00000041685	Fcho2	FCH domain on	protein_coding	1,382	1,416	1,430	1,646
ENSMUSG00000033981	Gria2	glutamate rece	protein_coding	11,724	11,633	11,714	13,735
ENSMUSG00000111939	Gm48177	predicted gene,	processed_pseudoge	123	135	135	284
ENSMUSG00000000838	Fmr1	fragile X messe	protein_coding	1,434	1,354	1,308	1,585
ENSMUSG00000078868	Gm14412	predicted gene	protein_coding	78	62	44	100
ENSMUSG00000013236	Ptprs	protein tyrosine	protein_coding	8,842	8,729	7,441	10,645
ENSMUSG00000028180	Zranb2	zinc finger, RAN	protein_coding	4,289	3,883	3,716	4,958
ENSMUSG00000020069	Hnrnph3	heterogeneous	protein_coding	1,550	1,441	1,396	1,744
ENSMUSG00000112158	Gm47822	predicted gene,	TEC	72	65	53	142
ENSMUSG00000018537	Pcgf2	polycomb grou	protein_coding	751	669	674	844
ENSMUSG00000021493	Pdlim7	PDZ and LIM dc	protein_coding	775	698	679	806
ENSMUSG00000035299	Mid1	midline 1	protein_coding	364	304	317	392
ENSMUSG00000004980	Hnrnpa2b1	heterogeneous	protein_coding	12,487	12,713	12,800	14,119
ENSMUSG00000037519	Ppfia1	protein tyrosine	protein_coding	1,220	1,289	1,229	1,505
ENSMUSG00000025261	Huwe1	HECT, UBA and	protein_coding	17,042	16,487	17,072	22,030
ENSMUSG00000096808			protein_coding	275	246	275	383
ENSMUSG00000018379	Srsf1	serine and argir	protein_coding	5,642	5,640	5,429	6,522
ENSMUSG00000020464	Pnpt1	polyribonucleot	protein_coding	763	739	702	885
ENSMUSG00000020982	Nemf	nuclear export	protein_coding	2,797	2,682	2,631	3,122
ENSMUSG00000104046	Gm37567	predicted gene,	TEC	871	879	833	1,075
ENSMUSG00000026116	Tmem131	transmembrane	protein_coding	3,093	3,090	2,983	3,264
ENSMUSG00000117286	Gm1043	predicted gene	protein_coding	1,548	1,622	1,502	1,999
ENSMUSG00000025747	Tyms	thymidylate syn	protein_coding	254	273	251	354
ENSMUSG00000036057	Ptpn23	protein tyrosine	protein_coding	2,177	2,142	1,989	2,323
ENSMUSG00000025241	Fyco1	FYVE and coilec	protein_coding	2,765	2,783	2,650	3,139
ENSMUSG00000040225	Prrc2c	proline-rich coil	protein_coding	9,696	9,621	9,145	11,307
ENSMUSG00000020640	Itsn2	intersectin 2	protein_coding	2,040	2,016	2,104	2,337
ENSMUSG00000019996	Map7	microtubule-as	protein_coding	2,264	1,992	1,978	2,579
ENSMUSG00000034243	Golgb1	golgi autoantig	protein_coding	7,363	6,832	7,086	7,894
ENSMUSG00000111485	Gm48443	predicted gene,	processed_pseudoge	742	645	730	1,042
ENSMUSG00000029004	Kmt2e	lysine (K)-specif	protein_coding	4,475	3,957	4,084	5,186
ENSMUSG00000053510	Nrd1	nardilysin, N-ar	protein_coding	3,790	3,144	3,330	4,311
ENSMUSG00000071054	Safb	scaffold attachr	protein_coding	3,442	3,264	3,396	4,130
ENSMUSG00000104837	Gm42520	predicted gene	TEC	81	83	80	136
ENSMUSG00000069892	9930111J21Rik2	RIKEN cDNA 99	protein_coding	469	419	402	553
ENSMUSG00000102326	Gm37788	predicted gene,	TEC	151	139	125	220
ENSMUSG00000106631	Gm42669	predicted gene	protein_coding	511	503	485	623
ENSMUSG00000025272	Tro	trophinin	protein_coding	11,335	11,292	10,583	13,784
ENSMUSG00000002028	Kmt2a	lysine (K)-specif	protein_coding	8,639	8,286	7,837	10,298
ENSMUSG00000037541	Shank2	SH3 and multip	protein_coding	2,666	2,683	2,512	3,094
ENSMUSG00000038346	Zfp384	zinc finger prot	protein_coding	858	846	815	1,108
ENSMUSG00000040721	Zfhx2	zinc finger hom	protein_coding	2,875	2,773	2,553	3,254
ENSMUSG00000112122	Gm47628	predicted gene,	processed_pseudoge	147	131	93	245
ENSMUSG00000010608	Rbm25	RNA binding m	protein_coding	3,715	3,841	3,228	4,755
ENSMUSG00000063511	Snrnp70	small nuclear ri	protein_coding	6,884	6,983	6,008	8,935
ENSMUSG00000028809	Srrm1	serine/arginine	protein_coding	4,745	4,541	3,839	6,287
ENSMUSG00000113795	Gm48119	predicted gene,	processed_pseudoge	750	702	652	1,143
ENSMUSG00000113393	Gm48415	predicted gene,	processed_pseudoge	992	1,002	818	1,738
ENSMUSG00000061331	Gm17132	predicted gene	unprocessed_pseudo	552	568	478	831
ENSMUSG00000112049	Gm48269	predicted gene,	processed_pseudoge	289	359	280	597
ENSMUSG00000022185	Acin1	apoptotic chror	protein_coding	3,191	3,366	3,027	4,134
ENSMUSG00000020863	Luc7l3	LUC7-like 3 (S. c	protein_coding	3,881	4,007	3,778	4,754
ENSMUSG00000020994	Pnn	pinin	protein_coding	2,290	2,352	2,193	3,025
ENSMUSG00000108847	B830042I05Rik	RIKEN cDNA B8	lncRNA	163	183	158	325
ENSMUSG00000111472	Gm47103	predicted gene,	processed_pseudoge	35	28	27	108
ENSMUSG00000026339	Ccdc93	coiled-coil dom	protein_coding	1,095	1,119	943	1,105
ENSMUSG00000042909	Or52h1	olfactory recep	protein_coding	24	38	20	35
ENSMUSG00000014633	Cmc2	COX assembly r	protein_coding	181	198	162	216
ENSMUSG00000066838	Zfp772	zinc finger prot	protein_coding	295	344	255	353
ENSMUSG00000035051	Dhx57	DEAH (Asp-Glu-	protein_coding	1,477	1,480	1,371	1,678
ENSMUSG00000039205	Ciz1	CDKN1A interac	protein_coding	1,067	1,009	880	1,276
ENSMUSG00000113161	Gm47457	predicted gene,	TEC	635	607	472	832
ENSMUSG00000100750	Gm29084	predicted gene	lncRNA	1,394	1,443	1,165	1,700
ENSMUSG00000108173	Gm44231	predicted gene,	TEC	850	936	747	1,089
ENSMUSG00000030138	Bms1	BMS1, ribosom	protein_coding	871	958	781	1,018
ENSMUSG00000034799	Unc13a	unc-13 homolo	protein_coding	4,191	4,777	3,889	4,978
ENSMUSG00000066129	Kndc1	kinase non-cata	protein_coding	4,928	5,170	4,939	5,329
ENSMUSG00000027411	Vps16	VSP16 CORVET/	protein_coding	930	923	985	1,093
ENSMUSG00000031161	Hdac6	histone deacety	protein_coding	1,154	1,129	1,319	1,505
ENSMUSG00000003810	Mast2	microtubule as	protein_coding	3,566	3,490	3,599	3,733
ENSMUSG00000105694	Gm43237	predicted gene	TEC	1	1	2	5
ENSMUSG00000110170	St6galnac2	ST6 (alpha-N-ac	protein_coding	21	8	14	21
ENSMUSG00000019977	Hbs1l	Hbs1-like (S. ce	protein_coding	1,003	907	1,019	1,277
ENSMUSG00000073436	Eme2	essential meiot	protein_coding	413	425	445	520
ENSMUSG00000111325	Gm47140	predicted gene,	lncRNA	126	138	117	229
ENSMUSG00000108803	4930533N22Rik	RIKEN cDNA 49	lncRNA	495	519	503	782
ENSMUSG00000109887	Gm28756	predicted gene	lncRNA	257	260	231	377
ENSMUSG00000040250	Ints13	integrator com	protein_coding	532	558	547	723
ENSMUSG00000086130	Gm16211	predicted gene	lncRNA	255	312	295	393
ENSMUSG00000055436	Srsf11	serine and argir	protein_coding	2,967	3,315	2,933	3,914
ENSMUSG00000097397	Gm16861	predicted gene,	lncRNA	45	55	39	84
ENSMUSG00000033004	Mycbp2	MYC binding pr	protein_coding	15,070	16,360	14,872	18,191
ENSMUSG00000091076	Vmn2r115	vomeronasal 2,	protein_coding	278	357	288	571
ENSMUSG00000021134	Srsf5	serine and argir	protein_coding	6,516	7,117	6,635	7,961
ENSMUSG00000103694	Gm37530	predicted gene,	TEC	142	197	150	243
ENSMUSG00000103234	Gm37158	predicted gene,	TEC	236	285	215	438
ENSMUSG00000117599	Gm49971	predicted gene,	TEC	231	297	185	455
ENSMUSG00000094472	Gm21897	predicted gene,	protein_coding	400	575	391	853
ENSMUSG00000111269	Gm47933	predicted gene,	processed_pseudoge	169	219	138	385
ENSMUSG00000108088	Gm44229	predicted gene,	TEC	8	11	6	16
ENSMUSG00000105471	A430073D23Rik	RIKEN cDNA A4	lncRNA	17	24	11	36
ENSMUSG00000111544	4930534H03Rik	RIKEN cDNA 49	lncRNA	64	75	47	121
ENSMUSG00000090362	Vmn2r79	vomeronasal 2,	protein_coding	31	44	25	51
ENSMUSG00000102289	Gm31258	predicted gene,	TEC	27	45	29	64
ENSMUSG00000009079	Ewsr1	Ewing sarcoma	protein_coding	3,997	4,620	3,715	5,248
ENSMUSG00000097391	Mirg	miRNA containi	lncRNA	538	654	498	786
ENSMUSG00000114995	Gm49284	predicted gene,	TEC	163	157	159	181
ENSMUSG00000034820	Cpsf7	cleavage and p	protein_coding	2,459	2,474	2,406	2,789
ENSMUSG00000072739	Gm10408	predicted gene	protein_coding	0	0	0	11
ENSMUSG00000103133	Gm37303	predicted gene,	TEC	33	35	30	63
ENSMUSG00000027201	Myef2	myelin basic pr	protein_coding	2,747	2,871	2,747	3,157
ENSMUSG00000085687	Gm16153	predicted gene	lncRNA	58	59	59	100
ENSMUSG00000097911	Gm26691	predicted gene,	lncRNA	147	147	150	212
ENSMUSG00000110317	Gm45250	predicted gene	lncRNA	108	117	93	140
ENSMUSG00000038943	Prc1	protein regulat	protein_coding	60	81	56	86
ENSMUSG00000091382	Vmn1r18	vomeronasal 1	protein_coding	100	108	90	148
ENSMUSG00000045903	Npas4	neuronal PAS d	protein_coding	81	118	110	166
ENSMUSG00000114970	Gm49069	predicted gene,	TEC	32	75	44	81
ENSMUSG00000046650	Or5an6	olfactory recep	protein_coding	14	18	15	24
ENSMUSG00000105791	Gm43341	predicted gene	TEC	3	6	2	9
ENSMUSG00000109095	Gm44799	predicted gene	TEC	7	14	5	19
ENSMUSG00000001082	Mfsd10	major facilitato	protein_coding	252	268	215	319
ENSMUSG00000027981	Rnpc3	RNA-binding re	protein_coding	1,149	1,316	1,046	1,635
ENSMUSG00000108713	Gm33027	predicted gene,	lncRNA	105	125	87	163
ENSMUSG00000105476	Gm35439	predicted gene,	lncRNA	70	89	71	145
ENSMUSG00000021767	Kat6b	K(lysine) acetyl	protein_coding	1,615	1,757	1,592	1,838
ENSMUSG00000116718	Gm49668	predicted gene,	lncRNA	102	137	99	186
ENSMUSG00000021611	Tert	telomerase rev	protein_coding	84	114	85	210
ENSMUSG00000109390	Gm45718	predicted gene	TEC	53	67	54	100
ENSMUSG00000115276	9930017N22Rik	RIKEN cDNA 99	TEC	44	56	51	88
ENSMUSG00000035151	Elmod2	ELMO/CED-12 c	protein_coding	791	883	832	1,097
ENSMUSG00000051832	E230016K23Rik	RIKEN cDNA E2	lncRNA	8	21	15	41
ENSMUSG00000036403	Cep135	centrosomal pr	protein_coding	356	384	365	494
ENSMUSG00000064043	Trerf1	transcriptional	protein_coding	1,085	1,098	1,063	1,445
ENSMUSG00000033540	Idua	iduronidase, al	protein_coding	461	533	484	677
ENSMUSG00000110311	Gm45410	predicted gene	TEC	100	111	105	167
ENSMUSG00000104416	Gm37067	predicted gene,	TEC	19	28	29	65
ENSMUSG00000114722	Gm31392	predicted gene,	lncRNA	60	82	75	129
ENSMUSG00000021666	Gfm2	G elongation fa	protein_coding	588	622	556	852
ENSMUSG00000026171	Rnf25	ring finger prot	protein_coding	854	929	906	1,154
ENSMUSG00000002949	Timm44	translocase of i	protein_coding	737	828	746	904
ENSMUSG00000056211	R3hdm1	R3H domain co	protein_coding	3,991	4,032	3,898	4,520
ENSMUSG00000072647	Adam1a	a disintegrin an	protein_coding	467	620	441	737
ENSMUSG00000111585	Gm47355	predicted gene,	processed_pseudoge	45	66	38	104
ENSMUSG00000002266	Zim1	zinc finger, imp	protein_coding	2,814	2,973	2,652	3,482
ENSMUSG00000034163	Zfc3h1	zinc finger, C3H	protein_coding	1,285	1,460	1,254	1,749
ENSMUSG00000069565	Dazap1	DAZ associated	protein_coding	1,273	1,305	1,199	1,545
ENSMUSG00000028307	Aldob	aldolase B, fruc	protein_coding	31	44	27	69
ENSMUSG00000070495	Ctcfl	CCCTC-binding	protein_coding	113	143	104	180
ENSMUSG00000040165	Cd209c	CD209c antigen	protein_coding	86	94	69	120
ENSMUSG00000046056	Sbsn	suprabasin	protein_coding	152	179	125	252
ENSMUSG00000113328	Gm47260	predicted gene,	TEC	187	221	164	272
ENSMUSG00000020070	Rufy2	RUN and FYVE c	protein_coding	2,065	2,195	1,959	2,646
ENSMUSG00000043411	Usp48	ubiquitin specif	protein_coding	2,302	2,385	2,092	3,207
ENSMUSG00000030231	Plekha5	pleckstrin hom	protein_coding	1,042	1,098	995	1,361
ENSMUSG00000054142	Vmn1r236	vomeronasal 1	protein_coding	58	72	50	113
ENSMUSG00000101906	Mrgprc2-ps	MAS-related GF	unprocessed_pseudo	21	39	20	65
ENSMUSG00000113388	Gm48111	predicted gene,	processed_pseudoge	386	423	287	1,040
ENSMUSG00000114367	Gm48216	predicted gene,	processed_pseudoge	243	221	152	374
ENSMUSG00000117670	Gm50270	predicted gene,	lncRNA	13	8	2	44
ENSMUSG00000028064	Sema4a	sema domain, i	protein_coding	1,482	1,535	1,425	1,916
ENSMUSG00000106060	Gm42488	predicted gene	lncRNA	29	32	27	51
ENSMUSG00000031441	Atp11a	ATPase, class V	protein_coding	3,202	3,317	2,951	3,951
ENSMUSG00000038569	Rad9b	RAD9 checkpoi	protein_coding	281	292	248	380
ENSMUSG00000032580	Rbm5	RNA binding m	protein_coding	3,713	4,239	3,952	5,588
ENSMUSG00000075144	Or5l13	olfactory recep	protein_coding	51	53	46	96
ENSMUSG00000027751	Supt20	SPT20 SAGA co	protein_coding	984	1,104	1,090	1,403
ENSMUSG00000040407	Akap9	A kinase (PRKA)	protein_coding	5,477	5,774	5,715	6,885
ENSMUSG00000055341	Zfp457	zinc finger prot	protein_coding	70	71	83	164
ENSMUSG00000028649	Macf1	microtubule-ac	protein_coding	23,367	25,820	23,001	29,488
ENSMUSG00000083889	E530001F21Rik	RIKEN cDNA E5	processed_pseudoge	36	58	39	109
ENSMUSG00000113517	Gm47905	predicted gene,	TEC	23	60	44	85
ENSMUSG00000037965	Zc3h7a	zinc finger CCC	protein_coding	726	1,135	939	1,384

ENSMUSG00000042308	Setd1a	SET domain cor	protein_coding	1,761	2,178	2,006
ENSMUSG00000108884	Gm45792	predicted gene	lncRNA	70	120	102
ENSMUSG00000027676	Ccdc39	coiled-coil dom	protein_coding	658	826	666
ENSMUSG00000026131	Dst	dystonin	protein_coding	25,427	26,744	26,413
ENSMUSG00000114290	4930435F18Rik	RIKEN cDNA 49	lncRNA	33	51	42
ENSMUSG00000044519	Zfp488	zinc finger prot	protein_coding	481	550	545
ENSMUSG00000054702	Ap1s3	adaptor-relatec	protein_coding	311	444	373
ENSMUSG00000066263	Or51k1	olfactory recep	protein_coding	228	314	264
ENSMUSG00000059729	Or2y1	olfactory recep	protein_coding	141	166	164
ENSMUSG00000022961	Son	Son DNA bindin	protein_coding	10,461	11,557	10,778
ENSMUSG00000089719	Gm15758	predicted gene	lncRNA	16	28	23
ENSMUSG00000107524	Gm44136	predicted gene,	TEC	15	21	17
ENSMUSG00000115801	Gm55330	predicted gene,	lncRNA	1,935	2,089	1,909
ENSMUSG00000035266	Helq	helicase, POLQ-	protein_coding	250	294	222
ENSMUSG00000108617	Gm31749	predicted gene,	lncRNA	7	17	6
ENSMUSG00000104037	Gm37776	predicted gene,	TEC	29	32	20
ENSMUSG00000112549	Gm35818	predicted gene,	lncRNA	54	56	51
ENSMUSG00000043987	Cep164	centrosomal pr	protein_coding	536	670	512
ENSMUSG00000106841	Gm42656	predicted gene	TEC	24	28	24
ENSMUSG00000019971	Cep290	centrosomal pr	protein_coding	1,012	1,000	968
ENSMUSG00000028245	Nsmaf	neutral sphingo	protein_coding	1,083	1,242	1,068
ENSMUSG00000029168	Dpysl5	dihydropyrimid	protein_coding	2,027	2,085	1,890
ENSMUSG00000107021	Gm42853	predicted gene	TEC	29	35	21
ENSMUSG00000042156	Dzip1	DAZ interacting	protein_coding	3,587	4,012	3,819
ENSMUSG00000085527	Gm15535	predicted gene	lncRNA	25	52	56
ENSMUSG00000113427	Gm46378	predicted gene,	lncRNA	96	172	128
ENSMUSG00000074507	Gm14340	predicted gene	lncRNA	60	90	63
ENSMUSG00000061601	Pclo	piccolo (presyn	protein_coding	10,597	11,761	10,711
ENSMUSG00000097583	6430590A07Rik	RIKEN cDNA 64	lncRNA	102	130	94
ENSMUSG00000042508	Dmtf1	cyclin D binding	protein_coding	1,416	1,627	1,493
ENSMUSG00000111092	Gm17875	predicted gene,	processed_pseudoge	74	150	63
ENSMUSG00000063804	Lin28b	lin-28 homolog	protein_coding	281	363	253
ENSMUSG00000107535	Gm44183	predicted gene,	TEC	206	284	221
ENSMUSG00000059851	Kmt5c	lysine methyltr	protein_coding	322	374	295
ENSMUSG00000038056	Kmt2c	lysine (K)-speci	protein_coding	5,321	5,807	5,215
ENSMUSG00000111696	Gm47932	predicted gene,	processed_pseudoge	57	86	57
ENSMUSG00000107041	Gm42735	predicted gene	TEC	20	39	21
ENSMUSG00000084336	Or4x6	olfactory recep	protein_coding	17	49	18
ENSMUSG00000107771	Gm8956	predicted gene	processed_pseudoge	69	125	66
ENSMUSG00000037795	N4bp2	NEDD4 binding	protein_coding	1,212	1,406	1,174
ENSMUSG00000029206	Nsun7	NOL1/NOP2/Su	protein_coding	312	423	327
ENSMUSG00000049173	Myoz3	myozenin 3	protein_coding	1	6	2
ENSMUSG00000004110	Cacna1e	calcium channe	protein_coding	7,142	8,590	7,239
ENSMUSG00000061751	Kalrn	kalirin, RhoGEF	protein_coding	2,822	3,317	2,897
ENSMUSG00000024830	Rps6kb2	ribosomal prot	protein_coding	276	300	239
ENSMUSG00000057672	Pkn1	protein kinase	protein_coding	1,350	1,489	1,319
ENSMUSG00000095567	Noc2l	NOC2 like nucle	protein_coding	1,402	1,487	1,355
ENSMUSG00000022983	Scaf4	SR-related CTD-	protein_coding	1,099	1,130	887
ENSMUSG00000097797	Gm26901	predicted gene,	lncRNA	11	16	8
ENSMUSG00000103322	Gm37404	predicted gene,	TEC	52	62	36
ENSMUSG00000021514	Zfp369	zinc finger prot	protein_coding	811	863	770
ENSMUSG00000079487	Med12	mediator comp	protein_coding	793	1,000	757
ENSMUSG00000110693	Gm45899	predicted gene	lncRNA	129	184	162
ENSMUSG00000056763	Cspp1	centrosome an	protein_coding	780	934	757
ENSMUSG00000111390	Gm48796	predicted gene,	lncRNA	129	155	128
ENSMUSG00000053550	Shisa7	shisa family me	protein_coding	2,784	3,194	2,751
ENSMUSG00000027582	Zgpat	zinc finger, CCC	protein_coding	1,400	1,551	1,364
ENSMUSG00000104563	Gm43041	predicted gene	TEC	178	214	174
ENSMUSG00000035576	L3mbtl1	L3MBTL1 histor	protein_coding	209	326	287
ENSMUSG00000102659	Gm37077	predicted gene,	TEC	21	38	33
ENSMUSG00000072770	Acrbp	proacrosin bind	protein_coding	79	88	84

ENSMUSG00000112149	Gm47611	predicted gene	processed_pseudoge	287	350	243	514	422	478	400	456	570	525	571	502	1.26	0.0403
ENSMUSG00000104871	Gm42639	predicted gene	TEC	5	15	0	28	20	30	18	18	47	43	33	53	1.73	0.0913
ENSMUSG00000116283	Gm49412	predicted gene	TEC	48	55	33	75	62	63	62	57	83	79	67	83	1.14	0.36
ENSMUSG00000110390	Gm45869	predicted gene	lncRNA	17	32	9	35	29	26	33	37	49	41	46	56	1.35	0.195
ENSMUSG00000113978	Gm48260	predicted gene	processed_pseudoge	64	103	42	129	106	114	105	105	171	129	151	172	1.27	0.13
ENSMUSG00000112117	Rmst	rhabdomyosarc	lncRNA	347	441	273	393	464	519	378	507	610	538	466	560	1.29	0.00433
ENSMUSG00000036916	Zfp280c	zinc finger prot	protein_coding	559	701	505	695	708	766	677	724	875	836	802	817	1.17	0.0169
ENSMUSG00000078485	Plekhn1	pleckstrin hom	protein_coding	1,053	1,201	989	1,176	1,203	1,306	1,159	1,269	1,326	1,293	1,310	1,280	1.12	0.0448
ENSMUSG00000054412	Gm4793	predicted gene	lncRNA	27	45	23	63	54	64	58	68	87	83	62	74	1.55	0.00726
ENSMUSG00000106714	Gm42546	predicted gene	TEC	166	181	129	243	225	241	234	257	277	276	229	263	1.33	0.00993
ENSMUSG00000105270	Gm42863	predicted gene	TEC	158	208	132	272	257	286	273	281	320	307	300	289	1.42	0.00302
ENSMUSG00000112689	Gm47476	predicted gene	processed_pseudoge	194	263	140	376	363	405	345	359	454	443	446	452	1.51	0.00198
ENSMUSG00000067949	Vmn1r-ps144	vomeronasal 1	transcribed_unproce	167	216	151	252	243	292	243	276	286	302	265	267	1.34	0.0048
ENSMUSG00000107182	Gm43268	predicted gene	TEC	223	294	174	345	317	388	331	388	413	418	393	405	1.37	0.0022
ENSMUSG00000107996	Gm44228	predicted gene	TEC	8	18	0	35	34	33	22	29	45	55	39	38	1.9	0.0387
ENSMUSG00000090211	Gm16050	predicted gene	lncRNA	7	10	2	13	13	20	10	14	19	21	18	20	1.77	0.0762
ENSMUSG00000111358	2900078I11Rik	RIKEN cDNA 29	TEC	5	7	0	16	13	17	8	16	24	24	23	19	1.85	0.0688
ENSMUSG00000041415	Dicer1	dicer 1, ribonuc	protein_coding	2,607	2,709	2,339	2,785	2,829	2,988	2,799	2,869	3,001	3,235	2,942	3,085	1.1	0.0313
ENSMUSG00000038816	Ctnnal1	catenin (cadher	protein_coding	469	540	411	623	655	766	637	691	800	851	760	766	1.35	0.000446
ENSMUSG00000112409	C030006N10Rik	RIKEN cDNA C0	TEC	1	4	0	7	9	16	5	11	20	21	18	21	3.29	0.017
ENSMUSG00000038733	Wdr26	WD repeat dom	protein_coding	4,257	3,967	3,716	4,132	4,309	4,592	3,855	4,092	4,534	4,562	4,452	4,303	1.05	0.19
ENSMUSG00000086796	4932702P03Rik	RIKEN cDNA 49	lncRNA	60	58	43	56	73	84	63	57	85	85	82	76	1.26	0.0849
ENSMUSG00000110717	1700047G07Rik	RIKEN cDNA 17	lncRNA	12	7	5	9	16	21	14	10	19	23	22	19	1.82	0.0418
ENSMUSG00000089798	1700028K03Rik	RIKEN cDNA 17	protein_coding	106	116	35	73	158	101	67	82	165	134	156	164	1.24	0.284
ENSMUSG00000026447	Pik3c2b	phosphatidylin	protein_coding	939	909	791	890	1,019	1,055	917	895	1,031	1,009	1,128	1,173	1.1	0.0963
ENSMUSG00000042423	Fbrs	fibrosin	protein_coding	1,600	1,728	1,521	1,729	2,018	1,902	1,689	1,690	2,047	1,994	2,033	2,130	1.11	0.0522
ENSMUSG00000086602	Gm15609	predicted gene	lncRNA	60	75	56	72	83	88	72	64	98	92	103	94	1.16	0.262
ENSMUSG00000029420	Rimbp2	RIMS binding p	protein_coding	2,775	2,928	2,429	3,029	3,406	3,253	3,011	2,993	3,486	3,038	3,415	3,281	1.13	0.00797
ENSMUSG00000115690	Gm48924	predicted gene	lncRNA	44	41	23	40	65	50	51	52	65	55	55	62	1.47	0.0148
ENSMUSG00000020516	Rps6kb1	ribosomal prot	protein_coding	1,226	1,123	1,028	1,398	1,408	1,413	1,305	1,314	1,542	1,331	1,419	1,415	1.14	0.0257
ENSMUSG00000022429	Dmc1	DNA meiotic re	protein_coding	28	19	13	42	45	41	33	34	51	37	53	41	1.53	0.0285
ENSMUSG00000104432	A430027C01Rik	RIKEN cDNA A4	TEC	24	21	15	39	48	39	28	37	54	50	42	57	1.53	0.0476
ENSMUSG00000115955	9530056E24Rik	RIKEN cDNA 95	TEC	5	3	0	9	14	13	5	12	18	11	11	21	2.48	0.0228
ENSMUSG00000042672	Dcst1	DC-STAMP dom	protein_coding	88	102	75	101	121	140	105	95	128	122	114	137	1.25	0.0303
ENSMUSG00000104433	Gm37426	predicted gene	TEC	7	6	5	12	20	20	10	9	23	16	15	19	2.02	0.0396
ENSMUSG00000026213	Stk11ip	serine/threonin	protein_coding	465	467	459	553	582	599	508	548	722	595	637	692	1.15	0.0645
ENSMUSG00000106855	5330437M03Rik	RIKEN cDNA 53	TEC	5	11	3	20	24	24	9	12	42	30	29	43	1.7	0.0935
ENSMUSG00000113941	Gm35755	predicted gene	processed_pseudoge	8	14	8	32	34	39	15	23	61	62	41	66	1.8	0.063
ENSMUSG00000021725	Parp8	poly (ADP-ribos	protein_coding	1,003	994	975	1,080	1,163	1,091	1,075	1,018	1,217	1,185	1,144	1,233	1.07	0.114
ENSMUSG00000110110	Gm30978	predicted gene	lncRNA	24	24	15	32	36	29	24	22	48	35	32	44	1.16	0.436
ENSMUSG00000112972	Gm48417	predicted gene	processed_pseudoge	85	67	51	151	170	162	94	81	207	169	208	78	1.42	0.113
ENSMUSG00000024949	Sf1	splicing factor 1	protein_coding	4,524	4,376	4,216	4,741	4,711	4,961	4,684	4,688	5,066	4,804	5,543	4,741	1.07	0.134
ENSMUSG00000116590	Gm53028	predicted gene	lncRNA	19	20	15	40	41	41	31	33	54	35	60	37	1.54	0.0363
ENSMUSG00000085328	Gm17131	predicted gene	unprocessed_pseudo	1,250	1,203	1,211	1,643	1,691	1,765	1,371	1,529	1,846	1,675	1,983	1,731	1.2	0.0108
ENSMUSG00000099608	4933411E06Rik	RIKEN cDNA 49	lncRNA	35	27	35	61	65	79	50	48	76	64	90	65	1.53	0.0319
ENSMUSG00000103821	D430013B06Rik	RIKEN cDNA D4	TEC	27	22	17	52	47	47	33	36	57	52	60	60	1.37	0.0732
ENSMUSG00000028399	Ptprd	protein tyrosin	protein_coding	5,987	6,022	5,727	7,069	6,407	6,996	6,396	6,374	7,272	7,134	7,390	7,267	1.05	0.233
ENSMUSG00000112013	Gm47967	predicted gene	processed_pseudoge	56	44	47	109	82	110	64	86	122	119	143	138	1.33	0.0908
ENSMUSG00000085246	Gm15893	predicted gene	lncRNA	70	60	59	109	78	90	77	93	122	134	125	111	1.13	0.418
ENSMUSG00000112000	Gm47956	predicted gene	lncRNA	84	67	59	113	89	110	78	97	127	128	112	130	1.15	0.219
ENSMUSG00000042104	Uggt2	UDP-glucose gl	protein_coding	1,041	1,041	973	1,725	1,346	1,698	1,265	1,625	1,700	1,580	1,559	1,744	1.24	0.0283
ENSMUSG00000078902	Gm14443	predicted gene	protein_coding	153	139	120	250	197	248	166	217	239	208	249	230	1.25	0.0414
ENSMUSG00000113395	Gm48365	predicted gene	processed_pseudoge	299	287	234	566	487	521	395	502	548	489	519	502	1.37	0.0196
ENSMUSG00000090386	Mir99ahg	Mir99a and Mir	lncRNA	184	114	107	205	167	205	160	207	223	200	212	227	1.22	0.11
ENSMUSG00000114228	Gm47605	predicted gene	processed_pseudoge	8	1	0	24	4	29	10	23	25	21	41	24	1.96	0.115
ENSMUSG00000054309	Cpsf3	cleavage and p	protein_coding	1,331	1,373	1,245	1,475	1,462	1,686	1,443	1,475	1,651	1,515	1,565	1,637	1.12	0.0263
ENSMUSG00000113523	Gm48366	predicted gene	processed_pseudoge	65	66	39	99	96	170	81	111	151	128	116	137	1.69	0.000805
ENSMUSG00000060149	BC002059	cDNA sequence	lncRNA	235	217	171	265	277	315	237	267	307	263	324	310	1.23	0.0549
ENSMUSG00000073907	Or56b35	olfactory recep	protein_coding	3	4	0	8	6	21	4	7	19	9	18	16	2.13	0.0876
ENSMUSG00000103070	Gm37903	predicted gene	TEC	226	242	198	281	294	318	258	293	331	321	334	328	1.23	0.00548
ENSMUSG00000105207	Gm42927	predicted gene	processed_pseudoge	332	443	204	1,004	968	1,458	723	1,049	1,464	1,184	1,653	1,530	2.12	0.00504
ENSMUSG00000086231	Rapgef4os3	Rap guanine nu	lncRNA	32	30	14	59	58	84	47	61	83	79	98	81	1.86	0.00991
ENSMUSG00000112788	Gm47821	predicted gene	TEC	336	368	228	555	561	667	427	509	637	589	716	615	1.45	0.00284
ENSMUSG00000106446	Gm42970	predicted gene	TEC	9	21	6	31	29	54	22	35	47	44	61	55	2.07	0.00757
ENSMUSG00000109082	Gm44586	predicted gene	TEC	3	8	0	21	21	52	17	22	51	55	61	79	3.42	0.00231
ENSMUSG00000024943	Smc5	structural main	protein_coding	1,065	1,182	900	1,273	1,207	1,422	1,204	1,410	1,493	1,320	1,438	1,407	1.19	0.00702
ENSMUSG00000109044	Gm44680	predicted gene	TEC	1	4	0	11	11	13	4	9	21	10	16	16	2.27	0.0616
ENSMUSG00000117238		novel transcript	lncRNA	212	267	146	334	348	348	282	341	414	364	390	413	1.37	0.00791
ENSMUSG00000025558	Dock9	dedicator of cy	protein_coding	3,595	3,621	3,419	4,050	3,855	4,377	3,622	3,925	4,296	4,140	4,554	4,402	1.07	0.146
ENSMUSG00000061665	Cd2ap	CD2-associated	protein_coding	1,378	1,333	1,338	1,560	1,536	1,710	1,433	1,489	1,621	1,584	1,668	1,692	1.1	0.0736
ENSMUSG00000020280	Pus10	pseudouridylat	protein_coding	228	275	199	324	304	431	256	326	357	354	408	353	1.28	0.0324
ENSMUSG00000027692	Tnik	TRAF2 and NCK	protein_coding	5,193	5,643	5,359	6,630	6,475	7,208	5,455	6,278	6,888	7,043	6,962	6,909	1.11	0.0304
ENSMUSG00000031502	Col4a1	collagen, type I	protein_coding	1,002	1,123	991	1,529	1,505	1,830	1,159	1,367	1,604	1,574	1,775	1,804	1.26	0.0356
ENSMUSG00000117183	Gm20008	predicted gene	processed_pseudoge	236	264	213	331	327	375	252	289	334	337	388	362	1.19	0.0983
ENSMUSG00000112596	Gm48804	predicted gene	TEC	32	48	33	67	57	81	44	76	88	76	74	71	1.44	0.0406
ENSMUSG00000034269	Setd5	SET domain cor	protein_coding	3,888	4,119	3,733	4,247	4,211	4,409	3,929	4,347	4,510	4,278	4,424	4,399	1.06	0.0838
ENSMUSG00000057378	Ryr3	ryanodine rece	protein_coding	1,327	1,905	1,279	2,243	2,092	2,861	1,604	2,240	2,760	2,362	2,550	2,598	1.3	0.0576
ENSMUSG00000026141	Col19a1	collagen, type X	protein_coding	183	255	191	366	311	477	173	328	456	382	381	352	1.29	0.115
ENSMUSG00000110393	Gm36445	predicted gene	lncRNA	53	52	45	85	63	93	50	74	101	81	92	84	1.19	0.222
ENSMUSG00000019518	Ap4m1	adaptor-relate	protein_coding	448	444	441	409	428	510	540	478	529	530	482	506	1.12	0.0478
ENSMUSG00000085311	Gm15445	predicted gene	lncRNA	9	17	6	7	11	17	26	15	22	24	19	25	1.76	0.0473
ENSMUSG00000029727	Cyp3a13	cytochrome P4	protein_coding	11	15	9	17	18	31	24	21	30	36	14	27	1.79	0.0242
ENSMUSG00000032593	Amigo3	adhesion mole	protein_coding	41	48	33	39	48	67	59	57	77	65	54	57	1.43	0.0267
ENSMUSG00000100833	Gm28988	predicted gene	lncRNA	9	13	9	17	15	20	26	17	31	30	21	21	1.58	0.082
ENSMUSG00000112352	Gm40617	predicted gene	lncRNA	5	7	5	12	11	16	19	16	20	24	16	14	2.13	0.0137
ENSMUSG00000092274	Neat1	nuclear parasp	lncRNA	3,769	3,881	3,404	4,127	4,136	4,412	4,795	4,223	4,815	4,720	4,323	4,949	1.16	0.0537
ENSMUSG00000089706	B230216N24Rik	RIKEN cDNA B2	lncRNA	133	168	108	192	155	206	201	201	247	235	198	230	1.27	0.0544
ENSMUSG00000082705	Gm15616	predicted gene	processed_pseudoge	1	6	2	7	4	12	12	10	13	15	11	15	2.45	0.0381
ENSMUSG00000087644	Gm14703	predicted gene	lncRNA	54	63	48	75	58	83	95	76	88	98	77	99	1.29	0.0613
ENSMUSG00000028344	Invs	inversin	protein_coding	415	467	391	483	496	623	529	575	657	606	575	533	1.27	0.000693
ENSMUSG00000036893	Ehmt1	euchromatic hi	protein_coding	836	919	813	925	952	1,062	987	1,074	1,130	1,075	1,038	1,050	1.17	0.00806
ENSMUSG00000046169	Adamts6	a disintegrin-lik	protein_coding	239	291	233	293	295	360	292	356	414	399	383	324	1.23	0.0226
ENSMUSG00000086735	Gm13977	predicted gene	lncRNA	27	51	24	55	62	75	60	73	100	91	85	72	1.72	0.00121
ENSMUSG00000102443	Gm37102	predicted gene	processed_pseudoge	37	65	51	84	89	113	79	93	137	108	110	95	1.58	0.00226
ENSMUSG00000104641	Gm43290	predicted gene	TEC	8	24	17	29	31	42	28	43	49	42	50	35	1.87	0.00742
ENSMUSG00000067276	Capn6	calpain 6	protein_coding	48	71	62	80	85	128	79	116	136	133	131	111	1.57	0.00125
ENSMUSG00000105286	Gm43140	predicted gene	lncRNA	1	3	2	7	6	12	5	9	12	14	10	10	2.62	0.0272
ENSMUSG00000018068	Ints2	integrator com	protein_coding	509	689	595	624	690	789	686	899	805	844	839	764	1.27	0.00158
ENSMUSG00000115814	Gm49181	predicted gene	unprocessed_pseudo	4	15	11	16	16	22	15	32	27	34	24	16	1.89	0.0321
ENSMUSG00000082286	Pisd-ps1	phosphatidylse	transcribed_unproce	88	115	97	131	118	136	135	149	140	155	162	135	1.25	0.0237
ENSMUSG00000105322	Gm43751	predicted gene	TEC	3	11	8	12	9	16	14	20	20	24	24	18	1.8	0.0655
ENSMUSG00000106251	Gm42658	predicted gene	lncRNA	9	14	12	23	16	26	22	34	41	39	32	28	1.7	0.0289
ENSMUSG00000085269	Gm15777	predicted gene	lncRNA	16	31	24	36	34	43	31	50	48	49	51	46	1.5	0.0206
ENSMUSG00000055670	Zzef1	zinc finger, ZZ-t	protein_coding	2,036	2,527	2,207	2,546	2,499	2,707	2,634	2,816	3,043	3,024	2,822	2,835	1.14	0.0162
ENSMUSG00000024186	Rgs11	regulator of G-	protein_coding	307	429	334	512	443	469	418	581	627	628	554	547	1.21	0.11
ENSMUSG00000036391	Sec24a	Sec24 related g	protein_coding	752	870	788	943	877	933	890	968	1,005	1,007	990	975	1.09	0.0419
ENSMUSG00000020620	Abca8b	ATP-binding ca	protein_coding	804	915	890	895	930	962	964	997	1,108	1,011	1,048	1,059	1.1	0.0169
ENSMUSG00000087623	Gm12404	predicted gene	lncRNA	21	38	33	41	43	48	53	66	60	56	65	59	1.59	0.00351
ENSMUSG00000105592	Gm42527	predicted gene	TEC	3	6	6	8	9	10	12	19	19	12	19	13	2.28	0.0138
ENSMUSG00000060503	Or2d2	olfactory recep	protein_coding	24	53	38	47	52	58	63	61	82	58	54	57	1.44	0.023
ENSMUSG00000097267	1700109K24Rik	RIKEN cDNA 17	lncRNA	21	38	36	45	39	58	46	57	68	64	50	47	1.43	0.0364
ENSMUSG00000109087	Gm45105	predicted gene	processed_pseudoge	21	39	36	44	44	62	47	60	82	52	51	59	1.52	0.0213
ENSMUSG00000104211	Gm37985	predicted gene	TEC	33	87	39	47	62	59	62	80	89	78	75	85	1.28	0.086
ENSMUSG00000109775	Gm45297	predicted gene	TEC	9	21	9	16	19	20	22	30	44	21	27	29	1.64	0.0767
ENSMUSG00000115756	Gm49519	predicted gene	lncRNA	70	116	86	88	97	112	118	118	123	112	114	159	1.25	0.0573
ENSMUSG00000026458	Ppfia4	protein tyrosin	protein_coding	2,868	3,094	2,786	2,986	3,054	2,972	3,081	3,147	3,200	3,266	3,142	3,362	1.04	0.136
ENSMUSG00000091345	Col6a5	collagen, type V	protein_coding	170	280	139	204	216	266	227	300	342	339	291	414	1.28	0.0989
ENSMUSG00000111758	Gm30373	predicted gene	lncRNA	21	92	38	51	67	89	66	76	83	84	88	79	1.48	0.0188
ENSMUSG00000029552	Tes	testin LIM dom	protein_coding	27	64	38	48	53	68	53	68	86	77	76	67	1.42	0.0622
ENSMUSG00000114623	Gm34788	predicted gene	lncRNA	9	36	19	21	33	39	32	44	46	53	46	37	1.78	0.00373
ENSMUSG00000066440	Zfyve26	zinc finger, FYV	protein_coding	554	848	622	652	824	870	914	833	879	1,026	913	873	1.29	0.00046
ENSMUSG00000023473	Celsr3	cadherin, EGF L	protein_coding	2,004	3,218	2,484	2,916	3,397	3,859	3,770	3,472	3,603	3,998	3,944	4,041	1.37	0.000217
ENSMUSG00000058979	Hdhd5	haloacid dehal	protein_coding	431	627	468	568	603	691	635	684	658	726	659	708	1.25	0.00124
ENSMUSG00000022948	Setd4	SET domain cor	protein_coding	108	185	119	162	154	170	197	189	215	207	201	204	1.24	0.0654
ENSMUSG00000041954	Tnfrsf18	tumor necrosis	protein_coding	82	145	77	129	132	177	161	168	187	160	179	186	1.48	0.000617
ENSMUSG00000115088	Gm49165	predicted gene	lncRNA	58	93	56	88	99	127	120	112	125	127	123	117	1.55	0.000146
ENSMUSG00000032558	Nphp3	nephronophthi	protein_coding	276	508	393	427	463	569	500	614	504	766	445	635	1.34	0.0182
ENSMUSG00000025377	Tepsin	TEPSIN, adapto	protein_coding	244	314	242	286	273	303	315	319	305	369	339	337	1.12	0.175
ENSMUSG00000118340	Gm50301	predicted gene	lncRNA	5	7	2	9	5	16	13	20	16	30	21	17	2.3	0.0136
ENSMUSG00000024561	Mbd1	methyl-CpG bin	protein_coding	1,143	1,239	1,192	1,171	1,275	1,332	1,277	1,185	1,300	1,297	1,311	1,321	1.07	0.0524
ENSMUSG00000049041	Or1r1	olfactory recep	protein_coding	15	35	23	27	41	47	38	22	54	52	49	43	1.49	0.0388
ENSMUSG00000002109	Ddb2	damage specifi	protein_coding	189	236	234	236	263	280	236	208	254	299	289	272	1.1	0.228
ENSMUSG00000102413	Gm36937	predicted gene	lncRNA	4	13	9	15	16	24	19	7	20	32	23	19	1.61	0.0965
ENSMUSG00000105868	Gm43766	predicted gene	TEC	81	98	71	72	93	123	110	78	97	135	108	122	1.25	0.09
ENSMUSG00000022568	Scrib	scribbled plana	protein_coding	1,262	1,300	1,222	1,254	1,509	1,385	1,308	1,224	1,411	1,574	1,464	1,536	1.07	0.12
ENSMUSG00000036026	Tmem63b	transmembran	protein_coding	2,777	2,845	2,641	2,737	2,867	2,953	2,694	2,752	2,871	3,139	2,921	3,108	1.02	0.468
ENSMUSG00000027346	Gpcpd1	glycerophosph	protein_coding	1,427	1,497	1,492	1,479	1,545	1,711	1,420	1,531	1,851	1,760	1,484	1,665	1.05	0.258
ENSMUSG00000053441	Adamts19	a disintegrin-lik	protein_coding	30	49	35	50	44	65	32	39	73	87	47	68	1.1	0.612
ENSMUSG00000063652	Slc22a21	solute carrier f	protein_coding	65	89	91	96	103	170	97	114	143	140	103	125	1.41	0.0188
ENSMUSG00000005682	Pan2	PAN2 poly(A) s	protein_coding	388	518	425	592	600	668	495	554	626	642	630	604	1.2	0.0218
ENSMUSG00000074340	Ovgp1	oviductal glyco	protein_coding	85	164	129	192	182	236	140	176	230	231	204	206	1.29	0.0876
ENSMUSG00000065968	Ifitm7	interferon indu	protein_coding	7	27	14	36	37	61	26	45	45	56	46	40	2.03	0.00212
ENSMUSG00000027710	Acad9	acyl-Coenzyme	protein_coding	1,068	1,251	1,198	1,385	1,380	1,623	1,310	1,410	1,540	1,531	1,466	1,483	1.17	0.00904
ENSMUSG00000105095	8430422M14Rik	RIKEN cDNA 84	TEC	27	48	38	60	63	85	58	63	74	73	76	69	1.56	0.00564
ENSMUSG00000039307	Hexdc	hexosaminidas	protein_coding	653	934	847	942	980	1,127	893	990	1,122	1,148	949	989	1.18	0.0221
ENSMUSG00000031328	Flna	filamin, alpha	protein_coding	1,496	2,071	1,713	1,966	2,265	2,768	1,868	2,297	2,859	2,744	2,434	2,463	1.27	0.0723
ENSMUSG00000106924	Gm42857	predicted gene	processed_pseudoge	304	463	340	490	507	590	407	547	655	625	558	523	1.29	0.0227
ENSMUSG00000016493	Cd46	CD46 antigen, c	protein_coding	187	263	175	320	355	393	255	342	404	430	357	438	1.42	0.00789
ENSMUSG00000032308	Ulk3	unc-51-like kin	protein_coding	295	369	313	379	424	478	354	425	464	473	419	468	1.24	0.00604
ENSMUSG00000104149	Gm37138	predicted gene	TEC	39	73	56	81	79	98	69	101	94	112	89	109	1.4	0.0149
ENSMUSG00000112998	Gm47656	predicted gene	TEC	45	84	48	79	84	101	72	96	100	118	95	130	1.38	0.0397
ENSMUSG00000110080	Gm6145	predicted gene	lncRNA	636	734	634	756	738	881	721	759	824	925	827	881	1.12	0.0642
ENSMUSG00000079710	Dynlt2a2	dynein light ch	protein_coding	67	76	69	89	94	124	97	103	110	130	115	122	1.39	0.0106
ENSMUSG00000086753	Gm15751	predicted gene	lncRNA	48	55	48	64	68	88	69	70	82	101	81	85	1.37	0.016
ENSMUSG00000038011	Dnah10	dynein, axonem	protein_coding	207	209	215	272	281	537	268	358	349	426	379	429	1.59	0.00267
ENSMUSG00000115261	Gm35769	predicted gene	lncRNA	13	15	17	23	31	39	17	27	30	34	30	38	1.68	0.0149
ENSMUSG00000026726	Cubn	cubilin (intrinsi	protein_coding	61	70	72	68	69	102	83	89	107	98	119	80	1.27	0.0898
ENSMUSG00000047642	D930020B18Rik	RIKEN cDNA D9	protein_coding	41	52	40	35	39	80	52	50	70	64	77	53	1.31	0.0959
ENSMUSG00000096351	Samd11	sterile alpha m	protein_coding	117	164	121	149	140	199	142	151	223	216	189	153	1.14	0.244
ENSMUSG00000113459	Gm30655	predicted gene	lncRNA	20	37	17	36	29	48	23	36	56	56	57	40	1.23	0.277
ENSMUSG00000001062	Vps9d1	VPS9 domain c	protein_coding	621	634	649	683	714	885	636	784	785	874	858	747	1.17	0.0116
ENSMUSG00000030400	Ercc2	excision repair	protein_coding	514	637	512	605	599	780	561	711	685	788	722	658	1.17	0.0228
ENSMUSG00000102618	Gm37848	predicted gene	TEC	4	8	3	3	8	16	6	12	12	10	14	15	2.32	0.0299
ENSMUSG00000106204	Gm21009	predicted gene	processed_pseudoge	1	3	0	0	4	13	3	8	9	10	10	12	6.71	0.00495
ENSMUSG00000106150	4921527H02Rik	RIKEN cDNA 49	TEC	12	15	6	9	10	25	13	27	24	21	22	27	1.77	0.0431
ENSMUSG00000114536	Gm48837	predicted gene	lncRNA	25	41	24	24	26	62	33	58	49	61	58	65	1.58	0.02
ENSMUSG00000023009	Nckap5l	NCK-associated	protein_coding	473	495	450	505	500	660	513	522	572	585	615	591	1.14	0.0365
ENSMUSG00000030091	Nup210	nucleoporin 21	protein_coding	695	893	693	797	805	1,218	915	919	1,067	1,122	1,069	971	1.25	0.0219
ENSMUSG00000102579	Gm37965	predicted gene	TEC	0	3	0	1	3	20	3	7	10	11	10	21	7.67	0.00288
ENSMUSG00000105990	Gm43307	predicted gene	TEC	45	80	54	75	75	132	74	102	117	103	108	116	1.51	0.0102
ENSMUSG00000034621	Gpatch8	G patch domair	protein_coding	2,130	2,354	2,138	2,226	2,483	2,961	2,412	2,647	2,555	2,617	2,913	2,775	1.19	0.00138
ENSMUSG00000087014	Gm16364	predicted gene	lncRNA	21	28	24	27	31	47	29	34	39	39	44	41	1.41	0.0578
ENSMUSG00000086889	Phf2os1	PHD finger prot	lncRNA	13	21	11	23	35	45	19	24	44	29	40	44	1.8	0.013
ENSMUSG00000044037	Als2cl	ALS2 C-termina	protein_coding	335	426	327	411	526	655	496	510	571	542	564	657	1.46	0.000768
ENSMUSG00000106965	Gm18290	predicted gene	processed_pseudoge	11	18	11	16	26	39	20	29	39	32	38	40	2.07	0.00421
ENSMUSG00000108701	Gm44616	predicted gene	TEC	15	27	12	19	25	45	22	24	44	31	32	33	1.59	0.0386
ENSMUSG00000045275	Lca5l	Leber congenit	protein_coding	128	189	133	158	177	213	169	184	223	185	205	219	1.22	0.037
ENSMUSG00000100937	Nscme3l	NSE3 homolog,	protein_coding	15	37	14	16	34	46	27	34	43	38	49	49	1.74	0.0157
ENSMUSG00000021494	Ddx41	DEAD box helic	protein_coding	479	528	452	500	571	569	500	526	548	551	668	548	1.1	0.0862
ENSMUSG00000109679	Gm45342	predicted gene	TEC	12	20	9	15	30	29	14	23	29	27	41	21	1.73	0.0293
ENSMUSG00000036864	Proser3	proline and seri	protein_coding	87	149	117	113	134	172	138	148	150	156	221	130	1.28	0.0494
ENSMUSG00000073973	Or52k2	olfactory recep	protein_coding	66	93	90	76	133	136	104	93	117	124	166	119	1.43	0.00568
ENSMUSG00000100868	Gm28095	predicted gene	lncRNA	0	4	5	1	10	12	3	6	7	8	15	10	3.03	0.0311
ENSMUSG00000102504	Gm21955	predicted gene	TEC	301	447	326	444	417	403	356	473	433	483	616	465	1.09	0.367
ENSMUSG00000051331	Cacna1c	calcium channe	protein_coding	2,206	2,562	2,343	2,531	2,501	2,773	2,527	2,718	2,636	2,650	3,040	2,689	1.09	0.0509
ENSMUSG00000026199	Ankzf1	ankyrin repeat	protein_coding	404	616	460	533	568	627	504	627	581	606	719	631	1.16	0.0839
ENSMUSG00000113921	Gm48054	predicted gene	processed_pseudoge	57	139	74	152	151	193	101	182	149	176	250	200	1.48	0.0413
ENSMUSG00000037363	Letm2	leucine zipper-	protein_coding	502	576	508	658	700	645	510	674	591	755	944	784	1.13	0.276
ENSMUSG00000011751	Sptbn4	spectrin beta, n	protein_coding	3,787	4,039	3,678	4,196	4,399	4,555	3,814	4,480	4,143	4,318	5,079	4,729	1.1	0.0558
ENSMUSG00000022228	Zscan26	zinc finger and	protein_coding	3,529	3,554	3,382	3,748	3,765	3,874	3,497	3,869	3,717	3,768	4,185	3,984	1.06	0.0711
ENSMUSG00000028197	Col24a1	collagen, type X	protein_coding	49	66	71	80	91	135	73	104	113	98	159	138	1.52	0.00813
ENSMUSG00000104348	Gm37691	predicted gene	TEC	41	55	50	72	63	86	56	75	85	64	107	92	1.29	0.0821
ENSMUSG00000028693	Nasp	nuclear autoan	protein_coding	376	428	345	402	457	502	422	456	466	444	610	512	1.18	0.0138
ENSMUSG00000029701	Rbm28	RNA binding m	protein_coding	1,102	1,117	1,051	1,187	1,282	1,347	1,278	1,309	1,262	1,222	1,679	1,417	1.17	0.00664
ENSMUSG00000033253	Szt2	SZT2 subunit of	protein_coding	1,108	1,660	1,193	1,357	1,502	1,488	1,439	1,561	1,757	1,853	1,853	1,757	1.13	0.172
ENSMUSG00000034579	Pla2g3	phospholipase	protein_coding	41	71	35	56	62	63	57	74	69	79	87	75	1.27	0.0918
ENSMUSG00000106087	Gm43609	predicted gene	TEC	12	22	9	21	24	24	19	25	33	33	42	29	1.42	0.129
ENSMUSG00000108227	4933412L11Rik	RIKEN cDNA 49	TEC	1	15	3	5	20	21	12	14	19	26	21	13	2.64	0.00769
ENSMUSG00000027284	Cdan1	congenital dyse	protein_coding	448	659	497	660	702	806	605	716	722	896	878	748	1.25	0.00951
ENSMUSG00000039233	Tbce	tubulin-specific	protein_coding	562	927	654	857	979	1,008	792	941	1,015	1,160	1,050	981	1.24	0.00823
ENSMUSG00000040938	Slc16a11	solute carrier f	protein_coding	375	500	394	447	501	543	485	512	540	628	559	552	1.19	0.0285
ENSMUSG00000104010	Gm37366	predicted gene	TEC	15	28	15	24	34	39	28	37	38	53	49	46	1.7	0.00783
ENSMUSG00000024764	Naa40	N(alpha)-acetyl	protein_coding	533	716	551	680	742	738	737	720	780	824	814	768	1.18	0.0327
ENSMUSG00000114134	Gm47408	predicted gene	lncRNA	180	318	175	269	335	410	311	343	397	468	436	353	1.48	0.00162
ENSMUSG00000084846	A730011C13Rik	RIKEN cDNA A7	lncRNA	34	42	27	44	50	46	33	55	58	58	56	57	1.26	0.166
ENSMUSG00000094365	Gm21982	predicted gene	lncRNA	0	7	0	8	16	10	0	22	24	41	28	10	3.28	0.0867
ENSMUSG00000109243	Gm45867	predicted gene	TEC	25	39	21	35	54	54	33	55	53	58	55	45	1.63	0.00281
ENSMUSG00000103046	Gm37309	predicted gene	TEC	31	42	33	39	54	54	37	54	57	55	64	52	1.38	0.0384
ENSMUSG00000107495	Gm44215	predicted gene	TEC	497	689	557	614	708	720	636	729	740	748	842	709	1.19	0.0172
ENSMUSG00000053137	Mapk11	mitogen-activa	protein_coding	472	575	486	538	609	575	480	583	563	605	637	620	1.09	0.141
ENSMUSG00000106767	Gm42727	predicted gene	TEC	9	32	21	28	36	34	23	30	41	41	50	38	1.36	0.146
ENSMUSG00000000600	Krit1	KRIT1, ankyrin r	protein_coding	2,048	2,348	1,887	2,433	2,444	2,512	2,393	2,416	2,421	2,415	2,722	2,564	1.12	0.02
ENSMUSG00000072572	Slc39a2	solute carrier f	protein_coding	79	149	72	164	160	217	163	154	182	175	285	199	1.49	0.0061
ENSMUSG00000113890	Gm48368	predicted gene	processed_pseudoge	892	1,185	816	1,238	1,238	1,395	1,233	1,292	1,378	1,356	1,633	1,430	1.25	0.0028
ENSMUSG00000001415	Smg5	SMG5 nonsens	protein_coding	2,066	2,209	1,933	2,255	2,317	2,305	2,234	2,346	2,297	2,199	2,484	2,387	1.09	0.0206
ENSMUSG00000044712	Slc38a6	solute carrier f	protein_coding	3,725	4,667	3,451	4,890	5,133	5,202	4,292	4,790	4,972	4,619	5,634	5,284	1.16	0.0301
ENSMUSG00000102307	Gm38194	predicted gene	TEC	935	1,274	864	1,567	1,775	1,728	1,336	1,616	1,587	1,536	2,136	1,904	1.39	0.00229
ENSMUSG00000107962	Gm43980	predicted gene	TEC	404	589	350	767	769	764	636	723	742	755	925	856	1.37	0.008
ENSMUSG00000023952	Gtpbp2	GTP binding pr	protein_coding	647	856	606	963	946	1,073	862	974	858	1,046	1,142	1,066	1.26	0.00408
ENSMUSG00000027709	Mccc1	methylcrotono	protein_coding	712	935	699	1,136	1,048	1,138	948	1,074	1,010	1,099	1,338	1,236	1.21	0.0474
ENSMUSG00000038658	Ric1	RAB6A GEF con	protein_coding	1,335	1,511	1,341	1,656	1,617	1,705	1,522	1,634	1,601	1,695	1,827	1,875	1.11	0.0307
ENSMUSG00000022842	Ece2	endothelin con	protein_coding	2,224	2,704	2,067	3,039	2,852	2,846	2,548	2,790	2,770	2,929	3,178	3,059	1.1	0.126
ENSMUSG00000030846	Tial1	Tia1 cytotoxic g	protein_coding	3,390	4,005	2,875	4,457	4,064	4,454	4,026	4,455	4,314	4,400	4,798	4,830	1.15	0.0277
ENSMUSG00000103839	Gm37607	predicted gene	TEC	259	287	209	329	375	424	315	403	393	391	506	434	1.4	0.000354
ENSMUSG00000020866	Cacna1g	calcium channe	protein_coding	2,506	2,823	2,269	3,349	3,395	3,689	3,150	3,744	3,545	3,832	4,236	4,023	1.28	0.00269
ENSMUSG00000062372	Otof	otoferlin	protein_coding	858	973	800	1,067	1,151	1,174	1,035	1,190	1,081	1,197	1,339	1,305	1.23	0.00741
ENSMUSG00000112038	Gm47056	predicted gene	processed_pseudoge	159	239	156	283	316	365	232	350	346	349	408	402	1.51	0.00114
ENSMUSG00000032621	Srek1	splicing regulat	protein_coding	1,923	2,441	1,776	2,759	2,848	3,226	2,420	3,042	3,111	2,936	3,268	3,324	1.3	0.00155
ENSMUSG00000102700	Gm38312	predicted gene	TEC	289	366	263	400	407	445	354	437	442	438	485	466	1.25	0.00678
ENSMUSG00000108064	Gm44423	predicted gene	TEC	25	41	15	51	52	72	40	59	55	55	72	66	1.68	0.0228
ENSMUSG00000024045	Akap8	A kinase (PRKA)	protein_coding	2,082	2,617	1,807	3,061	3,044	3,490	2,559	2,944	3,181	3,302	3,559	3,617	1.26	0.00807
ENSMUSG00000028248	Pnisr	PNN interacting	protein_coding	4,479	5,738	4,020	6,748	7,017	8,070	6,002	6,749	7,294	7,171	8,311	7,897	1.33	0.00124
ENSMUSG00000104245	Gm38155	predicted gene	lncRNA	28	59	26	55	72	76	58	75	76	71	91	78	1.67	0.00296
ENSMUSG00000111619	Gm48348	predicted gene	processed_pseudoge	177	247	173	261	302	329	297	340	344	300	412	332	1.48	0.000362
ENSMUSG00000021495	Fam193b	family with seq	protein_coding	1,633	2,047	1,668	2,151	2,143	2,330	2,172	2,358	2,278	2,361	2,750	2,541	1.2	0.00209
ENSMUSG00000107632	Gm44070	predicted gene	TEC	90	131	83	160	156	181	155	192	185	180	221	203	1.47	0.00187
ENSMUSG00000014355	Anapc1	anaphase prom	protein_coding	2,513	3,190	2,524	3,437	3,420	3,485	3,392	3,591	3,562	3,576	3,824	3,685	1.19	0.00376
ENSMUSG00000025574	Tk1	thymidine kinas	protein_coding	65	115	65	135	120	146	135	146	157	146	179	146	1.43	0.017
ENSMUSG00000034206	Polq	polymerase (DN	protein_coding	37	74	40	87	79	87	92	97	116	75	122	93	1.54	0.00776
ENSMUSG00000085913	Gm15601	predicted gene	lncRNA	46	66	37	86	73	91	94	106	110	97	133	92	1.55	0.0102

ENSMUSG00000114148	Gm47701	predicted gene,	lncRNA	56	77	42	83	103	118	103	99	116	104	141	155	1.63	0.00391
ENSMUSG00000106864	Gtf3c2	general transcri	protein_coding	964	1,291	930	1,275	1,356	1,339	1,322	1,338	1,488	1,403	1,638	1,641	1.2	0.011
ENSMUSG00000110077	Gm45632	predicted gene	TEC	24	45	15	45	60	59	47	67	72	67	74	87	1.81	0.00364
ENSMUSG00000109031	Gm45201	predicted gene	TEC	24	28	11	27	29	46	37	39	46	41	52	49	1.68	0.0146
ENSMUSG00000004565	Pnpla6	patatin-like phc	protein_coding	1,644	1,790	1,577	1,834	1,834	2,110	1,936	1,960	1,994	1,979	2,225	2,166	1.15	0.0201
ENSMUSG00000107796	Gm44068	predicted gene,	TEC	3	6	2	7	9	17	9	16	12	11	18	19	3.07	0.00263
ENSMUSG00000049676	Catsperg1	cation channel :	protein_coding	67	141	43	115	133	200	93	140	160	148	177	165	1.54	0.0189
ENSMUSG00000112319	Gm47221	predicted gene,	lncRNA	125	214	93	185	209	325	151	209	266	259	279	304	1.45	0.0302
ENSMUSG00000034656	Cacna1a	calcium channe	protein_coding	3,366	3,971	3,244	4,054	3,908	4,358	3,782	4,122	4,359	4,360	4,846	4,464	1.1	0.0744
ENSMUSG00000097536	2610037D02Rik	RIKEN cDNA 26	lncRNA	78	121	78	113	108	144	98	121	128	136	154	142	1.2	0.105
ENSMUSG00000033526	Ppip5k1	diphosphoinosi	protein_coding	3,482	3,811	3,154	3,697	3,525	3,862	3,487	3,572	3,980	3,883	4,035	3,942	1.02	0.595
ENSMUSG00000004896	Rrnad1	ribosomal RNA	protein_coding	679	776	608	839	754	815	741	751	822	829	954	859	1.05	0.384
ENSMUSG00000024290	Rock1	Rho-associated	protein_coding	1,433	1,564	1,153	1,630	1,593	1,725	1,540	1,614	1,705	1,697	1,911	1,731	1.12	0.0491
ENSMUSG00000027130	Slc12a6	solute carrier fa	protein_coding	3,872	4,220	3,629	4,283	4,131	4,362	4,008	4,315	4,421	4,449	4,524	4,363	1.05	0.174
ENSMUSG00000031503	Col4a2	collagen, type I'	protein_coding	1,417	2,349	1,532	2,486	2,554	2,937	1,864	2,357	2,739	2,813	2,786	3,223	1.25	0.0612
ENSMUSG00000037300	Ttc13	tetratricopeptic	protein_coding	764	949	794	1,141	1,128	1,252	912	1,062	1,121	1,135	1,262	1,291	1.19	0.0426
ENSMUSG00000024287	Thoc1	THO complex 1	protein_coding	788	1,059	852	1,296	1,234	1,425	1,019	1,146	1,272	1,365	1,482	1,409	1.21	0.0314
ENSMUSG00000028869	Gnl2	guanine nucleo	protein_coding	1,286	1,466	1,315	1,600	1,534	1,711	1,421	1,511	1,586	1,623	1,649	1,680	1.09	0.0795
ENSMUSG00000018986	Slfn3	schlafen 3	protein_coding	41	65	45	73	74	93	64	67	88	71	109	92	1.33	0.0391
ENSMUSG00000027966	Col11a1	collagen, type X	protein_coding	450	770	589	952	1,055	1,186	750	867	1,245	1,013	1,375	1,230	1.4	0.0425
ENSMUSG00000097545	Mir124a-1hg	Mir124-1 host g	lncRNA	1,117	1,543	1,122	1,647	1,790	1,930	1,515	1,659	1,842	1,767	2,086	2,050	1.27	0.00498
ENSMUSG00000113290	A530058O07Rik	RIKEN cDNA A5	TEC	37	77	45	84	87	113	64	78	103	95	122	126	1.4	0.0454
ENSMUSG00000105663	Gm43606	predicted gene	TEC	54	83	57	96	97	113	87	89	119	115	131	143	1.32	0.0495
ENSMUSG00000094840	Muc3a	mucin 3A, cell s	protein_coding	12	30	18	40	45	56	28	41	54	60	70	74	1.71	0.0133
ENSMUSG00000032637	Atxn2l	ataxin 2-like	protein_coding	3,407	4,118	3,553	4,458	4,521	4,800	4,134	4,526	4,790	4,786	5,192	5,156	1.16	0.00944

ENSMUSG00000110997	2610028D06Rik	RIKEN cDNA 26	TEC	7	21	9	32	28	43	18	22	49	34	34	45	1.59	0.0522			2.34	0.000188	0.00574	up	1.47	0.0619	1	3	1197	-1.86116428	-0.4034623	-1.55101432	0.35566931	0.07280023	1.00207517	-0.65989053	-0.30625826	1.27420631	0.47815229	0.50619809	1.09268827
ENSMUSG00000038371	Sbf2	SET binding fac	protein_coding	1,601	1,856	1,723	1,920	1,952	2,186	1,970	1,864	2,186	2,099	1,985	2,109	1.12	0.0193	0.18		1.18	0.000662	0.0128	up	1.05	0.293	1	3	1198	-2.07006242	-0.51068763	-1.29921679	-0.15079113	0.02557115	1.24335712	0.12431874	-0.46897111	1.24090163	0.80269612	0.20560618	0.85727813
ENSMUSG00000109505	Gm44677	predicted gene	TEC	7	13	5	29	23	45	22	22	49	40	26	44	2.07	0.026			2.97	0.000605	0.0121	up	1.44	0.215	1	3	1199	-1.50819499	-0.88217818	-1.79923829	0.29150227	-0.1223452	1.07075081	-0.17908546	-0.14079049	1.24961512	0.85108001	0.11407647	1.05480792
ENSMUSG00000029823	Luc7l2	LUC7-like 2 (S. (	protein_coding	5,093	6,131	5,223	7,214	6,864	7,573	6,511	6,726	7,736	7,661	6,936	7,689	1.17	0.00986	0.13		1.27	0.000084	0.00342	up	1.09	0.177	1	3	1200	-1.93196772	-0.64193115	-1.7567229	0.49525382	0.14718377	0.83652017	-0.22169451	0.00534565	0.98589857	0.91790643	0.22069754	0.94351033
ENSMUSG00000041488	Stx3	syntaxin 3	protein_coding	851	1,074	883	1,223	1,169	1,283	1,126	1,124	1,363	1,258	1,183	1,266	1.17	0.0267	0.206		1.26	0.000824	0.0148	up	1.08	0.263	1	3	1201	-2.01577351	-0.42667273	-1.76762043	0.48927107	0.16859863	0.82935948	-0.09695144	-0.10813747	1.25797975	0.68859951	0.25069441	0.73065272
ENSMUSG00000102729	Gm32444	predicted gene	TEC	76	239	102	365	377	491	345	363	531	476	386	431	2.01	0.00449	0.0921		2.33	0.000573	0.0118	up	1.16	0.548	1	3	1202	-2.13497408	-0.53063162	-1.78304397	0.24356089	0.30503339	0.83922099	0.13100236	0.23179803	1.00368402	0.77349024	0.35120813	0.56965161
ENSMUSG00000008200	Fnbp4	formin binding	protein_coding	1,149	1,476	1,105	1,772	1,653	2,067	1,698	1,762	1,970	1,956	1,853	1,954	1.31	0.00172	0.0636		1.41	0.0000563	0.00268	up	1.08	0.376	1	3	1203	-1.81623244	-0.6159405	-1.99735045	0.27896043	-0.0635748	1.04749274	0.06793315	0.25244517	0.80789648	0.77084466	0.50175084	0.76577472
ENSMUSG00000117434	1810014P07Rik	RIKEN cDNA 18	lncRNA	53	86	51	119	109	145	119	111	144	133	117	128	1.56	0.00125	0.0553		1.69	0.000109	0.00403	up	1.08	0.547	1	3	1204	-1.87461018	-0.64657686	-1.96313401	0.33901211	0.07875175	1.02222342	0.35179301	0.1205206	0.98387103	0.71603563	0.28029351	0.59181999
ENSMUSG00000049571	Cfap46	cilia and flagella	protein_coding	487	644	540	839	740	821	794	750	866	836	823	795	1.24	0.00387	0.0861		1.32	0.000115	0.00421	up	1.07	0.343	1	3	1205	-2.17935424	-0.72728918	-1.65301345	0.7267271	0.02942761	0.60651134	0.41675537	0.10655578	0.91038105	0.71120161	0.62367837	0.42841865
ENSMUSG00000104237	Gm33533	predicted gene	lncRNA	82	145	105	188	161	216	186	162	213	204	180	196	1.39	0.00687	0.11		1.52	0.000396	0.00937	up	1.1	0.416	1	3	1206	-2.24879162	-0.4930946	-1.53482206	0.45657944	-0.12067725	1.00058178	0.41111494	-0.09474235	0.9371206	0.7709293	0.29969464	0.61610717
ENSMUSG00000053889	Kirrel3os	kirre like nephr	lncRNA	5	11	5	17	14	17	21	17	26	18	18	22	1.75	0.0382			2.2	0.00212	0.0268	up	1.25	0.298	1	3	1207	-1.76283492	-0.60055282	-1.96494294	0.29153684	-0.19770849	0.25028385	0.68982466	0.21521859	1.3529162	0.43866323	0.40918334	0.87841244
ENSMUSG00000102602	A930004J17Rik	RIKEN cDNA A9	TEC	131	232	129	277	244	286	254	264	342	291	253	284	1.36	0.0226	0.192		1.52	0.00161	0.0225	up	1.12	0.389	1	3	1208	-1.96396638	-0.16652227	-2.01348707	0.47646045	0.01040029	0.58320147	0.15067442	0.28873164	1.27496078	0.65618604	0.14650045	0.5568602
ENSMUSG00000109394	A230057D06Rik	RIKEN cDNA A2	lncRNA	428	883	433	1,070	977	1,158	1,030	1,114	1,411	1,246	1,080	1,190	1.52	0.0102	0.132		1.75	0.000575	0.0118	up	1.15	0.383	1	3	1209	-2.03484863	-0.21751899	-2.00793911	0.30463364	0.05587004	0.52513699	0.20143716	0.41733012	1.08735538	0.73302415	0.33255707	0.60296218
ENSMUSG00000113476	Gm48309	predicted gene	TEC	159	337	162	415	387	436	391	425	541	472	470	457	1.53	0.0342	0.23		1.81	0.00292	0.0328	up	1.18	0.392	1	3	1210	-2.03703667	-0.26512762	-1.9990443	0.29760883	0.10676499	0.4395114	0.13205697	0.36424074	1.06813319	0.66701576	0.65509904	0.57077766
ENSMUSG00000051965	Nanos2	nanos C2HC-typ	protein_coding	0	4	3	12	6	14	9	11	14	17	12	13	2.11	0.0547			2.86	0.0045	0.0428	up	1.36	0.321	1	3	1211	-2.49719157	-0.72043059	-0.99655084	0.49110758	-0.3299618	0.77425913	0.08907421	0.38761203	0.68366001	1.05245245	0.51071369	0.5552557
ENSMUSG00000114253	Gm47798	predicted gene	lncRNA	24	65	48	93	86	101	87	88	108	106	94	102	1.57	0.00886	0.123		1.79	0.000587	0.0119	up	1.14	0.422	1	3	1212	-2.54056702	-0.57315181	-1.25513241	0.4028138	0.1548107	0.62767856	0.2073197	0.25041161	0.83529466	0.78829871	0.43031155	0.67191195
ENSMUSG00000106665	Gm43389	predicted gene	TEC	37	75	48	85	84	106	103	92	126	114	107	105	1.57	0.00129	0.0562		1.84	0.00000599	0.000724	up	1.18	0.196	1	3	1213	-2.17369681	-0.47756533	-1.60693757	-0.08139744	-0.12097371	0.60143792	0.4891838	0.15405594	1.18366285	0.8347676	0.64260746	0.55485529
ENSMUSG00000105454	Gm43830	predicted gene	TEC	115	231	164	288	298	338	299	297	384	339	367	362	1.54	0.00429	0.0899		1.82	0.0000705	0.00304	up	1.18	0.266	1	3	1214	-2.31693258	-0.57739164	-1.49107044	0.07501194	0.17866309	0.57362711	0.18527371	0.17097064	0.98520426	0.58480086	0.8374067	0.79443635
ENSMUSG00000113146	Gm47406	predicted gene	processed_pseudoge	86	173	117	213	227	244	219	198	273	241	257	230	1.5	0.00487	0.0947		1.7	0.000226	0.0064	up	1.13	0.39	1	3	1215	-2.31814944	-0.46815632	-1.56355839	0.19096423	0.4031811	0.63382899	0.27850515	-0.04219946	1.02938319	0.59505161	0.82191931	0.43923003
ENSMUSG00000062296	Trank1	tetratricopeptic	protein_coding	4,742	5,784	5,204	6,202	6,532	6,670	6,365	6,741	7,032	7,038	6,651	6,897	1.2	0.00133	0.0568		1.26	0.0000457	0.00234	up	1.05	0.389	1	3	1216	-2.259475	-0.66415679	-1.51439138	-0.10157568	0.31724734	0.4860063	0.10713552	0.57242203	0.91454933	0.92165812	0.46329618	0.75728402
ENSMUSG00000097767	Miat	myocardial infa	lncRNA	5,275	8,680	6,114	9,703	9,544	10,910	9,059	10,269	10,975	11,253	10,980	11,306	1.34	0.00301	0.0793		1.5	0.0000381	0.00214	up	1.12	0.249	1	3	1217	-2.28069356	-0.27089392	-1.68839229	0.18245894	0.11517477	0.66082928	-0.09731883	0.41366639	0.68487165	0.78729801	0.68678816	0.80621141
ENSMUSG00000102858	Gm37086	predicted gene	TEC	272	501	338	538	542	630	528	606	651	648	601	621	1.4	0.00229	0.0714		1.53	0.000101	0.00384	up	1.09	0.409	1	3	1218	-2.34948151	-0.20500078	-1.62790885	0.06909108	0.10049244	0.69453298	-0.00063295	0.54163976	0.82621538	0.80818098	0.50687664	0.63599482
ENSMUSG00000031706	Rfx1	regulatory facto	protein_coding	526	666	588	721	767	837	771	783	840	850	845	800	1.26	0.0000501	0.0204	up	1.33	0.000000347	0.000149	up	1.06	0.322	1	3	1219	-2.20845325	-0.71792984	-1.51320083	-0.2011375	0.20770966	0.78810498	0.24509032	0.34663592	0.81093405	0.89322741	0.85781801	0.49120105
ENSMUSG00000109196	Gm44715	predicted gene	processed_pseudoge	97	164	116	212	209	266	205	225	247	263	254	228	1.53	0.000653	0.0455	up	1.68	0.0000263	0.00168	up	1.1	0.442	1	3	1220	-2.13414744	-0.65633482	-1.67376701	0.16443066	0.1106293	0.95660259	0.05075205	0.37207933	0.68903679	0.91812836	0.7974032	0.405187

ENSMUSG00000050545	Fam228b	family with seq	protein_coding	126	145	175	188	205	202	215	187	242	255	201	196	1.27	0.0295	0.216		1.41	0.0015	0.0214	up	1.11	0.325	1	3	1437	-2.05275322	-1.41938472	-0.50134667	-0.12713907	0.3354127	0.25207037	0.57688191	-0.16238238	1.24302546	1.53868185	0.2130509	0.10388288
ENSMUSG00000097331	F420014N23Rik	RIKEN cDNA F4	lncRNA	24	36	53	38	50	47	55	47	81	67	38	62	1.35	0.105	0.38		1.68	0.00337	0.0359	up	1.25	0.188	1	3	1438	-1.91768504	-0.89031214	0.29817936	-0.71322357	0.11838798	-0.12273743	0.38723563	-0.08854879	1.83893435	1.07262124	-0.77021145	0.78735985
ENSMUSG00000074449	Potefam3a	POTE ankyrin d	protein_coding	29	37	39	41	52	65	55	36	71	72	53	54	1.42	0.0699	0.316		1.71	0.00366	0.0378	up	1.21	0.275	1	3	1439	-1.62837293	-0.97959585	-0.77382709	-0.59341538	0.15466458	1.0549134	0.39552513	-0.99863662	1.40813768	1.41971586	0.23412601	0.30676521
ENSMUSG00000043833	2900005J15Rik	RIKEN cDNA 29	lncRNA	50	60	63	69	82	84	88	69	119	113	81	93	1.32	0.0312	0.221		1.68	0.0000281	0.00174	up	1.27	0.0394	1	3	1440	-1.6460211	-1.04375563	-0.89450088	-0.52556347	0.12497721	0.22032756	0.4107399	-0.57027235	1.71396855	1.48620157	0.0806482	0.64325045
ENSMUSG00000111761	9230112J17Rik	RIKEN cDNA 92	lncRNA	9	15	14	14	24	23	19	15	38	39	26	30	1.55	0.0547			2.54	0.00000915	0.000926	up	1.64	0.0107	1	3	1441	-1.63808404	-0.66828018	-0.79429128	-0.84257067	0.32177313	0.17355834	-0.23888361	-0.74057897	1.53798069	1.58027289	0.46673277	0.84237092
ENSMUSG00000021098	4930447C04Rik	RIKEN cDNA 49	protein_coding	593	719	727	914	741	978	778	725	1,065	946	881	1,039	1.09	0.305	0.623		1.33	0.000619	0.0123	up	1.22	0.0164	1	3	1442	-1.83177061	-0.80849205	-0.7420659	0.53119029	-0.64316612	0.91275768	-0.37212154	-0.75791507	1.4051267	0.72373596	0.31820281	1.26451785
ENSMUSG00000026409	Pfkfb2	6-phosphofruct	protein_coding	771	903	943	1,034	950	1,026	948	1,005	1,172	1,076	992	1,091	1.08	0.208	0.524		1.19	0.00303	0.0336	up	1.1	0.0878	1	3	1443	-2.29696724	-0.84305242	-0.4426582	0.42528902	-0.37472898	0.35130361	-0.39021112	0.16163778	1.63090652	0.80733247	0.03227913	0.93886944
ENSMUSG00000102994	Gm37767	predicted gene	TEC	0	1	3	7	4	3	4	3	18	5	10	14	1.17	0.779			4.24	0.00234	0.0285	up	3.64	0.00316	1	3	1444	-1.90788302	-0.86588456	-0.38525091	0.35682285	-0.20279361	-0.48618177	-0.18654854	-0.43967244	1.78422811	0.08795037	0.88055485	1.36465865
ENSMUSG00000057110	Cntrl	centriolin	protein_coding	405	487	529	664	642	784	678	633	864	689	823	835	1.31	0.00206	0.0682		1.54	0.000000784	0.000227	up	1.17	0.0639	1	3	1445	-2.01544378	-1.27997977	-0.93510569	0.0473758	-0.10596667	0.78629293	0.13892102	-0.16545099	1.23630712	0.20851651	1.00881518	1.07571833
ENSMUSG00000104275	Gm37867	predicted gene	lncRNA	5	8	11	15	13	17	15	14	24	15	21	28	1.51	0.17			2.26	0.00388	0.0391	up	1.5	0.112	1	3	1446	-1.8767082	-1.14605952	-0.73362998	-0.02610273	-0.36937734	0.33209105	0.08449724	-0.13651609	1.23640663	-0.00832821	0.91584523	1.72788192
ENSMUSG00000111990	Gm47033	predicted gene	lncRNA	37	45	62	72	63	98	69	59	98	85	92	85	1.33	0.0544	0.281		1.67	0.000333	0.0083	up	1.25	0.0931	1	3	1447	-1.90111037	-1.39032336	-0.44587117	0.07381249	-0.38101949	1.21684494	-0.06286073	-0.60110504	1.20154951	0.66466862	0.97219526	0.65321933
ENSMUSG00000004626	Stxbp2	syntaxin bindin	protein_coding	277	385	409	501	543	676	487	523	698	652	643	618	1.42	0.00238	0.0723		1.67	0.00000826	0.00089	up	1.17	0.158	1	3	1448	-2.1943799	-1.09990845	-0.88821882	-0.15374581	0.14727486	0.99966448	-0.25760828	0.00891908	1.12692714	0.85990614	0.80469661	0.64647295
ENSMUSG00000096822	Or1j15	olfactory recep	protein_coding	61	86	84	112	119	145	104	102	153	125	135	121	1.37	0.018	0.174		1.55	0.000573	0.0118	up	1.14	0.289	1	3	1449	-2.09281192	-0.95649538	-1.02565356	0.06108464	0.32108113	1.16182585	-0.24174417	-0.32731897	1.39625824	0.50629289	0.83133224	0.36614902
ENSMUSG00000102813	Gm37795	predicted gene	TEC	15	22	32	36	39	41	26	30	46	48	46	45	1.29	0.193			1.77	0.00177	0.0236	up	1.37	0.0608	1	3	1450	-2.14348386	-1.17342995	-0.2607506	0.13658038	0.38649526	0.51963568	-0.83906711	-0.4248645	0.93671299	1.09196651	0.91074816	0.85945705
ENSMUSG00000105700	Gm42772	predicted gene	TEC	0	6	6	12	13	13	9	7	18	16	17	10	1.76	0.114			2.56	0.00498	0.0454	up	1.46	0.187	1	3	1451	-2.6810181	-0.59742687	-0.52737545	0.35596774	0.42645092	0.49013768	-0.05288272	-0.28422168	1.0165682	0.76766964	0.95058455	0.13554609
ENSMUSG00000103831	Gm37608	predicted gene	TEC	15	30	33	33	48	56	42	47	58	49	50	59	1.75	0.000626	0.0449	up	1.96	0.0000171	0.00127	up	1.12	0.399	1	3	1452	-2.39181833	-0.9325003	-0.6490904	-0.62766158	0.38218474	0.9024591	0.02071106	0.30516107	0.99121476	0.44550374	0.50111257	1.05272358
ENSMUSG00000103047	Gm37310	predicted gene	TEC	19	38	44	51	74	80	56	60	117	103	71	113	1.79	0.00319	0.0804		2.69	0.000000248	0.000128	up	1.5	0.0231	1	3	1453	-1.99454178	-0.99651359	-0.76243082	-0.48829702	0.28978068	0.45713597	-0.28373457	-0.1727181	1.39307409	1.06324047	0.19203367	1.30297099
ENSMUSG00000106832	Gm42632	predicted gene	TEC	11	17	18	19	26	30	19	23	33	35	30	32	1.54	0.0425			2.05	0.000333	0.0083	up	1.33	0.102	1	3	1454	-1.97594435	-0.93998157	-0.77103894	-0.6802249	0.30844851	0.72986367	-0.60376228	-0.06895584	1.05578379	1.28867505	0.76025928	0.89687758
ENSMUSG00000078190	Dnm3os	dynamin 3, opp	lncRNA	8	12	25	18	24	30	26	24	50	46	36	51	1.93	0.0306			3.38	0.0000339	0.00199	up	1.75	0.0271	1	3	1455	-1.80938215	-1.32869436	-0.14062547	-0.73461296	-0.22093923	0.16239788	-0.10821666	-0.2137236	1.35163223	1.11869783	0.5491537	1.3743128
ENSMUSG00000097619	4833422M21Rik	RIKEN cDNA 48	lncRNA	16	31	33	39	34	42	47	37	56	57	50	58	1.35	0.0899	0.353		1.88	0.00017	0.00543	up	1.39	0.0341	1	3	1456	-2.29246865	-0.78127064	-0.6258446	-0.12556039	-0.49075991	0.09009026	0.47296314	-0.25292754	1.02675929	1.12663472	0.65148822	1.2008961
ENSMUSG00000078813	Leng1	leukocyte recep	protein_coding	226	233	273	259	267	275	245	261	282	278	344	316	1.06	0.395	0.7		1.23	0.000982	0.0167	up	1.16	0.0129	1	3	1457	-1.47558847	-1.20870154	0.10067354	-0.36497694	-0.11664537	0.14958393	-0.82368276	-0.29852011	0.351111	0.23518912	2.0996565	1.3519011
ENSMUSG00000070000	Fcho1	FCH domain on	protein_coding	1,123	1,186	1,280	1,386	1,296	1,348	1,217	1,269	1,429	1,419	1,589	1,482	1.03	0.562	0.813		1.19	0.00109	0.0178	up	1.15	0.0069	1	3	1458	-1.6811574	-1.1444523	-0.38750254	0.41450113	-0.26069335	0.13605199	-0.89316341	-0.47186783	0.72918058	0.65294143	1.80957298	1.09658874
ENSMUSG00000110529	Gm45694	predicted gene	TEC	31	42	48	61	59	71	46	58	82	66	97	91	1.28	0.112	0.391		1.84	0.0000377	0.00214	up	1.43	0.00944	1	3	1459	-1.81287707	-1.03724738	-0.68834614	0.01881018	-0.09512994	0.46771781	-0.80082891	-0.1640704	0.95150165	0.23479509	1.57922734	1.34644776
ENSMUSG00000115837	9130002K18Rik	RIKEN cDNA 91	TEC	9	24	12	19	33	35	15	28	45	52	49	49	1.74	0.0126			3.05	8.06E-08	0.0000671	up	1.75	0.00176	1	3	1460	-1.60630206	-0.32709007	-1.31391055	-0.71942673	0.2415953	0.39808473	-0.99722598	-0.05377862	0.91915826	1.27362511	1.09359126	1.09167935
ENSMUSG00000015501	Hivep2	human immunc	protein_coding	2,511	2,656	2,484	2,820	2,959	2,938	2,455	2,916	3,161	3,112	3,237	3,252	1.08	0.0908	0.354		1.22	0.00000418	0.000617	up	1.13	0.00355	1	3	1461	-1.26417768	-0.72466039	-1.37055575	-0.14342219	0.32417696	0.25223374	-1.48032051	0.18167358	0.9675612	0.81457584	1.19869109	1.2442241
ENSMUSG00000112074	Gm48278	predicted gene	lncRNA	54	73	62	89	107	98	72	97	128	123	129	133	1.34	0.00946	0.128		1.84	0.000000011	0.0000207	up	1.37	0.00128	1	3	1462	-1.6719554	-0.83316145	-1.33221457	-0.1993722	0.41323949	0.12011979	-0.88996334	0.07209529	1.09182345	0.91666642	1.09832854	1.21439398
ENSMUSG00000004567	Mcoln1	mucolipin 1	protein_coding	1,283	1,293	1,283	1,371	1,431	1,398	1,278	1,425	1,524	1,478	1,590	1,492	1.06	0.0938	0.361		1.16	0.00000485	0.000644	up	1.1	0.00373	1	3	1463	-1.16926513	-1.06181864	-1.16349159	-0.28863493	0.28273602	-0.02745552	-1.21817581	0.2306838	1.13294254	0.72257443	1.70904893	0.8508559
ENSMUSG00000085037	4933421O10Rik	RIKEN cDNA 49	lncRNA	313	308	312	349	384	375	319	371	411	422	434	389	1.13	0.0515	0.275		1.29	0.0000214	0.00147	up	1.14	0.0211	1	3	1464	-1.18164231	-1.31890536	-1.20419165	-0.322084	0.43161863	0.24002578	-1.04209168	0.154341	1.00718563	1.22767848	1.46170642	0.54635905
ENSMUSG00000106819	Gm43498	predicted gene	processed_pseudoge	206	236	239	337	361	358	290	363	395	400	457	405	1.35	0.00305	0.0796		1.63	0.000000843	0.000232	up	1.21	0.0502	1	3	1465	-1.80066303	-1.30547107	-1.26228578	0.07906246	0.35659558	0.3196485	-0.5327449	0.38525629	0.73708004	0.79861129	1.37484391	0.84974381
ENSMUSG00000107320	Gm42549	predicted gene	TEC	28	38	41	68	86	76	49	77	99	106	117	94	1.65	0.00342	0.0817		2.38	0.000000141	0.0000988	up	1.44	0.0164	1	3	1466	-1.74721537	-1.23966543	-1.12083267	-0.05971279	0.4913936	0.19972082	-0.77353053	0.24180097	0.86624607	1.07095331	1.32777928	0.74306275
ENSMUSG00000105416	Gm42553	predicted gene	TEC	373	375	383	532	522	538	429	515	560	593	691	586	1.2	0.0331	0.226		1.46	0.0000108	0.00102	up	1.21	0.0231	1	3	1467	-1.42536155	-1.39676815	-1.29995281	0.30094505	0.20140291	0.36078029	-0.76199525	0.13702792	0.56106097	0.86135608	1.66697069	0.79453384
ENSMUSG00000106986	Gm42887	predicted gene	TEC	482	502	505	587	632	599	512	603	629	652	735	666	1.13	0.0683	0.312		1.29	0.000108	0.00403	up	1.14	0.0406	1	3	1468	-1.47520965	-1.1788373	-1.13187222	-0.02107116	0.5501898	0.1327491	-1.02738912	0.1839066	0.50694055	0.79250657	1.72033356	0.94775327
ENSMUSG00000024170	Telo2	telomere maint	protein_coding	367	343	422	403	468	576	371	469	495	430	496	514	1.23	0.0141	0.155		1.26	0.00438	0.0419	up	1.03	0.714	1	3	1469	-1.18110501	-1.58432606	-0.29941356	-0.59912676	0.36776079	1.74514173	-1.10265418	0.37155968	0.73702997	-0.18980592	0.7504582	0.98448111
ENSMUSG00000026970	Rbms1	RNA binding mo	protein_coding	519	551	577	572	766	752	514	653	798	649	775	751	1.21	0.017	0.169		1.34	0.000196	0.00588	up	1.11	0.188	1	3	1470	-1.31600609	-0.97811789	-0.70377626	-0.75769464	1.00522204	0.88810457	-1.38189342	0.0285672	1.26242712	-0.00678596	1.07454881	0.88540452
ENSMUSG00000047789	Slc38a9	solute carrier fa	protein_coding	663	708	734	803	915	853	720	796	909	825	976	918	1.13	0.0317	0.222		1.25	0.0000692	0.003	up	1.1	0.0685	1	3	1471	-1.65526624	-1.13506602	-0.83666258	-0.10539021	0.97132978	0.38825767	-0.99095241	-0.17849494	0.91627965	0.10842752	1.515879	1.00166479
ENSMUSG00000022070	Bora	bora, aurora kir	protein_coding	92	108	110	105	113	130	100	125	146	126	136	132	1.13	0.203	0.519		1.3	0.00402	0.0398	up	1.15	0.101	1	3	1472	-1.74109	-0.66028609	-0.48689031	-0.8006526	-0.28231422	0.69534498	-1.13480287	0.41899751	1.60274178	0.49514571	1.04882817	0.84497794
ENSMUSG00000037355	Uvssa	UV stimulated s	protein_coding	572	651	595	741	785	791	624	729	876	843	820	813	1.14	0.0584	0.292		1.31	0.000117	0.00423	up	1.14	0.0504	1	3	1473	-1.66700119	-0.8005316	-1.40938619	0.09658416	0.4953339	0.5559967	-1.08812745	-0.01681045	1.27638322	1.0027174	0.81209026	0.74275124
ENSMUSG00000091945	Vmn2r114	vomeronasal 2,	protein_coding	70	121	93	153	196	213	102	175	230	206	232	220	1.57	0.00117	0.0548		2.03	0.000000201	0.000122	up	1.29	0.0498	1	3	1474	-1.85516346	-0.71408271	-1.29651348	-0.12786957	0.53385966	0.77558009	-1.0892905	0.22091255	0.9910472	0.67541232	1.02119464	0.86491326
ENSMUSG00000108264	Gm20371	predicted gene	TEC	3	10	8	33	41	48	14	34	56	55	40	73	2.56	0.00866			4.17	0.0000487	0.00244	up	1.63	0.136	1	3	1475	-1.79618784	-1.08524952	-1.27136328	0.13853324	0.44259253	0.67812313	-0.79621375	0.14620043	0.91703775	0.87268155	0.37254315	1.38130262
ENSMUSG00000086607	4930511M06Rik	RIKEN cDNA 49	lncRNA	81	96	85	133	138	161	106	141	180	167	174	191	1.38	0.00922	0.125		1.8	0.00000107	0.00028	up	1.3	0.021	1	3	1476	-1.59145534	-1.08877667	-1.43134558	-0.02920093	0.09913027	0.63468916	-0.7779722	0.16275199	1.04897464	0.77025922	0.91746865	1.28547678
ENSMUSG00000117853	Vmn1r88	vomeronasal 1	protein_coding	100	146	132	191	206	240	164	209	279	242	267	284	1.44	0.000929	0.0514		1.88	3.17E-09	0.0000131	up	1.31	0.00849	1	3	1477	-1.94742562	-0.93113863	-1.21304913	-0.13491061	0.11288285	0.61037147	-0.59645197	0.15053685	1.13951007	0.63815013	0.97705455	1.19447004
ENSMUSG00000105530	Gm43829	predicted gene	TEC	1	8	5	12	14	28	6	18	31	24	21	24	2.49	0.0121			3.79	0.000134	0.00465	up	1.53	0.152	1	3	1478	-1.92077101	-0.63065034	-1.20475538	-0.22060495	-0.03518543	1.0688947	-0.90451966	0.31955093	1.27704029	0.794254	0.61425228	0.84249458
ENSMUSG00000109551	4930435N07Rik	RIKEN cDNA 49	TEC	0	4	3	16	11	37	5	19	38	28	31	36	3.06	0.0132			5.72	0.0000739	0.00315	up	1.87	0.107	1	3	1479	-1.92988144	-0.90749856	-1.06646344	0.05292688	-0.25976531	1.049485	-0.80325962	0.20732053	1.11266132	0.68513405	0.83282963	1.02651098
ENSMUSG00000068950	Or1j10	olfactory recep	protein_coding	97	189	161	198	238	278	196	246	283	266	290	296	1.49	0.0032	0.0804		1.76	0.0000204	0.00142	up	1.18	0.192	1	3	1480	-2.38298845	-0.53165152	-1.01703104	-0.38297252	0.22881766	0.77194722	-0.40492031	0.34373109	0.83437725	0.62004227	0.92687146	0.99377691
ENSMUSG00000102136	Gm37107	predicted gene	lncRNA	0	10	2	11	20	30	6	15	28	27	28	35	3.22	0.00547			5.39	0.0000386	0.00214	up	1.67	0.139	1	3	1481	-2.06458756	-0.35936808	-1.39621186	-0.28959883	0.3641691	0.89695103	-0.68699348	0.02989018	0.79678282	0.75962828	0.81156507	1.13777334
ENSMUSG00000103630	Gm37242	predicted gene	TEC	45	49	66	99	80	110	54	88	118	101	84	104	1.28	0.113	0.393		1.57	0.00317	0.0346	up	1.22	0.171	1	3	1482	-1.64317195	-1.42749943	-0.62167956	0.64872803	-0.02585996	1.03189805	-1.19445879	0.28111717	1.29600184	0.71168329	0.11616963	0.82707169
ENSMUSG00000112169	Gm48536	predicted gene	TEC	19	24	42	67	54	59	32	62	83	61	68	73	1.38	0.118	0.402		1.88	0.00141	0.0207	up	1.37	0.0988	1	3	1483	-1.91211282	-1.52015009	-0.44832997	0.65497931	0.12427507	0.33684377	-1.00013538	0.4817309	1.24630064	0.43758521	0.703176	0.89583735
ENSMUSG00000034610	Tut4	terminal uridyly	protein_coding	1,399	1,383	1,376	1,803	1,790	1,948	1,542	1,721	2,080	2,032	1,862	1,982	1.17	0.0239	0.195		1.33	0.0000432	0.0023	up	1.14	0.0678	1	3	1484	-1.35377615	-1.42692346	-1.46031906	0.28281485	0.23381057	0.79032566	-0.73410638	-0.02187201	1.22226588	1.06915016	0.49329046	0.90533946
ENSMUSG00000106386	Gm42991	predicted gene	TEC	1	3	2	23	18	39	8	19	46	39	39	40	2.89	0.0153			5.7	0.0000469	0.00238	up	1.97	0.0788	1	3	1485	-1.49047284	-1.24105308	-1.45557392	0.21135808	-0.05265575	0.90597986	-0.72162453	0.00364264	1.13835869	0.90134054	0.87747231	0.92322802
ENSMUSG00000021182	Ccdc88c	coiled-coil dom	protein_coding	670	695	683	884	838	1,000	828	901	1,079	918	975	1,080	1.22	0.000697	0.0463	up	1.38	1.25E-08	0.0000216	up	1.14	0.0226	1	3	1486	-1.52601596	-1.31882198	-1.41874601	0.09306103	-0.22218512	0.84317153	-0.29315178	0.20883262	1.3114197	0.32152543	0.68771893	1.31319161
ENSMUSG00000107826	Gm44242	predicted gene	TEC	4	4	6	21	15	34	14	31	49	36	31	40	2.63	0.00353			4.37	0.00000427	0.000617	up	1.66	0.0674	1	3	1487	-1.49713282	-1.46334742	-1.23107524	0.02380583	-0.3993565	0.71683128	-0.47443439	0.55149596	1.38480083	0.81957235	0.58627322	0.98256691
ENSMUSG00000028653	Trit1	tRNA isopenter	protein_coding	253	261	325	239	260	312	307	324	366	409	321	290	1.12	0.161	0.465		1.29	0.00151	0.0215	up	1.15	0.074	1	3	1488	-1.09880413	-0.91687672	0.43852179	-1.43783124	-0.93790938	0.17693215	0.07593971	0.41802114	1.21666465	1.97762752	0.36455076	-0.27683626
ENSMUSG00000113640	Adat3	adenosine dear	protein_coding	0	5	4	5	0	21	17	21	29	32	19	14	3.9	0.0225			6.27	0.00176	0.0236	up	1.61	0.36	1	3	1489	-1.69083363	-0.50121189	-0.62517097	-0.53225563	-1.69083363	0.69961447	0.47399131	0.66739875	1.11370482	1.2536167	0.55612809	0.27585161
ENSMUSG00000027208	Fgf7	fibroblast grow	protein_coding	38	33	42	40	30	63	50	52	52	75	56	62	1.27	0.142	0.438		1.6	0.00246	0.0294	up	1.26	0.103	1	3	1490	-0.8330226	-1.27806253	-0.54051744	-0.66747123	-1.5786399	1.03518032	0.13534808	0.21727183	0.26075705	1.73572096	0.5570189	0.95641657
ENSMUSG00000070458	Vmn2r73	vomeronasal 2,	protein_coding	8	8	8	8	4	21	14	10	21	18	20	18	1.51	0.193			2.39	0.00326	0.0352	up	1.58	0.0811	1	3	1491	-0.77696529	-0.68509949	-0.86974904	-0.78042263	-1.77923348	1.18838167	0.25560094	-0.3633484	1.20977786	0.76582514	1.07897607	0.75625665
ENSMUSG00000105549	Gm43540	predicted gene	processed_pseudoge	129	139	167	193	124	206	147	206	197	228	262	222	1.09	0.464	0.751		1.45	0.00148	0.0212	up	1.33	0.0135	1	3	1492	-1.35149119	-1.06372733	-0.35843774	0.2557777	-1.47586278	0.51947708	-0.84612346	0.52359166	0.34382144	0.98253547	1.60591584	0.8645233
ENSMUSG00000105974	Gm43318	predicted gene	TEC	8	25	18	37	28	48	21	34	62	62	65	60	1.48	0.157			2.81	0.000097	0.00376	up	1.9	0.0108	1	3	1493	-1.89676716	-0.5797669	-1.04447163	0.05390409	-0.44508141	0.54717535	-0.87740938	-0.08628863	1.07361019	1.07204911	1.1713759	1.01167047
ENSMUSG00000117819	Gm50253	predicted gene	protein_coding	112	173	161	180	158	203	141	170	204	222	230	225	1.08	0.499	0.775		1.41	0.00113	0.0182	up	1.31	0.0091	1	3	1494	-2.08609108	-0.17398652	-0.51494419	0.01931351	-0.58124237	0.60712664	-1.10524614	-0.24176046	0.63729012	1.07138868	1.2467882	1.12136362
ENSMUSG00000032816	Igdcc4	immunoglobuli	protein_coding	1,744	1,926	1,751	1,979	1,799	2,010	1,865	1,948	1,997	2,108	2,192	2,055	1.03	0.455	0.745		1.13	0.00196	0.0254	up	1.1	0.0185	1	3	1495	-1.49963987	-0.12886363	-1.44682877	0.25130154	-1.07643566	0.46647418	-0.57180274	0.03038365	0.37938264	1.13394634	1.68553255	0.77654978
ENSMUSG00000052296	Ppp6r1	protein phosph	protein_coding	3,028	3,248	3,205	3,235	3,152	3,351	3,178	3,324	3,304	3,416	3,490	3,401	1.02	0.303	0.622		1.07	0.00192	0.025	up	1.05	0.038	1	3	1496	-1.97975426	-0.21227171	-0.55162694	-0.3128883	-0.96731748	0.57409149	-0.75922447	0.374053	0.21657605	1.06367694	1.60151685	0.95316883
ENSMUSG00000109916	2410039M03Rik	RIKEN cDNA 24	unprocessed_pseudo	24	28	39	28	35	48	44	57	42	61	59	47	1.57	0.00706	0.111		1.76	0.000398	0.00939	up	1.12	0.415	1	3	1497	-1.62906871	-1.19607097	-0.2122148	-1.20919704	-0.5437504	0.51323049	0.14358607	1.09815286	-0.00411287	1.39358155	1.22679436	0.41906947
ENSMUSG00000028681	Ptch2	patched 2	protein_coding	96	107	129	117	100	162	132	150	116	165	224	159	1.21	0.125	0.413		1.47	0.00186	0.0245	up	1.21	0.113	1	3	1498	-1.28907397	-0.88815827	-0.21241088	-0.56821815	-1.12457969	0.71931933	-0.0993037	0.410866	-0.59896259	0.80865892	2.18575926	0.65610373
ENSMUSG00000109368	Gm45015	predicted gene	protein_coding	12	17	17	12	10	21	19	20	22	29	35	28	1.23	0.396			2	0.00184	0.0243	up	1.62	0.0176	1	3	1499	-1.14353323	-0.36251219	-0.41179237	-1.13475094	-1.48758046	0.19909371	-0.03231916	0.01396776	0.34827532	1.13669629	1.82792559	1.04652968
ENSMUSG00000105063	Gm43488	predicted gene	TEC	7	20	17	29	28	30	29	28	29	35	51	29	1.59	0.0481			1.99	0.00239	0.0289	up	1.25	0.283	1	3	1500	-2.36313496	-0.64822994	-0.98827571	0.24415519	0.10136052	0.30934406	0.25491423	0.12573086	0.20987995	0.73072691	1.76905806	0.25447084
ENSMUSG00000117815	Gm50166	predicted gene	lncRNA	8	22	23	25	36	35	41	46	40	45	62	32	2.04	0.000703			2.28	0.0000545	0.00262	up	1.12	0.536	1	3	1501	-2.28819013	-0.77940029	-0.77642175	-0.55223164	0.22335277	0.15285098	0.50270468	0.77119879	0.44257396	0.73985498	1.60719413	-0.04348647
ENSMUSG00000109378	Gm49396	predicted gene	protein_coding	97	121	104	145	189	152	167	160	158	181	206	164	1.43	0.00108	0.0533		1.52	0.0000827	0.0034	up	1.06	0.547	1	3	1502	-1.82297681	-0.96041932	-1.5660172	-0.19526924	1.02834502	0.01537408	0.43631836	0.24162688	0.18777785	0.83150922	1.45137996	0.35235121
ENSMUSG00000111236	Gm31614	predicted gene	lncRNA	8	14	11	23	33	30	29	20	31	34	40	21	2	0.0049			2.23	0.000703	0.0133	up	1.12	0.581	1	3	1503	-1.83205508	-0.97540657	-1.44287041	-0.04725178	0.82030334	0.61354173	0.5591178	-0.35721739	0.65537315	0.92225358	1.33288941	-0.24867779
ENSMUSG00000050954	Zfp169	zinc finger prot	protein_coding	253	307	308	393	365	384	384	415	397	526	454	435	1.23	0.0214	0.188		1.44	0.0000435	0.0023	up	1.17	0.0757	1	3	1504	-1.9908697	-1.08263624	-1.05397946	0.17315397	-0.20612694	0.04703256	0.04958088	0.45917759	0.21825597	1.74547455	0.93187978	0.70905705
ENSMUSG00000071665	Foxr2	forkhead box R	protein_coding	81	97	88	105	114	112	111	100	118	152	130	122	1.17	0.145	0.441		1.4	0.000952	0.0163	up	1.2	0.0627	1	3	1505	-1.68572848	-0.70910428	-1.24983719	-0.25325046	0.2046058	0.10859139	0.05664872	-0.54576724	0.41606587	2.01494241	1.03587029	0.60696317
ENSMUSG00000107546	Gm20560	predicted gene	lncRNA	1	1	2	4	6	4	8	7	5	10	11	9	2.95	0.0402			4.25	0.00402	0.0398	up	1.44	0.319	1	3	1506	-1.4374327	-1.39418479	-1.3422933	-0.33898727	0.31577831	-0.36232696	0.65788178	0.37524461	-0.00385354	1.22867978	1.38596213	0.91553194
ENSMUSG00000029202	Pds5a	PDS5 cohesin a	protein_coding	1,696	1,823	1,751	1,904	1,998	2,015	2,103	2,064	2,041	2,205	2,157	2,036	1.14	0.000408	0.0378	up	1.18	0.00000893	0.000919	up	1.03	0.389	1	3	1507	-1.86321204	-0.99055595	-1.48062467	-0.46345675	0.12672293	0.23265625	0.7573655	0.52651719	0.38634876	1.34163037	1.06851157	0.35809684
ENSMUSG00000032175	Tyk2	tyrosine kinase	protein_coding	269	297	268	321	344	324	337	358	360	377	389	335	1.18	0.0191	0.179		1.26	0.000719	0.0135	up	1.07	0.318	1	3	1508	-1.66269334	-0.87291005	-1.70398891	-0.22914108	0.35366373	-0.16106866	0.17073745	0.69362132	0.73229019	1.13863837	1.41705327	0.12379772
ENSMUSG00000051037	Zfp455	zinc finger prot	protein_coding	104	86	109	130	108	107	130	125	147	137	139	133	1.1	0.322	0.638		1.29	0.00435	0.0417	up	1.18	0.0587	1	3	1509	-0.95179804	-2.05465222	-0.65310675	0.51537647	-0.67220412	-0.73235082	0.51111447	0.21906426	1.32790559	0.853352	0.97831017	0.658989
ENSMUSG00000045106	Ccdc73	coiled-coil dom	protein_coding	220	236	285	367	299	333	389	317	367	379	409	413	1.21	0.0407	0.247		1.42	0.000108	0.00403	up	1.17	0.0698	1	3	1510	-1.88794199	-1.5677744	-0.70901809	0.53106195	-0.4873842	0.04685781	0.82397165	-0.19679255	0.53073552	0.69197002	1.08627547	1.1380388
ENSMUSG00000029607	Ankrd61	ankyrin repeat	protein_coding	17	14	23	28	24	31	29	32	39	35	39	39	1.42	0.0695			1.85	0.00078	0.0142	up	1.3	0.111	1	3	1511	-1.45588173	-1.94649783	-0.72839968	-0.02281262	-0.55022572	0.32113211	0.09451792	0.35022914	1.08893536	0.65632243	1.1319517	1.06072891
ENSMUSG00000030322	Mbd4	methyl-CpG bin	protein_coding	206	217	224	269	235	274	262	264	300	279	291	276	1.13	0.0838	0.343		1.25	0.000941	0.0162	up	1.11	0.115	1	3	1512	-1.77056742	-1.36389025	-1.1201895	0.40036591	-0.72680989	0.52878648	0.15522078	0.21785974	1.31137172	0.70762336	1.06648921	0.59373987
ENSMUSG00000097923	Gm26577	predicted gene	lncRNA	4	6	5	7	6	16	14	13	14	16	18	10	2.36	0.0129			2.82	0.00172	0.0234	up	1.19	0.491	1	3	1513	-1.45112241	-0.99119136	-1.2950649	-0.73338375	-0.82516193	0.94806282	0.69328058	0.51967069	0.75286116	1.02962359	1.31842599	0.03399951
ENSMUSG00000104391	Gm37960	predicted gene	TEC	13	11	14	19	14	28	28	27	29	28	36	24	1.7	0.0387			2.06	0.00349	0.0367	up	1.21	0.39	1	3	1514	-1.16464986	-1.49495654	-1.12620521	-0.39703742	-1.07961174	0.64257545	0.71483693	0.58941248	0.79252135	0.70346066	1.52923715	0.29041674
ENSMUSG00000112844	Gm47036	predicted gene	processed_pseudoge	65	72	62	87	88	141	135	116	118	132	149	140	1.68	0.00238	0.0723		1.89	0.000157	0.00518	up	1.12	0.47	1	3	1515	-1.40703018	-1.14955903	-1.54784568	-0.61341299	-0.56912211	0.97715204	0.79964587	0.30698742	0.35982084	0.74063518	1.16435132	0.93837732
ENSMUSG00000097231	Gm26852	predicted gene	lncRNA	23	22	18	24	33	45	44	54	47	46	56	48	2.02	0.000219			2.24	0.0000111	0.00104	up	1.11	0.508	1	3	1516	-1.16432477	-1.17198533	-1.61382653	-1.03250951	-0.31189844	0.52149371	0.45820368	1.10487241	0.66710311	0.60432845	1.20500654	0.73353669
ENSMUSG00000103685	Gm37074	predicted gene	TEC	4	4	5	9	9	13	18	20	15	19	24	20	2.71	0.0027			3.53	0.0000761	0.0032	up	1.3	0.277	1	3	1517	-1.45210792	-1.39632593	-1.32931293	-0.43436409	-0.51942663	0.10453603	0.69057635	0.86813607	0.38492353	0.83079742	1.34899662	0.90357146
ENSMUSG00000103693	Gm37529	predicted gene	lncRNA	7	6	3	12	13	17	17	22	18	21	24	26	2.51	0.0137			3.24	0.00116	0.0184	up	1.29	0.41	1	3	1518	-1.124274	-1.31593574	-1.91257954	-0.29835	-0.22317431	0.31351611	0.27186725	0.86654783	0.40660592	0.77618642	1.05362771	1.18596236
ENSMUSG00000105469	A930036I15Rik	RIKEN cDNA A9	TEC	8	14	9	20	18	38	24	20	28	25	25	38	1.94	0.0129			2.29	0.00134	0.02	up	1.18	0.471	1	3	1519	-1.68815356	-0.80995329	-1.51590934	-0.13109389	-0.39102636	1.44029804	0.30114132	-0.07943193	0.64054422	0.37407869	0.39334011	1.46616598
ENSMUSG00000038708	Golga4	golgi autoantig	protein_coding	3,528	4,085	3,665	4,437	4,320	4,777	4,478	4,545	4,476	4,418	4,768	5,104	1.15	0.00663	0.108		1.19	0.000683	0.0131	up	1.04	0.5	1	3	1520	-1.99252959	-0.61871737	-1.63620842	0.16034732	-0.09224179	0.85816979	0.2462877	0.38627081	0.24213335	0.11959428	0.84006659	1.48682732
ENSMUSG00000073116	Gm10471	predicted gene	protein_coding	13	24	18	28	30	42	38	39	41	36	45	49	1.8	0.0048			2.04	0.000371	0.0089	up	1.14	0.473	1	3	1521	-2.03104178	-0.80055223	-1.43407782	-0.40482756	-0.20860668	0.74325456	0.47836771	0.53116789	0.66319373	0.30168089	0.93956414	1.22187713
ENSMUSG00000040850	Psme4	proteasome (pr	protein_coding	1,000	1,165	1,012	1,184	1,120	1,433	1,238	1,349	1,388	1,326	1,449	1,335	1.18	0.0228	0.192		1.26	0.0012	0.0187	up	1.07	0.342	1	3	1522	-1.68170609	-0.50004508	-1.59076786	-0.37332789	-0.81152941	1.13848172	-0.02690877	0.65414331	0.88193334	0.51545026	1.22527378	0.56900268
ENSMUSG00000027365	Trpm7	transient recep	protein_coding	1,439	1,721	1,527	1,786	1,795	2,076	1,902	2,012	2,105	1,973	2,118	1,985	1.2	0.00141	0.0581		1.26	0.0000449	0.00233	up	1.05	0.385	1	3	1523	-2.02871741	-0.61441582	-1.56079167	-0.31831357	-0.27741891	0.89303467	0.18559596	0.63997527	1.00884805	0.48336278	1.05642897	0.53241169
ENSMUSG00000034973	Dop1a	DOP1 leucine zi	protein_coding	1,743	2,192	1,947	2,226	2,255	2,639	2,269	2,520	2,654	2,602	2,698	2,648	1.19	0.000399	0.0378	up	1.31	7.26E-08	0.0000671	up	1.09	0.0673	1	3	1524	-2.12847581	-0.49376716	-1.34449994	-0.38328031	-0.29217397	0.85026222	-0.24536891	0.51417849	0.89184867	0.74658429	1.01122661	0.87346582
ENSMUSG00000103983	Gm20045	predicted gene	lncRNA	114	163	149	213	255	293	227	265	272	255	318	279	1.63	0.0000214	0.0191	up	1.76	0.00000057	0.000193	up	1.08	0.476	1	3	1525	-2.07545296	-1.07011016	-1.34347798	-0.21845399	0.39258964	0.89308396	-0.01495316	0.5187159	0.62276955	0.38878389	1.19544325	0.71106206
ENSMUSG00000107577	Gm44103	predicted gene	TEC	35	51	48	67	72	93	77	87	89	89	108	98	1.64	0.00096	0.0518		1.92	0.00000702	0.000793	up	1.17	0.239	1	3	1526	-2.02464019	-1.19792091	-1.23207877	-0.32313447	-0.10519692	0.74232191	0.11406059	0.50193723	0.60627892	0.60871287	1.28718279	0.92247696
ENSMUSG00000115027	Vmn1r81	vomeronasal 1	protein_coding	179	257	221	312	350	468	341	365	441	462	514	515	1.57	0.000048	0.0204	up	1.99	2.94E-10	0.00000152	up	1.27	0.0251	1	3	1527	-1.87840782	-0.94116346	-1.35183217	-0.39239793	-0.05647026	0.85609231	-0.13242588	0.07552538	0.66584598	0.81625474	1.16713121	1.17184789
ENSMUSG00000115105	Gm49238	predicted gene	TEC	40	63	50	79	74	123	81	91	100	106	124	121	1.59	0.00281	0.0767		1.95	0.0000107	0.00101	up	1.22	0.157	1	3	1528	-1.87189769	-0.80460225	-1.39509576	-0.21958415	-0.38334983	1.15994151	-0.14801696	0.20956011	0.50048955	0.68271442	1.17480201	1.09503904
ENSMUSG00000037593	Rskr	ribosomal prot	protein_coding	128	222	151	249	238	340	231	265	298	278	361	319	1.43	0.0048	0.0941		1.67	0.0000384	0.00214	up	1.17	0.195	1	3	1529	-2.02085951	-0.39249075	-1.57559522	-0.01096609	-0.16120793	1.08792626	-0.26667882	0.19910074	0.61253209	0.36217617	1.314165	0.85189806
ENSMUSG00000040860	Crocc	ciliary rootlet c	protein_coding	1,268	1,860	1,338	2,006	2,191	2,640	2,020	2,345	2,309	2,567	3,043	2,628	1.42	0.000114	0.0251	up	1.63	7.52E-08	0.0000671	up	1.15	0.13	1	3	1530	-1.91456755	-0.50556484	-1.71957527	-0.22006258	0.11571069	0.8305587	-0.19357555	0.37402822	0.31550943	0.72248256	1.38232648	0.81272971
ENSMUSG00000028093	Acp6	acid phosphata	protein_coding	267	342	292	315	373	422	362	403	406	451	461	395	1.28	0.000278	0.0331	up	1.41	0.000000338	0.000148	up	1.1	0.154	1	3	1531	-1.87102543	-0.46655097	-1.36258968	-0.93908234	0.03985472	0.78260536	-0.13902461	0.50239834	0.54544822	1.194126	1.33920704	0.37463336
ENSMUSG00000084897	Gm14226	predicted gene	lncRNA	49	76	56	76	83	96	88	93	85	101	110	91	1.4	0.00442	0.0913		1.51	0.000333	0.0083	up	1.07	0.498	1	3	1532	-2.07002772	-0.37108703	-1.62059062	-0.36490438	0.0175747	0.66750874	0.3034454	0.54422453	0.13039383	0.94057639	1.36999012	0.45289604
ENSMUSG00000036882	Arhgap33	Rho GTPase act	protein_coding	1,106	1,349	1,171	1,339	1,630	1,726	1,620	1,487	1,527	1,701	1,867	1,844	1.3	3.31E-08	0.00017	up	1.4	8.65E-13	1.79E-08	up	1.07	0.118	1	3	1533	-1.82172922	-0.67844764	-1.49679107	-0.72131271	0.44193062	0.78467661	0.40380662	-0.10419962	0.05220989	0.69672378	1.25898169	1.18415105
ENSMUSG00000104027	Gm37211	predicted gene	TEC	1	4	2	3	8	8	8	7	9	9	11	9	3.14	0.0202			3.82	0.00478	0.0443	up	1.22	0.571	1	3	1534	-1.75543663	-0.52801887	-1.65539508	-1.09999592	0.41227839	0.48920232	0.44764447	0.38939305	0.64321958	0.72538027	1.21316384	0.71856457
ENSMUSG00000109094	Gm44587	predicted gene	TEC	9	13	6	16	21	39	23	28	48	45	66	48	2.51	0.000154			4.63	2.56E-11	0.000000264	up	1.84	0.00065	1	3	1535	-1.23248954	-0.94020129	-1.57672952	-0.68653997	-0.33168381	0.59506069	-0.22976895	0.04389097	0.97414346	0.84254967	1.61186732	0.95690097
ENSMUSG00000028456	Unc13b	unc-13 homolo	protein_coding	660	799	690	834	909	1,022	946	945	1,208	985	1,255	1,121	1.28	0.000223	0.0307	up	1.53	1.4E-10	0.000000963	up	1.19	0.00661	1	3	1536	-1.67777127	-0.76262137	-1.46429262	-0.55841583	-0.12956824	0.45534149	0.06709425	0.06032247	1.30856032	0.26796375	1.50860614	0.9247809
ENSMUSG00000107306	Gm42577	predicted gene	TEC	100	129	102	127	140	152	129	139	180	160	191	181	1.22	0.0297	0.216		1.55	0.000000404	0.000159	up	1.27	0.00322	1	3	1537	-1.60266438	-0.45208539	-1.49567396	-0.5468664	-0.09453545	0.3175552	-0.4494075	-0.12120256	1.16581	0.58594336	1.48005036	1.21307673
ENSMUSG00000033257	Ttll4	tubulin tyrosine	protein_coding	515	700	624	707	844	885	789	753	942	861	1,031	908	1.28	0.00371	0.0845		1.47	0.00000685	0.000792	up	1.14	0.113	1	3	1538	-2.12314529	-0.59979737	-1.18155687	-0.54976556	0.37571703	0.62916734	0.01548655	-0.22307198	0.95942334	0.47917998	1.45304851	0.76531434
ENSMUSG00000062470	Fbxl12os	F-box and leuci	lncRNA	72	99	95	107	122	134	112	123	145	135	163	130	1.31	0.0244	0.197		1.53	0.000308	0.00791	up	1.16	0.176	1	3	1539	-2.11082208	-0.80277427	-0.98291816	-0.47006669	0.14688374	0.61760818	-0.26675487	0.18618562	0.9877835	0.63317555	1.62335536	0.43834352
ENSMUSG00000034771	Tle2	transducin-like	protein_coding	501	688	571	625	721	820	746	742	850	800	873	814	1.27	0.00398	0.087		1.4	0.0000453	0.00234	up	1.1	0.234	1	3	1540	-2.10295182	-0.28901473	-1.37073919	-0.85068469	-0.00972157	0.77352785	0.19325536	0.16658772	0.99126325	0.61735999	1.15838507	0.72273276
ENSMUSG00000070427	Il18bp	interleukin 18 b	protein_coding	104	170	117	156	204	208	180	187	253	212	251	240	1.42	0.000734	0.0478	up	1.74	4.11E-08	0.0000531	up	1.23	0.0342	1	3	1541	-1.93872667	-0.32044144	-1.57680753	-0.63332712	0.33554591	0.41170426	-0.11524841	0.02306456	1.18678445	0.48467037	1.16140774	0.98137388
ENSMUSG00000032582	Rbm6	RNA binding mo	protein_coding	1,602	1,745	1,612	1,933	2,085	2,263	1,721	1,885	1,937	1,968	2,291	2,179	1.15	0.0404	0.247		1.21	0.00493	0.0452	up	1.05	0.45	1	3	1542	-1.46975008	-0.78798935	-1.42124205	0.04325508	0.65888369	1.3356042	-0.89960146	-0.16402129	0.06008383	0.18702389	1.43392705	1.02382648
ENSMUSG00000113850	Gm47191	predicted gene	transcribed_processe	30	49	38	53	83	99	44	76	72	66	108	94	1.77	0.00737	0.114		1.99	0.000998	0.0168	up	1.13	0.551	1	3	1543	-1.64461414	-0.6735311	-1.2437768	-0.4926373	0.65027135	1.19013736	-0.94335257	0.39463778	0.25989674	0.0519974	1.44125743	1.00971385
ENSMUSG00000053483	Usp21	ubiquitin specif	protein_coding	607	672	682	673	753	815	701	716	684	768	821	816	1.13	0.0144	0.157		1.17	0.00125	0.0192	up	1.04	0.463	1	3	1544	-1.8118848	-0.76220934	-0.61522341	-0.76095492	0.43015361	1.27287291	-0.3344613	-0.10745534	-0.59095631	0.63781111	1.35976708	1.28254071
ENSMUSG00000078984	Gm11027	predicted gene	protein_coding	184	214	216	243	297	333	235	245	272	279	359	365	1.29	0.0291	0.215		1.49	0.000641	0.0126	up	1.15	0.221	1	3	1545	-1.61656168	-0.97482502	-0.93434804	-0.41648307	0.51695522	1.07790285	-0.56597024	-0.38505697	0.09828627	0.22404654	1.44349883	1.53255531
ENSMUSG00000102816	Gm37814	predicted gene	lncRNA	1	10	5	8	21	29	10	13	24	24	35	43	3.08	0.0026			5.35	0.00000335	0.000572	up	1.73	0.0628	1	3	1546	-1.79344725	-0.54865877	-1.18320654	-0.74102749	0.37294607	0.82999223	-0.50834477	-0.2534723	0.53178988	0.56675999	1.18811545	1.53855351
ENSMUSG00000083579	Gm15538	predicted gene	processed_pseudoge	0	1	5	4	4	9	4	7	8	9	14	13	2.5	0.0817			4.48	0.00253	0.0299	up	1.8	0.135	1	3	1547	-2.22865428	-1.09747902	-0.20107218	-0.32665521	-0.38072756	0.64985416	-0.36177047	0.36821958	0.4004299	0.60046034	1.34066021	1.23673453
ENSMUSG00000020703	5530401A14Rik	RIKEN cDNA 55	lncRNA	2	13	14	19	30	44	28	33	46	49	67	59	2.82	0.000187			4.62	1.06E-08	0.0000207	up	1.64	0.0281	1	3	1548	-2.06517199	-0.99880046	-0.89759873	-0.59876917	0.00094357	0.59512032	-0.09307404	0.12057097	0.65763074	0.7814156	1.37821292	1.11952029
ENSMUSG00000114345	Gm10873	predicted gene	lncRNA	1	8	8	9	15	18	14	18	18	24	24	23	2.48	0.0024			3.38	0.0000175	0.00129	up	1.37	0.146	1	3	1549	-2.33678464	-0.74706499	-0.89478906	-0.61061855	0.13489716	0.49121377	0.0183124	0.42372052	0.44211919	1.07550656	1.06993925	0.93354839
ENSMUSG00000038342	Mlxip	MLX interacting	protein_coding	748	776	842	871	895	1,015	889	841	940	909	965	1,087	1.12	0.0649	0.304		1.21	0.00254	0.03	up	1.07	0.246	1	3	1550	-1.66422602	-1.32886846	-0.57165239	-0.24795016	0.01122459	1.20735774	-0.05925347	-0.57329565	0.47084138	0.15646424	0.72449526	1.87486293
ENSMUSG00000053552	Ebf4	early B cell fact	protein_coding	447	490	489	567	559	630	545	551	583	633	640	658	1.14	0.00744	0.114		1.26	0.0000015	0.000341	up	1.1	0.0348	1	3	1551	-1.86260622	-1.1368853	-1.14314141	0.06309591	-0.05848815	0.94203559	-0.27305582	-0.18687793	0.28712422	0.97874708	1.0714517	1.31860033
ENSMUSG00000067629	Syngap1	synaptic Ras GT	protein_coding	3,427	3,520	3,768	4,083	4,107	4,571	4,234	4,071	4,226	4,442	4,657	4,616	1.15	0.00561	0.102		1.21	0.0000963	0.00375	up	1.06	0.262	1	3	1552	-1.82689459	-1.5641203	-0.89471887	-0.10263316	-0.04373833	1.01650181	0.25689809	-0.13056032	0.23718926	0.73273342	1.20362517	1.11571781
ENSMUSG00000060206	Zfp462	zinc finger prot	protein_coding	2,774	2,692	2,952	2,964	2,796	3,258	3,102	3,075	3,067	2,997	3,571	3,792	1.07	0.169	0.474		1.18	0.00147	0.0211	up	1.1	0.0714	1	3	1553	-1.01470415	-1.31022345	-0.39893223	-0.35981396	-0.93626391	0.58358691	0.09268722	0.0070233	-0.01953845	-0.24782517	1.49946984	2.10453415
ENSMUSG00000004113	Cacna1b	calcium channe	protein_coding	4,424	4,473	4,972	5,098	5,110	5,199	5,313	5,531	4,741	5,456	5,984	6,225	1.12	0.0382	0.24		1.18	0.00154	0.0218	up	1.06	0.275	1	3	1554	-1.52450397	-1.41947463	-0.40427462	-0.16362374	-0.14158938	0.0238325	0.23292673	0.62148553	-0.86187236	0.48933475	1.38258451	1.76517469
ENSMUSG00000027309	Dnaaf9	dynein axonem	protein_coding	3,910	3,823	3,945	4,385	4,251	4,454	4,682	4,682	4,462	4,732	4,599	5,360	1.13	0.0248	0.198		1.19	0.000769	0.0141	up	1.06	0.265	1	3	1555	-1.28359459	-1.51556984	-1.19108756	-0.08823675	-0.41295171	0.07342779	0.59669209	0.59689966	0.09281609	0.7070955	0.40820716	2.01630214
ENSMUSG00000104069	Gm37198	predicted gene	TEC	20	15	20	25	21	28	26	34	26	34	38	43	1.37	0.132			1.74	0.00503	0.0457	up	1.27	0.185	1	3	1556	-0.92681501	-1.61668517	-0.98261497	-0.18808034	-0.71916183	0.08378351	-0.15137733	0.88640156	-0.16389359	0.83506841	1.20932073	1.73405403
ENSMUSG00000029036	Atad3a	ATPase family,	protein_coding	610	727	668	660	724	755	735	738	702	829	842	865	1.11	0.14	0.435		1.22	0.00463	0.0436	up	1.1	0.179	1	3	1557	-1.76553502	-0.11299232	-0.91139752	-1.03226737	-0.1419601	0.26379685	0.00257459	0.0401423	-0.44027898	1.1742378	1.32785055	1.59582922
ENSMUSG00000097464	Gm26736	predicted gene	TEC	3	6	3	5	10	9	8	9	11	14	15	17	2.18	0.0601			3.44	0.0016	0.0223	up	1.58	0.153	1	3	1558	-1.62200293	-0.69359823	-1.49105753	-0.77136976	0.30257542	0.12472287	-0.19329757	0.15447662	0.49454516	1.01660621	1.2041928	1.47420693
ENSMUSG00000021819	Zswim8	zinc finger SWI	protein_coding	3,351	3,561	3,252	3,678	3,944	4,002	3,913	3,769	4,194	4,295	4,996	4,511	1.13	0.0259	0.203		1.3	0.00000129	0.000307	up	1.15	0.009	1	3	1559	-1.28861139	-0.79762541	-1.53081495	-0.53613149	0.03046718	0.14947905	-0.0341095	-0.33914689	0.5310643	0.72578227	1.96389307	1.12575375
ENSMUSG00000050266	Or52b1	olfactory recep	protein_coding	3	5	3	7	8	8	6	8	11	15	19	16	1.53	0.274			3.12	0.00144	0.0209	up	2.04	0.0141	1	3	1560	-1.34143276	-0.76894012	-1.43767587	-0.23418517	-0.08078227	-0.01235611	-0.56585545	-0.16003504	0.45800614	1.13488524	1.74100758	1.26736384
ENSMUSG00000010097	Nxf1	nuclear RNA ex	protein_coding	1,805	2,019	1,967	2,110	2,365	2,189	2,234	2,424	2,284	2,359	2,981	2,842	1.17	0.00614	0.105		1.32	0.000000482	0.000178	up	1.14	0.0219	1	3	1561	-1.60483358	-0.83465892	-1.01736699	-0.53305178	0.26022283	-0.27687729	-0.13712362	0.43308196	0.01878311	0.24489527	1.89220649	1.55472253
ENSMUSG00000048703	Speer4b	spermatogenes	protein_coding	8	19	15	21	24	19	21	27	25	29	36	29	1.43	0.112			1.88	0.0027	0.0312	up	1.32	0.151	1	3	1562	-2.25228561	-0.38111193	-1.05484235	-0.16852789	0.22116278	-0.42966634	-0.14054716	0.54264067	0.37292923	0.84915986	1.57658147	0.86450728
ENSMUSG00000104724	Gm43162	predicted gene	TEC	1	7	5	9	10	9	8	12	14	16	25	24	1.77	0.163			3.54	0.00104	0.0172	up	2	0.0423	1	3	1563	-1.94522811	-0.55284526	-1.04221629	-0.17954706	-0.07248515	-0.20467919	-0.44065326	0.21170678	0.40479667	0.71880206	1.63190316	1.47044565
ENSMUSG00000019763	Rmnd1	required for me	protein_coding	467	539	414	477	511	524	571	489	545	546	705	652	1.1	0.176	0.485		1.29	0.000425	0.00977	up	1.17	0.0303	1	3	1564	-0.8873511	0.07832151	-1.69676836	-0.74798495	-0.28652044	-0.11628949	0.49257745	-0.58932705	0.15993831	0.16842655	1.99817207	1.4268055
ENSMUSG00000034675	Dbn1	drebrin 1	protein_coding	3,719	3,915	3,843	4,347	4,484	4,510	4,547	4,203	4,203	4,394	5,117	5,188	1.12	0.0292	0.215		1.19	0.000672	0.013	up	1.07	0.224	1	3	1565	-1.53166745	-1.03197768	-1.21306701	-0.01170149	0.29168319	0.348348	0.42833481	-0.3421399	-0.34067155	0.0933389	1.58707894	1.72244122
ENSMUSG00000034818	Celf5	CUGBP, Elav-lik	protein_coding	4,079	4,224	4,327	4,654	4,836	4,884	5,241	4,639	4,786	5,016	5,675	5,326	1.13	0.00664	0.108		1.2	0.0000568	0.00269	up	1.06	0.192	1	3	1566	-1.63716183	-1.28022287	-1.03508312	-0.28960785	0.10319883	0.20448607	0.93135491	-0.32351388	-0.00187559	0.47988965	1.75102282	1.09751287
ENSMUSG00000091071	1700030C10Rik	RIKEN cDNA 17	transcribed_unproce	3	2	4	2	3	2	2	4	2	0	86	39	-1.08	0.929			9.87	0.00434	0.0417	up	10.6	0.0031	1	4	1567	-0.28345561	-0.36065055	-0.23806207	-0.49031958	-0.30259425	-0.49478157	-0.50026025	-0.20688517	-0.50122896	-0.72894152	2.62191926	1.48526528
ENSMUSG00000097099	Gm9917	predicted gene	lncRNA	97	59	48	67	58	86	75	66	119	79	98	124	1.05	0.743	0.908		1.56	0.00422	0.0409	up	1.48	0.0093	1	4	1568	0.69372508	-0.95752222	-1.52784464	-0.57886324	-0.97272285	0.28231825	-0.19645812	-0.62091818	1.47599663	-0.0021849	0.74772779	1.6567464
ENSMUSG00000083084	Gm14667	predicted gene	processed_pseudoge	197	124	122	129	148	126	120	170	214	217	320	380	-1.01	0.949	0.985		1.98	0.000165	0.00533	up	2	0.000118	0.627	4	1569	0.22476534	-0.84837938	-0.88228965	-0.7535781	-0.44955359	-0.8139968	-0.90543405	-0.13784944	0.4477157	0.48192945	1.55898612	2.07768439
ENSMUSG00000104916	Gm42830	predicted gene	TEC	643	484	485	652	708	603	552	657	1,028	1,015	1,477	1,747	1.11	0.55	0.807		2.33	0.00000237	0.000467	up	2.09	0.0000366	0.36	4	1570	-0.43369503	-1.05103288	-1.04744692	-0.40092723	-0.21379573	-0.57439104	-0.77156273	-0.38352574	0.66677295	0.6346138	1.57102756	2.00396299
ENSMUSG00000062861	Zfp28	zinc finger prot	protein_coding	361	521	493	469	425	528	416	482	665	653	547	486	1.01	0.933	0.98		1.28	0.00396	0.0394	up	1.27	0.00486	1	4	1571	-1.76927755	0.25312468	-0.06482059	-0.34878506	-0.88565996	0.33471549	-1.00934474	-0.18861403	1.7006676	1.58967225	0.53637943	-0.14805754
ENSMUSG00000035270	Impg2	interphotorece	protein_coding	26	38	33	101	40	98	44	35	141	129	47	111	1.11	0.699	0.887		2.24	0.00264	0.0307	up	2.01	0.00824	1	4	1572	-1.21603361	-0.74574819	-0.93994495	0.82585557	-0.69158048	0.76272103	-0.55237659	-0.8444445	1.52930964	1.32523681	-0.46022301	1.00722828
ENSMUSG00000111374	Gm50490	predicted gene	processed_pseudoge	85	107	77	136	90	120	92	114	133	142	121	134	1.03	0.774	0.92		1.31	0.00571	0.0496	up	1.27	0.011	1	4	1573	-1.26510412	-0.19667521	-1.70610404	1.03196722	-0.9824649	0.402152	-0.89394077	0.10566674	0.90286847	1.26202009	0.40271711	0.93689741
ENSMUSG00000023942	Slc29a1	solute carrier fa	protein_coding	548	526	552	534	626	604	471	504	669	680	583	613	1.02	0.806	0.933		1.18	0.00531	0.0473	up	1.16	0.0108	1	4	1574	-0.39871729	-0.75404283	-0.33150655	-0.63253923	0.79085514	0.46442595	-1.70322976	-1.13453694	1.39355079	1.55285379	0.15692763	0.5959593
ENSMUSG00000111063	Zfp660	zinc finger prot	lncRNA	266	296	251	306	265	366	289	252	332	372	422	437	1.04	0.687	0.88		1.4	0.000858	0.0152	up	1.34	0.00302	1	4	1575	-0.86961775	-0.3501434	-1.15397801	-0.17614103	-0.89439301	0.74448412	-0.45968336	-1.12208205	0.23733681	0.82668589	1.5141414	1.70339038
ENSMUSG00000003233	Dvl3	dishevelled seg	protein_coding	1,438	1,288	1,214	1,517	1,483	1,531	1,236	1,320	1,526	1,575	1,633	1,782	1.02	0.738	0.906		1.19	0.00211	0.0267	up	1.17	0.00588	1	4	1576	-0.09525286	-1.02452036	-1.52035599	0.36366926	0.17152444	0.44293933	-1.36954475	-0.82115382	0.41158708	0.6853664	0.9998502	1.75589108
ENSMUSG00000045103	Dmd	dystrophin, mu	protein_coding	2,541	2,711	2,294	2,737	3,052	2,991	2,294	2,293	3,076	3,262	3,136	3,191	1.03	0.602	0.834		1.23	0.000544	0.0115	up	1.19	0.00329	1	4	1577	-0.65255442	-0.17651491	-1.39769408	-0.10549192	0.69984061	0.54882789	-1.3966564	-1.40181779	0.75772735	1.1949355	0.90029216	1.02910603
ENSMUSG00000081752	Sms-ps	spermine synth	processed_pseudoge	0	4	0	0	0	0	0	0	0	0	0	0	-123000	0.00133			-238000	0.000696	0.0132	down	-1	1	1	5	1578	-0.28867513	3.17542648	-0.28867513	-0.28867513	-0.28867513	-0.28867513	-0.28867513	-0.28867513	-0.28867513	-0.28867513	-0.28867513	-0.28867513
ENSMUSG00000022313	Utp23	UTP23 small su	protein_coding	233	297	264	325	230	169	250	254	209	239	226	201	-1.24	0.0176	0.172		-1.27	0.0051	0.0461	down	-1.03	0.721	1	5	1579	-0.14718967	1.28844002	0.59100747	1.86249409	-0.23280056	-1.90261109	0.26479964	0.34024117	-0.75414443	0.00465822	-0.33585318	-0.97904168
ENSMUSG00000020018	Snrpf	small nuclear ri	protein_coding	255	330	293	283	241	228	259	251	234	251	224	248	-1.18	0.0124	0.146		-1.21	0.00333	0.0357	down	-1.02	0.74	1	5	1580	-0.07648624	2.29143966	1.18158478	0.84091433	-0.55247888	-1.03857426	0.05710636	-0.19345384	-0.81864416	-0.20425691	-1.18962012	-0.29753072
ENSMUSG00000023845	Lnpep	leucyl/cystinyl a	protein_coding	6,271	7,044	6,550	6,669	5,727	5,746	5,806	6,024	6,266	6,198	5,641	5,451	-1.14	0.00163	0.0616		-1.13	0.00369	0.0379	down	1.01	0.793	1	5	1581	0.3608047	1.884327	0.93155968	1.16584267	-0.82494625	-0.77982678	-0.64617418	-0.16428501	0.35126999	0.20846053	-1.20054643	-1.46648593
ENSMUSG00000025226	Fbxl15	F-box and leuci	protein_coding	189	221	207	188	156	156	166	173	186	154	146	146	-1.23	0.0161	0.165		-1.27	0.00393	0.0393	down	-1.03	0.696	1	5	1582	0.64599001	1.85460901	1.32160031	0.62495514	-0.72778273	-0.72525258	-0.31443809	0.0085462	0.53541886	-0.8154714	-1.20287799	-1.20529674
ENSMUSG00000078377	Gm4294	predicted gene	processed_pseudoge	0	1	0	140	0	0	0	0	0	0	0	0	-3540000	0.0000252	0.0191	down	-6020000	0.0000129	0.00108	down	-1	1	1	5	1583	-0.32030748	0.04522969	-0.32030748	3.15784507	-0.32030748	-0.32030748	-0.32030748	-0.32030748	-0.32030748	-0.32030748	-0.32030748	-0.32030748
ENSMUSG00000038763	Alpk3	alpha-kinase 3	protein_coding	39	21	46	59	17	16	22	21	17	22	23	20	-2.03	0.00115			-1.92	0.00163	0.0226	down	1.05	0.787	1	5	1584	0.99552835	-0.43422462	1.47164864	2.2222597	-0.80067558	-0.90313756	-0.31276454	-0.37557809	-0.76191459	-0.33556991	-0.25164883	-0.51392298
ENSMUSG00000021250	Fos	FBJ osteosarcor	protein_coding	332	333	267	414	144	129	238	268	136	134	232	238	-1.73	0.00182	0.0652		-1.82	0.000587	0.0119	down	-1.06	0.758	1	5	1585	1.01163832	1.0223279	0.41748987	1.66201668	-1.07689507	-1.30242081	0.11386983	0.41854874	-1.19756556	-1.2254276	0.04205188	0.11436583
ENSMUSG00000022553	Maf1	MAF1 homolog	protein_coding	960	922	977	959	817	791	858	850	820	807	848	890	-1.15	0.00189	0.0665		-1.14	0.00387	0.039	down	1.01	0.763	1	5	1586	1.28989576	0.74620031	1.5304055	1.28398703	-0.89277616	-1.32365578	-0.23368026	-0.3605967	-0.84014202	-1.05976641	-0.3986563	0.25878503
ENSMUSG00000000184	Ccnd2	cyclin D2	protein_coding	910	415	2,253	378	332	390	398	449	377	348	276	401	-2.48	0.000224	0.0307	down	-2.77	0.0000323	0.00193	down	-1.12	0.645	1	5	1587	1.08070185	-0.2328555	2.81964907	-0.3738541	-0.56777256	-0.3276813	-0.29681313	-0.11132692	-0.37857221	-0.4991716	-0.8266539	-0.28564971
ENSMUSG00000020458	Rtn4	reticulon 4	protein_coding	16,031	14,616	16,427	14,848	14,448	13,981	14,277	14,126	14,075	13,634	14,362	14,170	-1.09	0.00722	0.112		-1.1	0.00248	0.0296	down	-1.01	0.741	1	5	1588	1.7386253	0.06631989	2.180344	0.35149764	-0.14267399	-0.73650471	-0.35771473	-0.54959737	-0.61534087	-1.19033496	-0.25034755	-0.49427264
ENSMUSG00000015478	Rnf5	ring finger prot	protein_coding	1,133	922	1,149	989	924	898	980	945	908	907	921	943	-1.12	0.00493	0.0949		-1.14	0.000763	0.014	down	-1.02	0.637	1	5	1589	1.92224879	-0.53992493	2.09193724	0.28187205	-0.51893863	-0.8600664	0.17406392	-0.25913907	-0.72472429	-0.7314779	-0.56234853	-0.27350225
ENSMUSG00000052214	Opa3	optic atrophy 3	protein_coding	1,187	1,029	1,262	1,033	978	942	1,054	1,019	947	961	985	1,024	-1.13	0.0186	0.177		-1.15	0.00542	0.048	down	-1.02	0.696	1	5	1590	1.58437763	-0.02851178	2.28334944	0.01704143	-0.59663176	-1.01373573	0.23959616	-0.13512901	-0.95600963	-0.79352998	-0.51433494	-0.08648184
ENSMUSG00000030600	Lrfn1	leucine rich rep	protein_coding	1,701	1,509	1,587	1,412	1,409	1,399	1,472	1,405	1,417	1,417	1,412	1,397	-1.09	0.00708	0.111		-1.1	0.00274	0.0314	down	-1.01	0.819	1	5	1591	2.49135069	0.54728134	1.35772336	-0.52707962	-0.5640245	-0.68050186	0.14421915	-0.60413781	-0.46955086	-0.47256688	-0.52730266	-0.69541035
ENSMUSG00000044628	Rnf208	ring finger prot	protein_coding	2,381	2,078	2,177	1,908	1,834	1,729	1,987	1,858	1,846	1,962	1,771	1,884	-1.15	0.000487	0.0386	down	-1.14	0.000823	0.0147	down	1.01	0.845	1	5	1592	2.24367752	0.72860726	1.24480264	-0.20600688	-0.63874587	-1.27906255	0.23921025	-0.49807229	-0.57142377	0.10050501	-1.01908427	-0.34440705
ENSMUSG00000003072	Atp5d	ATP synthase, H	protein_coding	2,414	2,232	2,283	1,850	2,012	1,827	2,002	1,874	1,915	1,985	1,864	1,885	-1.14	0.00717	0.112		-1.15	0.00382	0.0387	down	-1.01	0.86	1	5	1593	2.02154126	1.16426202	1.4111229	-0.86389048	0.04162493	-0.99624811	-0.01211939	-0.72158228	-0.49001472	-0.10828881	-0.78374928	-0.66265804
ENSMUSG00000025228	Actr1a	ARP1 actin-rela	protein_coding	4,685	4,374	4,673	3,959	3,998	3,908	4,194	3,949	4,024	4,046	3,978	3,789	-1.1	0.00834	0.119		-1.12	0.00251	0.0298	down	-1.01	0.716	1	5	1594	1.85489321	0.85588436	1.8163776	-0.58362407	-0.44210691	-0.77216345	0.24818347	-0.62177216	-0.3493931	-0.27256723	-0.51482514	-1.21888661
ENSMUSG00000032997	Chpf	chondroitin pol	protein_coding	2,123	1,899	2,181	1,736	1,758	1,624	1,860	1,679	1,734	1,727	1,669	1,664	-1.15	0.00191	0.0666		-1.17	0.000371	0.0089	down	-1.02	0.676	1	5	1595	1.75034132	0.57224045	2.0350147	-0.36054465	-0.22904533	-1.05023625	0.35613117	-0.71113828	-0.37413288	-0.41663439	-0.77152887	-0.80046698
ENSMUSG00000003469	Phyhip	phytanoyl-CoA	protein_coding	5,048	4,836	5,045	4,485	4,557	4,022	4,581	4,368	4,250	4,442	4,293	4,421	-1.11	0.00632	0.107		-1.12	0.00333	0.0357	down	-1.01	0.852	1	5	1596	1.62408341	0.99258237	1.61416186	-0.11394798	0.12118427	-1.70479585	0.19843808	-0.5007426	-0.89978457	-0.25320614	-0.75434308	-0.32362978
ENSMUSG00000039615	Stub1	STIP1 homolog	protein_coding	3,126	2,898	3,088	2,752	2,753	2,588	2,840	2,728	2,632	2,723	2,606	2,809	-1.09	0.0175	0.172		-1.1	0.00567	0.0494	down	-1.01	0.719	1	5	1597	1.88112381	0.62044525	1.67709862	-0.23281295	-0.22516551	-1.24471172	0.29033096	-0.37303539	-0.9662709	-0.40507084	-1.12990482	0.1079735
ENSMUSG00000024121	Atp6v0c	ATPase, H+ tran	protein_coding	14,699	12,702	13,484	12,229	12,399	11,419	12,482	12,023	11,514	11,813	11,854	12,499	-1.1	0.00721	0.112		-1.11	0.00209	0.0266	down	-1.01	0.702	1	5	1598	2.42128834	0.34450882	1.19390779	-0.1951926	0.00160553	-1.16769356	0.09604472	-0.43597178	-1.04951177	-0.6865135	-0.63746028	0.11498827
ENSMUSG00000058756	Thra	thyroid hormor	protein_coding	14,976	13,035	14,090	13,131	12,453	11,952	12,887	12,653	12,022	12,365	12,482	12,371	-1.11	0.00222	0.0702		-1.12	0.000455	0.0102	down	-1.01	0.662	1	5	1599	2.34511201	0.22677116	1.41456774	0.33926975	-0.4692348	-1.09487447	0.05340292	-0.22691822	-1.00623062	-0.57781642	-0.43414846	-0.5699006
ENSMUSG00000079144	A130010J15Rik	RIKEN cDNA A1	protein_coding	215	188	224	185	131	148	190	179	162	191	140	146	-1.25	0.0105	0.136		-1.27	0.0055	0.0485	down	-1.01	0.89	1	5	1600	1.31740042	0.48674831	1.56291204	0.37076912	-1.55712278	-0.88905701	0.54574764	0.18784463	-0.40038088	0.56320006	-1.21210456	-0.97595697
ENSMUSG00000062825	Actg1	actin, gamma, c	protein_coding	20,336	19,850	20,579	18,350	17,622	17,249	18,398	18,292	18,863	19,191	15,707	17,001	-1.11	0.0097	0.129		-1.12	0.00398	0.0396	down	-1.01	0.773	1	5	1601	1.27169895	0.96346574	1.42264932	-0.03568405	-0.55065377	-0.82233749	-0.00253451	-0.07619596	0.31434807	0.53372526	-2.01160896	-1.00687259
ENSMUSG00000018846	Pank3	pantothenate k	protein_coding	2,738	2,735	2,772	2,696	2,403	2,389	2,542	2,505	2,577	2,608	2,374	2,281	-1.11	0.000743	0.0481	down	-1.11	0.000584	0.0119	down	-1	0.999	1	5	1602	1.12000103	1.09748252	1.30611527	0.87875616	-0.89681767	-0.98479312	-0.02906204	-0.25924638	0.1812864	0.36627617	-1.08209563	-1.69790273
ENSMUSG00000019124	Scrn1	secernin 1	protein_coding	11,091	10,936	11,466	10,841	9,900	9,507	9,923	10,059	10,074	10,427	9,592	9,610	-1.13	0.0000498	0.0204	down	-1.12	0.000141	0.00481	down	1.01	0.786	1	5	1603	1.2271927	1.00419858	1.75750904	0.86473874	-0.57868047	-1.2212297	-0.54125357	-0.32529691	-0.30153057	0.24601961	-1.08126491	-1.05040253
ENSMUSG00000067787	Blcap	bladder cancer	protein_coding	4,136	3,908	4,213	3,975	3,866	3,720	3,934	3,779	3,852	3,837	3,671	3,803	-1.06	0.0163	0.167		-1.07	0.00519	0.0466	down	-1.01	0.727	1	5	1604	1.53704144	0.12590053	1.9961685	0.54987543	-0.14140522	-1.10300209	0.28840154	-0.70858031	-0.23401496	-0.32888955	-1.4304278	-0.55106752
ENSMUSG00000073062	Zxdb	zinc finger, X-lir	protein_coding	846	790	876	727	756	682	754	652	701	761	675	671	-1.14	0.0106	0.136		-1.15	0.00486	0.0448	down	-1.01	0.841	1	5	1605	1.46970089	0.72689858	1.85028922	-0.16595444	0.24778651	-0.83534516	0.21582232	-1.31332069	-0.54801356	0.31567996	-0.94881738	-1.01472626
ENSMUSG00000030410	Dmwd	dystrophia myo	protein_coding	2,646	2,532	2,664	2,315	2,281	2,273	2,391	2,270	2,397	2,348	2,198	2,191	-1.1	0.00406	0.0878		-1.11	0.00151	0.0215	down	-1.01	0.801	1	5	1606	1.66675028	0.99635953	1.76983762	-0.36097612	-0.58428703	-0.64063956	0.12738501	-0.65723576	0.16376204	-0.15021484	-1.14271673	-1.18802443
ENSMUSG00000020496	Rnf187	ring finger prot	protein_coding	6,617	6,393	7,116	5,800	5,654	5,399	6,022	5,729	5,682	5,849	5,436	5,390	-1.14	0.0023	0.0714		-1.16	0.000408	0.00949	down	-1.02	0.636	1	5	1607	1.31852777	0.92038606	2.16266572	-0.20503893	-0.49955423	-1.02953165	0.22933265	-0.34612249	-0.44253971	-0.10758519	-0.95112826	-1.04941174
ENSMUSG00000021395	Spin1	spindlin 1	protein_coding	5,940	5,895	6,333	5,449	5,155	5,186	5,471	5,305	5,413	5,371	5,097	5,016	-1.12	0.00267	0.0759		-1.13	0.000944	0.0162	down	-1.01	0.775	1	5	1608	1.21014659	1.10139848	2.1273524	-0.0209629	-0.81049006	-0.72458558	0.03652581	-0.40278709	-0.11695414	-0.22712049	-0.97286214	-1.19966087
ENSMUSG00000033953	Ppp3r1	protein phosph	protein_coding	11,029	10,551	11,469	10,316	9,707	9,562	9,932	9,789	9,840	9,850	9,397	9,510	-1.11	0.00342	0.0817		-1.12	0.0013	0.0197	down	-1.01	0.78	1	5	1609	1.48516528	0.76839904	2.11869853	0.40399839	-0.57973032	-0.82241665	-0.20993197	-0.44474377	-0.35982066	-0.34441899	-1.10459277	-0.9106061
ENSMUSG00000034723	Tmx4	thioredoxin-rel	protein_coding	18,522	17,964	18,595	17,490	16,637	16,390	17,176	16,818	16,721	16,935	16,214	16,334	-1.08	0.0166	0.169		-1.1	0.0056	0.049	down	-1.01	0.711	1	5	1610	1.6476526	1.00082426	1.73031157	0.43670881	-0.61842101	-0.93474737	0.05408354	-0.39008197	-0.51314129	-0.24396292	-1.1621041	-1.00712213
ENSMUSG00000030327	Necap1	NECAP endocyt	protein_coding	5,298	5,010	5,303	4,819	4,379	4,353	4,682	4,601	4,752	4,736	4,341	4,256	-1.13	0.00201	0.0681		-1.13	0.00259	0.0303	down	1	0.924	1	5	1611	1.60883443	0.85920945	1.62184513	0.33781923	-0.94324673	-1.02341695	-0.048494	-0.28391499	0.14936843	0.10533452	-1.05882751	-1.32451101
ENSMUSG00000047388	Atmin	ATM interactor	protein_coding	3,311	3,128	3,412	3,047	2,770	2,778	2,931	2,968	2,932	2,914	2,754	2,707	-1.13	0.000636	0.0453	down	-1.14	0.000129	0.00452	down	-1.01	0.711	1	5	1612	1.51843862	0.73837861	1.93250314	0.37706966	-0.92353916	-0.88310528	-0.15510131	0.01663599	-0.14834986	-0.23181715	-1.00177659	-1.23933667
ENSMUSG00000030842	Lamtor1	late endosomal	protein_coding	1,227	1,166	1,340	1,173	1,047	1,020	1,118	1,091	1,083	1,116	1,078	1,075	-1.15	0.000234	0.0316	down	-1.13	0.00089	0.0155	down	1.02	0.63	1	5	1613	1.15217819	0.46661007	2.34064755	0.54644101	-0.95241067	-1.28576181	-0.07999513	-0.40794436	-0.50138901	-0.11283551	-0.55953813	-0.6060022
ENSMUSG00000004637	Wwox	WW domain-co	protein_coding	844	823	879	814	704	696	742	706	740	740	705	713	-1.18	0.0000249	0.0191	down	-1.16	0.0000878	0.0035	down	1.02	0.625	1	5	1614	1.33755085	1.02463224	1.84703055	0.88924245	-0.87899368	-1.00719562	-0.24712686	-0.83454471	-0.27120441	-0.27461409	-0.86123473	-0.723542
ENSMUSG00000044147	Arf6	ADP-ribosylatio	protein_coding	843	884	920	825	704	684	775	720	732	720	724	682	-1.2	0.0000912	0.0249	down	-1.22	0.0000235	0.00159	down	-1.01	0.837	1	5	1615	0.95678656	1.43081944	1.82424621	0.74716097	-0.79690432	-1.07465364	0.13263634	-0.58107942	-0.42582402	-0.58488486	-0.53405641	-1.09424686
ENSMUSG00000026159	Agfg1	ArfGAP with FG	protein_coding	2,977	3,231	3,129	2,822	2,696	2,804	2,848	2,836	2,893	2,866	2,720	2,716	-1.09	0.00176	0.0645		-1.08	0.0016	0.0223	down	1	0.964	1	5	1616	0.63283032	2.1166716	1.53502803	-0.32988603	-1.1530825	-0.44753619	-0.16440488	-0.24274371	0.11458846	-0.05044647	-0.99007276	-1.02094588
ENSMUSG00000028833	Ncdn	neurochondrin	protein_coding	17,733	17,535	18,169	15,896	15,061	14,858	15,531	15,431	15,540	15,579	15,429	14,789	-1.14	0.0000183	0.0191	down	-1.13	0.0000503	0.00249	down	1.01	0.807	1	5	1617	1.51636724	1.35744591	1.85934737	-0.02621157	-0.7869232	-0.97720157	-0.35374483	-0.44430162	-0.3450427	-0.30982279	-0.44626138	-1.04365086
ENSMUSG00000002379	Ndufa11	NADH:ubiquino	protein_coding	997	1,021	1,058	819	753	757	865	822	827	858	817	743	-1.22	0.00309	0.08		-1.2	0.0055	0.0485	down	1.01	0.826	1	5	1618	1.27508786	1.47982318	1.78111627	-0.37261206	-1.05531587	-1.01701967	0.08466446	-0.34427898	-0.28577633	0.01411431	-0.39196963	-1.16783354
ENSMUSG00000014956	Ppp1cb	protein phosph	protein_coding	7,919	8,056	8,318	7,096	6,776	6,690	7,399	7,156	7,064	6,998	6,931	6,589	-1.12	0.00329	0.0808		-1.14	0.000764	0.014	down	-1.02	0.679	1	5	1619	1.20432099	1.43100483	1.85560578	-0.24811102	-0.85733427	-1.02583789	0.30353274	-0.13746772	-0.30887894	-0.43176921	-0.55820538	-1.22685991
ENSMUSG00000028373	Astn2	astrotactin 2	protein_coding	2,249	2,400	2,394	2,157	2,146	2,077	2,174	2,114	2,147	2,142	2,110	2,032	-1.08	0.0128	0.149		-1.09	0.00426	0.0412	down	-1.01	0.752	1	5	1620	0.64386373	1.90475343	1.85790697	-0.16861636	-0.27300337	-0.89701958	-0.01505136	-0.56303798	-0.26090087	-0.30264595	-0.59856292	-1.32768573
ENSMUSG00000037049	Smpd1	sphingomyelin	protein_coding	2,531	2,648	2,634	2,194	2,140	2,062	2,376	2,199	2,313	2,240	2,115	2,023	-1.14	0.00924	0.126		-1.15	0.00473	0.0441	down	-1.01	0.84	1	5	1621	1.13053944	1.62587695	1.56825039	-0.42396578	-0.69021684	-1.09051276	0.44163907	-0.3964735	0.14989889	-0.19597111	-0.8194254	-1.29963936
ENSMUSG00000063873	Slc24a3	solute carrier fa	protein_coding	5,183	5,177	5,318	4,824	4,543	4,853	4,759	4,729	4,719	4,669	4,573	4,689	-1.09	0.000639	0.0453	down	-1.1	0.000066	0.00292	down	-1.01	0.602	1	5	1622	1.37212492	1.34785727	1.87182745	-0.02798553	-1.19099738	0.0917489	-0.29067916	-0.4137015	-0.45464716	-0.66298552	-1.06442471	-0.57813759
ENSMUSG00000067279	Ppp1r3c	protein phosph	protein_coding	1,345	1,357	1,439	1,254	1,118	1,137	1,161	1,178	1,142	1,106	1,109	1,195	-1.17	0.00087	0.0504		-1.18	0.000337	0.00835	down	-1.01	0.842	1	5	1623	1.21120715	1.30810256	1.98106641	0.41813474	-0.86005299	-0.67123684	-0.44043542	-0.27536321	-0.62207898	-0.98075937	-0.94812697	-0.12045707
ENSMUSG00000022619	Mapk8ip2	mitogen-activat	protein_coding	9,343	9,690	9,525	8,674	8,242	8,076	9,020	8,795	8,485	8,512	8,202	8,520	-1.09	0.0118	0.143		-1.1	0.00391	0.0391	down	-1.01	0.721	1	5	1624	1.10834445	1.71711111	1.43020879	-0.13153963	-0.98322099	-1.32210151	0.52138563	0.09887646	-0.49822619	-0.4460988	-1.06332094	-0.43141839
ENSMUSG00000024261	Syt4	synaptotagmin	protein_coding	8,963	8,798	8,942	8,305	7,678	7,837	8,451	7,962	7,959	7,856	8,155	7,774	-1.1	0.00551	0.101		-1.1	0.00299	0.0333	down	-1.01	0.858	1	5	1625	1.56860586	1.23663929	1.5276205	0.20229692	-1.20323105	-0.83525691	0.51440066	-0.55247246	-0.56083168	-0.79361207	-0.12402483	-0.98013424
ENSMUSG00000027327	1700037H04Rik	RIKEN cDNA 17	protein_coding	1,427	1,470	1,571	1,233	1,136	1,223	1,258	1,239	1,154	1,226	1,222	1,185	-1.17	0.00298	0.079		-1.19	0.00106	0.0174	down	-1.01	0.788	1	5	1626	1.12903729	1.42919465	2.09303297	-0.31213623	-1.11617443	-0.39838517	-0.1166516	-0.27192705	-0.95889337	-0.37029417	-0.40634223	-0.70046067
ENSMUSG00000032875	Arhgef17	Rho guanine nu	protein_coding	7,213	7,309	7,534	6,662	6,463	6,425	6,957	6,542	6,498	6,574	6,493	6,648	-1.09	0.0106	0.136		-1.1	0.00563	0.0492	down	-1.01	0.843	1	5	1627	1.16456998	1.40618703	1.96126027	-0.28543403	-0.83884406	-0.9467308	0.50487256	-0.61667818	-0.74061456	-0.52813669	-0.7548504	-0.32560112
ENSMUSG00000028953	Abcf2	ATP-binding cas	protein_coding	2,153	2,191	2,191	2,017	1,931	1,950	1,962	1,934	1,912	1,919	2,054	1,820	-1.1	0.00765	0.116		-1.11	0.00264	0.0307	down	-1.01	0.77	1	5	1628	1.24409134	1.54056772	1.5431029	0.14156957	-0.58677434	-0.42017721	-0.32293745	-0.56507	-0.75277023	-0.69106233	0.4471388	-1.57767879
ENSMUSG00000046541	Zfp526	zinc finger prot	protein_coding	305	339	358	317	289	286	276	288	274	271	317	269	-1.16	0.0111	0.138		-1.17	0.00405	0.0399	down	-1.01	0.836	1	5	1629	0.25628126	1.38955522	1.99112712	0.67423657	-0.32644252	-0.45992017	-0.83603063	-0.37676959	-0.89138124	-0.9983651	0.66772371	-1.09001463
ENSMUSG00000026080	Chst10	carbohydrate su	protein_coding	2,126	2,202	2,422	2,097	2,045	1,857	2,033	2,020	1,952	2,011	1,988	1,900	-1.11	0.0134	0.152		-1.13	0.00475	0.0442	down	-1.01	0.747	1	5	1630	0.51868833	1.01927397	2.38193938	0.32319381	-0.03769514	-1.39683658	-0.11735874	-0.2101583	-0.69407109	-0.27354956	-0.43918565	-1.07424043
ENSMUSG00000042229	Rabif	RAB interacting	protein_coding	793	810	900	718	709	656	744	700	710	701	711	643	-1.14	0.0116	0.142		-1.16	0.00368	0.0378	down	-1.02	0.744	1	5	1631	0.88127823	1.11211951	2.27033893	-0.18777914	-0.31362872	-1.12911379	0.20466109	-0.45809848	-0.30432479	-0.44174408	-0.29264822	-1.34106053
ENSMUSG00000029557	Mrm2	mitochondrial r	protein_coding	263	264	277	229	226	190	223	227	208	205	221	226	-1.19	0.00736	0.114		-1.2	0.00256	0.0301	down	-1.01	0.845	1	5	1632	1.26761012	1.31917452	1.74881802	0.01186413	-0.10917382	-1.63160559	-0.24162618	-0.06954684	-0.86938025	-0.99229516	-0.32317957	-0.11065938
ENSMUSG00000031513	Leprotl1	leptin receptor	protein_coding	1,907	1,874	1,958	1,679	1,662	1,513	1,699	1,731	1,643	1,617	1,731	1,534	-1.12	0.00615	0.105		-1.14	0.00183	0.0242	down	-1.01	0.737	1	5	1633	1.37382135	1.15686935	1.7040089	-0.2080534	-0.33436054	-1.49036727	-0.0627323	0.16519367	-0.4751927	-0.6741595	0.16940535	-1.3244329
ENSMUSG00000031988	Vps26b	VPS26 retromer	protein_coding	2,637	2,576	2,700	2,486	2,361	2,230	2,491	2,407	2,358	2,333	2,409	2,326	-1.1	0.00161	0.0616		-1.1	0.000484	0.0106	down	-1.01	0.792	1	5	1634	1.38432383	0.96842514	1.80276156	0.33582528	-0.58127894	-1.58590146	0.36692455	-0.23795864	-0.59868514	-0.78797547	-0.22432311	-0.8421376
ENSMUSG00000001729	Akt1	thymoma viral	protein_coding	4,498	3,946	4,319	4,219	3,870	3,730	3,692	4,110	3,965	3,789	3,526	4,105	-1.1	0.00424	0.0896		-1.1	0.0034	0.036	down	-1	0.967	1	5	1635	1.76699038	-0.09327297	1.18771136	0.85670073	-0.36768702	-0.89075994	-1.03326646	0.48348395	-0.02539944	-0.6670901	-1.68288142	0.46547094
ENSMUSG00000038602	Slc35f1	solute carrier fa	protein_coding	2,392	2,238	2,214	2,356	2,101	2,075	1,980	2,221	2,135	2,053	2,072	2,106	-1.1	0.00644	0.107		-1.1	0.00442	0.0423	down	-1	0.943	1	5	1636	1.80567894	0.63395357	0.44528873	1.5378685	-0.4756549	-0.69572697	-1.51531533	0.49514395	-0.19219266	-0.88007609	-0.72019304	-0.4387747
ENSMUSG00000070509	Rgma	repulsive guida	protein_coding	2,663	2,402	2,499	2,384	2,189	2,344	2,048	2,334	2,261	2,215	2,149	2,303	-1.12	0.00216	0.0697		-1.11	0.00209	0.0266	down	1	0.968	1	5	1637	2.03223952	0.54962013	1.11565371	0.4440682	-0.77228978	0.19969092	-1.71598936	0.13935135	-0.30963222	-0.60263353	-1.03165398	-0.04842498
ENSMUSG00000028525	Pde4b	phosphodiester	protein_coding	3,207	3,349	3,418	3,358	2,959	2,975	2,822	2,939	3,039	2,864	2,807	3,013	-1.14	0.000206	0.0307	down	-1.14	0.000232	0.00651	down	1	0.937	1	5	1638	0.69410592	1.31724556	1.60829966	1.35502726	-0.46391252	-0.38302365	-1.13902844	-0.56035916	-0.08203291	-0.93133147	-1.21337944	-0.20161081
ENSMUSG00000024892	Pcx	pyruvate carbo	protein_coding	2,514	2,499	2,494	2,596	2,326	2,205	2,087	2,196	2,288	2,383	2,099	2,175	-1.15	0.000436	0.0378	down	-1.13	0.00154	0.0218	down	1.02	0.688	1	5	1639	1.09499359	1.0175572	0.99103535	1.52724269	0.05618653	-0.65806407	-1.38801704	-0.70857323	-0.16627339	0.38024557	-1.30818352	-0.83814966
ENSMUSG00000029924	Slc37a3	solute carrier fa	protein_coding	1,282	1,129	1,215	1,259	1,118	1,068	1,022	1,014	1,061	1,095	1,062	1,087	-1.16	0.00039	0.0377	down	-1.13	0.00191	0.0249	down	1.02	0.597	1	5	1640	1.81554994	0.16594155	1.10975202	1.5752633	0.03085107	-0.5564115	-1.10887234	-1.21321456	-0.63639059	-0.23635408	-0.61931398	-0.32680084
ENSMUSG00000019785	Clvs2	clavesin 2	protein_coding	827	761	850	776	706	565	661	814	715	703	644	623	-1.17	0.0135	0.152		-1.2	0.00364	0.0377	down	-1.02	0.689	1	5	1641	1.17347333	0.4941253	1.4013699	0.65049489	-0.12126794	-1.86872185	-0.63710245	1.04300999	-0.01854267	-0.15016198	-0.85214769	-1.11452884
ENSMUSG00000034118	Tpst1	protein-tyrosin	protein_coding	959	852	853	879	815	760	816	827	810	787	778	794	-1.1	0.0216	0.189		-1.12	0.00561	0.049	down	-1.02	0.686	1	5	1642	2.39163102	0.49316516	0.51460786	0.98293469	-0.22338587	-1.31120718	-0.19828707	0.01860907	-0.31185947	-0.77031418	-0.94932403	-0.63657001
ENSMUSG00000026869	Psmd5	proteasome (pr	protein_coding	1,815	1,736	1,847	1,661	1,539	1,415	1,554	1,633	1,455	1,584	1,487	1,517	-1.15	0.00523	0.0981		-1.17	0.00158	0.0221	down	-1.02	0.736	1	5	1643	1.49801042	0.97035904	1.70593425	0.44753272	-0.44502116	-1.42173514	-0.33543691	0.24825529	-1.10136957	-0.1117052	-0.84273458	-0.61208915
ENSMUSG00000034958	Atcay	ataxia, cerebell	protein_coding	5,482	5,246	5,389	5,047	4,774	4,539	4,804	5,109	4,791	4,970	4,716	4,765	-1.1	0.00333	0.0809		-1.1	0.00317	0.0346	down	1	0.991	1	5	1644	1.72165841	0.96142048	1.4274958	0.29455844	-0.6655727	-1.53341905	-0.55881618	0.50510794	-0.60539398	0.02916183	-0.87677531	-0.69942567
ENSMUSG00000019731	Slc35e1	solute carrier fa	protein_coding	2,084	2,057	2,081	2,025	1,835	1,794	1,911	1,915	1,855	1,839	1,820	1,867	-1.11	0.00156	0.0608		-1.12	0.000342	0.00841	down	-1.01	0.731	1	5	1645	1.46579358	1.22701098	1.43971973	0.94645834	-0.81881965	-1.22172082	-0.09655328	-0.05397863	-0.62888829	-0.7782657	-0.96895609	-0.51180018
ENSMUSG00000024767	Otub1	OTU domain, ul	protein_coding	4,127	3,896	4,029	3,806	3,579	3,357	3,523	3,647	3,438	3,491	3,478	3,556	-1.12	0.000291	0.0334	down	-1.14	0.0000639	0.00288	down	-1.01	0.74	1	5	1646	1.84811501	0.97201742	1.48196096	0.61751165	-0.3124986	-1.27601085	-0.55074706	-0.02607151	-0.91987463	-0.68518506	-0.74233592	-0.40688142
ENSMUSG00000020261	Slc36a1	solute carrier fa	protein_coding	1,576	1,556	1,470	1,383	1,294	1,287	1,293	1,348	1,360	1,324	1,273	1,295	-1.15	0.00113	0.0538		-1.14	0.00143	0.0208	down	1.01	0.894	1	5	1647	1.89944245	1.72413073	0.95649068	0.14525388	-0.74274745	-0.81660154	-0.75097694	-0.20484919	-0.07781986	-0.43678865	-0.95916471	-0.73636941
ENSMUSG00000038582	Pptc7	PTC7 protein pl	protein_coding	1,984	2,032	1,864	1,772	1,693	1,546	1,506	1,715	1,656	1,653	1,581	1,544	-1.18	0.000336	0.0348	down	-1.19	0.000191	0.00577	down	-1	0.922	1	5	1648	1.55287464	1.80102471	0.90742294	0.39103577	-0.07186566	-0.99529342	-1.25867035	0.05895356	-0.29774232	-0.31738989	-0.76557491	-1.00477508
ENSMUSG00000042444	Mindy2	MINDY lysine 4	protein_coding	3,403	3,552	3,383	3,150	3,012	3,030	3,035	3,146	3,100	3,106	3,170	3,005	-1.1	0.00122	0.055		-1.09	0.00389	0.0391	down	1.01	0.687	1	5	1649	1.30078456	2.09331986	1.19243469	-0.1172838	-0.93392025	-0.82832131	-0.79927618	-0.13842357	-0.41270667	-0.37540751	-1.117E-05	-0.98118867
ENSMUSG00000039485	Tspyl4	TSPY-like 4	protein_coding	20,579	20,128	20,402	18,213	17,252	16,703	16,926	18,525	17,855	17,728	17,432	16,804	-1.14	0.00357	0.0836		-1.14	0.00528	0.0471	down	1.01	0.898	1	5	1650	1.6458764	1.35381845	1.53178956	0.03532931	-0.67838931	-1.1045185	-0.93018609	0.25929291	-0.22611256	-0.31998028	-0.54174349	-1.02517639
ENSMUSG00000048027	Rgmb	repulsive guida	protein_coding	2,533	2,631	2,604	2,307	2,154	2,143	2,046	2,247	2,192	2,258	2,172	2,097	-1.17	0.000256	0.0331	down	-1.16	0.000777	0.0141	down	1.01	0.735	1	5	1651	1.26672683	1.71973923	1.59542309	0.16653018	-0.64204942	-0.70034209	-1.24181268	-0.14579733	-0.43711882	-0.08795519	-0.54065727	-0.95268653
ENSMUSG00000025786	Zdhhc3	zinc finger, DHH	protein_coding	1,674	1,714	1,626	1,641	1,465	1,489	1,400	1,512	1,497	1,537	1,480	1,439	-1.13	0.000111	0.0251	down	-1.12	0.000404	0.00945	down	1.01	0.653	1	5	1652	1.33793793	1.70567134	0.87519611	1.02403072	-0.74106554	-0.49699477	-1.44009378	-0.25056131	-0.41184786	0.00310375	-0.58483289	-1.02054369
ENSMUSG00000007812	Zfp655	zinc finger prot	protein_coding	1,366	1,386	1,293	1,255	1,146	1,089	1,158	1,178	1,197	1,186	1,153	1,037	-1.16	0.00132	0.0568		-1.16	0.000987	0.0167	down	-1	0.981	1	5	1653	1.51613859	1.68897474	0.87077128	0.51926374	-0.53090712	-1.12331644	-0.41437061	-0.21342023	-0.03272453	-0.13968791	-0.45883425	-1.68188727
ENSMUSG00000048732	Klhl11	kelch-like 11	protein_coding	1,661	1,759	1,725	1,583	1,417	1,444	1,451	1,478	1,506	1,497	1,445	1,376	-1.16	0.000662	0.0456	down	-1.15	0.000872	0.0153	down	1.01	0.895	1	5	1654	1.07800616	1.80064579	1.55471906	0.47257906	-0.91017844	-0.67979085	-0.61989644	-0.38440534	-0.15033341	-0.22508091	-0.66424616	-1.27201853
ENSMUSG00000001687	Ubl3	ubiquitin-like 3	protein_coding	3,842	3,540	3,771	3,271	3,283	3,214	2,932	3,207	3,165	3,037	3,259	2,962	-1.14	0.00344	0.0817		-1.16	0.000845	0.015	down	-1.02	0.696	1	5	1655	1.8450239	0.88772762	1.62735004	-0.02914913	0.01219771	-0.23344023	-1.29594768	-0.25835835	-0.41378764	-0.89078393	-0.07277291	-1.17805941
ENSMUSG00000041028	Ghitm	growth hormor	protein_coding	8,507	7,841	8,448	7,333	7,384	7,424	6,704	7,336	7,632	7,264	7,160	6,865	-1.11	0.00405	0.0878		-1.11	0.00479	0.0444	down	1	0.948	1	5	1656	1.7990271	0.66568253	1.70256979	-0.26392692	-0.16857747	-0.0927091	-1.50601407	-0.25780516	0.28963432	-0.39569269	-0.59486811	-1.17732023
ENSMUSG00000052738	Suclg1	succinate-CoA l	protein_coding	2,576	2,228	2,489	2,185	2,167	2,079	1,987	2,137	2,069	2,157	2,029	2,001	-1.13	0.00777	0.117		-1.15	0.00278	0.0317	down	-1.01	0.762	1	5	1657	2.13059946	0.33202482	1.69918201	0.09008375	-0.00998916	-0.52269983	-1.07447472	-0.18551314	-0.57871207	-0.07073413	-0.81768918	-0.9920778
ENSMUSG00000017466	Timp2	tissue inhibitor	protein_coding	18,623	17,217	18,170	16,408	16,232	15,905	16,461	16,273	16,498	16,654	15,955	15,975	-1.09	0.00315	0.0802		-1.08	0.00443	0.0423	down	1	0.907	1	5	1658	2.1686408	0.62570009	1.68433018	-0.31997625	-0.53223505	-0.93104172	-0.25735331	-0.48188044	-0.21278937	-0.02802891	-0.86964129	-0.84572472
ENSMUSG00000041697	Cox6a1	cytochrome c o	protein_coding	2,500	2,170	2,387	2,111	2,028	1,953	1,966	1,979	2,074	2,049	1,941	1,838	-1.16	0.00229	0.0714		-1.16	0.00167	0.0229	down	-1	0.949	1	5	1659	2.11857288	0.50006659	1.59043473	0.19000209	-0.26705825	-0.68950863	-0.61253898	-0.54231822	-0.00967106	-0.14846768	-0.75621695	-1.37329653
ENSMUSG00000071379	Hpcal1	hippocalcin-like	protein_coding	2,609	2,478	2,347	2,301	2,311	1,939	2,161	2,124	2,129	2,161	2,041	2,230	-1.14	0.00107	0.0533		-1.14	0.00126	0.0193	down	1	0.929	1	5	1660	1.91977329	1.28775455	0.62681807	0.38334452	0.43588908	-1.68608353	-0.38073744	-0.58694331	-0.558911	-0.37772376	-1.06718865	0.00400818
ENSMUSG00000027673	Ndufb5	NADH:ubiquino	protein_coding	2,182	2,163	2,181	1,923	2,016	1,735	1,875	1,856	1,891	1,848	1,820	1,816	-1.13	0.00564	0.102		-1.15	0.00171	0.0234	down	-1.01	0.738	1	5	1661	1.51749977	1.40892978	1.51569762	-0.09458883	0.51076138	-1.39181753	-0.41113685	-0.54153215	-0.30773553	-0.59850731	-0.78882146	-0.81874889
ENSMUSG00000033174	Mgll	monoglyceride	protein_coding	1,215	1,193	1,177	1,079	1,051	906	977	929	994	1,059	932	936	-1.21	0.000113	0.0251	down	-1.19	0.000327	0.00822	down	1.02	0.726	1	5	1662	1.57118123	1.38888888	1.25649681	0.4169087	0.16896301	-1.23752887	-0.52995328	-1.00653967	-0.36189053	0.23531584	-0.97261559	-0.92922653
ENSMUSG00000043445	Pgp	phosphoglycola	protein_coding	1,236	1,282	1,251	960	986	891	920	936	939	1,005	871	891	-1.27	0.002	0.068		-1.28	0.00134	0.02	down	-1.01	0.927	1	5	1663	1.46810487	1.73379619	1.55755464	-0.32451926	-0.14088818	-0.84186814	-0.62436304	-0.49984732	-0.48100222	-0.00559441	-0.99923505	-0.84213808
ENSMUSG00000056234	Ncoa4	nuclear recepto	protein_coding	3,189	3,382	3,284	2,949	2,849	2,812	2,835	2,813	2,988	2,923	2,616	2,679	-1.13	0.000743	0.0481	down	-1.14	0.000256	0.00703	down	-1.01	0.807	1	5	1664	1.06113637	1.81698136	1.43736722	0.05972912	-0.38068189	-0.54870877	-0.44286344	-0.54498355	0.22787241	-0.05360468	-1.46559987	-1.16664428
ENSMUSG00000035150	Eif2s3x	eukaryotic tran	protein_coding	4,545	4,556	4,572	4,053	3,820	3,724	3,897	3,911	4,108	3,876	3,757	3,874	-1.15	0.00015	0.0282	down	-1.13	0.000748	0.0138	down	1.02	0.651	1	5	1665	1.51446809	1.54602574	1.59118152	0.02036128	-0.7516717	-1.08223515	-0.491926	-0.44508454	0.19368873	-0.56172078	-0.96574093	-0.56734627
ENSMUSG00000036158	Prickle1	prickle planar c	protein_coding	1,103	1,089	1,134	984	938	896	917	954	976	935	877	937	-1.16	0.000433	0.0378	down	-1.16	0.000462	0.0103	down	1	0.907	1	5	1666	1.46527302	1.31230484	1.8049831	0.10434598	-0.46225883	-0.99771822	-0.72411454	-0.26509483	0.00164138	-0.50515463	-1.25395994	-0.48024733
ENSMUSG00000028756	Pink1	PTEN induced p	protein_coding	8,476	8,403	8,745	7,482	7,555	7,290	7,389	7,111	7,612	7,244	7,118	7,006	-1.13	0.00916	0.125		-1.14	0.00395	0.0394	down	-1.01	0.788	1	5	1667	1.45274067	1.33751556	1.87015399	-0.20689215	-0.07910184	-0.55330055	-0.37298354	-0.88336138	0.02148421	-0.63680531	-0.86909779	-1.08035187
ENSMUSG00000021816	Ppp3cb	protein phosph	protein_coding	5,823	5,702	5,983	5,560	5,382	5,231	5,150	5,249	5,428	5,319	5,218	5,232	-1.1	0.0000647	0.0229	down	-1.09	0.000237	0.00662	down	1.01	0.701	1	5	1668	1.42315573	0.98998247	1.97908863	0.47002679	-0.19759697	-0.7823334	-1.09956137	-0.70965469	-0.02234733	-0.43988088	-0.83416445	-0.77671453
ENSMUSG00000030307	Slc6a11	solute carrier fa	protein_coding	22,917	23,295	24,294	21,356	21,200	20,655	20,053	20,415	21,293	20,725	19,983	20,343	-1.12	0.00216	0.0697		-1.12	0.00218	0.0273	down	1	0.994	1	5	1669	1.12887739	1.38808608	2.05261017	0.01318121	-0.10236256	-0.51418402	-0.98173976	-0.69870317	-0.03317171	-0.46078822	-1.03714506	-0.75466034
ENSMUSG00000007038	Neu1	neuraminidase	protein_coding	1,221	1,220	1,317	1,085	1,035	1,013	1,047	1,119	1,058	1,097	1,033	1,046	-1.14	0.000302	0.0338	down	-1.14	0.000288	0.00757	down	1	0.917	1	5	1670	1.21180849	1.20217509	2.14252316	-0.21341695	-0.78074166	-1.03767916	-0.63564652	0.15584287	-0.51077325	-0.08015697	-0.80245186	-0.65148324
ENSMUSG00000027599	Armc1	armadillo repea	protein_coding	1,868	1,876	1,947	1,679	1,615	1,555	1,653	1,657	1,618	1,683	1,549	1,607	-1.14	0.0005	0.0388	down	-1.14	0.000265	0.00716	down	-1	0.925	1	5	1671	1.34437539	1.39451455	1.89443396	-0.06868062	-0.58674667	-1.07922431	-0.27699175	-0.24470123	-0.5591564	-0.04148488	-1.12974894	-0.6465891
ENSMUSG00000023004	Tuba1b	tubulin, alpha 1	protein_coding	20,656	19,162	20,235	17,446	17,303	16,609	17,628	17,539	17,090	17,181	16,799	16,745	-1.12	0.00382	0.0855		-1.14	0.000772	0.0141	down	-1.02	0.641	1	5	1672	1.99471174	0.98038956	1.71644543	-0.28668623	-0.39810108	-0.94992276	-0.14666553	-0.21539038	-0.5646587	-0.49314031	-0.79653021	-0.84045153
ENSMUSG00000024758	Rtn3	reticulon 3	protein_coding	28,116	27,123	28,331	25,503	24,816	23,898	24,620	25,370	25,019	24,881	23,893	24,110	-1.1	0.000573	0.042	down	-1.11	0.000189	0.00574	down	-1.01	0.777	1	5	1673	1.68766968	1.08273511	1.81609176	0.04726702	-0.41234976	-1.04563271	-0.5454433	-0.04112458	-0.27510509	-0.36795708	-1.04931654	-0.89685451
ENSMUSG00000005683	Cs	citrate synthase	protein_coding	6,099	5,984	5,988	5,447	5,349	5,089	5,434	5,574	5,461	5,447	5,142	5,230	-1.1	0.00243	0.0729		-1.1	0.00088	0.0154	down	-1.01	0.789	1	5	1674	1.6955701	1.37442289	1.38581328	-0.19537698	-0.50031152	-1.32998729	-0.23505151	0.18794726	-0.15346286	-0.19707836	-1.1571566	-0.87532841
ENSMUSG00000051675	Trim32	tripartite motif	protein_coding	3,716	3,766	3,745	3,153	3,140	3,011	3,286	3,280	3,142	3,259	3,066	3,024	-1.13	0.0109	0.137		-1.15	0.0033	0.0355	down	-1.02	0.706	1	5	1675	1.47054947	1.63189972	1.56659455	-0.50719404	-0.55703118	-1.05869083	-0.01029838	-0.03208911	-0.54675343	-0.1096637	-0.84177503	-1.00554804
ENSMUSG00000071649	B3gat3	beta-1,3-glucur	protein_coding	2,566	2,281	2,439	2,067	2,063	2,011	2,018	2,164	2,069	2,076	2,019	2,164	-1.13	0.00265	0.0759		-1.12	0.00448	0.0427	down	1.01	0.839	1	5	1676	2.20772853	0.71057039	1.56216817	-0.52738796	-0.55271386	-0.86702338	-0.82372042	0.04952279	-0.51421643	-0.46952565	-0.82291935	0.04751716

ENSMUSG00000043702	Pde12	phosphodiester	protein_coding	693	695	643	638	606	555	552	583	623	576	620	587	-1.16	0.000784
ENSMUSG00000043991	Pura	purine rich elen	protein_coding	5,233	4,835	4,839	4,760	4,302	4,336	4,247	4,211	4,418	4,242	4,638	4,295	-1.15	0.000443
ENSMUSG00000045248	Med26	mediator comp	protein_coding	344	349	365	273	251	238	268	254	270	285	291	245	-1.31	0.0000331
ENSMUSG00000047030	Spata2	spermatogenes	protein_coding	927	855	843	779	736	663	739	734	726	786	727	718	-1.18	0.000267
ENSMUSG00000017664	Slc35c2	solute carrier fa	protein_coding	946	856	912	834	738	701	750	801	825	801	794	793	-1.18	0.000285
ENSMUSG00000028528	Dnajc6	DnaJ heat shocl	protein_coding	10,743	10,360	10,545	10,014	9,355	9,166	9,659	9,894	9,900	9,825	9,505	9,338	-1.09	0.000523
ENSMUSG00000035847	Ids	iduronate 2-sul	protein_coding	16,453	16,121	16,614	15,401	14,460	14,370	14,856	14,779	15,242	15,076	14,930	14,579	-1.1	0.000571
ENSMUSG00000026817	Ak1	adenylate kinas	protein_coding	2,188	2,055	2,069	2,048	1,859	1,878	1,911	1,791	1,958	1,983	1,859	1,950	-1.13	0.000247
ENSMUSG00000053963	Stum	mechanosenso	protein_coding	1,602	1,545	1,638	1,484	1,265	1,310	1,402	1,323	1,510	1,313	1,309	1,436	-1.18	0.0000909
ENSMUSG00000031604	Msmo1	methylsterol m	protein_coding	2,568	2,397	2,480	2,258	2,133	1,990	2,241	2,101	2,152	2,253	2,020	2,239	-1.15	0.000305
ENSMUSG00000021273	Fdft1	farnesyl diphos	protein_coding	3,110	2,931	2,967	2,783	2,542	2,448	2,837	2,596	2,684	2,663	2,554	2,742	-1.13	0.0000966
ENSMUSG00000093930	Hmgcs1	3-hydroxy-3-me	protein_coding	8,301	7,857	8,159	7,552	7,177	6,886	7,624	7,133	7,530	7,265	6,920	7,641	-1.11	0.000648
ENSMUSG00000028437	Ubap1	ubiquitin-assoc	protein_coding	1,332	1,378	1,536	1,278	1,086	1,190	1,190	1,218	1,134	1,239	1,178	1,245	-1.18	0.000109
ENSMUSG00000043391	2510009E07Rik	RIKEN cDNA 25	protein_coding	2,630	2,673	2,748	2,484	2,335	2,328	2,356	2,333	2,605	2,488	2,287	2,504	-1.13	0.000311
ENSMUSG00000043496	Tril	TLR4 interactor	protein_coding	2,644	2,794	2,797	2,431	2,227	2,315	2,262	2,404	2,473	2,477	2,176	2,347	-1.16	0.000177
ENSMUSG00000090394	4930523C07Rik	RIKEN cDNA 49	protein_coding	52	128	78	143	36	37	46	43	31	94	44	36	-2.44	0.000273
ENSMUSG00000025340	Rabgef1	RAB guanine nu	protein_coding	964	936	961	933	826	805	874	847	942	931	917	901	-1.13	0.000679
ENSMUSG00000116378	Gcat	glycine C-acetyl	protein_coding	37	34	27	25	0	10	10	14	28	41	27	27	-3.45	0.00000409
ENSMUSG00000030523	Trpm1	transient recep	protein_coding	6	17	12	12	16	10	6	4	3	3	3	3	-1.33	0.456
ENSMUSG00000095325	Zfp870	zinc finger prot	protein_coding	286	383	393	363	341	306	281	257	284	323	235	306	-1.2	0.0178
ENSMUSG00000000948	Gm38393	predicted gene	protein_coding	767	757	782	880	797	667	732	632	650	607	696	583	-1.13	0.149
ENSMUSG00000055200	Sertad3	SERTA domain c	protein_coding	52	53	48	53	49	42	46	39	38	32	35	36	-1.18	0.231
ENSMUSG00000050052	Tdrp	testis developm	protein_coding	647	684	670	722	604	601	634	553	531	604	610	546	-1.14	0.0201
ENSMUSG00000032578	Cish	cytokine induci	protein_coding	151	143	199	203	128	90	138	95	93	109	87	99	-1.55	0.00432
ENSMUSG00000036114	Rpp25l	ribonuclease P/	protein_coding	175	222	214	209	170	158	192	161	160	168	152	149	-1.2	0.0349
ENSMUSG00000048787	Dcun1d3	DCN1, defectiv	protein_coding	773	675	605	929	690	821	515	545	423	520	552	699	-1.16	0.169
ENSMUSG00000047539	Fbxo28	F-box protein 2	protein_coding	1,442	1,331	1,299	1,453	1,330	1,222	1,201	1,231	1,122	1,175	1,122	1,375	-1.11	0.0386
ENSMUSG00000050931	Sgms2	sphingomyelin	protein_coding	339	302	257	339	267	230	234	233	169	281	153	249	-1.28	0.0359
ENSMUSG00000039458	Mtmr12	myotubularin re	protein_coding	470	492	564	772	506	517	481	471	467	434	479	429	-1.16	0.0671
ENSMUSG00000041025	Iffo2	intermediate fil	protein_coding	815	839	806	893	806	804	770	847	715	759	748	717	-1.04	0.335
ENSMUSG00000073775	Kti12	KTI12 homolog,	protein_coding	251	231	236	257	192	214	196	225	188	202	200	193	-1.18	0.0251
ENSMUSG00000039725	Trp53rka	transformation	protein_coding	351	388	380	317	345	327	345	280	278	303	236	253	-1.11	0.153
ENSMUSG00000053128	Rnf26	ring finger prot	protein_coding	466	518	492	466	475	449	481	447	417	428	374	395	-1.05	0.426
ENSMUSG00000018593	Sparc	secreted acidic	protein_coding	37,483	36,957	39,809	34,557	36,403	34,994	36,352	35,115	32,602	33,614	32,396	32,032	-1.04	0.301
ENSMUSG00000031987	Egln1	egl-9 family hyp	protein_coding	1,849	1,883	1,910	1,735	1,816	1,724	1,797	1,720	1,610	1,614	1,584	1,612	-1.05	0.243
ENSMUSG00000003161	Sri	sorcin	protein_coding	1,617	1,573	1,673	1,508	1,601	1,553	1,548	1,403	1,403	1,366	1,277	1,392	-1.04	0.235
ENSMUSG00000059713	Rcan3	regulator of cal	protein_coding	3,233	3,234	3,358	3,113	3,137	3,126	3,257	2,938	2,801	2,818	2,745	2,765	-1.04	0.343
ENSMUSG00000029826	Zc3hav1	zinc finger CCC	protein_coding	784	724	734	744	727	752	754	697	670	686	637	667	-1.02	0.612
ENSMUSG00000039983	Ccdc32	coiled-coil dom	protein_coding	847	791	849	762	767	773	828	759	666	723	690	733	-1.04	0.457
ENSMUSG00000091478	Gm10039	predicted pseu	processed_pseudoge	314	260	315	205	197	219	296	226	49	195	85	104	-1.17	0.378
ENSMUSG00000029780	Nt5c3	5'-nucleotidase	protein_coding	846	820	808	719	786	736	747	685	726	737	681	671	-1.08	0.0717
ENSMUSG00000031760	Mt3	metallothionei	protein_coding	2,749	2,686	2,759	2,339	2,608	2,295	2,554	2,385	2,248	2,385	2,328	2,224	-1.07	0.16
ENSMUSG00000046062	Ppp1r15b	protein phosph	protein_coding	2,065	2,004	2,016	1,742	1,809	1,710	1,798	1,705	1,676	1,674	1,695	1,585	-1.11	0.00622
ENSMUSG00000057530	Ece1	endothelin con	protein_coding	2,960	3,090	2,923	2,813	3,120	2,382	2,678	2,487	2,293	2,623	2,384	2,263	-1.11	0.0723
ENSMUSG00000021967	Mrpl57	mitochondrial r	protein_coding	501	434	478	438	421	365	400	379	359	402	363	366	-1.18	0.00174
ENSMUSG00000027593	Raly	hnRNP-associat	protein_coding	1,729	1,620	1,639	1,598	1,608	1,463	1,514	1,392	1,404	1,539	1,423	1,388	-1.1	0.00843
ENSMUSG00000028684	Urod	uroporphyrinog	protein_coding	762	743	767	700	760	692	741	672	634	670	656	637	-1.04	0.398
ENSMUSG00000033350	Chst2	carbohydrate su	protein_coding	1,796	1,674	1,740	1,504	1,714	1,551	1,670	1,535	1,433	1,532	1,466	1,495	-1.04	0.371
ENSMUSG00000020821	Kif1c	kinesin family n	protein_coding	4,635	4,136	4,470	4,243	4,511	3,974	4,324	4,080	3,922	3,995	3,850	4,093	-1.04	0.313
ENSMUSG00000029616	Erp29	endoplasmic re	protein_coding	2,615	2,530	2,591	2,459	2,608	2,325	2,495	2,389	2,245	2,367	2,294	2,355	-1.04	0.246
ENSMUSG00000027663	Zmat3	zinc finger matr	protein_coding	3,108	3,066	2,976	2,718	2,897	2,681	2,644	2,658	2,406	2,677	2,716	2,780	-1.09	0.0369
ENSMUSG00000032349	Elovl5	ELOVL family m	protein_coding	2,826	2,676	2,723	2,587	2,536	2,372	2,564	2,355	2,214	2,420	2,341	2,461	-1.1	0.00116
ENSMUSG00000074030	Exoc8	exocyst comple	protein_coding	1,122	1,067	1,089	1,042	1,060	964	1,028	960	943	940	934	988	-1.08	0.0504
ENSMUSG00000091811	Inafm1	InaF motif cont	protein_coding	777	718	747	676	698	630	675	688	606	680	624	662	-1.08	0.0801
ENSMUSG00000033557	Fam20b	FAM20B, glyco	protein_coding	4,463	4,328	4,406	4,171	4,245	4,039	4,158	4,036	3,908	4,101	3,976	4,011	-1.05	0.0725
ENSMUSG00000046229	Scand1	SCAN domain-c	protein_coding	511	491	551	456	474	403	461	429	369	443	395	411	-1.14	0.0414
ENSMUSG00000039770	Ypel5	yippee like 5	protein_coding	2,476	2,437	2,405	2,404	2,137	2,270	2,449	2,198	2,183	2,177	2,055	2,071	-1.07	0.0425
ENSMUSG00000061130	Ppm1b	protein phosph	protein_coding	3,358	3,285	3,081	3,174	2,980	2,973	3,178	2,975	3,016	2,941	2,731	2,899	-1.07	0.0551
ENSMUSG00000070705	Eid2b	EP300 interacti	protein_coding	714	673	697	667	611	599	661	564	580	644	551	581	-1.13	0.0201
ENSMUSG00000078235	Fam43b	family with seq	protein_coding	531	495	500	476	435	417	509	408	419	455	406	400	-1.13	0.041
ENSMUSG00000025903	Lypla1	lysophospholip	protein_coding	958	891	915	848	719	732	948	784	701	836	666	699	-1.14	0.0392
ENSMUSG00000029012	Orc5	origin recogniti	protein_coding	660	667	619	609	551	559	654	567	566	581	523	558	-1.1	0.047
ENSMUSG00000050447	Lypd6	LY6/PLAUR don	protein_coding	819	809	826	838	600	683	847	634	567	684	655	700	-1.19	0.00799
ENSMUSG00000035062	Zc4h2	zinc finger, C4H	protein_coding	441	397	446	425	346	408	398	371	339	375	342	359	-1.12	0.0346
ENSMUSG00000042747	Krtcap2	keratinocyte as	protein_coding	474	401	437	379	287	418	440	340	351	346	319	302	-1.15	0.108
ENSMUSG00000046709	Mapk10	mitogen-activa	protein_coding	7,672	7,947	6,544	7,661	6,745	6,750	7,119	7,108	6,667	6,417	6,302	6,718	-1.08	0.0805
ENSMUSG00000020029	Nudt4	nudix (nucleosi	protein_coding	3,693	3,613	3,372	3,200	3,293	3,225	3,474	3,358	3,002	3,120	3,036	3,253	-1.04	0.303
ENSMUSG00000100235	Gm28557	predicted gene	protein_coding	103	110	66	81	86	79	84	74	46	49	48	52	-1.11	0.458
ENSMUSG00000016503	Gtf3a	general transcri	protein_coding	465	468	418	442	408	410	501	414	399	369	382	347	-1.04	0.492
ENSMUSG00000032220	Myo1e	myosin IE	protein_coding	449	472	408	391	345	388	530	378	357	362	346	344	-1.05	0.481
ENSMUSG00000007659	Bcl2l1	BCL2-like 1	protein_coding	2,543	2,121	2,148	1,966	2,039	2,063	2,265	1,952	1,876	1,827	1,995	1,813	-1.06	0.222
ENSMUSG00000078670	Fam174b	family with seq	protein_coding	699	685	602	552	608	548	638	576	501	501	575	482	-1.07	0.258
ENSMUSG00000099908	Gm28539	predicted gene	protein_coding	270	164	233	265	167	186	254	205	123	163	116	184	-1.15	0.38
ENSMUSG00000028967	Errfi1	ERBB receptor f	protein_coding	839	739	786	768	720	695	761	719	600	649	672	719	-1.08	0.149
ENSMUSG00000117098	Gm49909	predicted gene	protein_coding	1,471	1,028	1,224	997	830	902	1,192	1,006	689	767	834	981	-1.2	0.127
ENSMUSG00000052684	Jun	jun proto-onco	protein_coding	1,191	1,178	1,188	1,265	1,062	1,098	1,248	1,206	1,041	1,028	1,096	1,072	-1.04	0.343
ENSMUSG00000039680	Mrps6	mitochondrial r	protein_coding	540	463	541	503	449	412	549	520	426	422	463	426	-1.06	0.358
ENSMUSG00000056851	Pcbp2	poly(rC) binding	protein_coding	6,504	6,140	6,786	6,182	6,072	6,069	6,534	6,316	5,790	5,871	6,077	6,090	-1.02	0.354
ENSMUSG00000042275	Pelo	pelota mRNA su	protein_coding	393	333	412	351	316	286	325	325	292	307	252	305	-1.19	0.00595
ENSMUSG00000062647	Rpl7a	ribosomal prote	protein_coding	5,995	5,421	5,993	5,324	5,067	5,220	5,392	5,191	5,213	5,369	4,812	5,132	-1.09	0.018
ENSMUSG00000029103	Lrpap1	low density lipo	protein_coding	5,627	5,150	5,837	5,089	4,971	5,002	5,186	5,001	4,904	4,957	4,733	5,010	-1.08	0.0178
ENSMUSG00000037503	Fam168b	family with seq	protein_coding	6,782	6,241	6,766	6,185	6,070	5,980	6,124	5,962	5,946	5,995	5,590	6,099	-1.08	0.00519
ENSMUSG00000028291	Akirin2	akirin 2	protein_coding	1,368	1,296	1,487	1,238	1,211	1,210	1,267	1,194	1,181	1,157	1,155	1,157	-1.1	0.0134
ENSMUSG00000000532	Acvr1b	activin A recept	protein_coding	2,965	2,920	3,057	2,863	2,787	2,674	2,856	2,792	2,768	2,694	2,712	2,694	-1.06	0.0285
ENSMUSG00000005034	Prkacb	protein kinase,	protein_coding	24,312	23,134	25,133	23,407	22,546	21,925	22,925	22,519	22,389	21,487	21,374	21,448	-1.07	0.0589
ENSMUSG00000029757	Dync1i1	dynein cytoplas	protein_coding	2,943	2,810	3,038	2,745	2,510	2,564	2,818	2,561	2,517	2,644	2,420	2,569	-1.1	0.0176
ENSMUSG00000001847	Rac1	Rac family smal	protein_coding	9,023	8,399	9,056	7,997	8,118	8,061	8,579	7,835	7,827	7,752	7,488	7,793	-1.06	0.0681
ENSMUSG00000051853	Arf3	ADP-ribosylatio	protein_coding	16,408	15,509	16,966	15,080	14,936	14,342	15,702	14,346	14,594	14,467	13,976	14,852	-1.08	0.0346
ENSMUSG00000028221	Pip4p2	phosphatidylino	protein_coding	1,252	1,222	1,270	1,166	1,124	1,060	1,187	1,091	1,089	1,095	1,066	1,087	-1.1	0.0175
ENSMUSG00000044528	Tram1l1	translocation a	protein_coding	1,433	1,350	1,430	1,286	1,269	1,191	1,372	1,215	1,258	1,201	1,143	1,207	-1.09	0.0543
ENSMUSG00000051864	Tbc1d22a	TBC1 domain fa	protein_coding	720	707	777	634	624	591	714	621	636	602	553	596	-1.11	0.0254
ENSMUSG00000024812	Tjp2	tight junction p	protein_coding	1,628	1,531	1,607	1,506	1,526	1,448	1,647	1,476	1,447	1,341	1,370	1,460	-1.03	0.46
ENSMUSG00000080921	Rpl38-ps2	ribosomal prote	processed_pseudoge	340	282	389	274	311	247	350	300	238	229	179	257	-1.06	0.624
ENSMUSG00000046324	Ermp1	endoplasmic re	protein_coding	2,406	2,246	2,222	2,170	2,085	1,953	2,189	2,052	1,988	1,948	2,011	2,096	-1.09	0.0198
ENSMUSG00000048756	Foxo3	forkhead box O	protein_coding	1,877	1,812	1,719	1,669	1,620	1,574	1,745	1,547	1,574	1,496	1,531	1,581	-1.09	0.00895
ENSMUSG00000032840	Spring1	SREBF pathway	protein_coding	1,388	1,226	1,308	1,182	1,122	1,061	1,331	1,097	1,161	1,035	1,104	1,110	-1.11	0.0257
ENSMUSG00000028690	Mmachc	methylmalonic	protein_coding	471	378	448	412	354	350	434	382	354	340	347	361	-1.12	0.082
ENSMUSG00000047423	AI837181	expressed sequ	protein_coding	948	803	888	840	772	735	864	758	787	762	695	771	-1.11	0.0263
ENSMUSG00000063049	Ing2	inhibitor of gro	protein_coding	449	328	355	344	324	288	335	347	323	311	272	297	-1.13	0.075
ENSMUSG00000068856	Sf3b4	splicing factor 3	protein_coding	1,406	1,175	1,266	1,144	1,245	1,049	1,216	1,181	1,116	1,089	1,068	1,089	-1.06	0.183
ENSMUSG00000020109	Dnajb12	DnaJ heat shocl	protein_coding	1,252	1,090	1,159	1,099	1,054	1,055	1,106	1,054	1,045	1,083	1,051	993	-1.08	0.0287
ENSMUSG00000014867	Surf4	surfeit gene 4	protein_coding	2,158	1,825	1,899	1,714	1,774	1,721	1,841	1,672	1,558	1,678	1,605	1,525	-1.08	0.0376
ENSMUSG00000036442	Thap11	THAP domain c	protein_coding	632	537	597	507	514	478	532	497	444	491	440	436	-1.12	0.0365
ENSMUSG00000020628	Trappc12	trafficking prot	protein_coding	1,848	1,747	1,749	1,726	1,668	1,643	1,652	1,631	1,661	1,661	1,580	1,567	-1.07	0.0193
ENSMUSG00000000058	Cav2	caveolin 2	protein_coding	779	661	678	586	595	559	606	604	585	543	536	521	-1.14	0.0804
ENSMUSG00000001910	Nacc1	nucleus accum	protein_coding	3,545	3,106	3,288	3,140	3,037	2,998	3,062	3,109	3,062	2,869	2,873	2,824	-1.07	0.0374
ENSMUSG00000057531	Dtnbp1	dystrobrevin bi	protein_coding	878	760	764	739	685	709	770	764	671	704	663	700	-1.07	0.161
ENSMUSG00000023156	Rpp14	ribonuclease P	protein_coding	675	615	599	521	447	465	555	571	506	503	456	491	-1.18	0.00918
ENSMUSG00000029312	Klhl8	kelch-like 8	protein_coding	711	706	659	620	561	578	668	654	579	579	566	614	-1.09	0.0752
ENSMUSG00000004748	Mtfp1	mitochondrial f	protein_coding	1,048	935	979	877	835	813	934	930	815	846	822	858	-1.09	0.0709
ENSMUSG00000032348	Gsta4	glutathione S-tr	protein_coding	960	890	929	816	804	750	893	842	778	803	743	798	-1.09	0.0521
ENSMUSG00000063856	Gpx1	glutathione per	protein_coding	1,356	1,177	1,341	1,138	1,097	1,005	1,196	1,184	1,063	1,103	1,090	1,035	-1.12	0.0392
ENSMUSG00000038077	Kcna6	potassium volta	protein_coding	3,743	3,394	3,715	3,330	3,187	3,006	3,303	3,441	2,958	2,996	2,959	2,978	-1.1	0.104
ENSMUSG00000063063	Ctnna2	catenin (cadher	protein_coding	3,378	3,137	3,384	3,043	2,964	2,835	3,072	3,053	2,922	2,850	2,841	2,939	-1.08	0.0083
ENSMUSG00000023307	Marchf5	membrane asso	protein_coding	1,409	1,359	1,346	1,262	1,241	1,235	1,311	1,252	1,219	1,229	1,191	1,175	-1.07	0.041
ENSMUSG00000019505	Ubb	ubiquitin B	protein_coding	41,415	38,033	39,525	33,988	34,512	32,277	36,512	34,625	33,247	34,449	32,923	32,629	-1.11	0.00998
ENSMUSG00000036752	Tubb4b	tubulin, beta 4E	protein_coding	7,012	6,499	6,667	5,948	5,734	5,546	6,219	5,882	5,776	5,931	5,609	5,517	-1.12	0.00152
ENSMUSG00000045319	Proser2	proline and seri	protein_coding	127	101	110	77	88	68	102	85	76	64	82	67	-1.2	0.115
ENSMUSG00000024309	Pfdn6	prefoldin subur	protein_coding	788	709	746	641	620	613	689	651	624	589	605	595	-1.12	0.0588
ENSMUSG00000041037	Irgq	immunity-relate	protein_coding	3,097	3,041	3,084	2,679	2,711	2,659	2,891	2,813	2,697	2,610	2,663	2,627	-1.07	0.0231
ENSMUSG00000003955	Fam162a	family with seq	protein_coding	472	417	461	495	389	404	433	434	425	369	368	354	-1.11	0.0687
ENSMUSG00000036376	Abt1	activator of bas	protein_coding	376	326	353	350	304	322	331	315	309	279	295	298	-1.11	0.0616
ENSMUSG00000023861	Mpc1	mitochondrial p	protein_coding	1,758	1,749	1,805	1,662	1,643	1,645	1,510	1,655	1,612	1,532	1,559	1,509	-1.08	0.0293
ENSMUSG00000021218	Gdi2	guanosine diph	protein_coding	7,417	6,817	7,305	7,090	6,634	6,728	6,477	6,703	6,588	6,356	6,743	6,352	-1.08	0.0246
ENSMUSG00000026811	St6galnac6	ST6 (alpha-N-ac	protein_coding	2,085	1,962	2,024	2,008	1,909	1,906	1,814	1,902	1,797	1,788	1,791	1,745	-1.07	0.0443
ENSMUSG00000029729	Zkscan1	zinc finger with	protein_coding	2,445	2,412	2,465	2,428	2,275	2,282	2,289	2,321	2,298	2,231	2,261	2,192	-1.06	0.0434
ENSMUSG00000031948	Kars	lysyl-tRNA synt	protein_coding	1,926	1,920	1,971	1,887	1,698	1,743	1,754	1,759	1,668	1,823	1,577	1,699	-1.11	0.00134
ENSMUSG00000066979	Bub3	BUB3 mitotic ch	protein_coding	1,908	1,848	1,858	1,846	1,595	1,718	1,793	1,724	1,718	1,784	1,585	1,636	-1.09	0.0161
ENSMUSG00000022848	Slc49a4	solute carrier fa	protein_coding	2,123	1,953	2,067	1,966	1,840	1,912	1,934	1,949	1,834	1,934	1,696	1,752	-1.06	0.103
ENSMUSG00000029998	Pcyox1	prenylcysteine	protein_coding	4,612	3,933	4,604	4,203	3,908	3,907	3,939	4,092	3,885	3,974	3,417	3,420	-1.09	0.0319
ENSMUSG00000017288	Vps53	VPS53 GARP co	protein_coding	2,013	1,897	1,920	1,929	1,762	1,715	1,873	1,851	1,769	1,814	1,586	1,630	-1.08	0.0424
ENSMUSG00000021266	Wars1	tryptophanyl-tR	protein_coding	2,362	2,259	2,372	2,275	2,187	2,104	2,218	2,086	2,136	2,143	1,911	1,918	-1.08	0.0881
ENSMUSG00000024614	Tmx3	thioredoxin-rel	protein_coding	3,226	3,002	2,969	3,020	2,762	2,903	2,711	2,658	2,650	2,719	2,716	2,638	-1.11	0.00145
ENSMUSG00000026965	Anapc2	anaphase prom	protein_coding	2,426	2,367	2,259	2,228	1,993	2,234	2,128	2,134	1,921	2,213	1,994	1,902	-1.09	0.0354
ENSMUSG00000038619	Ensa	endosulfine alp	protein_coding	5,508	5,054	5,162	5,009	4,751	4,925	4,793	4,998	4,894	4,730	4,530	4,743	-1.06	0.0534
ENSMUSG00000017831	Rab5a	RAB5A, membe	protein_coding	3,907	3,599	3,871	3,590	3,326	3,515	3,439	3,473	3,492	3,365	3,230	3,301	-1.09	0.0167
ENSMUSG00000049119	Fam110b	family with seq	protein_coding	721	652	721	639	548	618	612	643	576	588	527	575	-1.13	0.0415
ENSMUSG00000026670	Uap1	UDP-N-acetylgl	protein_coding	830	849	818	795	725	765	733	769	726	752	712	720	-1.1	0.0198
ENSMUSG00000025872	Thoc3	THO complex 3	protein_coding	907	855	909	795	726	821	718	758	732	743	675	680	-1.15	0.00826
ENSMUSG00000042428	Mgat3	mannoside ace	protein_coding	4,870	4,676	4,812	4,425	4,166	4,434	4,281	4,353	4,255	4,307	4,096	4,264	-1.09	0.00384
ENSMUSG00000032199	Polr2m	polymerase (RN	protein_coding	6,628	5,647	6,038	5,998	5,596	5,566	5,297	5,549	5,140	5,308	5,420	5,735	-1.1	0.0158
ENSMUSG00000032262	Elovl4	elongation of v	protein_coding	1,393	1,221	1,305	1,298	1,162	1,241	1,141	1,198	1,106	1,106	1,082	1,190	-1.1	0.032
ENSMUSG00000022044	Stmn4	stathmin-like 4	protein_coding	3,211	2,802	3,005	2,806	2,725	2,800	2,735	2,606	2,576	2,662	2,664	2,732	-1.09	0.0164
ENSMUSG00000022892	App	amyloid beta (A	protein_coding	50,869	45,934	48,069	46,311	45,564	44,767	44,926	45,239	44,384	44,414	44,219	45,289	-1.06	0.0134
ENSMUSG00000047085	Lrrc4b	leucine rich rep	protein_coding	5,909	5,088	5,776	5,019	5,152	4,959	5,096	4,897	4,869	4,892	4,851	4,995	-1.08	0.0275
ENSMUSG00000033768	Nrxn2	neurexin II	protein_coding	15,080	13,922	13,960	13,704	13,614	13,451	13,551	13,318	13,003	12,814	12,915	13,477	-1.05	0.0397
ENSMUSG00000074918	Inafm2	InaF motif cont	protein_coding	976	860	879	806	816	840	823	842	766	772	789	801	-1.06	0.123
ENSMUSG00000034226	Rhov	ras homolog fa	protein_coding	394	359	382	368	311	326	345	327	295	300	290	344	-1.15	0.0304
ENSMUSG00000026511	Srp9	signal recogniti	protein_coding	1,798	1,645	1,750	1,670	1,551	1,516	1,566	1,560	1,456	1,428	1,474	1,551	-1.11	0.0257
ENSMUSG00000005469	Prkaca	protein kinase,	protein_coding	7,514	7,129	7,472	7,031	6,841	6,746	6,842	6,998	6,548	6,653	6,536	6,725	-1.06	0.0313
ENSMUSG00000055932	Fto	fat mass and ob	protein_coding	6,793	6,379	6,540	6,234	6,090	5,890	6,076	6,172	5,895	5,793	5,805	5,991	-1.07	0.0192
ENSMUSG00000043716	Rpl7	ribosomal prote	protein_coding	5,885	5,228	6,123	4,959	5,090	4,955	5,046	4,740	4,538	4,535	4,330	5,194	-1.12	0.061
ENSMUSG00000068039	Tcp1	t-complex prote	protein_coding	6,104	6,011	6,540	5,775	5,570	5,694	5,685	5,914	5,283	5,544	5,275	5,771	-1.07	0.0474
ENSMUSG00000025318	Jph3	junctophilin 3	protein_coding	4,733	4,529	4,706	4,060	4,193	4,064	4,243	4,354	3,943	4,026	3,983	4,120	-1.07	0.0829
ENSMUSG00000031749	St3gal2	ST3 beta-galact	protein_coding	2,516	2,420	2,469	2,213	2,215	2,226	2,260	2,309	2,061	2,114	2,109	2,178	-1.07	0.123
ENSMUSG00000022354	Ndufb9	NADH:ubiquino	protein_coding	2,004	1,930	2,005	1,844	1,871	1,730	1,804	1,816	1,767	1,701	1,694	1,784	-1.08	0.0518
ENSMUSG00000049866	Arl4c	ADP-ribosylatio	protein_coding	3,146	3,035	3,113	2,808	2,816	2,675	2,836	2,836	2,680	2,598	2,613	2,645	-1.08	0.0317
ENSMUSG00000070287	Slc35g2	solute carrier fa	protein_coding	1,074	1,081	1,078	983	969	925	984	980	910	903	936	913	-1.09	0.0409
ENSMUSG00000020444	Guk1	guanylate kinas	protein_coding	1,032	976	968	940	865	825	899	919	831	884	858	841	-1.11	0.0278
ENSMUSG00000009394	Syn2	synapsin II	protein_coding	11,394	10,822	10,903	10,114	9,878	9,563	10,123	10,211	9,592	9,958	9,688	9,745	-1.09	0.0244
ENSMUSG00000018481	Appbp2	amyloid beta pr	protein_coding	4,380	4,237	4,286	4,025	3,863	3,901	3,993	4,062	3,886	3,953	3,853	3,909	-1.07	0.0233
ENSMUSG00000040794	C1qtnf4	C1q and tumor	protein_coding	855	749	860	660	612	634	619	629	570	635	556	628	-1.25	0.000706
ENSMUSG00000056553	Ptprn2	protein tyrosine	protein_coding	8,131	7,706	7,900	7,241	7,068	7,030	7,148	7,099	6,927	7,003	6,860	7,088	-1.09	0.000494

ENSMUSG00000031840	Rab3a	RAB3A, membe	protein_coding	11,417	10,694	10,806	10,117	9,855	9,800	9,795	10,104	9,537	9,532	9,714	10,153	-1.09	0.00479	0.0941	-1.11	0.00076	0.014 down	-1.02	0.594	1	7	1917	2.20044281	1.01293085	1.20162518	0.00684986	-0.46838993	-0.56873907	-0.57874954	-0.01635032	-1.06227574	-1.0705646	-0.7282662	0.07149634
[illegible]	[illegible]	[illegible]	[illegible]	[illegible]	[illegible]	[illegible]	[illegible]	[illegible]	[illegible]	[illegible]	[illegible]	[illegible]	[illegible]	[illegible]	[illegible]	[illegible]	[illegible]	[illegible]	[illegible]	[illegible]	[illegible]	[illegible]	[illegible]	[illegible]	[illegible]	[illegible]	[illegible]	[illegible]	[illegible]	[illegible]	[illegible]	[illegible]	[illegible]	[illegible]	[illegible]	[illegible]	[illegible]	[illegible]
ENSMUSG00000116165	Pdxp	pyridoxal (pyrid	protein_coding	4,189	3,989	4,337	3,769	3,794	3,293	3,703	3,697	3,491	3,599	3,329	3,556	-1.12	0.00471	0.0936	-1.16	0.000197	0.00589 down	-1.04	0.38	1	7	2031	1.42744935	0.84054798	1.84303536	0.16374505	0.24389978	-1.43802775	-0.04595606	-0.06558749	-0.74823734	-0.38533124	-1.30860932	-0.52692833
ENSMUSG00000028445	Enho	energy homeos	protein_coding	971	915	937	751	817	724	729	754	772	764	690	710	-1.18	0.00509	0.0965	-1.22	0.000814	0.0146 down	-1.03	0.616	1	7	2032	1.81124587	1.28830924	1.49287669	-0.44646854	0.28739718	-0.75152591	-0.69665977	-0.40259346	-0.19915756	-0.29441044	-1.16387699	-0.9251363
ENSMUSG00000019210	Atp6v1e1	ATPase, H+ trar	protein_coding	4,381	4,249	4,441	3,872	3,990	3,721	3,864	3,961	3,997	3,800	3,689	3,729	-1.09	0.027	0.207	-1.11	0.00562	0.0491 down	-1.02	0.588	1	7	2033	1.57469722	1.08959972	1.78964814	-0.38706309	0.08910186	-1.01423267	-0.41944392	-0.02456045	0.1168046	-0.68199042	-1.15182839	-0.9807326
ENSMUSG00000025204	Ndufb8	NADH:ubiquino	protein_coding	2,617	2,529	2,552	2,244	2,309	2,069	2,157	2,294	2,205	2,127	2,083	2,145	-1.13	0.00889	0.124	-1.16	0.000835	0.0149 down	-1.03	0.483	1	7	2034	1.74708131	1.32108147	1.43627529	-0.14756975	0.20084422	-1.13816937	-0.62903159	0.12516571	-0.36266272	-0.79973351	-1.05352583	-0.69975524
ENSMUSG00000031633	Slc25a4	solute carrier fa	protein_coding	12,679	11,987	12,528	11,235	11,371	10,747	11,092	11,465	11,101	11,160	10,146	10,968	-1.08	0.0192	0.179	-1.12	0.00133	0.02 down	-1.03	0.389	1	7	2035	1.76365589	0.86649911	1.57190225	-0.16692304	0.02514436	-0.87373212	-0.37097054	0.15680382	-0.35754556	-0.27398055	-1.7913172	-0.54953539
ENSMUSG00000006315	Tmem147	transmembran	protein_coding	914	834	913	718	749	690	733	727	740	752	629	675	-1.16	0.0132	0.151	-1.21	0.00192	0.025 down	-1.04	0.558	1	7	2036	1.73933041	0.9112397	1.72218305	-0.40495357	-0.03508726	-0.75800475	-0.22383907	-0.30584789	-0.14939638	-0.00694218	-1.5408914	-0.94779066
ENSMUSG00000054793	Cadm4	cell adhesion m	protein_coding	5,184	4,925	5,303	4,608	4,596	4,422	4,558	4,638	4,530	4,614	4,242	4,351	-1.1	0.0114	0.141	-1.13	0.00111	0.018 down	-1.03	0.473	1	7	2037	1.61257685	0.84465728	1.95334957	-0.14998766	-0.19053112	-0.766033	-0.31555059	-0.0548232	-0.40761813	-0.1314936	-1.38704067	-1.00750572
ENSMUSG00000031818	Cox4i1	cytochrome c o	protein_coding	5,794	5,745	5,943	5,097	5,278	4,889	5,063	5,166	4,892	5,011	4,631	4,894	-1.11	0.00823	0.119	-1.16	0.0000856	0.00346 down	-1.05	0.203	1	7	2038	1.42490598	1.31477367	1.7510909	-0.2220624	0.2254301	-0.75527149	-0.30920819	-0.04864515	-0.74881248	-0.44138274	-1.44950742	-0.74131079
ENSMUSG00000034994	Eef2	eukaryotic tran	protein_coding	35,562	35,225	36,476	32,774	32,514	31,376	31,312	32,761	31,477	31,756	30,214	30,537	-1.09	0.00254	0.0745	-1.13	0.0000444	0.00231 down	-1.03	0.289	1	7	2039	1.41528398	1.25960401	1.82941943	0.08238178	-0.047316	-0.62895897	-0.66210904	0.07589386	-0.57632099	-0.43239943	-1.24432621	-1.07115241
ENSMUSG00000058254	Tspan7	tetraspanin 7	protein_coding	15,386	14,294	15,289	13,416	13,597	12,790	13,526	13,382	13,299	13,104	12,733	12,906	-1.1	0.0071	0.111	-1.12	0.000664	0.0128 down	-1.02	0.481	1	7	2040	1.91803222	0.76080116	1.81842096	-0.23485857	-0.02411519	-0.98409982	-0.10696637	-0.27426308	-0.37191442	-0.60417269	-1.05433943	-0.84252478
ENSMUSG00000067925	Rtl8a	retrotransposo	protein_coding	6,276	5,804	6,214	5,158	5,221	4,963	5,281	5,164	5,006	5,072	4,853	4,860	-1.14	0.00135	0.057	-1.18	0.0000202	0.00141 down	-1.04	0.298	1	7	2041	1.87950613	1.00626379	1.7686653	-0.30898282	-0.17362166	-0.73734876	-0.04680814	-0.29656533	-0.64039783	-0.49539946	-0.98546271	-0.96984851
ENSMUSG00000020708	Psmc5	protease (prosc	protein_coding	3,652	3,481	3,678	3,152	3,152	2,990	3,170	3,244	3,094	3,135	2,963	3,021	-1.11	0.00451	0.0921	-1.14	0.00029	0.00761 down	-1.03	0.449	1	7	2042	1.71253072	1.06042245	1.81111468	-0.28951337	-0.28845601	-1.00268711	-0.21374174	0.10085221	-0.54020543	-0.36148892	-1.12504652	-0.86378096
ENSMUSG00000051391	Ywhag	tyrosine 3-mon	protein_coding	29,491	29,050	29,977	26,486	26,030	25,184	26,329	26,394	25,275	25,803	24,972	24,916	-1.11	0.00172	0.0636	-1.14	0.0000533	0.00259 down	-1.03	0.367	1	7	2043	1.5569256	1.32922188	1.80452718	-0.06798444	-0.33005247	-0.82930552	-0.15766515	-0.12030006	-0.77463781	-0.46264513	-0.95702814	-0.99105593
ENSMUSG00000026932	Nacc2	nucleus accuml	protein_coding	5,130	4,938	4,990	4,675	4,296	4,219	4,403	4,368	4,304	4,357	3,936	4,227	-1.14	0.00142	0.0585	-1.17	0.000113	0.00416 down	-1.03	0.513	1	7	2044	1.71777314	1.23687614	1.36989654	0.54958125	-0.50801874	-0.73526286	-0.20168932	-0.29996221	-0.48621324	-0.33181377	-1.59950709	-0.71165983
ENSMUSG00000068923	Syt11	synaptotagmin	protein_coding	21,883	21,501	21,673	20,324	19,866	19,323	20,049	20,384	19,906	19,634	18,323	19,290	-1.07	0.0151	0.16	-1.11	0.000404	0.00945 down	-1.03	0.271	1	7	2045	1.56913913	1.23271898	1.38536987	0.15996487	-0.27499064	-0.80296061	-0.10030583	0.21606724	-0.23668805	-0.49800514	-1.81513171	-0.83517812
ENSMUSG00000025428	Atp5a1	ATP synthase, H	protein_coding	18,143	18,176	18,636	16,444	16,011	15,935	16,267	16,808	16,169	16,171	15,109	15,423	-1.1	0.00718	0.112	-1.14	0.00025	0.00689 down	-1.03	0.332	1	7	2046	1.35947367	1.38664988	1.76197177	-0.11805361	-0.51887862	-0.59065108	-0.28100844	0.21034329	-0.37144862	-0.36978302	-1.38840891	-1.08020631
ENSMUSG00000059995	Atxn7l3	ataxin 7-like 3	protein_coding	3,397	3,296	3,332	3,011	2,875	2,837	2,928	3,057	2,922	2,953	2,664	2,771	-1.11	0.0233	0.193	-1.15	0.00276	0.0315 down	-1.03	0.477	1	7	2047	1.67162406	1.26537429	1.413682	0.06491556	-0.54880725	-0.7213067	-0.30368607	0.26422841	-0.33104089	-0.19102121	-1.55060047	-1.03336174
ENSMUSG00000025781	Atp5c1	ATP synthase, H	protein_coding	4,384	4,113	4,460	3,900	3,743	3,659	3,786	4,037	3,788	3,780	3,580	3,784	-1.11	0.00359	0.0836	-1.13	0.000439	0.01 down	-1.02	0.564	1	7	2048	1.66427538	0.7357208	1.91233592	-0.03579259	-0.62881627	-0.95667431	-0.46529281	0.46367704	-0.45619532	-0.48835078	-1.27216034	-0.47272674
ENSMUSG00000047710	Champ1	chromosome al	protein_coding	1,447	1,405	1,447	1,340	1,282	1,220	1,268	1,365	1,279	1,220	1,234	1,267	-1.1	0.00658	0.108	-1.13	0.000237	0.00662 down	-1.03	0.377	1	7	2049	1.5560154	1.0782756	1.55639644	0.33340826	-0.3677845	-1.1510785	-0.54952921	0.62429378	-0.41281029	-1.14978011	-0.96722946	-0.55017742
ENSMUSG00000017707	Serinc3	serine incorpor	protein_coding	6,299	5,869	6,229	5,461	5,713	5,683	5,779	5,916	5,570	5,479	5,345	5,482	-1.03	0.257	0.577	-1.09	0.00225	0.0278 down	-1.06	0.0547	1	7	2050	1.83212706	0.46683122	1.61736176	-0.92123631	-0.05121576	-0.15255278	0.16774905	0.6194262	-0.54117476	-0.8561643	-1.33401106	-0.84714033
ENSMUSG00000027195	Hsd17b12	hydroxysteroid	protein_coding	2,212	2,043	2,110	1,913	1,985	1,922	1,949	2,100	1,949	1,927	1,772	1,854	-1.04	0.283	0.601	-1.1	0.00558	0.0489 down	-1.06	0.0902	1	7	2051	1.87246941	0.55898667	1.09181845	-0.52329434	0.08624731	-0.44607242	-0.21556641	1.01256191	-0.22150094	-0.40291316	-1.77390944	-1.03882705
ENSMUSG00000009863	Sdhb	succinate dehyc	protein_coding	2,439	2,242	2,284	2,057	2,040	1,925	2,038	2,129	2,067	2,043	1,875	1,808	-1.11	0.0152	0.161	-1.16	0.000501	0.0108 down	-1.04	0.304	1	7	2052	1.94725238	0.9352931	1.15661781	-0.09178559	-0.18972053	-0.87638153	-0.19798106	0.31982206	-0.0306088	-0.1725805	-1.18641387	-1.61351347
ENSMUSG00000031703	Itfg1	integrin alpha F	protein_coding	8,101	7,602	8,157	7,260	7,167	7,052	7,223	7,302	7,386	7,111	6,723	6,733	-1.08	0.0251	0.2	-1.11	0.00234	0.0285 down	-1.03	0.427	1	7	2053	1.70195263	0.65392376	1.81546535	-0.10452674	-0.31630469	-0.58054709	-0.18777478	-0.00906806	0.17859136	-0.44329813	-1.36676327	-1.34165032
ENSMUSG00000115987	Vps28	vacuolar protei	protein_coding	1,859	1,710	1,816	1,550	1,574	1,505	1,599	1,587	1,626	1,620	1,477	1,443	-1.11	0.0117	0.142	-1.12	0.00303	0.0336 down	-1.02	0.688	1	7	2054	1.87950956	0.78709426	1.57523948	-0.4915703	-0.29512596	-0.87475883	-0.08541759	-0.18681698	0.12861465	0.07848107	-1.10851756	-1.4067318
ENSMUSG00000000420	Galnt1	polypeptide N-a	protein_coding	2,367	2,273	2,218	2,072	2,147	2,013	2,145	2,172	2,163	2,058	1,995	1,982	-1.05	0.104	0.379	-1.09	0.00577	0.0499 down	-1.03	0.267	1	7	2055	1.94143003	1.1921435	0.74024956	-0.51958179	0.13999512	-1.04620674	0.1192108	0.352306	0.27196501	-0.64457615	-1.21713114	-1.32980419
ENSMUSG00000001017	Chtop	chromatin targe	protein_coding	3,273	3,183	3,196	2,924	3,049	2,913	3,089	3,093	2,905	2,949	2,823	2,753	-1.03	0.204	0.521	-1.1	0.000321	0.00814 down	-1.06	0.0204	1	7	2056	1.5897962	1.06441631	1.13831753	-0.53674261	0.24917737	-0.60959507	0.49353719	0.51868354	-0.65896563	-0.37833528	-1.19771572	-1.67257382
ENSMUSG00000028779	Pef1	penta-EF hand	protein_coding	1,278	1,186	1,199	978	1,039	949	1,141	1,056	1,010	1,058	919	891	-1.11	0.0897	0.353	-1.2	0.0029	0.0326 down	-1.08	0.205	1	7	2057	1.73805521	1.06141358	1.15662611	-0.65439428	-0.12116268	-0.91840317	0.71288952	0.02191754	-0.37395587	0.03938168	-1.20038194	-1.4619857
ENSMUSG00000027546	Atp9a	ATPase, class II,	protein_coding	11,131	10,938	11,042	9,859	9,442	9,541	10,486	9,843	9,727	9,781	9,121	9,245	-1.09	0.013	0.15	-1.13	0.00041	0.00952 down	-1.04	0.297	1	7	2058	1.55194868	1.30010702	1.43648286	-0.1904527	-0.80977223	-0.66116586	0.69396534	-0.21421241	-0.38459869	-0.3051449	-1.30533026	-1.11182686
ENSMUSG00000034189	Hsdl1	hydroxysteroid	protein_coding	2,007	2,020	2,070	1,754	1,713	1,682	1,892	1,749	1,693	1,719	1,633	1,682	-1.12	0.00544	0.101	-1.17	0.0000693	0.003 down	-1.05	0.247	1	7	2059	1.3448525	1.42199408	1.71914204	-0.28571258	-0.56791461	-0.78833327	0.6297506	-0.31557751	-0.70561196	-0.5274534	-1.13988516	-0.78525074
ENSMUSG00000036275	9530068E07Rik	RIKEN cDNA 95	protein_coding	2,661	2,606	2,754	2,254	2,316	2,275	2,482	2,340	2,292	2,395	2,183	2,260	-1.09	0.031	0.221	-1.12	0.0033	0.0355 down	-1.03	0.447	1	7	2060	1.4131636	1.13165619	1.87813365	-0.81559974	-0.4514003	-0.69186368	0.47730279	-0.31496461	-0.59447848	-0.00194877	-1.24566883	-0.78433183
ENSMUSG00000040385	Ppp1ca	protein phosph	protein_coding	3,072	2,965	3,227	2,666	2,660	2,519	2,824	2,674	2,656	2,667	2,423	2,593	-1.12	0.00819	0.119	-1.15	0.000588	0.0119 down	-1.03	0.441	1	7	2061	1.39088168	0.96215483	1.98615997	-0.31773436	-0.34244862	-0.99344181	0.37432477	-0.28069117	-0.36274433	-0.31048346	-1.45626372	-0.64971376
ENSMUSG00000003346	Abhd17a	abhydrolase do	protein_coding	1,121	1,050	1,134	860	925	890	955	881	865	889	835	787	-1.14	0.0359	0.234	-1.23	0.000724	0.0136 down	-1.08	0.207	1	7	2062	1.65003581	1.07196722	1.75419075	-0.64865793	-0.02064888	-0.35744423	0.24701088	-0.43790061	-0.60150217	-0.36454916	-0.89437913	-1.39812258
ENSMUSG00000048758	Rpl29	ribosomal prot	protein_coding	2,621	2,545	2,734	2,093	2,232	2,212	2,384	2,258	2,217	2,219	2,060	2,050	-1.1	0.0606	0.295	-1.17	0.00189	0.0248 down	-1.06	0.223	1	7	2063	1.42131323	1.10679233	1.86947399	-0.96207554	-0.28797339	-0.38094376	0.41036652	-0.16396361	-0.35927048	-0.34708197	-1.12885946	-1.17777786
ENSMUSG00000028757	Ddost	dolichyl-di-pho	protein_coding	2,539	2,542	2,616	2,191	2,255	2,173	2,382	2,353	2,277	2,293	2,088	2,141	-1.08	0.0564	0.287	-1.12	0.00298	0.0333 down	-1.04	0.297	1	7	2064	1.26882323	1.28592697	1.68509799	-0.7575359	-0.36162316	-0.87083193	0.38840361	0.21889977	-0.23042205	-0.13458912	-1.41718376	-1.07496566
ENSMUSG00000029632	Ndufa4	Ndufa4, mitoch	protein_coding	2,651	2,576	2,723	2,100	2,252	2,060	2,213	2,274	2,264	2,222	1,921	1,948	-1.14	0.0346	0.231	-1.2	0.00312	0.0343 down	-1.05	0.406	1	7	2065	1.44016282	1.18488825	1.67894444	-0.62715602	-0.00934362	-0.79458392	-0.16283741	0.07366801	0.03704075	-0.12974274	-1.4061132	-1.28492737
ENSMUSG00000032046	Abhd12	abhydrolase do	protein_coding	5,048	5,009	5,085	4,268	4,504	4,203	4,538	4,484	4,443	4,292	4,180	4,334	-1.09	0.0308	0.219	-1.12	0.00462	0.0435 down	-1.03	0.508	1	7	2066	1.54228575	1.43347136	1.64381638	-0.80737384	-0.05615785	-1.02095534	0.04981893	-0.11821623	-0.24495501	-0.7302753	-1.09747106	-0.5939878
ENSMUSG00000037419	Endod1	endonuclease c	protein_coding	4,779	4,676	4,603	3,859	3,937	3,919	4,212	3,974	4,138	3,966	3,684	3,794	-1.12	0.0228	0.192	-1.15	0.00387	0.039 down	-1.03	0.548	1	7	2067	1.73993189	1.48671044	1.30252911	-0.74272912	-0.51029773	-0.56479267	0.27090742	-0.4039644	0.06460893	-0.42772641	-1.27714413	-0.93803334
ENSMUSG00000004267	Eno2	enolase 2, gam	protein_coding	20,035	19,508	19,651	18,335	18,143	17,176	18,158	18,674	17,621	18,432	16,980	17,377	-1.07	0.0238	0.195	-1.1	0.00238	0.0289 down	-1.02	0.44	1	7	2068	1.67112969	1.17410389	1.31098252	0.01799679	-0.17753531	-1.19815298	-0.16197339	0.36010948	-0.72161413	0.11656624	-1.4109604	-0.98065238
ENSMUSG00000024099	Ndufv2	NADH:ubiquino	protein_coding	3,052	3,009	3,087	2,698	2,740	2,600	2,836	2,852	2,632	2,850	2,521	2,549	-1.07	0.087	0.348	-1.12	0.005	0.0455 down	-1.05	0.279	1	7	2069	1.34593041	1.1433817	1.50912143	-0.42906823	-0.20846807	-0.95767493	0.28988213	0.37098927	-0.78304794	0.35842425	-1.39817181	-1.24129822
ENSMUSG00000031451	Gas6	growth arrest s	protein_coding	2,860	2,794	2,849	2,588	2,638	2,548	2,650	2,692	2,448	2,658	2,346	2,448	-1.05	0.109	0.386	-1.12	0.000374	0.00892 down	-1.06	0.0519	1	7	2070	1.40695017	1.02440702	1.34243872	-0.21460887	0.09606466	-0.46378179	0.16968355	0.42405737	-1.10944602	0.21635701	-1.78523527	-1.10688656
ENSMUSG00000048307	Ankrd46	ankyrin repeat	protein_coding	3,893	3,862	3,952	3,471	3,607	3,404	3,614	3,607	3,443	3,504	3,114	3,204	-1.07	0.115	0.397	-1.14	0.000898	0.0156 down	-1.07	0.082	1	7	2071	1.2707024	1.16178175	1.47701111	-0.29904085	0.22414107	-0.56665266	0.25047843	0.22677173	-0.4104462	-0.17007123	-1.77484237	-1.38983319
ENSMUSG00000017776	Crk	v-crk avian sarc	protein_coding	3,296	3,365	3,377	3,024	3,043	2,819	3,135	2,939	2,845	2,978	2,801	2,867	-1.09	0.014	0.154	-1.14	0.000432	0.00988 down	-1.04	0.299	1	7	2072	1.21790598	1.52488189	1.58011415	-0.05290959	0.04221626	-1.08463013	0.48072233	-0.47121178	-0.94768736	-0.2750803	-1.17747654	-0.8368449
ENSMUSG00000039740	Alg2	asparagine-link	protein_coding	7,023	6,994	7,078	6,619	6,668	6,163	6,693	6,522	6,343	6,530	6,010	6,272	-1.06	0.0524	0.277	-1.1	0.00233	0.0284 down	-1.04	0.273	1	7	2073	1.28324753	1.20207079	1.43146018	0.14596938	0.28919998	-1.21783742	0.36070563	-0.13415029	-0.66724595	-0.11125326	-1.69830575	-0.88386084
ENSMUSG00000038888	Ctu1	cytosolic thiour	protein_coding	262	252	264	232	205	177	223	192	193	209	169	159	-1.27	0.00326	0.0805	-1.38	0.000042	0.00224 down	-1.09	0.289	1	7	2074	1.35915276	1.11470015	1.42858404	0.61232376	-0.13264594	-0.97607931	0.36887989	-0.51453916	-0.47590512	-0.01307067	-1.2180408	-1.55335959
ENSMUSG00000011752	Pgam1	phosphoglycera	protein_coding	9,862	9,588	9,996	8,846	8,553	7,969	8,687	8,715	8,488	8,619	7,904	7,741	-1.13	0.00367	0.0842	-1.17	0.000169	0.00543 down	-1.04	0.396	1	7	2075	1.48031204	1.14097529	1.64316177	0.17236252	-0.23283764	-1.08019121	-0.04542336	-0.00677362	-0.32425302	-0.14036328	-1.17890589	-1.4280636
ENSMUSG00000051065	Mb21d2	Mab-21 domair	protein_coding	975	964	975	889	872	837	908	869	849	874	822	814	-1.09	0.0331	0.226	-1.13	0.00183	0.0242 down	-1.04	0.352	1	7	2076	1.51851697	1.3295225	1.50359787	0.05859123	-0.24388896	-0.89015823	0.38679307	-0.30732017	-0.66410964	-0.21365817	-1.16942182	-1.30846466
ENSMUSG00000058672	Tubb2a	tubulin, beta 2A	protein_coding	13,045	12,636	12,956	11,464	10,982	10,530	11,771	11,326	10,946	11,151	10,421	10,354	-1.12	0.00197	0.0675	-1.17	0.000031	0.00187 down	-1.04	0.287	1	7	2077	1.61993184	1.22866418	1.53589252	0.03568941	-0.48974909	-1.00376182	0.35967814	-0.11201221	-0.53060895	-0.30256882	-1.13145599	-1.2096992
ENSMUSG00000024091	Vapa	vesicle-associat	protein_coding	2,927	2,884	3,174	2,578	2,590	2,408	2,650	2,695	2,516	2,738	2,456	2,399	-1.12	0.00896	0.124	-1.14	0.00142	0.0207 down	-1.05	0.581	1	7	2078	1.12705178	0.95187196	2.08822704	-0.36718904	-0.3090935	-1.160543	-0.04096114	0.15464237	-0.64913167	0.34222473	-0.9305347	-1.20656482
ENSMUSG00000032320	Rcn2	reticulocalbin 2	protein_coding	4,995	4,978	5,268	4,493	4,455	4,315	4,762	4,512	4,474	4,623	4,209	4,217	-1.09	0.019	0.179	-1.13	0.00174	0.0235 down	-1.03	0.44	1	7	2079	1.17215654	1.12386408	1.929925	-0.32888242	-0.44752814	-0.89857976	0.49669537	-0.26638421	-0.38879816	0.07633256	-1.24726725	-1.22153359
ENSMUSG00000035964	Tmem59l	transmembran	protein_coding	5,609	5,549	5,924	4,860	4,727	4,405	5,091	4,796	4,821	4,956	4,406	4,352	-1.15	0.0069	0.11	-1.18	0.00139	0.0205 down	-1.03	0.626	1	7	2080	1.28328952	1.17436079	1.83395433	-0.15544255	-0.43197209	-1.13785171	0.30919261	-0.28844268	-0.2360586	0.0412919	-1.13550444	-1.25681708
ENSMUSG00000057614	Gnai1	guanine nucleo	protein_coding	4,566	4,356	4,631	4,102	3,903	3,706	4,271	4,060	3,919	3,932	3,786	3,721	-1.11	0.0188	0.178	-1.15	0.00125	0.0192 down	-1.04	0.386	1	7	2081	1.52111484	0.8985134	1.70887008	0.10555485	-0.54817728	-1.2283137	0.63906764	-0.02787214	-0.49360546	-0.45111656	-0.94766953	-1.17636615
ENSMUSG00000018326	Ywhab	tyrosine 3-mon	protein_coding	16,082	15,531	16,430	14,708	14,533	13,897	15,025	14,825	14,210	14,502	14,110	13,840	-1.08	0.0296	0.216	-1.11	0.00256	0.0301 down	-1.03	0.404	1	7	2082	1.52062989	0.88890321	1.90868603	-0.0967951	-0.31352077	-1.12324918	0.28879812	0.04678062	-0.72129084	-0.35256907	-0.8483709	-1.19800201
ENSMUSG00000021087	Rtn1	reticulon 1	protein_coding	24,467	23,044	24,385	21,843	21,523	20,395	22,387	21,931	21,540	21,496	20,729	20,652	-1.09	0.0189	0.178	-1.11	0.00314	0.0344 down	-1.02	0.547	1	7	2083	1.78450144	0.78010734	1.72857427	-0.11731671	-0.36454632	-1.266367	0.29448926	-0.0501276	-0.35145696	-0.38607765	-0.99458339	-1.05719666
ENSMUSG00000021877	Arf4	ADP-ribosylatio	protein_coding	2,814	2,703	2,837	2,576	2,498	2,391	2,637	2,607	2,455	2,519	2,305	2,390	-1.08	0.0381	0.24	-1.13	0.000661	0.0128 down	-1.05	0.189	1	7	2084	1.48030211	0.85583922	1.60651648	0.11506299	-0.35590387	-1.02130039	0.47648498	0.30199781	-0.62193193	-0.22777448	-1.57969059	-1.02957535
ENSMUSG00000032399	Rpl4	ribosomal prot	protein_coding	12,328	11,960	12,810	11,627	11,469	10,781	11,860	11,614	11,226	11,352	10,714	10,836	-1.07	0.0527	0.277	-1.1	0.00241	0.029 down	-1.04	0.275	1	7	2085	1.22693639	0.66839917	1.93224515	0.14953423	-0.10168589	-1.2372291	0.51439746	0.12957223	-0.49470319	-0.28975815	-1.35254546	-1.14516286
ENSMUSG00000029030	Tprgl	transformation	protein_coding	3,541	3,428	3,728	3,394	3,423	2,967	3,601	3,115	3,156	3,186	3,019	2,859	-1.08	0.0594	0.293	-1.15	0.000208	0.00613 down	-1.07	0.0701	1	7	2086	0.93832201	0.54922917	1.55630686	0.42916202	0.52995517	-1.18054741	1.14116832	-0.59962779	-0.44368758	-0.32827736	-0.97088693	-1.62111648
ENSMUSG00000042364	Snx18	sorting nexin 18	protein_coding	870	876	968	841	911	825	898	805	769	802	791	733	-1.03	0.448	0.742	-1.15	0.00154	0.0218 down	-1.11	0.0154	1	7	2087	0.46612324	0.5481057	1.84066271	0.0411866	1.05178871	-0.21303474	0.87104255	-0.51437181	-1.09413625	-0.56863512	-0.74119123	-1.68754036
ENSMUSG00000027637	Rab5if	RAB5 interactin	protein_coding	505	516	613	477	481	463	527	419	433	442	461	424	-1.12	0.0707	0.317	-1.2	0.00279	0.0317 down	-1.07	0.25	1	7	2088	0.50940491	0.70603338	2.32485058	-0.02892539	0.05880311	-0.30091292	0.90283169	-1.1782423	-0.88891225	-0.70059916	-0.33351122	-1.07082041
ENSMUSG00000021496	Pcbd2	pterin 4 alpha c	protein_coding	183	184	215	169	176	149	174	148	148	167	152	144	-1.16	0.046	0.259	-1.23	0.00464	0.0436 down	-1.06	0.454	1	7	2089	0.77967247	0.83172676	2.19486355	0.11408615	0.43732883	-0.89091049	0.35886478	-0.94219386	-0.95272942	0.00468647	-0.75815535	-1.17723989
ENSMUSG00000035274	Tpbg	trophoblast gly	protein_coding	532	490	613	496	463	442	482	411	428	485	417	402	-1.19	0.00827	0.119	-1.23	0.00117	0.0184 down	-1.04	0.583	1	7	2090	1.04100667	0.3577845	2.25181822	0.45928047	-0.1091217	-0.4908267	0.2174954	-1.05837462	-0.74329913	0.27302633	-0.95445066	-1.24433877
ENSMUSG00000033735	Spr	sepiapterin red	protein_coding	606	568	718	545	589	530	605	534	508	547	487	529	-1.08	0.186	0.496	-1.17	0.00464	0.0436 down	-1.09	0.135	1	7	2091	0.7385433	0.11400363	2.43656261	-0.2887481	0.45820409	-0.55566099	0.72386256	-0.4868078	-0.95896639	-0.25806548	-1.35302208	-0.56990534
ENSMUSG00000040945	Rcc2	regulator of chr	protein_coding	2,035	1,986	2,288	1,922	1,959	1,887	2,006	1,887	1,802	1,871	1,822	1,822	-1.06	0.13	0.42	-1.12	0.00346	0.0365 down	-1.06	0.163	1	7	2092	0.74952112	0.37669228	2.54454892	-0.11553224	0.17527311	-0.39925829	0.53230984	-0.39829451	-1.09344992	-0.52562307	-0.92109807	-0.92508917
ENSMUSG00000015981	Stk32c	serine/threonin	protein_coding	1,870	1,702	1,889	1,643	1,708	1,597	1,834	1,600	1,537	1,640	1,535	1,558	-1.05	0.208	0.525	-1.13	0.00293	0.0328 down	-1.07	0.0874	1	7	2093	1.50661726	0.23419824	1.64735146	-0.23412725	0.28391638	-0.61614298	1.24661394	-0.59140697	-1.1290219	-0.25970523	-1.14415403	-0.94413892
ENSMUSG00000030588	Yif1b	Yip1 interacting	protein_coding	514	455	546	390	445	396	478	381	388	411	364	388	-1.12	0.0772	0.331	-1.22	0.00145	0.021 down	-1.09	0.167	1	7	2094	1.4432446	0.48807258	1.93182731	-0.69352611	0.32159643	-0.57137954	0.86828675	-0.85243728	-0.72402414	-0.29214338	-1.1873471	-0.73217012
ENSMUSG00000049086	Bmyc	brain expressec	protein_coding	1,010	859	929	797	831	716	820	794	733	854	770	726	-1.14	0.0209	0.187	-1.17	0.00508	0.046 down	-1.03	0.651	1	7	2095	2.1140195	0.49700908	1.27616224	-0.23668182	0.16645752	-1.26337177	0.03852494	-0.27975072	-1.04256008	0.43826081	-0.57298404	-1.13508566
ENSMUSG00000027223	Mapk8ip1	mitogen-activa	protein_coding	8,559	8,168	8,464	7,299	7,555	7,096	7,651	7,506	7,373	7,600	7,244	7,016	-1.09	0.0188	0.178	-1.11	0.00379	0.0385 down	-1.02	0.592	1	7	2096	1.79252033	1.07690589	1.62128373	-0.64326514	-0.11638103	-1.0720671	0.07593636	-0.21546704	-0.48820856	-0.02532677	-0.75918178	-1.24674889
ENSMUSG00000028436	Dcaf12	DDB1 and CUL4	protein_coding	1,220	1,211	1,231	1,043	1,093	950	1,093	1,059	1,002	1,070	1,030	946	-1.12	0.0248	0.198	-1.16	0.00267	0.031 down	-1.04	0.468	1	7	2097	1.41889381	1.3268298	1.51216648	-0.33887671	0.17686125	-1.37005482	0.18068111	-0.17035136	-0.78584258	-0.05474194	-0.47999296	-1.41557208
ENSMUSG00000031778	Cx3cl1	chemokine (C-X	protein_coding	7,155	6,824	7,130	6,366	6,318	5,969	6,348	6,386	6,116	6,166	6,396	5,861	-1.1	0.00208	0.0685	-1.12	0.000167	0.00537 down	-1.02	0.507	1	7	2098	1.73483078	0.98957376	1.68064701	-0.10369037	-0.22293715	-1.11271438	-0.14698741	-0.05294515	-0.73301204	-0.60351763	-0.02883116	-1.40041625
ENSMUSG00000069227	Gprin1	G protein-regul	protein_coding	2,415	2,293	2,510	2,202	2,231	2,065	2,182	2,183	2,141	2,121	2,184	2,004	-1.09	0.0146	0.157	-1.11	0.00117	0.0184 down	-1.03	0.444	1	7	2099	1.44256609	0.61301131	2.06591986	-0.03884083	0.1740699	-1.06680239	-0.18150377	-0.17537586	-0.48453957	-0.63625744	-0.1719741	-1.5402732
ENSMUSG00000059734	Ndufs8	NADH:ubiquino	protein_coding	1,324	1,199	1,291	1,193	1,164	1,043	1,187	1,154	1,092	1,139	1,087	1,006	-1.1	0.0518	0.275	-1.16	0.00247	0.0294 down	-1.05	0.29	1	7	2100	1.73435716	0.47841806	1.41694266	0.4162369	0.11250793	-1.24556935	0.35704205	0.00015285	-0.68249307	-0.1626315	-0.73389464	-1.69106905
ENSMUSG00000020149	Rab1a	RAB1A, membe	protein_coding	6,008	5,376	5,742	5,083	5,113	4,749	5,160	5,079	4,992	4,916	4,707	4,491	-1.1	0.0184	0.177	-1.16	0.000347	0.00847 down	-1.05	0.226	1	7	2101	2.00437753	0.63862651	1.4479938	-0.04807988	0.02506949	-0.87851817	0.13545938	-0.05660012	-0.26862589	-0.4551133	-0.98515511	-1.55943424
ENSMUSG00000047514	Tspyl1	testis-specific p	protein_coding	5,735	5,281	5,594	5,062	5,026	4,793	5,150	4,997	4,831	4,814	4,807	4,499	-1.09	0.0343	0.23	-1.14	0.000491	0.0107 down	-1.05	0.174	1	7	2102	1.89035538	0.68403655	1.52481371	0.06753069	-0.03721481	-0.72630723	0.31846971	-0.11948355	-0.61048765	-0.66231805	-0.6851004	-1.64429435
ENSMUSG00000026112	Coa5	cytochrome C c	protein_coding	2,738	2,570	2,869	2,414	2,448	2,428	2,603	2,486	2,418	2,433	2,303	2,278	-1.06	0.0948	0.362	-1.12	0.00129	0.0196 down	-1.06	0.125	1	7	2103	1.39674023	0.44488852	2.09924555	-0.48704653	-0.27660845	-0.40177401	0.6355952	-0.04888063	-0.4614572	-0.37102651	-1.18544861	-1.34422756
ENSMUSG00000028081	Rps3a1	ribosomal prot	protein_coding	8,754	7,999	9,089	7,582	7,690	7,736	8,418	7,674	7,735	7,685	7,210	7,186	-1.06	0.129	0.419	-1.12	0.00308	0.0339 down	-1.06	0.15	1	7	2104	1.47098476	0.21328981	1.99735335	-0.53433841	-0.33661563	-0.25420192	0.92516797	-0.36585018	-0.25489011	-0.34603194	-1.23424298	-1.28062474
ENSMUSG00000031059	Ndufb11	NADH:ubiquino	protein_coding	1,499	1,419	1,665	1,239	1,296	1,235	1,386	1,244	1,312	1,279	1,195	1,149	-1.13	0.0284	0.212	-1.18	0.00244	0.0293 down	-1.05	0.414	1	7	2105	1.21506829	0.68668475	2.2390463	-0.60155414	-0.1817391	-0.63502584	0.46294632	-0.56460949	-0.05953	-0.30608781	-0.9441714	-1.31102788
ENSMUSG00000005873	Reep5	receptor access	protein_coding	12,121	11,201	12,621	10,841	10,816	10,669	11,143	10,777	10,776	10,709	10,179	10,217	-1.08	0.029	0.214	-1.12	0.00122	0.019 down	-1.04	0.297	1	7	2106	1.56844485	0.30868459	2.21430544	-0.2111295	-0.24902618	-0.46686463	0.22680735	-0.30662651	-0.30685697	-0.40714262	-1.21482509	-1.15577074
ENSMUSG00000020612	Prkar1a	protein kinase,	protein_coding	26,746	25,351	27,477	24,422	24,351	23,842	25,239	24,051	24,279	24,329	23,349	23,316	-1.07	0.0309	0.22	-1.09	0.00341	0.0361 down	-1.02	0.443	1	7	2107	1.57372999	0.51407065	2.107261	-0.22384327	-0.28114218	-0.69875976	0.42668203	-0.52615675	-0.33973989	-0.29972682	-1.11222262	-1.14015239
ENSMUSG00000031534	Smim19	small integral m	protein_coding	934	866	962	823	830	756	879	803	780	820	741	744	-1.1	0.0459	0.259	-1.16	0.001	0.0168 down	-1.06	0.209	1	7	2108	1.46136463	0.56138961	1.81486422	-0.04210531	0.05205636	-1.02897973	0.7416339	-0.33241872	-0.66390241	-0.08068664	-1.2667133	-1.2165026
ENSMUSG00000007892	Rplp1	ribosomal prot	protein_coding	6,325	5,669	6,537	5,019	5,213	4,806	5,759	5,166	5,056	5,302	4,836	4,611	-1.12	0.0387	0.242	-1.19	0.00221	0.0276 down	-1.06	0.323	1	7	2109	1.57817427	0.56841874	1.88428683	-0.55210078	-0.20289619	-0.9492348	0.71283919	-0.28710323	-0.48412914	-0.04735797	-0.89214009	-1.32875683
ENSMUSG00000023010	Tmbim6	transmembran	protein_coding	9,608	9,146	9,823	8,349	8,758	8,384	9,191	8,500	8,614	8,586	8,045	7,992	-1.06	0.109	0.386	-1.11	0.00376	0.0384 down	-1.05	0.196	1	7	2110	1.45909036	0.70594801	1.79806489	-0.68571923	0.04421109	-0.62122894	0.78149755	-0.41159489	-0.20887561	-0.25922326	-1.25135716	-1.35081281
ENSMUSG00000056429	Tgoln1	trans-golgi netv	protein_coding	8,865	8,565	9,048	8,119	8,253	7,878	8,478	8,201	8,110	8,090	7,735	7,666	-1.05	0.0677	0.311	-1.09	0.00168	0.023 down	-1.04	0.191	1	7	2111	1.43488044	0.75802336	1.83873826	-0.29357612	0.02802132	-0.88452676	0.55762313	-0.09610202	-0.31594538	-0.36282856	-1.24472471	-1.41958297
ENSMUSG00000019054	Fis1	fission, mitocho	protein_coding	2,028	1,865	2,216	1,650	1,685	1,576	1,810	1,759	1,677	1,711	1,540	1,563	-1.14	0.0259	0.203	-1.19	0.00167	0.0229 down	-1.05	0.367	1	7	2112	1.37279419	0.59736404	2.19858951	-0.52680867	-0.33380875	-0.94214388	0.32104524	0.05665113	-0.37908197	-0.19632225	-1.15108999	-1.01718859
ENSMUSG00000074649	BC029722	cDNA sequence	protein_coding	1,855	1,717	1,909	1,587	1,658	1,534	1,717	1,669	1,567	1,601	1,489	1,542	-1.07	0.112	0.391	-1.14	0.00321	0.0349 down	-1.06	0.178	1	7	2113	1.54513552	0.52311433	1.92584931	-0.50774197	0.06692624	-0.94977664	0.52440349	0.15377048	-0.66927338	-0.39538144	-1.33781152	-0.87921441
ENSMUSG00000032437	Stt3b	STT3, subunit o	protein_coding	2,434	2,390	2,577	2,162	2,301	2,160	2,385	2,264	2,165	2,209	2,078	2,070	-1.05	0.155	0.455	-1.12	0.000579	0.0119 down	-1.07	0.0442	1	7	2114	1.08949285	0.81717969	1.93709834	-0.66549334	0.25238469	-0.68078763	0.78396824	0.01599038	-0.64603028	-0.34959401	-1.25053094	-1.305278
ENSMUSG00000048076	Arf1	ADP-ribosylatio	protein_coding	7,632	7,455	7,883	6,820	6,898	6,760	7,214	6,966	6,932	6,737	6,391	6,479	-1.07	0.0565	0.287	-1.12	0.00108	0.0176 down	-1.05	0.176	1	7	2115	1.35847458	0.98916819	1.86914559	-0.412421	-0.23450711	-0.550378	0.47124098	-0.078755	-0.15594408	-0.60596322	-1.43272311	-1.21733783
ENSMUSG00000001783	Rtcb	RNA 2',3'-cyclic	protein_coding	3,759	3,601	3,889	3,285	3,275	3,221	3,582	3,409	3,300	3,327	3,082	3,109	-1.08	0.0396	0.244	-1.13	0.000473	0.0104 down	-1.05	0.155	1	7	2116	1.39629146	0.80658115	1.86446532	-0.45228089	-0.49192552	-0.71899738	0.73235861	0.0538648	-0.3874097	-0.27865281	-1.32149044	-1.20280461
ENSMUSG00000060636	Rpl35a	ribosomal prot	protein_coding	3,865	3,662	4,001	3,352	3,426	3,334	3,685	3,529	3,382	3,361	3,168	3,130	-1.06	0.168	0.474	-1.14	0.00353	0.0368 down	-1.07	0.125	1	7	2117	1.38892577	0.66925167	1.85181662	-0.50761804	-0.21772503	-0.57793763	0.75174394	0.17709199	-0.39060065	-0.47321975	-1.25643985	-1.41528903
ENSMUSG00000031827	Cotl1	coactosin-like 1	protein_coding	2,058	1,774	2,226	1,599	1,837	1,546	1,866	1,657	1,688	1,598	1,462	1,318	-1.11	0.146	0.443	-1.26	0.000956	0.0163 down	-1.14	0.0649	1	7	2118	1.31539848	0.27753582	1.86886193	-0.43753841	0.52050888	-0.66956051	0.62717831	-0.19513982	-0.06832692	-0.44119481	-1.04886526	-1.74885769
ENSMUSG00000041355	Ssr2	signal sequence	protein_coding	1,069	1,015	1,129	927	1,014	908	961	920	915	928	847	842	-1.09	0.0706	0.317	-1.17	0.0006	0.0121 down	-1.08	0.11	1	7	2119	1.30136541	0.71620962	1.9331833	-0.31126785	0.69613192	-0.5492191	0.09617872	-0.39646555	-0.46455611	-0.30212105	-1.32837163	-1.39106768
ENSMUSG00000057880	Abat	4-aminobutyrat	protein_coding	19,338	18,726	20,166	18,109	18,757	17,410	17,886	17,818	17,851	17,876	17,362	16,834	-1.06	0.0573	0.289	-1.09	0.00553	0.0486 down	-1.03	0.384	1	7	2120	1.25799034	0.6161775	2.09562618	-0.05242956	0.64954933	-0.83788189	-0.29964655	-0.37621357	-0.33917324	-0.31143916	-0.89312076	-1.50943864
ENSMUSG00000018677	Slc25a39	solute carrier fa	protein_coding	2,014	1,660	1,978	1,479	1,770	1,582	1,648	1,676	1,549	1,602	1,455	1,438	-1.07	0.196	0.509	-1.18	0.000951	0.0162 down	-1.1	0.0447	1	7	2121	1.86035423	0.07663611	1.69290923	-0.97063168	0.66816794	-0.36394642	0.01363776	0.16609634	-0.55532826	-0.24510997	-1.11813705	-1.22464824
ENSMUSG00000025132	Arhgdia	Rho GDP dissoc	protein_coding	7,244	6,495	7,398	6,297	6,559	6,241	6,754	6,391	6,406	6,235	6,195	6,207	-1.06	0.0744	0.324	-1.1	0.00334	0.0357 down	-1.04	0.254	1	7	2122	1.75297253	-0.07614251	2.10586887	-0.59359553	0.08707521	-0.74361815	0.57964381	-0.3455736	-0.30577184	-0.75898875	-0.86753285	-0.8343372
ENSMUSG00000020349	Ppp2ca	protein phosph	protein_coding	9,222	8,281	9,010	8,143	8,216	7,717	8,253	8,210	7,883	7,859	7,647	7,580	-1.07	0.0359	0.234	-1.12	0.000427	0.00981 down	-1.05	0.157	1	7	2123	2.02630532	0.25301471	1.64319077	-0.02326435	0.12327247	-0.90671886	0.19657447	0.11139587	-0.55741749	-0.60781931	-1.05708007	-1.20145354
ENSMUSG00000022982	Sod1	superoxide dis	protein_coding	6,167	5,578	6,254	5,497	5,574	5,323	5,660	5,511	5,347	5,332	5,146	5,219	-1.06	0.1	0.372	-1.12	0.00366	0.0378 down	-1.05	0.209	1	7	2124	1.77282132	0.10751678	2.00407051	-0.13367831	0.09751332	-0.66591963	0.3488257	-0.09029961	-0.58949454	-0.63753964	-1.22301184	-0.98950406
ENSMUSG00000006932	Ctnnb1	catenin (cadher	protein_coding	13,657	11,708	12,909	10,329	10,871	11,103	10,964	10,953	9,864	10,280	10,054	9,769	-1.11	0.0366	0.236	-1.22	0.0000582	0.00272 down	-1.1	0.054	1	7	2125	2.09612018	0.61398492	1.55343553	-0.58939325	-0.0982616	0.1044991	-0.01655187	-0.02667583	-1.03108908	-0.63458442	-0.84793879	-1.12354489
ENSMUSG00000005354	Txn2	thioredoxin 2	protein_coding	1,757	1,635	1,809	1,502	1,479	1,591	1,580	1,555	1,456	1,504	1,477	1,435	-1.08	0.0808	0.338	-1.14	0.00237	0.0287 down	-1.06	0.203	1	7	2126	1.61632036	0.62785364	2.01191792	-0.52469194	-0.73569541	0.25267842	0.16369623	-0.05486929	-0.9434951	-0.5127166	-0.75642013	-1.14457811
ENSMUSG00000020132	Rab21	RAB21, membe	protein_coding	3,656	3,412	3,654	3,208	3,213	3,350	3,394	3,216	3,210	3,212	3,124	3,116	-1.06	0.0687	0.313	-1.1	0.00177	0.0236 down	-1.04	0.198	1	7	2127	1.82390424	0.55644058	1.81673705	-0.5653335	-0.54039266	0.22023839	0.46211361	-0.52429374	-0.55710586	-0.54436261	-1.05033858	-1.09760692
ENSMUSG00000031823	Zdhhc7	zinc finger, DHH	protein_coding	713	661	822	657	556	604	677	619	581	652	599	559	-1.16	0.0207	0.186	-1.19	0.0055	0.0485 down	-1.03	0.67	1	7	2128	0.98786664	0.30432568	2.30923552	0.25699549	-1.2280265	-0.49440901	0.52617502	-0.2747096	-0.83367077	0.18539291	-0.56435647	-1.1748189
ENSMUSG00000020267	Hint1	histidine triad r	protein_coding	3,339	3,073	3,309	2,887	2,857	2,861	2,959	3,041	2,810	2,937	2,755	2,767	-1.08	0.0622	0.299	-1.12	0.00368	0.0378 down	-1.04	0.305	1	7	2129	1.81189647	0.58223262	1.74724793	-0.39749228	-0.55941093	-0.53726825	-0.00937648	0.42051294	-0.82123535	-0.12654606	-1.13148376	-1.05907684
ENSMUSG00000020978	Klhdc2	kelch domain c	protein_coding	3,950	3,748	4,036	3,636	3,481	3,407	3,498	3,636	3,461	3,567	3,391	3,372	-1.1	0.00959	0.128	-1.11	0.00194	0.0252 down	-1.02	0.628	1	7	2130	1.61568069	0.72047501	1.98039562	0.20392884	-0.54056969	-0.9042606	-0.45564893	0.20179435	-0.63433476	-0.12546453	-0.98271526	-1.07928075
ENSMUSG00000021314	Amph	amphiphysin	protein_coding	4,719	4,492	4,890	4,280	4,187	4,183	4,234	4,267	4,040	4,220	3,975	3,977	-1.09	0.00365	0.084	-1.13	0.0000159	0.00123 down	-1.04	0.168	1	7	2131	1.52240635	0.75233877	2.08108502	-0.00362489	-0.34541507	-0.36002489	-0.17132911	-0.05071601	-0.90320476	-0.22441792	-1.15175861	-1.14533482
ENSMUSG00000009630	Ppp2cb	protein phosph	protein_coding	2,293	2,148	2,360	1,975	2,039	1,933	2,069	2,051	2,025	1,977	1,931	1,870	-1.08	0.0449	0.256	-1.12	0.00307	0.0339 down	-1.04	0.351	1	7	2132	1.60518839	0.66312752	2.0232601	-0.54836237	-0.08964462	-0.85139076	0.12223244	-0.00477305	-0.18846346	-0.53487403	-0.85676296	-1.33053721
ENSMUSG00000056579	Tug1	taurine upregul	protein_coding	6,102	5,695	6,274	5,415	5,426	5,369	5,662	5,574	5,409	5,357	5,421	5,240	-1.07	0.042	0.25	-1.1	0.00315	0.0345 down	-1.03	0.365	1	7	2133	1.66381481	0.40300573	2.17398689	-0.51721126	-0.48200469	-0.67330557	0.29619964	0.00834532	-0.54023101	-0.71570722	-0.49953686	-1.11735578
ENSMUSG00000028152	Tspan5	tetraspanin 5	protein_coding	1,906	1,803	1,961	1,602	1,597	1,553	1,745	1,685	1,570	1,617	1,585	1,609	-1.1	0.00842	0.12	-1.14	0.000421	0.00971 down	-1.03	0.397	1	7	2134	1.59177955	0.88365252	1.95270763	-0.61159369	-0.64791746	-1.0005571	0.47036033	0.02258646	-0.86353	-0.49564278	-0.74277858	-0.55906687
ENSMUSG00000075232	Amd1	S-adenosylmet	protein_coding	3,857	3,838	4,108	3,514	3,462	3,369	3,576	3,578	3,410	3,440	3,493	3,284	-1.09	0.00854	0.121	-1.12	0.000602	0.0121 down	-1.03	0.438	1	7	2135	1.18928269	1.11183987	2.16264035	-0.24531775	-0.47715518	-0.89155527	0.02095383	0.02967721	-0.70652565	-0.57279218	-0.33766592	-1.28338202
ENSMUSG00000041740	Rnf10	ring finger prot	protein_coding	4,896	4,738	4,956	4,196	4,311	4,154	4,519	4,449	4,180	4,255	4,166	4,038	-1.08	0.0592	0.293	-1.13	0.00199	0.0257 down	-1.05	0.232	1	7	2136	1.57044361	1.09154631	1.74892976	-0.6765401	-0.28335867	-0.82028598	0.40384032	0.17671436	-0.72990629	-0.471369	-0.77777956	-1.23223477
ENSMUSG00000044792	Isca1	iron-sulfur clust	protein_coding	3,748	3,631	3,947	3,180	3,110	3,137	3,367	3,377	3,143	3,271	3,107	2,998	-1.12	0.0138	0.154	-1.16	0.000904	0.0156 down	-1.04	0.4	1	7	2137	1.40487235	1.03389127	2.01541134	-0.51545839	-0.7740963	-0.67349186	0.15027932	0.18382121	-0.65087609	-0.18682452	-0.7852524	-1.20227592
ENSMUSG00000022351	Sqle	squalene epoxi	protein_coding	2,227	2,156	2,372	2,122	1,903	1,866	2,246	2,053	2,104	2,105	1,814	1,861	-1.1	0.0233	0.193	-1.12	0.00441	0.0421 down	-1.02	0.581	1	7	2138	0.90021468	0.51961034	1.65026409	0.332068	-0.94365518	-1.17323054	1.00471817	-0.05711486	0.23305908	0.23647281	-1.50047754	-1.20192906
ENSMUSG00000026229	Psmd1	proteasome (pr	protein_coding	4,684	4,597	4,772	4,418	4,070	4,143	4,502	4,325	4,352	4,324	3,979	4,130	-1.08	0.00677	0.109	-1.1	0.00117	0.0184 down	-1.01	0.611	1	7	2139	1.28929664	0.9570708	1.61254928	0.26571404	-1.1665988	-0.85958079	0.59409031	-0.10820468	0.00073778	-0.11021478	-1.56278643	-0.91207336
ENSMUSG00000002844	Adprh	ADP-ribosylargi	protein_coding	1,837	1,775	1,776	1,502	1,372	1,403	1,789	1,472	1,597	1,500	1,307	1,361	-1.14	0.0343	0.23	-1.19	0.00435	0.0418 down	-1.05	0.47	1	7	2140	1.42004226	1.13074908	1.13518629	-0.2466326	-0.98696129	-0.80413792	1.19689604	-0.4143734	0.25451905	-0.25842398	-1.37777734	-1.04908619
ENSMUSG00000054364	Rhob	ras homolog fa	protein_coding	5,082	4,852	5,030	4,195	4,074	4,070	4,863	4,066	4,417	4,300	3,652	3,773	-1.12	0.0585	0.292	-1.19	0.0049	0.045 down	-1.06	0.359	1	7	2141	1.42544321	1.00589258	1.33149681	-0.30861041	-0.57176939	-0.57988406	1.0259348	-0.58886875	0.15611545	-0.0843791	-1.55107731	-1.26029383
ENSMUSG00000014606	Slc25a11	solute carrier fa	protein_coding	2,202	2,139	2,209	1,908	1,768	1,847	2,027	1,835	1,944	1,949	1,618	1,740	-1.13	0.0107	0.137	-1.17	0.00137	0.0204 down	-1.03	0.532	1	7	2142	1.40147097	1.09767828	1.43569745	-0.08939255	-0.8745988	-0.42554391	0.53894816	-0.49344023	0.1008052	0.12967813	-1.78142874	-1.03987394
ENSMUSG00000034353	Ramp1	receptor (calcit	protein_coding	856	825	838	703	679	638	754	677	718	710	574	619	-1.17	0.0101	0.132	-1.23	0.000767	0.0141 down	-1.05	0.451	1	7	2143	1.51814558	1.20796766	1.33868734	-0.10735469	-0.38182403	-0.87592049	0.46940524	-0.40599405	0.06868849	-0.01886423	-1.70045805	-1.11247877
ENSMUSG00000026750	Psmb7	proteasome (pr	protein_coding	1,989	2,076	2,107	1,874	1,826	1,828	1,990	1,766	1,896	1,838	1,714	1,772	-1.09	0.0343	0.23	-1.11	0.00517	0.0465 down	-1.03	0.515	1	7	2144	0.80830912	1.4668973	1.69523394	-0.09513794	-0.49666353	-0.47207773	0.81601203	-1.00032961	0.07643198	-0.39617476	-1.45335257	-0.94914823
ENSMUSG00000068749	Psma5	proteasome sul	protein_coding	976	1,143	1,100	843	871	857	979	921	929	899	728	831	-1.12	0.0844	0.345	-1.2	0.00514	0.0463 down	-1.07	0.291	1	7	2145	0.49808467	1.80656871	1.49044123	-0.68653844	-0.42162902	-0.55485156	0.52813315	0.0329107	0.09770779	-0.16404291	-1.83462973	-0.7921546
ENSMUSG00000029059	Prxl2b	peroxiredoxin l	protein_coding	1,153	1,280	1,320	958	944	887	1,089	955	972	932	785	817	-1.21	0.0115	0.141	-1.34	0.000127	0.00446 down	-1.1	0.196	1	7	2146	0.89779285	1.55457451	1.7499286	-0.24211886	-0.33399627	-0.71077111	0.54054155	-0.26125224	-0.15480052	-0.40923039	-1.43273967	-1.19792799
ENSMUSG00000035530	Eif1	eukaryotic tran	protein_coding	5,862	6,444	6,745	5,299	5,294	5,217	5,702	5,468	5,407	5,369	4,922	4,965	-1.12	0.0492	0.27	-1.18	0.00529	0.0472 down	-1.05	0.413	1	7	2147	0.59980517	1.58966943	2.06825956	-0.45317361	-0.46230625	-0.61608453	0.31052576	-0.12644411	-0.24386838	-0.31676414	-1.22092663	-1.12869227
ENSMUSG00000030662	Ipo5	importin 5	protein_coding	3,996	4,142	4,301	3,881	3,843	3,848	4,036	3,866	3,808	3,742	3,532	3,610	-1.05	0.0991	0.37	-1.11	0.000118	0.00425 down	-1.06	0.0285	1	7	2148	0.54292898	1.19964681	1.88743293	0.01089705	-0.16797344	-0.14392307	0.72386735	-0.06030307	-0.33539593	-0.65408761	-1.69827923	-1.30481077
ENSMUSG00000044533	Rps2	ribosomal prot	protein_coding	5,868	6,226	6,661	5,368	5,324	5,235	6,101	5,497	5,350	5,457	4,585	4,833	-1.09	0.14	0.434	-1.19	0.00218	0.0273 down	-1.1	0.113	1	7	2149	0.59090523	1.15521061	1.8013521	-0.2574594	-0.33531735	-0.49437007	0.96105582	-0.03063345	-0.28823992	-0.10045709	-1.75046571	-1.25158077
ENSMUSG00000060036	Rpl3	ribosomal prot	protein_coding	14,887	15,448	16,648	13,161	12,941	12,950	14,808	13,695	13,810	13,531	11,767	12,026	-1.11	0.0722	0.32	-1.18	0.00366	0.0378 down	-1.06	0.269	1	7	2150	0.78866315	1.15280759	1.89040439	-0.4242081	-0.59029185	-0.58349603	0.73590317	-0.03324113	0.04919147	-0.15180037	-1.52383906	-1.31009352
ENSMUSG00000028452	Vcp	valosin contain	protein_coding	9,826	10,016	10,233	9,232	9,230	8,918	9,650	9,337	9,315	9,147	8,412	8,458	-1.06	0.0413	0.249	-1.11	0.000117	0.00423 down	-1.05	0.0712	1	7	2151	0.9107543	1.22810121	1.58366045	-0.12019757	-0.12437095	-0.69109443	0.61189288	0.06745482	0.02727181	-0.27198713	-1.65612256	-1.56536282
ENSMUSG00000058569	Tmed9	transmembran	protein_coding	1,438	1,463	1,615	1,334	1,334	1,280	1,404	1,328	1,313	1,281	1,172	1,178	-1.09	0.0701	0.316	-1.18	0.000607	0.0121 down	-1.08	0.109	1	7	2152	0.78092601	0.97920621	2.0880666	-0.0546844	-0.05194052	-0.5124823	0.51696482	-0.10578211	-0.23097649	-0.50636758	-1.4765505	-1.42637975
ENSMUSG00000003970	Rpl8	ribosomal prot	protein_coding	6,975	7,021	7,638	6,242	6,162	6,015	6,836	6,370	6,109	6,187	5,445	5,507	-1.1	0.0678	0.311	-1.2	0.00039	0.00925 down	-1.09	0.0859	1	7	2153	0.94948041	1.01615689	1.86679268	-0.16871237	-0.29845468	-0.54090543	0.74633514	0.03557052	-0.38491808	-0.2576621	-1.53813517	-1.42554782
ENSMUSG00000024425	Ndfip1	Nedd4 family ir	protein_coding	13,508	13,611	14,704	12,369	11,960	11,997	13,141	12,351	12,285	12,181	10,702	10,795	-1.1	0.0903	0.354	-1.18	0.0023	0.0282 down	-1.08	0.176	1	7	2154	0.91810317	1.00122394	1.84777119	-0.04478911	-0.41254114	-0.37841843	0.61656462	-0.06105817	-0.11975701	-0.21199471	-1.62486424	-1.5302401
ENSMUSG00000015776	Med22	mediator comp	protein_coding	611	589	692	552	506	532	621	553	562	511	469	453	-1.1	0.165	0.47	-1.22	0.00409	0.0401 down	-1.11	0.141	1	7	2155	0.85957492	0.54611778	1.92586925	0.01094785	-0.69992905	-0.28842936	0.99647975	0.02279491	0.15630321	-0.61678066	-1.31553702	-1.59741157
ENSMUSG00000045912	C2cd4c	C2 calcium-dep	protein_coding	712	700	798	615	582	591	695	604	648	608	558	563	-1.14	0.0239	0.195	-1.19	0.00286	0.0323 down	-1.04	0.496	1	7	2156	1.02755085	0.86678393	2.09213577	-0.31812998	-0.80683162	-0.66580898	0.80026884	-0.47003341	0.15394047	-0.41834956	-1.16937008	-1.09215621

ENSMUSG00000051232	Tmem199	transmembrane	protein_coding	802	883	859	771	730	723	859	813	740	764	645	708	-1.06	0.281
ENSMUSG00000106037	Gm4332	predicted gene	processed_pseudogene	691	845	774	605	552	495	705	702	575	634	430	459	-1.19	0.128
ENSMUSG00000001525	Tubb5	tubulin, beta 5	protein_coding	12,506	12,174	12,640	10,792	10,525	10,448	11,960	11,024	10,967	10,829	10,098	9,919	-1.09	0.0161
ENSMUSG00000008036	Ap2s1	adaptor-related	protein_coding	1,898	1,788	1,973	1,527	1,549	1,462	1,748	1,609	1,597	1,598	1,411	1,366	-1.13	0.0589
ENSMUSG00000028692	Akr1a1	aldo-keto reduc	protein_coding	5,971	5,782	6,277	5,243	5,027	5,067	5,894	5,220	5,298	5,363	4,788	4,771	-1.1	0.0487
ENSMUSG00000093674	Rpl41	ribosomal prote	protein_coding	5,806	5,677	6,131	4,721	4,750	4,538	5,642	4,821	5,013	5,077	4,249	4,043	-1.13	0.0642
ENSMUSG00000067274	Rplp0	ribosomal prote	protein_coding	3,866	3,737	3,904	3,294	3,282	3,331	3,827	3,588	3,339	3,454	3,117	3,113	-1.05	0.229
ENSMUSG00000039105	Atp6v1g1	ATPase, H+ trar	protein_coding	1,985	1,963	2,107	1,693	1,750	1,620	1,976	1,783	1,718	1,805	1,584	1,578	-1.09	0.108
ENSMUSG00000039530				3,347	3,355	3,705	2,853	2,929	2,695	3,464	3,102	2,924	3,019	2,467	2,478	-1.09	0.228
ENSMUSG00000032959	Pebp1	phosphatidyletl	protein_coding	6,252	6,435	6,469	5,113	5,205	5,052	5,707	5,463	5,344	5,351	4,517	4,471	-1.13	0.0612
ENSMUSG00000006273	Atp6v1b2	ATPase, H+ trar	protein_coding	13,167	13,620	13,456	11,781	11,336	11,126	12,345	11,986	11,953	11,813	10,636	10,490	-1.11	0.0334
ENSMUSG00000029621	Arpc1a	actin related pr	protein_coding	2,750	2,867	2,878	2,464	2,355	2,331	2,634	2,499	2,542	2,519	2,151	2,141	-1.12	0.047
ENSMUSG00000062933	Gm10123	predicted pseu	processed_pseudogene	6,464	6,886	7,149	5,693	5,655	5,704	6,240	6,445	5,932	5,916	5,007	5,023	-1.09	0.153
ENSMUSG00000076437	Selenoh	selenoprotein H	protein_coding	320	359	367	284	272	265	305	310	295	290	222	242	-1.15	0.0877
ENSMUSG00000035078	Mtmr9	myotubularin r	protein_coding	2,144	2,331	2,334	1,980	2,018	1,840	2,134	2,065	2,052	1,990	1,813	1,749	-1.09	0.0771
ENSMUSG00000040952	Rps19	ribosomal prote	protein_coding	2,791	3,195	3,087	2,371	2,459	2,186	2,944	2,489	2,496	2,426	2,168	2,055	-1.14	0.11
ENSMUSG00000025870	Arl10	ADP-ribosylatio	protein_coding	925	959	1,002	749	799	723	897	766	788	810	673	683	-1.14	0.0438
ENSMUSG00000028691	Prdx1	peroxiredoxin 1	protein_coding	3,124	3,297	3,335	2,761	2,733	2,556	3,071	2,699	2,811	2,842	2,380	2,237	-1.13	0.0457
ENSMUSG00000025351	Cd63	CD63 antigen	protein_coding	1,532	1,589	1,576	1,249	1,367	1,322	1,543	1,430	1,385	1,364	1,173	1,141	-1.05	0.406
ENSMUSG00000069972	Rps13-ps2	ribosomal prote	processed_pseudogene	1,255	1,316	1,435	1,013	996	1,023	1,360	1,063	1,132	1,067	922	777	-1.13	0.162
ENSMUSG00000027963	Extl2	exostosin-like g	protein_coding	2,195	2,087	2,286	1,958	1,927	2,130	2,129	1,888	1,889	1,914	1,683	1,855	-1.06	0.218
ENSMUSG00000058655	Eif4b	eukaryotic tran	protein_coding	8,743	8,296	8,909	8,167	8,268	8,422	8,430	8,086	8,235	8,089	7,633	7,886	-1.03	0.26
ENSMUSG00000015932	Dstn	destrin	protein_coding	3,879	3,529	3,882	3,425	3,405	3,499	3,776	3,533	3,469	3,336	3,188	3,251	-1.04	0.318
ENSMUSG00000025967	Eef1b2	eukaryotic tran	protein_coding	3,094	2,816	3,089	2,745	2,794	2,632	2,992	2,857	2,681	2,746	2,527	2,551	-1.04	0.308
ENSMUSG00000022108	Itm2b	integral membr	protein_coding	17,043	15,713	17,144	15,429	15,204	15,199	16,597	15,652	14,658	14,864	14,042	14,109	-1.04	0.172
ENSMUSG00000030224	Strap	serine/threonir	protein_coding	3,199	3,023	3,241	2,909	2,697	2,733	3,112	2,891	2,694	2,722	2,523	2,569	-1.08	0.058
ENSMUSG00000018446	C1qbp	complement co	protein_coding	1,225	1,181	1,199	1,081	1,089	1,114	1,127	1,081	1,071	1,083	987	983	-1.06	0.13
ENSMUSG00000039501	Znfx1	zinc finger, NFX	protein_coding	1,773	1,677	1,764	1,522	1,440	1,578	1,609	1,536	1,532	1,436	1,404	1,312	-1.09	0.0568
ENSMUSG00000007836	Hnrnpa0	heterogeneous	protein_coding	3,688	3,591	3,962	3,282	3,262	3,260	3,458	3,225	3,221	3,310	2,852	2,908	-1.1	0.0393
ENSMUSG00000021610	Clptm1l	CLPTM1-like	protein_coding	2,086	2,045	2,294	1,816	1,829	1,928	1,972	1,846	1,864	1,963	1,596	1,628	-1.09	0.134
ENSMUSG00000022661	Cd200	CD200 molecul	protein_coding	5,344	5,328	5,699	4,687	4,746	4,802	5,333	5,144	4,933	4,860	4,300	4,358	-1.05	0.296
ENSMUSG00000006333	Rps9	ribosomal prote	protein_coding	5,420	5,362	5,770	4,840	4,806	4,866	5,293	4,951	4,846	4,798	4,393	4,268	-1.07	0.111
ENSMUSG00000009927	Rps25	ribosomal prote	protein_coding	4,379	4,245	4,639	3,735	3,810	3,772	4,210	3,948	3,848	3,888	3,487	3,423	-1.08	0.138
ENSMUSG00000022336	Eif3e	eukaryotic tran	protein_coding	2,126	2,025	2,126	1,832	1,856	1,823	2,025	1,882	1,899	1,835	1,688	1,729	-1.07	0.138
ENSMUSG00000057841	Rpl32	ribosomal prote	protein_coding	3,544	3,375	3,548	3,003	3,115	3,044	3,386	3,145	3,121	3,160	2,760	2,787	-1.06	0.188
ENSMUSG00000051234	Rnf7	ring finger prot	protein_coding	1,633	1,586	1,662	1,449	1,417	1,458	1,563	1,446	1,440	1,452	1,335	1,355	-1.08	0.103
ENSMUSG00000061111	Mcrip1	MAPK regulated	protein_coding	1,099	1,001	1,124	927	921	938	1,009	924	943	938	822	836	-1.09	0.0762
ENSMUSG00000021114	Atp6v1d	ATPase, H+ trar	protein_coding	4,485	4,452	4,796	4,243	4,218	4,034	4,531	4,294	4,019	4,277	3,794	3,943	-1.05	0.198
ENSMUSG00000047675	Rps8	ribosomal prote	protein_coding	6,408	6,390	7,073	6,059	6,073	5,605	6,376	6,042	5,739	5,916	4,969	5,241	-1.08	0.176
ENSMUSG00000056665	Them6	thioesterase su	protein_coding	552	555	601	501	487	479	528	499	476	497	419	480	-1.11	0.0685
ENSMUSG00000024150	Mcfd2	multiple coagul	protein_coding	2,387	2,384	2,520	2,204	2,177	2,116	2,307	2,184	2,084	2,182	1,969	2,048	-1.08	0.0656
ENSMUSG00000078812	Eif5a	eukaryotic tran	protein_coding	4,813	4,881	5,199	4,425	4,364	4,085	4,585	4,312	4,180	4,337	3,845	4,093	-1.11	0.00359
ENSMUSG00000021660	Btf3	basic transcript	protein_coding	2,562	2,590	2,735	2,347	2,417	2,416	2,557	2,353	2,317	2,397	2,160	2,286	-1.05	0.21
ENSMUSG00000030754	Copb1	coatomer prote	protein_coding	2,054	1,992	2,118	1,901	1,942	1,923	2,015	1,912	1,874	1,856	1,773	1,790	-1.04	0.239
ENSMUSG00000031447	Lamp1	lysosomal-asso	protein_coding	10,726	10,516	11,104	9,568	10,011	9,667	10,788	9,647	9,662	9,483	8,861	9,234	-1.04	0.171
ENSMUSG00000036155	Mgat5	mannoside ace	protein_coding	4,135	4,144	4,419	4,058	4,020	3,859	4,139	3,803	3,964	3,920	3,747	3,798	-1.06	0.0511
ENSMUSG00000027828	Ssr3	signal sequence	protein_coding	4,828	4,580	5,229	4,357	4,504	4,361	4,682	4,325	4,435	4,315	4,070	4,137	-1.06	0.13
ENSMUSG00000100755	Rps23-ps1	ribosomal prote	processed_pseudogene	3,686	3,478	4,198	3,403	3,477	3,234	3,682	3,263	3,331	3,235	2,888	3,120	-1.08	0.144
ENSMUSG00000030772	Dkk3	dickkopf WNT s	protein_coding	2,186	2,195	2,342	2,138	2,105	2,110	2,250	2,027	2,089	1,957	1,871	1,899	-1.04	0.22
ENSMUSG00000025544	Tm9sf2	transmembrane	protein_coding	4,161	4,255	4,445	4,072	4,087	3,886	4,314	3,928	3,930	3,884	3,586	3,464	-1.04	0.214
ENSMUSG00000028138	Adh5	alcohol dehydro	protein_coding	1,054	1,032	1,102	975	975	920	1,049	974	961	905	785	827	-1.06	0.239
ENSMUSG00000060743	H3f3a	H3.3 histone A	protein_coding	2,851	2,816	3,089	2,530	2,618	2,562	2,949	2,564	2,561	2,506	2,054	2,185	-1.06	0.345
ENSMUSG00000067870	Rpl31-ps8	ribosomal prote	processed_pseudogene	2,693	2,725	3,026	2,587	2,556	2,503	2,769	2,627	2,398	2,460	2,387	2,277	-1.05	0.272
ENSMUSG00000078974	Sec61g	SEC61, gamma	protein_coding	194	217	258	183	190	165	234	189	171	174	155	152	-1.09	0.335
ENSMUSG00000025579	Gaa	glucosidase, alp	protein_coding	31,611	31,772	33,170	30,840	29,231	30,138	32,744	30,311	29,473	29,796	28,479	29,031	-1.04	0.204
ENSMUSG00000006373	Pgrmc1	progesterone r	protein_coding	12,519	12,204	13,188	11,472	10,971	11,205	13,012	11,422	11,278	11,351	10,222	10,438	-1.06	0.178
ENSMUSG00000037742	Eef1a1	eukaryotic tran	protein_coding	67,929	67,442	72,242	62,743	62,467	62,803	70,801	65,820	62,658	62,623	56,731	57,925	-1.03	0.404
ENSMUSG00000058927	Gm10053	predicted gene	protein_coding	1,704	1,765	1,880	1,606	1,553	1,606	1,653	1,821	1,553	1,488	1,471	1,536	-1.05	0.291
ENSMUSG00000024683	Mrpl16	mitochondrial r	protein_coding	425	444	456	381	370	359	411	408	379	363	329	391	-1.1	0.0903
ENSMUSG00000069682	Gm10275	predicted pseu	transcribed_processed	3,765	3,649	3,971	3,271	3,232	3,261	3,514	3,558	3,215	3,261	2,972	3,161	-1.08	0.14
ENSMUSG00000032324	Tspan3	tetraspanin 3	protein_coding	6,650	6,302	7,045	6,000	5,918	6,032	6,196	6,101	5,936	5,743	5,477	5,871	-1.07	0.0829
ENSMUSG00000049036	Tmem121	transmembrane	protein_coding	284	257	290	215	205	219	242	222	214	205	187	202	-1.18	0.0344
ENSMUSG00000022421	Nptxr	neuronal pentra	protein_coding	10,012	9,934	10,015	9,181	9,016	8,980	9,807	9,385	8,820	9,159	8,810	9,153	-1.05	0.0845
ENSMUSG00000003316	Glg1	golgi apparatus	protein_coding	9,870	9,979	10,201	9,332	9,088	9,102	9,694	9,609	8,798	8,968	8,993	9,045	-1.05	0.0438
ENSMUSG00000031016	Wee1	WEE 1 homolog	protein_coding	540	572	584	486	489	476	564	516	458	473	477	482	-1.07	0.213
ENSMUSG00000060126	Tpt1	tumor protein,	protein_coding	7,385	7,064	7,917	6,059	6,378	6,265	7,432	7,247	5,940	6,501	5,716	5,931	-1.04	0.477
ENSMUSG00000068391	Chrac1	chromatin acce	protein_coding	258	253	257	226	217	215	261	237	191	215	183	203	-1.07	0.379
ENSMUSG00000020038	Cry1	cryptochrome 1	protein_coding	592	629	669	547	465	543	571	566	514	552	490	502	-1.13	0.0297
ENSMUSG00000020520	Galnt10	polypeptide N-a	protein_coding	864	848	892	740	687	794	800	816	745	761	703	736	-1.08	0.0926
ENSMUSG00000035666	Gtf3c4	general transcri	protein_coding	2,048	2,087	2,099	1,860	1,769	1,877	2,036	1,949	1,830	1,873	1,771	1,809	-1.06	0.1
ENSMUSG00000042628	Zfyve1	zinc finger, FYV	protein_coding	1,360	1,376	1,460	1,084	948	1,136	1,380	1,108	1,024	1,048	906	1,020	-1.16	0.0533
ENSMUSG00000044030	Irf2bp1	interferon regu	protein_coding	1,386	1,385	1,414	1,163	1,173	1,196	1,343	1,214	1,147	1,175	1,108	1,145	-1.09	0.0967
ENSMUSG00000071657	Bscl2	Berardinelli-Sei	protein_coding	2,181	2,226	2,162	1,803	1,841	1,915	2,062	1,945	1,838	1,900	1,805	1,775	-1.08	0.109
ENSMUSG00000028964	Park7	Parkinson disea	protein_coding	1,924	2,002	2,129	1,890	1,743	1,752	1,914	1,829	1,785	1,794	1,728	1,724	-1.1	0.00713
ENSMUSG00000031007	Atp6ap2	ATPase, H+ trar	protein_coding	5,068	5,055	5,365	4,861	4,543	4,457	4,853	4,771	4,620	4,663	4,457	4,434	-1.09	0.00612
ENSMUSG00000022454	Nell2	NEL-like 2	protein_coding	5,400	5,358	5,606	5,096	4,668	4,704	4,937	4,927	4,944	4,732	4,633	4,463	-1.12	0.00281
ENSMUSG00000031227	Magee1	MAGE family m	protein_coding	7,066	7,194	7,076	6,768	6,351	6,290	6,801	6,641	6,482	6,377	6,256	6,100	-1.08	0.0171
ENSMUSG00000002957	Ap2a2	adaptor-related	protein_coding	5,858	5,937	5,750	5,276	5,075	4,874	5,458	5,342	5,214	5,185	4,930	4,992	-1.1	0.00562
ENSMUSG00000021957	Tkt	transketolase	protein_coding	5,961	5,861	5,820	5,311	5,209	4,963	5,606	5,484	5,275	5,216	4,837	5,078	-1.08	0.0149
ENSMUSG00000040652	Oaz2	ornithine decar	protein_coding	4,323	4,266	4,526	3,887	3,683	3,577	4,095	4,028	3,797	3,891	3,575	3,570	-1.1	0.0172
ENSMUSG00000047921	Trappc9	trafficking prot	protein_coding	2,238	2,174	2,294	2,019	1,828	1,819	2,103	2,038	1,945	1,900	1,824	1,740	-1.12	0.0184
ENSMUSG00000006299	Aamp	angio-associate	protein_coding	3,268	3,231	3,277	2,971	2,828	2,814	3,250	3,044	2,861	2,893	2,803	2,765	-1.07	0.0639
ENSMUSG00000007777	0610009B22Rik	RIKEN cDNA 06	protein_coding	392	396	387	329	310	292	374	346	314	332	302	278	-1.13	0.0512
ENSMUSG00000052833	Sae1	SUMO1 activati	protein_coding	1,871	2,003	1,888	1,595	1,532	1,337	1,724	1,729	1,594	1,490	1,583	1,416	-1.16	0.0233
ENSMUSG00000001416	Cct3	chaperonin con	protein_coding	3,167	3,362	3,374	2,766	2,930	2,697	3,145	2,975	2,883	2,778	2,781	2,724	-1.08	0.0822
ENSMUSG00000022426	Josd1	Josephin domai	protein_coding	935	1,012	1,008	868	856	785	961	901	828	823	834	816	-1.09	0.0681
ENSMUSG00000043881	Kbtbd7	kelch repeat an	protein_coding	1,525	1,601	1,668	1,417	1,379	1,322	1,503	1,491	1,404	1,344	1,383	1,324	-1.09	0.0514
ENSMUSG00000023147	Get1	guided entry of	protein_coding	2,154	2,200	2,292	1,946	1,890	1,825	2,070	1,986	1,890	1,895	1,844	1,712	-1.1	0.0323
ENSMUSG00000044894	Uqcrq	ubiquinol-cytoc	protein_coding	1,228	1,325	1,337	1,067	1,023	965	1,104	1,084	1,019	1,033	979	890	-1.19	0.0136
ENSMUSG00000007950	Abhd8	abhydrolase do	protein_coding	4,080	4,326	4,513	3,382	3,509	3,408	3,802	3,600	3,470	3,488	3,301	3,082	-1.14	0.0422
ENSMUSG00000070493	Chchd2	coiled-coil-helix	protein_coding	3,347	3,406	3,616	2,704	2,736	2,530	2,983	2,798	2,836	2,746	2,601	2,375	-1.18	0.0284
ENSMUSG00000041881	Ndufa7	NADH:ubiquino	protein_coding	987	1,007	1,004	778	789	724	878	843	817	799	725	659	-1.17	0.0335
ENSMUSG00000026123	Plekhb2	pleckstrin homo	protein_coding	9,847	9,590	9,760	8,415	8,385	8,082	8,839	8,677	8,492	8,436	7,890	7,659	-1.11	0.0317
ENSMUSG00000033629	Hacd3	3-hydroxyacyl-(	protein_coding	6,227	6,103	6,273	5,610	5,622	5,398	5,891	5,744	5,613	5,616	5,397	5,225	-1.07	0.0432
ENSMUSG00000030654	Arl6ip1	ADP-ribosylatio	protein_coding	6,380	6,491	6,901	5,577	5,721	5,249	6,050	5,971	5,642	5,656	5,183	5,044	-1.1	0.0752
ENSMUSG00000074748	Atxn7l3b	ataxin 7-like 3B	protein_coding	4,581	4,651	4,850	4,149	4,112	3,792	4,341	4,267	4,083	3,990	3,830	3,814	-1.1	0.0245
ENSMUSG00000007739	Cct4	chaperonin con	protein_coding	3,358	3,318	3,358	2,813	2,964	2,651	3,188	2,957	2,818	2,844	2,867	2,658	-1.09	0.0568
ENSMUSG00000051695	Pcbp1	poly(rC) binding	protein_coding	4,529	4,307	4,563	3,863	3,919	3,664	4,391	3,934	3,796	3,904	3,876	3,626	-1.09	0.0424
ENSMUSG00000025791	Pgm1	phosphoglucom	protein_coding	3,157	3,222	3,258	2,866	2,737	2,746	3,063	2,875	2,842	2,866	2,856	2,534	-1.09	0.0171
ENSMUSG00000030007	Cct7	chaperonin con	protein_coding	4,695	4,640	4,714	4,225	3,977	3,847	4,455	4,058	4,002	3,966	4,031	3,722	-1.12	0.00581
ENSMUSG00000017978	Cadps2	Ca2+-depender	protein_coding	2,799	2,977	2,994	2,676	2,666	2,416	3,006	2,588	2,507	2,621	2,516	2,382	-1.07	0.141
ENSMUSG00000061983	Rps12	ribosomal prote	protein_coding	2,123	2,159	2,280	1,811	1,865	1,738	2,232	1,802	1,758	1,851	1,763	1,725	-1.1	0.0954
ENSMUSG00000035585	Tsen34	tRNA splicing e	protein_coding	1,438	1,438	1,528	1,286	1,326	1,222	1,418	1,247	1,229	1,285	1,218	1,182	-1.09	0.07
ENSMUSG00000051615	Rap2a	RAS related pro	protein_coding	2,833	2,767	3,030	2,503	2,515	2,391	2,794	2,516	2,408	2,463	2,302	2,349	-1.09	0.107
ENSMUSG00000001663	Gstt1	glutathione S-t	protein_coding	393	376	380	289	348	305	412	299	287	326	269	254	-1.06	0.5
ENSMUSG00000060419	Rps16-ps2	ribosomal prote	processed_pseudogene	1,836	1,670	1,887	1,497	1,452	1,577	1,908	1,485	1,411	1,617	1,386	1,174	-1.07	0.331
ENSMUSG00000067578	Cbln4	cerebellin 4 pre	protein_coding	1,030	1,005	1,046	958	902	903	1,096	898	962	842	826	835	-1.06	0.233
ENSMUSG00000006651	Aplp1	amyloid beta (A	protein_coding	24,061	22,033	24,186	20,928	20,371	20,294	23,154	20,361	20,814	20,753	19,677	19,565	-1.08	0.0379
ENSMUSG00000007458	M6pr	mannose-6-pho	protein_coding	3,463	3,330	3,501	3,154	3,107	3,147	3,398	3,120	3,119	3,102	3,013	2,968	-1.05	0.0703
ENSMUSG00000034300	Fam53c	family with seq	protein_coding	1,585	1,580	1,645	1,435	1,378	1,379	1,642	1,355	1,419	1,365	1,350	1,331	-1.09	0.0669
ENSMUSG00000117310	Ptp4a1	protein tyrosin	protein_coding	3,406	3,221	3,495	3,057	3,039	2,899	3,364	2,960	3,023	2,913	2,955	2,908	-1.07	0.0682
ENSMUSG00000041773	Enc1	ectodermal-neu	protein_coding	5,094	5,147	5,166	4,670	4,187	4,487	5,223	4,629	4,428	4,293	4,712	4,248	-1.08	0.044
ENSMUSG00000005803	Sqor	sulfide quinone	protein_coding	267	227	269	208	163	179	244	178	179	167	188	134	-1.28	0.0677
ENSMUSG00000021770	Samd8	sterile alpha mo	protein_coding	2,431	2,303	2,410	2,294	2,048	2,079	2,314	2,111	2,120	1,966	2,017	1,985	-1.1	0.00281
ENSMUSG00000024231	Cul2	cullin 2	protein_coding	2,082	2,128	2,280	2,269	2,044	2,084	2,166	2,247	2,010	2,042	1,884	1,922	-1.02	0.485
ENSMUSG00000022526	Zfp251	zinc finger prot	protein_coding	1,081	1,044	1,214	1,124	1,019	1,053	1,043	1,062	951	1,032	968	966	-1.07	0.123
ENSMUSG00000050628	Ubald2	UBA-like domai	protein_coding	289	287	361	319	285	250	267	290	236	267	239	250	-1.14	0.0834
ENSMUSG00000028646	Rragc	Ras-related GTF	protein_coding	2,080	2,213	2,270	2,189	1,862	1,753	2,197	2,015	1,908	1,931	1,809	1,892	-1.12	0.0228
ENSMUSG00000024742	Fen1	flap structure s	protein_coding	247	247	283	247	210	181	241	235	218	197	171	202	-1.17	0.0238
ENSMUSG00000031897	Psmb10	proteasome (pr	protein_coding	498	485	521	519	428	380	503	460	434	432	360	406	-1.14	0.0732
ENSMUSG00000037253	Mex3c	mex3 RNA bind	protein_coding	819	846	968	807	860	731	853	822	796	702	749	720	-1.05	0.304
ENSMUSG00000038622	Med30	mediator comp	protein_coding	365	378	464	400	379	341	406	395	355	344	322	336	-1.05	0.392
ENSMUSG00000040133	Gpr176	G protein-coupl	protein_coding	576	561	682	598	536	482	557	562	556	524	484	462	-1.13	0.0418
ENSMUSG00000036885	Arhgef26	Rho guanine nu	protein_coding	1,296	1,478	1,679	1,092	1,143	1,222	1,250	1,204	1,238	1,148	1,029	1,114	-1.15	0.0388
ENSMUSG00000073755	5730409E04Rik	RIKEN cDNA 57	protein_coding	1,708	1,753	1,894	1,552	1,520	1,561	1,552	1,580	1,548	1,518	1,456	1,545	-1.11	0.00623
ENSMUSG00000024151	Msh2	mutS homolog	protein_coding	1,180	1,244	1,386	1,175	1,130	1,125	1,155	1,180	1,107	1,135	1,106	1,101	-1.08	0.0417
ENSMUSG00000045328	Cenpe	centromere pro	protein_coding	22	56	80	19	23	20	17	28	16	16	13	22	-1.79	0.0554
ENSMUSG00000050029	Rap2c	RAP2C, membe	protein_coding	969	994	1,023	911	900	880	906	846	900	859	892	796	-1.1	0.0232
ENSMUSG00000028675	Pnrc2	proline-rich nuc	protein_coding	1,401	1,576	1,821	1,288	1,266	1,258	1,141	1,211	1,155	1,054	1,205	1,104	-1.25	0.00464
ENSMUSG00000036949	Slc39a12	solute carrier fa	protein_coding	2,042	1,953	2,245	1,929	1,815	1,828	1,806	1,750	1,845	1,696	1,813	1,686	-1.13	0.00426
ENSMUSG00000020646	Mboat2	membrane bou	protein_coding	2,443	2,612	2,630	2,389	2,405	2,358	2,300	2,206	2,375	2,304	2,114	2,205	-1.09	0.0157
ENSMUSG00000002741	Ykt6	YKT6 v-SNARE	protein_coding	1,548	1,620	1,723	1,529	1,467	1,432	1,472	1,488	1,483	1,370	1,382	1,463	-1.09	0.0105
ENSMUSG00000024601	Isoc1	isochorismatase	protein_coding	1,326	1,416	1,408	1,303	1,243	1,179	1,178	1,231	1,200	1,189	1,162	1,165	-1.13	0.0018
ENSMUSG00000032281	Acsbg1	acyl-CoA synthe	protein_coding	4,282	4,507	4,809	3,982	3,908	3,841	3,775	3,879	3,896	3,807	3,707	3,581	-1.14	0.00848
ENSMUSG00000000339	Rtca	RNA 3'-termina	protein_coding	824	824	867	798	758	776	759	820	802	738	623	705	-1.06	0.221
ENSMUSG00000034501	Pcnx4	pecanex homol	protein_coding	1,526	1,577	1,626	1,499	1,391	1,484	1,422	1,513	1,438	1,426	1,303	1,344	-1.07	0.116
ENSMUSG00000032564	Cpne4	copine IV	protein_coding	829	952	1,042	915	701	898	791	765	786	786	655	697	-1.19	0.00436
ENSMUSG00000028945	Rheb	Ras homolog er	protein_coding	2,115	2,187	2,277	2,113	2,013	2,069	1,959	1,982	2,096	1,930	1,895	1,862	-1.08	0.0205
ENSMUSG00000001891	Ugp2	UDP-glucose py	protein_coding	1,966	1,969	2,162	1,941	1,758	1,868	1,795	1,855	1,874	1,751	1,641	1,572	-1.1	0.0267
ENSMUSG00000051359	Ncald	neurocalcin del	protein_coding	10,656	10,183	11,332	10,250	9,490	9,766	9,625	9,869	10,018	9,649	9,136	9,148	-1.09	0.00931
ENSMUSG00000005802	Slc30a4	solute carrier fa	protein_coding	1,297	1,410	1,491	1,369	1,242	1,266	1,240	1,260	1,295	1,340	1,126	1,142	-1.11	0.0116
ENSMUSG00000032932	Hspa13	heat shock prot	protein_coding	2,472	2,689	2,681	2,450	2,302	2,265	2,398	2,384	2,315	2,480	2,110	2,122	-1.1	0.0393
ENSMUSG00000058600	Rpl30	ribosomal prote	protein_coding	2,449	2,604	2,864	2,408	2,399	2,289	2,418	2,424	2,293	2,384	2,131	2,036	-1.08	0.102
ENSMUSG00000035478	Mbd3	methyl-CpG bin	protein_coding	1,701	1,848	2,043	1,671	1,612	1,520	1,707	1,633	1,595	1,600	1,512	1,482	-1.12	0.0109
ENSMUSG00000016940	Kctd2	potassium chan	protein_coding	1,235	1,388	1,402	1,169	1,164	1,055	1,195	1,109	1,121	1,113	1,029	1,011	-1.15	0.0145
ENSMUSG00000048807	Slc35e4	solute carrier fa	protein_coding	538	585	633	535	537	468	538	496	497	503	452	429	-1.12	0.0555
ENSMUSG00000000751	Rpa1	replication prot	protein_coding	1,597	1,574	1,683	1,404	1,470	1,531	1,407	1,479	1,459	1,392	1,325	1,242	-1.06	0.157
ENSMUSG00000024208	Uqcc2	ubiquinol-cytoc	protein_coding	954	900	940	736	746	788	745	787	802	737	712	648	-1.15	0.0275
ENSMUSG00000054843	Atrnl1	attractin like 1	protein_coding	2,011	2,030	2,177	1,812	1,739	1,918	1,735	1,788	1,907	1,754	1,700	1,583	-1.12	0.0216
ENSMUSG00000025139	Tollip	toll interacting	protein_coding	4,065	4,075	4,366	3,575	3,528	3,518	3,858	3,788	3,684	3,469	3,390	3,415	-1.09	0.035
ENSMUSG00000031021	Tmem9b	TMEM9 domain	protein_coding	2,003	1,943	2,057	1,719	1,709	1,714	1,833	1,806	1,847	1,666	1,650	1,568	-1.09	0.0521
ENSMUSG00000027698	Nceh1	neutral cholest	protein_coding	3,685	3,626	3,827	3,431	3,274	3,374	3,448	3,404	3,443	3,319	3,093	3,152	-1.08	0.0143
ENSMUSG00000036766	Dner	delta/notch-like	protein_coding	8,114	8,185	8,745	7,313	6,866	7,247	7,453	7,497	7,343	7,219	6,513	6,328	-1.11	0.0125
ENSMUSG00000020152	Actr2	ARP2 actin-rela	protein_coding	5,678	5,657	6,112	5,140	4,984	5,126	5,195	5,242	5,414	5,096	4,861	4,855	-1.1	0.017
ENSMUSG00000029126	Nsg1	neuron specific	protein_coding	9,100	9,222	9,750	8,008	7,658	7,762	8,188	8,194	8,031	7,922	7,437	7,256	-1.13	0.0057
ENSMUSG00000063358	Mapk1	mitogen-activat	protein_coding	8,891	8,943	9,301	8,365	7,578	7,770	8,761	8,011	7,830	8,196	7,355	7,035	-1.11	0.01
ENSMUSG00000058603	Rpl28-ps1	ribosomal prote	processed_pseudogene	1,779	1,761	2,061	1,610	1,506	1,487	1,716	1,628	1,581	1,603	1,418	1,238	-1.14	0.0416
ENSMUSG00000031696	Vps35	VPS35 retromer	protein_coding	5,075	5,039	5,344	4,653	4,558	4,560	4,889	4,747	4,606	4,629	4,446	4,198	-1.07	0.0499
ENSMUSG00000035152	Ap2b1	adaptor-related	protein_coding	8,579	8,656	8,745	8,115	7,952	7,929	8,342	8,202	8,117	7,986	7,878	7,482	-1.05	0.0586
ENSMUSG00000056296	Synpr	synaptoporin	protein_coding	1,539	1,544	1,741	1,363	1,096	1,302	1,366	1,309	1,431	1,340	1,150	994	-1.22	0.00738
ENSMUSG00000031029	Eif3f	eukaryotic tran	protein_coding	2,738	3,038	3,314	2,456	2,306	2,565	2,629	2,561	2,612	2,541	2,218	2,164	-1.15	0.0262
ENSMUSG00000022010	Tsc22d1	TSC22 domain f	protein_coding	7,088	7,513	7,782	6,631	6,518	6,698	6,973	6,761	6,860	6,555	6,133	5,887	-1.08	0.0981
ENSMUSG00000047215	Rpl9	ribosomal prote	protein_coding	6,099	6,620	7,126	5,658	5,247	5,416	6,228	5,800	5,788	5,656	4,764	4,570	-1.12	0.108
ENSMUSG00000000693	Loxl3	lysyl oxidase-lik	protein_coding	122	224	135	155	128	131	137	127	118	106	119	123	-1.19	0.0965
ENSMUSG00000114147	Gm18939	predicted gene,	transcribed_processe	0	112	5	42	1	3	1	8	1	0	0	0	-12.2	0.0321
ENSMUSG00000028889	Yrdc	yrdC domain co	protein_coding	345	391	363	425	294	371	347	343	278	308	280	340	-1.12	0.124
ENSMUSG00000054836	Elp6	elongator acety	protein_coding	257	307	263	269	168	236	250	254	212	203	190	212	-1.2	0.0306
ENSMUSG00000053025	Sv2b	synaptic vesicle	protein_coding	2,298	2,412	2,463	2,212	1,977	2,247	2,292	2,084	2,168	2,044	2,149	1,931	-1.09	0.052
ENSMUSG00000056153	Socs6	suppressor of c	protein_coding	788	867	886	751	701	790	796	704	748	701	667	642	-1.1	0.0625
ENSMUSG00000060780	Lrrtm1	leucine rich rep	protein_coding	808	916	884	854	742	722	850	802	744	730	755	680	-1.11	0.0537
ENSMUSG00000023169	Slc38a1	solute carrier fa	protein_coding	11,886	12,463	12,325	11,720	11,089	11,485	12,038	11,663	11,683	11,324	11,584	10,955	-1.05	0.0359
ENSMUSG00000039262	Prrc2b	proline-rich coil	protein_coding	19,481	20,385	20,470	19,745	18,856	19,182	20,092	19,546	19,505	19,041	18,960	18,309	-1.03	0.111
ENSMUSG00000026999	Nup35	nucleoporin 35	protein_coding	363	361	353	316	274	357	335	346	299	273	281	264	-1.06	0.338
ENSMUSG00000012405	Rpl15	ribosomal prote	protein_coding	2,903	3,221	3,085	2,923	2,652	3,017	2,972	3,054	2,664	2,580	2,270	2,500	-1.04	0.508
ENSMUSG00000026374	Tsn	translin	protein_coding	2,875	2,900	2,916	2,745	2,474	2,783	2,913	2,740	2,641	2,582	2,265	2,404	-1.05	0.252
ENSMUSG00000024074	Crim1	cysteine rich tra	protein_coding	3,662	3,865	3,681	3,392	3,272	3,163	3,590	3,348	3,318	2,996	3,144	3,118	-1.09	0.0737
ENSMUSG00000031974	Abcb10	ATP-binding ca	protein_coding	1,070	1,119	1,061	971	957	986	1,070	1,035	948	914	881	896	-1.04	0.419

ENSMUSG00000027602	Map1lc3a	microtubule-as	protein_coding	2,227	2,194	2,322	1,867	1,865	1,757	1,804	1,892	1,699	1,956	1,731	1,708
ENSMUSG00000013646	Sh3bp5l	SH3 binding do	protein_coding	929	1,018	1,021	892	856	867	859	932	849	838	856	829
ENSMUSG00000014349	Ube2z	ubiquitin-conju	protein_coding	2,566	2,640	2,691	2,338	2,297	2,340	2,323	2,451	2,361	2,282	2,302	2,216
ENSMUSG00000029405	G3bp2	GTPase activati	protein_coding	12,783	12,876	13,178	11,924	11,891	11,840	11,841	12,184	11,719	11,659	11,849	11,313
ENSMUSG00000004383	Large1	LARGE xylosyl-	protein_coding	1,974	1,999	1,974	1,776	1,736	1,730	1,816	1,909	1,715	1,729	1,805	1,687
ENSMUSG00000035764	Fbxo45	F-box protein 4	protein_coding	1,493	1,462	1,457	1,318	1,332	1,324	1,325	1,382	1,250	1,267	1,327	1,271
ENSMUSG00000017639	Rab11fip4	RAB11 family in	protein_coding	8,550	8,694	9,027	8,135	7,919	7,661	7,909	8,121	7,650	7,944	7,661	7,885
ENSMUSG00000042604	Kcna4	potassium volta	protein_coding	707	730	769	658	582	592	581	627	550	602	542	590
ENSMUSG00000003721	Insig2	insulin induced	protein_coding	1,325	1,513	1,456	1,242	1,066	1,079	1,134	1,152	1,074	1,152	1,033	991
ENSMUSG00000046791	Riox1	ribosomal oxyg	protein_coding	227	242	242	200	168	172	185	205	157	174	170	161
ENSMUSG00000072437	Nanos1	nanos C2HC-typ	protein_coding	1,167	1,226	1,229	1,039	991	994	1,032	1,049	973	995	978	988
ENSMUSG00000041040	Fam117b	family with seq	protein_coding	2,868	2,894	3,052	2,841	2,775	2,601	2,701	2,742	2,650	2,746	2,685	2,556
ENSMUSG00000049657	Zbtb5	zinc finger and	protein_coding	440	444	446	418	393	364	391	412	378	379	381	367
ENSMUSG00000055943	Emc7	ER membrane p	protein_coding	3,564	3,537	3,671	3,379	3,179	3,195	3,254	3,418	3,211	3,288	3,213	3,084
ENSMUSG00000007944	Ttc9b	tetratricopeptic	protein_coding	563	596	573	483	523	510	629	535	460	445	467	439
ENSMUSG00000020376	Rnf130	ring finger prot	protein_coding	4,638	4,864	4,905	4,806	4,548	4,546	5,107	4,835	4,460	4,626	4,217	4,258
ENSMUSG00000023367	Tmem176a	transmembrane	protein_coding	850	870	707	701	805	801	919	666	694	697	594	606
ENSMUSG00000002602	Axl	AXL receptor ty	protein_coding	1,506	1,631	1,430	1,594	1,567	1,571	1,581	1,589	1,471	1,400	1,135	1,131
ENSMUSG00000043257	Pigv	phosphatidylinc	protein_coding	313	398	281	338	350	321	354	310	290	254	214	224
ENSMUSG00000109523	Gdf1	growth differen	protein_coding	52	0	95	0	0	83	50	0	0	0	0	0
ENSMUSG00000058135	Gstm1	glutathione S-tr	protein_coding	5,333	4,857	5,477	4,720	5,521	4,709	5,041	4,862	4,430	4,641	4,567	4,353
ENSMUSG00000028029	Aimp1	aminoacyl tRNA	protein_coding	919	849	897	818	829	879	895	830	795	812	747	770
ENSMUSG00000090946	Ccdc71l	coiled-coil dom	protein_coding	562	454	551	468	500	453	554	478	437	424	402	415

ENSMUSG00000027602	-1.18	0.00292	0.0782		-1.21	0.000347	0.00846	down	-1.03	0.563	1	7	2397	1.43056425	1.28895632	1.81937409	-0.20341168	-0.21357165	-0.75867924	-0.5209237	-0.08146723	-1.06861251	0.22657431	-0.89939045	-1.01941252
ENSMUSG00000013646	-1.1	0.0374	0.237		-1.14	0.00176	0.0236	down	-1.04	0.313	1	7	2398	0.54602915	1.82986083	1.86352733	-0.03243914	-0.59721084	-0.42708939	-0.5453446	0.58266132	-0.710085	-0.88563964	-0.59303546	-1.03123455
ENSMUSG00000014349	-1.09	0.0225	0.192		-1.12	0.00203	0.0259	down	-1.03	0.438	1	7	2399	1.10062547	1.56533022	1.87223547	-0.39547336	-0.68020132	-0.38748453	-0.50006447	0.35984465	-0.2412956	-0.78547929	-0.64675248	-1.26128476
ENSMUSG00000029405	-1.06	0.0349	0.232		-1.09	0.0025	0.0297	down	-1.03	0.365	1	7	2400	1.24382675	1.40259961	1.91050349	-0.27896353	-0.33935266	-0.43259386	-0.43076343	0.1939595	-0.65731644	-0.76935985	-0.41588157	-1.42665801
ENSMUSG00000004383	-1.07	0.0588	0.292		-1.11	0.00299	0.0333	down	-1.04	0.292	1	7	2401	1.34462936	1.54614062	1.337321	-0.3770734	-0.74708753	-0.80374411	-0.01258119	0.79867741	-0.94432961	-0.81487026	-0.11935581	-1.20772647
ENSMUSG00000035764	-1.07	0.0755	0.327		-1.12	0.00145	0.021	down	-1.05	0.168	1	7	2402	1.72686819	1.37349696	1.31628165	-0.39046195	-0.21618459	-0.30855174	-0.30035811	0.41674614	-1.27733343	-1.05700299	-0.27567644	-1.00782371
ENSMUSG00000017639	-1.09	0.00952	0.128		-1.1	0.00214	0.027	down	-1.02	0.642	1	7	2403	1.0459458	1.35904431	2.0647728	0.11387606	-0.38957909	-1.01111424	-0.41480043	0.08230071	-1.03790692	-0.33195302	-1.01073132	-0.46985467
ENSMUSG00000042604	-1.2	0.000368	0.0363	down	-1.25	0.00000642	0.000749	down	-1.04	0.39	1	7	2404	1.1081475	1.39797754	1.87029102	0.46723294	-0.61434172	-0.46394169	-0.63660772	0.0382929	-1.11072247	-0.32878106	-1.22981204	-0.4977352
ENSMUSG00000003721	-1.25	0.0000429	0.0204	down	-1.3	0.000000815	0.000231	down	-1.04	0.43	1	7	2405	0.8867831	1.88987569	1.59957232	0.40469393	-0.71583002	-0.63293042	-0.26988845	-0.15404841	-0.66552201	-0.15241085	-0.94490064	-1.24539423
ENSMUSG00000046791	-1.24	0.00282	0.0767		-1.37	0.00000472	0.000638	down	-1.11	0.142	1	7	2406	1.15324493	1.58365645	1.58666715	0.31438377	-0.76652345	-0.65408524	-0.20255492	0.47071525	-1.17500104	-0.58027535	-0.70151224	-1.02871531
ENSMUSG00000072437	-1.15	0.00629	0.106		-1.18	0.000534	0.0113	down	-1.03	0.493	1	7	2407	1.19303435	1.76037442	1.78847278	-0.1371723	-0.67491139	-0.63629116	-0.22025449	-0.03167136	-0.8815811	-0.63003235	-0.81923673	-0.71073066
ENSMUSG00000041040	-1.08	0.0152	0.161		-1.1	0.00229	0.0281	down	-1.02	0.559	1	7	2408	0.80145615	0.98233843	2.06097083	0.61056009	0.13321111	-1.16616038	-0.40663077	-0.10607213	-0.79240383	-0.07228023	-0.52555351	-1.51943575
ENSMUSG00000049657	-1.12	0.0295	0.216		-1.16	0.00206	0.0263	down	-1.04	0.414	1	7	2409	1.29889884	1.42055778	1.46016518	0.57453455	-0.25956449	-1.26640249	-0.30272445	0.39658057	-0.7634435	-0.72886447	-0.66529035	-1.16444717
ENSMUSG00000055943	-1.08	0.0154	0.162		-1.11	0.00221	0.0276	down	-1.02	0.541	1	7	2410	1.27584056	1.13304122	1.82815665	0.28092755	-0.85322143	-0.75970432	-0.42238239	0.49173238	-0.6699125	-0.22970675	-0.65781919	-1.41695177
ENSMUSG00000007944	-1.01	0.898	0.968		-1.22	0.00132	0.0199	down	-1.21	0.00183	1	8	2411	0.74072648	1.23271877	0.8849265	-0.55139812	0.11555068	-0.1030013	1.70182867	0.30133264	-0.95674412	-1.21975186	-0.82071115	-1.3254772
ENSMUSG00000020376	-1.01	0.781	0.923		-1.09	0.00571	0.0496	down	-1.08	0.0126	1	8	2412	-0.02218497	0.80600751	0.95307098	0.59482099	-0.36274504	-0.37074875	1.65665656	0.70105909	-0.70366091	-0.0683685	-1.67628995	-1.50761701
ENSMUSG00000023367	1.02	0.767	0.917		-1.2	0.00277	0.0316	down	-1.23	0.000865	1	8	2413	1.02475263	1.19731081	-0.29871402	-0.35230919	0.63293154	0.59928794	1.60081326	-0.71097341	-0.42665103	-0.39297211	-1.50833027	-1.36514615
ENSMUSG00000002602	1.02	0.7	0.887		-1.2	0.00329	0.0354	down	-1.23	0.00085	1	8	2414	0.26031562	0.90027077	-0.15797038	0.71414299	0.57673258	0.60001233	0.64777624	0.68835828	0.06727685	-0.32434605	-1.97348922	-1.99907999
ENSMUSG00000043257	1	0.961	0.989		-1.35	0.00119	0.0186	down	-1.36	0.000941	1	8	2415	0.22008828	1.58166373	-0.37102394	0.64927536	0.83587965	0.35919127	0.91392021	0.16750817	-0.20186518	-0.88203373	-1.75023297	-1.52237083
ENSMUSG00000109523	-1.11	0.947			-240	0.001	0.0168	down	-217	0.00124	1	8	2416	1.08798336	-0.66807575	1.67278438	-0.66807575	-0.66807575	1.52577034	1.05806796	-0.66807575	-0.66807575	-0.66807575	-0.66807575	-0.66807575
ENSMUSG00000058135	-1.01	0.746	0.909		-1.13	0.00116	0.0184	down	-1.12	0.00337	1	8	2417	1.16725101	-0.01360422	1.50427233	-0.37360402	1.60729688	-0.4051629	0.45533823	-0.00069987	-1.1715538	-0.58812179	-0.78877317	-1.39263868
ENSMUSG00000028029	-1.02	0.701	0.888		-1.11	0.00573	0.0497	down	-1.1	0.0158	1	8	2418	1.51148955	0.24974575	1.11715702	-0.33299211	-0.11154356	0.7999878	1.09505192	-0.09760808	-0.77933806	-0.44772218	-1.73928304	-1.26494502
ENSMUSG00000090946	-1.03	0.654	0.865		-1.21	0.000602	0.0121	down	-1.18	0.00256	1	8	2419	1.52231487	-0.3439562	1.35490474	-0.08132081	0.49715743	-0.36040812	1.39087503	0.09454031	-0.67847838	-0.92125044	-1.36175103	-1.1126274
