# Supplementary material for: Weight loss aggravates obesity-induced hypothalamic inflammation in mid-aged mice
Source: GeroScience. 2025 Oct 17;48(4):5329–42. doi: 10.1007/s11357-025-01933-x (PMC13575038; doi:10.1007/s11357-025-01933-x)
Supplement: Supplementary file 8 [file 11357_2025_1933_MOESM8_ESM.pdf]

Supplemental table S4

Mid aged hypothalami KEGG pathways

	Overlapping genes	Percentage of changed genes	Adjusted P-value
Parkinson disease	37/249	14.86	1.5991978964679698E-12
Prion disease	36/273	13.19	7.590195460486483E-11
Oxidative phosphorylation	24/133	18.05	4.850765470128784E-10
Huntington disease	36/306	11.76	1.1257268903987683E-9
Pathways of neurodegeneration	44/475	9.26	1.519335649516149E-8
Amyotrophic lateral sclerosis	37/364	10.16	2.6925896140360836E-8
Non-alcoholic fatty liver disease	23/155	14.84	3.3836591015647834E-8
Ribosome	23/158	14.56	4.3529759198135816E-8
Diabetic cardiomyopathy	25/203	12.32	2.5453131731948394E-7
Alzheimer disease	34/369	9.21	9.611151192276847E-7
Thermogenesis	24/232	10.34	1.1429371668208388E-5
Retrograde endocannabinoid signaling	18/148	12.16	2.8035964895516726E-5
Coronavirus disease	23/232	9.91	3.511793871377748E-5
Vibrio cholerae infection	10/50	20.00	6.572758668345648E-5
Salmonella infection	23/249	9.24	1.0090689089411308E-4
Protein processing in endoplasmic reticulum	17/171	9.94	6.13676917896875E-4
Glycolysis / Gluconeogenesis	10/67	14.93	8.022505842935701E-4
Phagosome	15/152	9.87	0.001683424
Vasopressin-regulated water reabsorption	07/44	15.91	0.006520571
Endocytosis	19/252	7.54	0.006899784
Synaptic vesicle cycle	09/78	11.54	0.010981511546577808
RNA transport	15/186	8.06	0.011901998670108155
N-Glycan biosynthesis	07/50	14.00	0.011901998670108155
Collecting duct acid secretion	05/27	18.52	0.016505806
Autophagy	12/137	8.76	0.016505806
Epithelial cell signaling in Helicobacter pylori infection	08/70	11.43	0.018144784424553764
HIF-1 signaling pathway	10/109	9.17	0.026280416365842274
Bacterial invasion of epithelial cells	08/77	10.39	0.030925364
Proteasome	06/46	13.04	0.03111624
mTOR signaling pathway	12/154	7.79	0.036392459
Cortisol synthesis and secretion	07/65	10.77	0.040816803
Longevity regulating pathway	9/102	8.82	0.046177619873792874
Mitophagy	07/68	10.29	0.049202567

Young hypothalami KEGG pathways

	Overlapping genes	Percentage of changed genes	Adjusted P-value
Insulin signaling pathway	13/137	9.49	2.3218886596153645E-4
Neurotrophin signaling pathway	12/119	10.08	2.3218886596153645E-4
Dopaminergic synapse	12/132	9.09	4.5998615046183625E-4
Lipid and atherosclerosis	15/215	6.98	6.441239928866919E-4
Amphetamine addiction	08/69	11.59	0.00175691
Insulin resistance	9/108	8.33	0.006607123
Estrogen signaling pathway	10/137	7.30	0.007172165
Kaposi sarcoma-associated herpesvirus infection	12/193	6.22	0.007172165
Human cytomegalovirus infection	13/225	5.78	0.007297474
Signaling pathways regulating pluripotency of stem cell	10/143	6.99	0.007378476
Growth hormone synthesis, secretion and action	9/119	7.56	0.007473632
Axon guidance	11/182	6.04	0.010499673
Longevity regulating pathway	8/102	7.84	0.010499673
Apelin signaling pathway	9/137	6.57	0.016007478326142174
Colorectal cancer	07/86	8.14	0.016007478326142174
Tight junction	10/169	5.92	0.016971707441899155
Focal adhesion	11/201	5.47	0.017403907
Diabetic cardiomyopathy	11/203	5.42	0.017558078
Long-term potentiation	06/67	8.96	0.017558078
Prion disease	13/273	4.76	0.019736277322445303
Circadian entrainment	07/97	7.22	0.021519642230805252
Prostate cancer	07/97	7.22	0.021519642230805252
Oxytocin signaling pathway	9/154	5.84	0.021519642230805252
cAMP signaling pathway	11/216	5.09	0.021519642230805252
Non-alcoholic fatty liver disease	9/155	5.81	0.021519642230805252
Regulation of actin cytoskeleton	11/218	5.05	0.021604731326235044
Relaxin signaling pathway	8/129	6.20	0.02274885
Chronic myeloid leukemia	06/76	7.89	0.02274885
C-type lectin receptor signaling pathway	7/104	6.73	0.023353882263573496
T cell receptor signaling pathway	7/104	6.73	0.023353882263573496
Hepatitis B	9/162	5.56	0.023353882263573496
Hippo signaling pathway	9/163	5.52	0.023569705
Parathyroid hormone synthesis, secretion and action	7/106	6.60	0.024188684211157985
Alzheimer disease	15/369	4.06504065	0.024233355988527307
cGMP-PKG signaling pathway	9/167	5.389221557	0.024233355988527307
Autophagy	8/137	5.839416058	0.024233355988527307
Yersinia infection	8/137	5.839416058	0.024233355988527307
Fluid shear stress and atherosclerosis	8/139	5.755395683	0.025757209662296634
Aldosterone-regulated sodium reabsorption	04/37	10.81081081	0.027408099
ErbB signaling pathway	06/85	7.058823529	0.027686810428600727
Cholinergic synapse	7/113	6.194690265	0.027686810428600727
Leukocyte transendothelial migration	7/114	6.140350877	0.028354276914465768
Human immunodeficiency virus 1 infection	10/212	4.716981132	0.032405794
Sphingolipid signaling pathway	7/119	5.882352941	0.034110154
Pathways of neurodegeneration	17/475	3.578947368	0.036686998
GnRH signaling pathway	06/93	6.451612903	0.036829921
Alcoholism	9/186	4.838709677	0.036829921
IL-17 signaling pathway	06/94	6.382978723	0.036829921
TGF-beta signaling pathway	06/94	6.382978723	0.036829921
Renal cell carcinoma	05/69	7.246376812	0.040668074429793644
Phosphatidylinositol signaling system	06/97	6.18556701	0.040668074429793644
Chemokine signaling pathway	9/192	4.6875	0.040668074429793644
Prolactin signaling pathway	05/70	7.142857143	0.040668074429793644
Aldosterone synthesis and secretion	06/98	6.12244898	0.040668074429793644
Pathways in cancer	18/531	3.389830508	0.040964442
AGE-RAGE signaling pathway in diabetic complications	6/100	6	0.043088308
Melanogenesis	6/101	5.940594059	0.043886879
Ras signaling pathway	10/232	4.310344828	0.043886879
Vascular smooth muscle contraction	7/133	5.263157895	0.045541846596865616
Wnt signaling pathway	8/166	4.819277108	0.045807181
Glioma	05/75	6.666666667	0.046248289831599294
Thyroid hormone synthesis	05/75	6.666666667	0.046248289831599294
